# Evaluation of MassFrontier, MetFrag, MS-FINDER, and SIRIUS for Metabolite Annotation Using an Experimental LC–HRMS Dataset

**DOI:** 10.3390/biomedicines14040872

**Published:** 2026-04-10

**Authors:** Dmitrii A. Leonov, Irina A. Mednova, Alexander A. Chernonosov

**Affiliations:** 1 Institute of Chemical Biology and Fundamental Medicine, Siberian Branch of Russian Academy of Sciences, Lavrentyev Avenue 8, 630090 Novosibirsk, Russia; d.leonov1@g.nsu.ru; 2 Department of Physics, Novosibirsk State University, Pirogova Str. 2, 630090 Novosibirsk, Russia; 3 Mental Health Research Institute, Tomsk National Research Medical Center, Russian Academy of Sciences, Aleutskaya Str. 4, 634014 Tomsk, Russia; irinka145@yandex.ru

**Keywords:** untargeted metabolomics, mass spectrometry, in silico annotation, biomarker discovery, PRM, SIRIUS, MetFrag, MS-FINDER, MassFrontier

## Abstract

**Background**: Untargeted metabolomics enables comprehensive profiling of biological systems, but accurate metabolite annotation remains a critical bottleneck due to incomplete spectral libraries and structural isomerism. The use of in silico annotation tools can increase the coverage of annotated compounds, but it remains unclear whether these tools, in the absence of reference standards, can reliably annotate real-world experimental LC-HRMS data and whether they are sufficient for this task. **Methods**: This study assesses the performance and limitations of four widely used in silico structure prediction tools (MassFrontier, MetFrag, MS-FINDER, and SIRIUS/CSI:FingerID) when applied to an experimentally acquired feature set previously used to differentiate patients with depressive disorders from healthy controls. To ensure uniform evaluation across tools under realistic but optimized conditions, the quality of MS/MS data was improved using a parallel reaction monitoring method, allowing acquisition of interpretable fragmentation spectra for 26 of the 28 detected features. **Results**: For most features, all tools were able to suggest structure candidates. However, none of the tools proved sufficient as a standalone solution for reliable metabolite annotation. Due to their different algorithms, each tool had strengths and weaknesses in fragmentation interpretation, candidate generation, and ranking, resulting in incomplete or inconsistent annotations. While the combined application of all four tools provided a substantial improvement in putative annotation over conventional spectral library matching, the in silico structure prediction tools often prioritized chemically implausible, biologically irrelevant, or artifactual candidates. Consequently, manual expert evaluation was required to assess the chemical plausibility and biological relevance of the proposed structures. This ultimately reduced the number of biologically plausible metabolites putatively associated with disease to ten. **Conclusions**: Overall, these results demonstrate that existing in silico annotation tools can substantially support the annotation of experimental metabolomics data, but are insufficient on their own. Reliable identification of metabolites in complex biological matrices still depends on high-quality MS/MS data acquisition, the combined use of complementary tools, and mandatory post-annotation expert curation.

## 1. Introduction

Untargeted metabolomics is widely used for the comprehensive analysis of biological samples and for generating new hypotheses, including the discovery of novel biomarkers associated with various diseases. This approach involves two key tasks: detecting features that distinguish between two or more groups of samples and identifying the corresponding compounds. While advances in instrumentation and data processing have substantially improved feature detection, metabolite annotation remains the principal bottleneck. A critical unresolved issue is whether currently available annotation tools—particularly in silico structure prediction methods—can reliably annotate real experimental untargeted LC–HRMS data and whether they are sufficient for this without extensive manual intervention. The initial stage of annotation typically involves spectral matching against reference databases. However, public spectral libraries [1], such as MassBank [2], GNPS [3], MoNA, NIST, HMDB, and several commercial libraries, cover only a fraction of existing metabolites, particularly those found in complex biological matrices. As a result, many acquired MS/MS spectra lack reliable reference matches [4]. The annotation process is further complicated by the presence of isomers that have the same exact mass and exhibit highly similar fragmentation patterns. Reliable identification of such compounds requires additional orthogonal information, such as retention time or nuclear magnetic resonance data [5].

Moreover, a single metabolite can produce multiple LC–MS features corresponding to various adducts, oligomeric forms, or in-source fragments. Incorrect grouping of these signals may result in misassigned identities and false-positive interpretations [6]. Annotation reliability also strongly depends on the quality of the acquired MS/MS spectra [4], which in turn is influenced by ionization mode, ionization efficiency, analyte abundance, collision energy, and scan type. Moreover, in data-dependent acquisition (DDA) modes commonly used in untargeted workflows, MS/MS spectra are often missing or of insufficient quality for confident identification. When molecular formulas are assigned based on exact mass, they may correspond to numerous structural candidates. However, expanding structural databases to include a large number of possible isomers for a single molecular formula further reduces the probability of correct identification unless orthogonal constraints are applied [7]. Several mass spectrometry vendors also provide commercial libraries such as mzCloud, which contains high-resolution MS^n^ spectra organized into “fragmentation trees.” This approach allows not only the identification of compounds present in the database but also the recognition of structurally related analogs [4]. Software such as Compound Discoverer facilitates spectral matching in mzCloud and performs structure searches in ChemSpider across multiple databases based on exact mass. However, due to the limited compound coverage of mzCloud and the lack of orthogonal information in ChemSpider searches, the proportion of confidently identified metabolites remains relatively low. These limitations raise the question of whether alternative strategies can meaningfully improve annotation coverage for real datasets.

In response, a growing number of commercial and open-access in silico annotation tools have been developed. These tools infer candidate structures either by predicting fragmentation patterns from chemical structures or by matching fragmentation patterns of standards directly to experimental MS/MS spectra. These include commercial solutions such as MassFrontier and freely available programs that rely on different prediction algorithms, including MetFrag [8], MS-FINDER [9], SIRIUS [10], and others [7]. Despite their increasing adoption, the reliability of annotations produced by these tools remains debated [11]. The scores assigned by in silico methods implemented in tools such as CSI:FingerID, MetFrag, or CFM-ID to top-ranked candidate structures are often insufficient to distinguish correct from incorrect annotations [11]. As a result, researchers are increasingly using combinations of in silico tools to extend annotation coverage, implicitly acknowledging that no single method is sufficient. While such multi-tool strategies have become common practice [12,13], their performance depends on the quality of the underlying MS/MS spectra, the databases used, and the researcher’s ability to evaluate the plausibility of the proposed structures [14]. Importantly, systematic comparisons of different in silico annotation tools applied to the same real experimental dataset under consistent conditions remain scarce. Consequently, it is still unclear to what extent these tools genuinely enable metabolite annotation in untargeted metabolomics and where their practical limitations lie.

Therefore, in this work, we compared the performance of four widely used in silico annotation tools (MassFrontier, MetFrag, MS-FINDER, and SIRIUS/CSI:FingerID) in combination with targeted PRM acquisition and manual expert curation. Using a previously acquired serum dataset containing incompletely annotated features and originally analyzed in Compound Discoverer using mzCloud and ChemSpider searches [15], we assessed the strengths, limitations, and complementarity of these in silico annotation tools for metabolite annotation in complex biological matrices. Although limited in scope, this dataset provides a realistic test case for assessing whether current in silico approaches are sufficient for metabolite annotation in complex biological matrices and to what extent expert curation remains indispensable.

## 2. Materials and Methods

### 2.1. Reagents

Acetonitrile of LC–MS grade was obtained from J.T. Baker (Phillipsburg, NJ, USA). LC–MS grade methanol and formic acid were purchased from Sigma-Aldrich (St. Louis, MO, USA). All other reagents were of analytical grade. Deionized water was purified using a Milli-Q system (Millipore, Bedford, MA, USA).

### 2.2. Sample Preparation

Pooled serum samples were obtained from the experimental groups described previously [15]. Briefly, the study included patients with depressive disorders and age-matched healthy volunteers, and all procedures were approved by the local ethics committee. Blood samples were collected in the morning after overnight fasting and processed according to the protocol described in the work [15]. Then a 50 μL aliquot of blood serum was transferred to a 1.5 mL tube, followed by the addition of 50 μL methanol. The mixture was vortexed to homogenize the sample and precipitate serum proteins. Subsequently, 100 μL of acetonitrile was added, and the sample was thoroughly mixed before undergoing sonication in an ultrasonic bath for 10 min. The samples were then shaken on a TS-100C thermomixer (Biosan, Riga, Latvia) for 10 min at 4 °C and centrifuged at 13,000× *g* for 10 min. A 50 μL aliquot of the resulting supernatant was transferred to glass chromatographic vials for UPLC–HRMS analysis. In some cases, an additional sample preparation step was employed to increase analyte concentration. Following the same extraction procedure, samples were subjected to evaporation at 45 °C for 40 min using a CentriVap concentrator (Labconco, Kansas City, MO, USA), reducing the total volume from 200 μL to approximately 70 μL prior to analysis.

### 2.3. Sample Preparation for Sodium Formate Cluster Analysis

For sodium formate cluster analysis, pooled serum samples prepared from the same study groups described in Section 2.2 was used. A stock solution of 4 M NaCl in water was prepared and stored at 4 °C before analysis. On the day of the experiment, it was diluted to 1 M, then serially tenfold diluted to prepare NaCl solutions ranging from 10^−1^ to 10^−7^ M in serum. To achieve the same final concentrations in serum, 5 μL of each tenfold-concentrated NaCl solution was added to 45 μL of pooled blood serum. The mixture was incubated for 30 min at room temperature on a TS-100C thermomixer (Biosan, Latvia) and then subjected to the full sample preparation process described above.

### 2.4. Liquid Chromatography–Tandem Mass Spectrometry

Sample analysis was performed using a Q Exactive HF Orbitrap mass spectrometer (Thermo Fisher Scientific, Bremen, Germany) coupled to a Dionex UltiMate 3000 HPLC system (Thermo Fisher Scientific, Waltham, MA, USA). Chromatographic separation was achieved on a C18 reversed-phase column (2.0 mm × 75 mm, ProntoSil-120-3-C18; EcoNova, Novosibirsk, Russia) maintained at 40 °C.

The mobile phases consisted of (A) water and (B) acetonitrile, each containing 0.1% formic acid (*v*/*v*). Separation was carried out at a flow rate of 0.3 mL/min using the following gradient of phase B: 2% (0–1 min), a linear increase to 98% (1–27 min), held at 98% (27–30 min), returned to 2% (30–30.1 min), and equilibrated at 2% (30.1–33 min).

Three distinct mass spectrometric acquisition methods were employed. Electrospray ionization was operated in positive ion mode throughout, with a spray voltage of 4.2 kV and a capillary temperature of 320 °C.

### 2.5. Full Scan Analysis

For initial analysis, full-scan data-dependent MS^2^ (FS-dd-MS^2^) acquisition was performed at a resolution of 120,000 (FWHM at *m*/*z* 200). Instrument parameters included a scan range of *m*/*z* 67–1000, an automatic gain control (AGC) target of 3 × 10^6^, and a maximum injection time of 100 ms. Precursor ions were fragmented using normalized collision energies of 20, 50, and 100 eV. Subsequent dd-MS^2^ analysis of the most intense precursor ions was performed with five acquisition cycles per full MS scan at a resolution of 15,000 in positive ion mode, using an AGC target of 10^5^ and a maximum injection time of 100 ms.

### 2.6. Targeted Selected Ion Monitoring

For targeted selected ion monitoring (t-SIM), an inclusion list containing 28 target compounds was employed (Table 1). Data acquisition was carried out at a resolution of 120,000 (FWHM at *m*/*z* 200) across a scan range of *m*/*z* 150–2000. The AGC target was set to 1 × 10^6^, with a maximum injection time of 200 ms and a 4 *m*/*z* isolation window. Precursor ions were fragmented using stepped normalized collision energy (NCE) values of 20, 50, and 100 eV. Targeted tandem MS (dd–MS^2^) was subsequently performed in positive ion mode at a resolving power of 15,000, with an AGC target of 1 × 10^5^, a maximum injection time of 100 ms, and five acquisition cycles per scan.

### 2.7. Parallel Reaction Monitoring

Parallel reaction monitoring (PRM) experiments were also conducted using the same inclusion list of 28 target compounds. Data acquisition was performed at a resolution of 120,000 (FWHM at *m*/*z* 200). The fragmentation parameters were optimized using stepped collision energies ranging from 10 to 200 eV, applied in predefined increments (e.g., 10–30 eV, 40–60 eV, etc.). PRM MS/MS acquisition was carried out in positive ion mode at a resolution of 45,000 (FWHM at *m*/*z* 200), with an AGC target of 1 × 10^5^, a maximum injection time of 500 ms, a single acquisition cycle, and a narrow isolation window of 0.4 *m*/*z* to enhance selectivity.

### 2.8. Identification of Biomarker Structures

Compound annotation was performed using four analytical tools that combine comparison of experimental MS/MS spectra with in silico fragmentation algorithms based on different theoretical principles: MassFrontier, MetFrag, MS-FINDER, SIRIUS (CSI:FingerID). Among all evaluated programs, only MassFrontier could process raw experimental data in the .raw format, whereas all other tools required a fragment list and precursor molecular mass.

Potential chemical structures were generated using experimentally obtained fragment ion lists and the molecular masses of each analyte, with the aid of the specified analytical software. Each proposed structure was evaluated by the fragmentation ions it was predicted to produce. The degree of concordance between predicted and experimental spectra, along with the confidence in detected fragment peaks, determined the candidate’s overall score.

#### 2.8.1. MassFrontier

MassFrontier (version 8.0.0, Thermo Fisher Scientific, USA) is a commercial software suite for the interpretation of high-resolution tandem mass spectra. Experimental data in the .raw format were imported into the software and analyzed with a mass error tolerance of 10 ppm. Where applicable, the Joint Component Detection algorithm was applied to isolate spectral components corresponding to individual analytes. Compound identification utilized MassFrontier’s built-in algorithms, including forward/reverse identity searches and hierarchical tree-based spectral matching. The search settings were as follows: tree depth 2, with both mzCloud Reference and mzCloud Autoprocessed enabled.

#### 2.8.2. MetFrag

MetFrag (https://msbi.ipb-halle.de/MetFrag/, last accessed 30 June 2025) [8] is an open-access tool available in both console and web-based formats; the latter was used in this study. The tool requires fragment ion *m*/*z* values and relative intensities, as well as precursor ion mass and ionization mode. Relevant data were uploaded and analyzed with a mass error tolerance of 10 ppm. The settings were as follows: Parent Ion mode for candidate search, search mass tolerance of 10 ppm, MS/MS peak match tolerance of 10 ppm for candidate scoring, and ionization mode [M+H]^+^. Database searches prioritized ChEBI, HMDB, LIPID MAPS, and KEGG, with additional queries performed in PubChem to expand possible structural coverage.

#### 2.8.3. MS-FINDER

MS-FINDER (version 3.60) [9] is an open-source desktop application for Windows, designed to annotate unknown metabolites based on fragmentation data. The input data included a list of precursor *m*/*z* values as well as fragment ions with their relative intensities. Compound identification utilized the 14 embedded spectral libraries within the software, which integrate experimental and computational reference spectra for endogenous and xenobiotic compounds. The software was configured to report the top five hits.

#### 2.8.4. SIRIUS (CSI:FingerID)

SIRIUS (version 6.3.3) [10], which integrates the MSNovelist algorithm for improved structure elucidation [16], is open-access software available free of charge for academic use. The input parameters specified were fragmentation spectra (*m*/*z* values and intensities in .txt format), instrument type, ionization mode, and precursor mass. The settings for formula search were as follows: Orbitrap instrument type, isotope pattern filtering enabled, MS^2^ mass accuracy of 10 ppm, and restriction to formulas from biological compound databases. The ZODIAC algorithm [17] was then applied to post-process the data and refine the confidence scores for the molecular formula. Subsequently, structural annotation was performed using CSI:FingerID [18], which integrates fragmentation tree-based molecular fingerprint prediction with similarity searches across the databases available within the software. All available databases were used.

## 3. Results

As the study dataset, we used an extended set of features previously shown to distinguish patients with depressive disorders from healthy controls [15]. This set included 28 features (Table 1, columns *m*/*z* and Formula), some of which had not been considered previously due to annotation uncertainty. Initial annotation in [15] was performed using Compound Discoverer with spectral matching against mzCloud and exact mass searches in ChemSpider. Of these, four compounds (**1**, **7**, **12** and **15**) were identified using the mzCloud database: betaine, (4S,5S,8S,10R)-4,5,8-trihydroxy-10-methyl-3,4,5,8,9,10-hexahydro-2H-oxecin-2-one, piperine, and 17α-Methyl-androstan-3-hydroxyimine-17β-ol, respectively. Only two of these identifications (**1** and **12**) had similarity scores greater than 87, whereas the other two had scores of 55–60. Compound **13** showed a similarity score of 52, which is relatively low.

The remaining compounds were previously annotated using ChemSpider solely on the basis of exact mass. This outcome reflects the well-known limitation of spectral-library approaches, which typically enable confident annotation for only a small fraction of detected features in untargeted metabolomics experiments [19,20]. It has been reported that, for current LC–MS/MS instrumentation, MS/MS spectral coverage in untargeted experiments is often only about 30–60% [19]. Even when MS/MS spectra are available, only about 10% of detected compounds can typically be identified, leaving a large unannotated fraction that is often referred to as “dark matter” in metabolomics [20].

The lack of annotation may be due either to the absence of a reference MS/MS spectrum in the database or to the absence or poor quality of the experimentally acquired MS/MS spectrum in untargeted analysis. To exclude the latter possibility, we analyzed the mass spectrometric spectra of all compounds in the dataset. We reanalyzed pooled blood serum samples using full-scan data-dependent MS^2^ (FS-dd-MS^2^) acquisition and found that only six of the 28 compounds (**1**, **7**, **8**, **12**, **13** and **15**) had fragmentation spectra. For the remaining compounds, the MS signals were overlapped by other, more intense signals, preventing the acquisition of MS/MS spectra for the metabolites of interest. The incompleteness of initial annotation of this set of compounds provided a suitable basis for a systematic comparison of in silico annotation tools under real experimental conditions, especially after improving the quality of MS/MS spectra. Therefore, the next step was to apply targeted mass spectrometric methods using inclusion lists.

### 3.1. Acquisition of MS/MS Spectra

Two inclusion-list-based techniques are available on the Thermo Scientific Orbitrap [21]: targeted selected ion monitoring (t-SIM) and parallel reaction monitoring (PRM). Both methods involve two steps: detection of a specified *m*/*z* value and acquisition of the corresponding fragmentation spectrum. The difference between them is that, in t-SIM, the precursor-ion spectrum is additionally recorded for verification, whereas in PRM the selected ions are fragmented immediately.

Each analyte was analyzed using both methods with multiple collision energies. The use of multiple collision energies is essential because the optimal collision energy for obtaining an informative fragmentation spectrum differs among metabolites [19,22]. Combining information from spectra acquired at different collision energies can significantly improve identification confidence.

For compounds **5**–**7**, **9**, **18**, **20**, **24**, and **28**, the fragmentation spectra remained insufficiently informative due to low signal intensity. In these cases, the extracts were concentrated and the injection volume used for LC-MS analysis was increased to obtain more intense signals. As a result, fragmentation spectra were obtained for 15 compounds using t-SIM and for 26 compounds using PRM (Figure S1). For the remaining compounds, the main reason for the absence of MS/MS spectra was low signal intensity or the absence of signals in the fragmentation spectrum. These results indicate that PRM is the preferred technique for acquiring fragmentation spectra for blood serum metabolites.

### 3.2. Comparative Analysis of In Silico Annotation Tools

**Figure 1:**
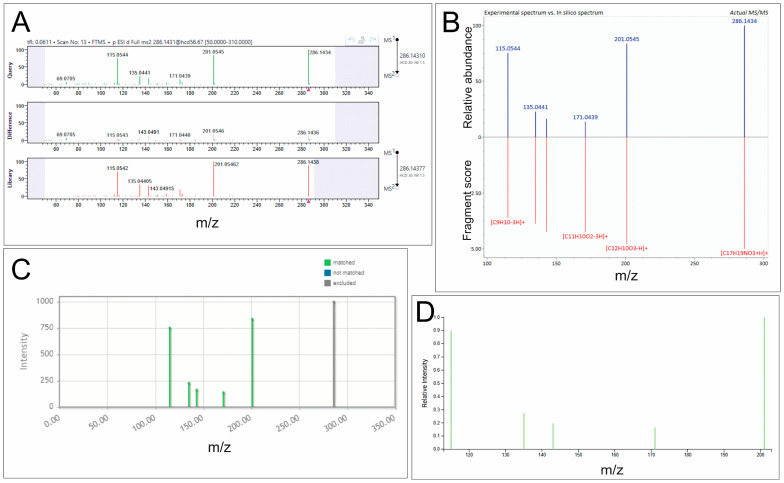
Representative annotation results for the fragmentation spectrum of compound **12** (piperine) obtained using different in silico tools. (**A**) MassFrontier: the upper panel shows the experimental spectrum, the lower panel shows the reference spectrum from the mzCloud library, and the middle panel shows the spectral differences. (**B**) MS-FINDER: the experimental spectrum is shown in the upper panel, and the predicted in silico spectrum for the proposed structure is shown below. (**C**) MetFrag: fragment ions from the experimental spectrum that match the proposed structure are highlighted in green, whereas unmatched signals are shown in gray. (**D**) SIRIUS: fragment ions assigned as consistent with the proposed structure are highlighted in green.

Since different tools use various approaches to structure prediction, we selected representative freely available tools such as MetFrag (https://msbi.ipb-halle.de/MetFrag/, last accessed 30 June 2025), MS-FINDER (version 3.60), SIRIUS (version 6.3.3) [23], and the commercially available software, MassFrontier (version 8.0.0), which allows for processing Orbitrap data in .raw format. The same experimentally acquired fragmentation spectra were processed and evaluated using these tools according to the annotation workflow shown in Figure 1 and Figure S2.

MassFrontier was used for initial processing of the mass spectra, peak alignment and mass list generation. It filtered the raw data for noise and grouped ion signals. The experimental fragmentation spectra were then analyzed both by comparison with reference spectra from the mzCloud Reference database and with in silico spectra (prediction of fragmentation mass spectra based on rules) from the mzCloud Autoprocessed database. Four compounds (**1**, **8**, **12** and **15**) were annotated by MassFrontier using the mzCloud Reference database. For three of these compounds (**1**, **8**, and **12**), the proposed structures were corroborated by other tools. However, MassFrontier failed to generate plausible candidates for most features, reflecting its strong dependence on existing reference spectra and limited ability to propose novel structures for compounds absent from spectral libraries. Notably, using the in silico spectral dataset did not produce any viable candidates for the structures of the compounds (Figure S3).

The MS/MS peak lists generated in MassFrontier, together with their intensities, were then uploaded to MetFrag, MS-FINDER, and SIRIUS in plain-text or web-compatible formats and used for annotation (Table 2). MetFrag is one of the earliest developed in silico annotation tools [19,24], which uses combinatorial bond-breaking to find potential fragment structures matching peaks in the fragmentation spectra [8,25]. The precursor ion mass was employed in the identification of structural candidates, whilst the fragmentation spectra provided a means to evaluate the adequacy of these candidates. Annotation performed by MetFrag was often based on a limited number of fragments, occasionally ignoring other fragments present in the spectra. Nevertheless, MetFrag enabled the annotation of compounds even when fragmentation spectra contained few informative peaks. However, annotation quality was strongly influenced by the choice of database. When restricted to HMDB and ChEBI, MetFrag predominantly proposed biologically plausible metabolites, whereas broader databases increased the likelihood of synthetic or exogenous candidates. As a result, plausible structures were proposed for 18 compounds (Table 1, “MetFrag” column; Figure S3).

In MS-FINDER, the MS/MS peak lists together with their intensities, as well as the precursor *m*/*z* for each compound, were uploaded manually via the “Create new compound” menu. MS-FINDER applies hydrogen rearrangement rules in combination with multiple compound databases to enhance the accuracy of structural predictions. In the first step, possible molecular formulas were calculated based on the measured precursor ion masses. In the second step, candidate structures corresponding to these formulas were generated and ranked according to their spectral matching scores. As a result, putative structures were proposed for 20 compounds (Table 1, see the “MS-FINDER” column, Figure S3). In several cases, MS-FINDER proposed structures not suggested by any other tool, thereby expanding annotation coverage. At the same time, it frequently generated chemically unlikely candidates, including structures requiring implausible hydrogen losses or derived from databases containing non-endogenous compounds. Therefore, MS-FINDER annotations required particularly careful expert curation.

The final tool used for metabolite identification was SIRIUS [10,11,16], which determines molecular formulas by integrating precursor ion *m*/*z*, isotopic patterns from MS^1^ data, and MS^2^ fragmentation spectra. For each compound, we uploaded the MS/MS spectrum and specified the precursor *m*/*z* via the Input Compound interface. SIRIUS then constructs fragmentation trees [26] to model relationships between fragment ions and neutral losses. The CSI:FingerID module predicts probabilistic molecular fingerprints from these trees, which are matched against large structural databases containing over 100 million compounds, such as PubChem. Using SIRIUS, eight compounds were annotated (Table 1, see the “SIRIUS” column, Figure S3), six of which agreed with annotations generated by other tools. In two cases, SIRIUS uniquely suggested plausible candidates. SIRIUS structural predictions were sometimes too general, yielding limited specificity when multiple structures shared similar fingerprints.

Across all tools, full agreement was observed only for a small subset of compounds. Partial agreement between two or three tools was more common, whereas for several features the tools produced either different rankings of the same annotation or entirely distinct candidate sets.

### 3.3. Manual Expert Curation

Despite improved annotation rates, automated rankings frequently included chemically unstable, synthetic, or biologically implausible structures. Although each algorithm ranks structures according to its internal scoring criteria, the top-ranked candidate structures are often chemically unstable, synthetic, or biologically implausible. Therefore, manual expert curation was applied as a mandatory final step, during which automated annotation of the compounds by all four algorithms and the proposed structures for each compound were evaluated and compared. Among the ranked proposed structures, we first considered the top 1–3 candidates proposed by each algorithm and compared them with the rankings of the others. If a proposed structure was a synthetic compound or unlikely to belong to the human metabolome (e.g., drugs or highly hydrophobic compounds), it was excluded from consideration, and the next-highest-ranked structure was analyzed. Additionally, information was sought regarding potential associations between the structure and depressive episodes. The final selected structures are presented in Table 1.

### 3.4. Quality and Quantity of Annotation

The combined use of all four tools enabled putative annotation of 22 of the 28 compounds. For some compounds, structures with aliphatic chains of various lengths (**2**, **5**, **8**, **13**, **15**, **16**, **17**, **18**, **20** and **23**) were suggested (Figure S3). For other compounds, including those annotated by all evaluated in silico annotation tools as well as those suggested by only some of them, multiple structural candidates were considered.

The compounds were annotated at the highest possible level of confidence in MassFrontier. This process involved comparing them to a set of fragmentation spectra from the mzCloud database. However, of the four compounds annotated, only three (**1**, **8**, and **12**) were accepted as plausible; the structure proposed for compound **15** was discarded as a less likely variant than the structure suggested by other tools. It should be noted that MassFrontier was unable to propose structures for most of the analyzed compounds, likely because these metabolites were not represented in mzCloud or other MS/MS databases. However, for the compounds successfully annotated by MassFrontier, the proposed structures were consistently confirmed by the other annotation tools used in this study.

Among other tools, the best annotation quality was achieved by MetFrag for 18 compounds. Its predictions depended on the database from which the candidate structures were obtained. Databases such as HMDB or ChEBI provided more reliable chemical structures. However, when predicting structures, it often used only one or two fragments and did not take other signals on the spectrum into account. The use of larger databases like PubChem, on the one hand, allowed more signals in the fragmentation spectrum to be taken into account, but on the other hand, it suggested synthetic compounds or those of exogenous origin as the most probable structures.

The largest number of candidate structures (20 in total) was proposed using MS-FINDER. No structure variants were proposed for three of these compounds by other tools. Additional variants were generated for nine compounds, and previously proposed structures of eight compounds were confirmed. However, the plausibility of these structures is questionable because they are taken from various sources, such as the UNPD, which often produces structures that are chemically unlikely to be metabolites. Also, when calculating the theoretical fragmentation spectra, MS-FINDER sometimes interprets the signals on the spectrum as fragments involving losses of 5–7 hydrogen atoms.

The combination of FingerID and CANOPUS in SIRIUS provided candidate structures for most compounds (except **21**, **22**, **25**–**29**); however, structures only for eight compounds were selected as plausible. The results of this analysis were compared with those obtained using the other tools, and showed that six of the compounds had the same annotation as other tools, while the other two compounds had been identified by SIRIUS alone.

Because chemical standards were unavailable for most structures, we consider these annotations to correspond to MSI levels 2 and 3. In bounds of level 2 we recognize compounds identified with search in mzCloud as highest level of annotation, then compounds whose one and the same structure was suggested by different in silico algorithms, then structures from MetFrag, MS-FINDER, SIRIUS with descending confidence (MSI level 3).

### 3.5. Relationship of the Annotated Compounds to Depression

To determine which of the proposed structures was actually the most plausible, we carried out a literature search and selected only those compounds for which a link to depression was evident. Thus, compound **1**, annotated as betaine, is essential for the remethylation of homocysteine to methionine [27]. A deficiency in betaine can therefore lead to elevated homocysteine and reduced methionine, impairing the synthesis of monoamine neurotransmitters—dopamine, serotonin, and norepinephrine [28,29]. This impairment is implicated in the pathogenesis of depressive symptoms. Consistent with this, several studies have linked low serum betaine levels to depression [28,30] and have shown that betaine alleviates depressive symptoms in both clinical and preclinical studies [31,32].

Compound **12** (piperine), although an exogenous substance, exhibits antidepressant-like effects in animal models [33,34,35,36]. Piperine is the principal alkaloid of black pepper. However, a recent human study found that piperine levels, like those of 2-aminoheptanoate, were inversely associated with low-fat dairy consumption [37]. This association likely reflects the dietary habits of the study cohort rather than a direct biochemical interaction.

Compound **3**, annotated as 2-oxoarginine, a guanidine compound derived from arginine and proline metabolism, was decreased in our study. This finding is significant within the context of depression pathophysiology, where stress triggers an adaptive shift in arginine metabolism toward polyamine synthesis—the “polyamine stress response” [38,39]. The observed reduction in 2-oxoarginine likely reflects this metabolic diversion, indicating a heightened consumption of arginine for polyamine production.

A significant proportion of the identified metabolites were lipid derivatives, aligning with the established role of lipids in the central nervous system and their implication in the pathophysiology of depression. These molecules influence key processes including neurotransmitter metabolism, neuroinflammation, and oxidative stress, positioning them as potential biomarkers for the disorder [40,41]. We hypothesize that compounds **5**, **8**, **15**, **17**, and **20** may constitute long-chain hydrocarbon lipids. Furthermore, we identified two specific lipids: compound **13** as (4E,8E,10E-d18:3)sphingosine, a sphingolipid, and compound **23** as LysoPC(18:2(9Z,12Z)), a glycerophospholipid. Both classes are fundamental components of biological membranes and act as signaling molecules in cellular transduction pathways [42]. The presence of (4E,8E,10E-d18:3)sphingosine exclusively in the depression cohort supports the concept of aberrant sphingolipid metabolism in this disorder [43,44]. Similarly, the observed alterations in glycerophospholipid metabolism in depression patients, as reflected by changes in LysoPC(18:2(9Z,12Z)), are consistent with existing literature [45,46].

Compound **15**, classified as anandamide, is an endocannabinoid; alterations in the concentration of this metabolite have previously been shown in patients with depressive disorders [47,48]. The higher anandamide levels observed in our study in depressed patients may be a consequence of a compensatory response to the low-grade inflammation often observed in this disorder [49]. In this model, inflammatory stimuli promote endocannabinoid secretion from immune cells as a negative feedback mechanism to dampen the inflammatory response [50].

Compound **19**, annotated as nutriacholic acid, is a bile acid. Previous studies have reported links between altered bile acid profiles and depressive disorders [51,52,53]. Bile acids can influence the pathophysiology of depression by modulating blood–brain barrier permeability and engaging key processes such as neuroinflammation, oxidative-nitrosative stress, and endoplasmic reticulum stress [52,54].

### 3.6. Sodium Formate Clusters

Although fragmentation spectra were obtained for almost all of the compounds **22**, **25**–**29**, none of them were annotated by any tool. These compounds exhibited similar fragmentation patterns, in which higher molecular weight compounds contained molecular ions of lower molecular weight compounds in their fragmentation spectra (Figures S1 and S4). A review of the literature revealed that these *m*/*z* values could correspond to sodium formate cluster ions, formed during electrospray ionization [55,56].

Sodium formate cluster ions are known to occur in LC–MS experiments using formic acid-containing mobile phases [55], and are unlikely to represent direct biological metabolites. However, they were included in the dataset because they were part of the feature set that discriminated between the studied groups of patients [15]. We compared the intensity of sodium formate cluster signals in pure solutions and in blood serum after addition of NaCl. In pure solution, increasing NaCl concentration led to a proportional increase in cluster signal intensity. In contrast, when NaCl was added to blood serum at concentrations ranging from 10^−6^ to 10^−1^ M, the intensity of the clusters remained unchanged. These observations suggest that cluster formation in serum depends not only on sodium concentration, but also on one or more serum components that promote or stabilize these ions. Thus, although compounds **22**, **25**–**28** cannot be interpreted as metabolites and were not informative for structural annotation, they remain of interest as indirect markers associated with sample composition. Their exact origin and analytical significance require further investigation and lie beyond the scope of the present study.

### 3.7. Limitations

The main limitations of our study are the dataset with a limited number of features, not using reference standards for validation due to heterogeneity and unpredictability of the revealed compounds, and set of used in silico tools.

## 4. Discussion

Despite the rapid expansion of publicly available MS/MS spectral libraries, routine library-based approaches still annotate only a limited fraction of detected features in untargeted LC-MS metabolomics [4,18]. A major obstacle is the frequent lack of high-quality fragmentation spectra, since DDA is generally optimized to maximize metabolome coverage rather than to obtain informative MS/MS spectra for each precursor [19,57]. Since the reliability of compound identification is directly linked to the quality of the acquired MS/MS spectra [4], the latter determines whether downstream in silico annotation tools can be applied effectively.

The effectiveness of annotation also depends on the chemical class of the analyzed compound. Substance classes that are well represented in spectral and structural databases and that yield characteristic fragmentation patterns may be predicted more reliably than chemically heterogeneous classes or compounds with less well-defined MS/MS. By contrast, closely related metabolites and structurally similar compounds remain more difficult to distinguish, even when high-quality fragmentation spectra are available.

A separate challenge arises in the case of features that are not direct metabolites. For example, in the studied dataset, a series of ions with high *m*/*z* values (compounds **22** and **25**–**28**) were not annotated by any of the four tools and were later identified as sodium formate cluster ions formed during electrospray ionization [55,56]. Although these ions are non-biological, they were distinguishing features between the patient and healthy control groups, suggesting potential value as indirect biomarkers. Because their signals do not correlate with the amount of sodium chloride added to serum samples, their formation may depend on another, as yet unidentified, component of the serum extract, which requires further investigation.

Nevertheless, the application of in silico fragmentation prediction tools such as MassFrontier, MetFrag, MS-FINDER, and SIRIUS expands the number of annotated compounds compared with conventional database matching alone, showing that in silico tools can improve annotation coverage when suitable MS/MS data are available [1,19].

MassFrontier prioritizes chemically plausible structures but is limited by its libraries and general fragmentation rules [58]. MetFrag and MS-FINDER broaden the search space but may propose biologically irrelevant candidates, reflecting a trade-off be-tween sensitivity and specificity. SIRIUS is effective in determining molecular formulas based on isotope patterns and fragmentation, but it may generate overly generalized structures when fragmentation evidence is insufficient to discriminate among isomers. Ion mobility-mass spectrometry could improve discrimination of isomeric candidates by providing an additional gas-phase separation dimension and collision cross section information. Even when isomers have identical exact masses and similar MS/MS spectra, differences in ion shape and size may result in distinct mobilities, thereby increasing annotation confidence.

Such heterogeneity in annotation underscores that no single algorithm can guarantee the identification of a fully chemically and biologically plausible structure with absolute certainty. However, consensus among multiple tools regarding a single structure substantially increases confidence in the annotation, indicating that combined in silico tools are more effective than individual tools, though still not sufficient on their own. On the other hand, the combined use of multiple tools does not replace, but rather emphasizes, the importance of expert evaluation and manual verification of proposed structures. At least a two-level manual expert curation of annotated compounds is required. At the first level, the chemical plausibility and biological relevance of the proposed structures must be assessed. The second stage involves evaluation of their potential involvement in biochemical pathways, and, when several equally plausible structures are suggested by in silico tools, identifying the most valid one. Nevertheless, by integrating data acquisition, computational an-notation, and validation, such a workflow enhances the transparency, reproducibility, and chemical soundness of metabolite identification in complex biological matrices.

## 5. Conclusions

This study provides an evaluation of four commonly used in silico annotation tools by applying them to an experimental LC–HRMS dataset acquired from plasma samples and assessing their performance against subsequent expert-curated analysis. The application of targeted acquisition methods significantly improved the quality of MS/MS spectra, yielding fragmentation information for nearly all features of interest. PRM was identified as the preferred method for spectral acquisition in the metabolomic analysis of biological matrices, especially for low-abundance metabolites, as it provides MS/MS spectra of sufficient quality for interpretation. These enhanced spectra enabled a rigorous comparison of annotation outputs across MassFrontier, MetFrag, MS-FINDER, and SIRIUS. The side-by-side assessment revealed substantial variability among the tools, indicating that no single tool is sufficient for confident annotation of all experimentally detected features. MetFrag and MS-FINDER produced the highest numbers of putative identifications, while MassFrontier provided the most chemically plausible and biologically relevant structures when cross-validated with the others. SIRIUS offered additional molecular formulas based on fingerprint structure ranking, although the reliability of its structural predictions was low. Several high-mass ions that remained unannotated by all tools were determined to be sodium formate clusters, and, despite their non-biological origin, they could reflect sample-related processes and therefore could be considered indirect biomarkers. Overall, the results show that widely used in silico tools provide complementary but often divergent annotations when applied to real experimental LC–HRMS data, and their outputs alone are insufficient without expert verification. The reliability of the proposed structures therefore must be verified by a two-level expert curation: first, the chemical plausibility and biological relevance of the proposed structures must be assessed; second, their involvement in biochemical pathways should be evaluated to select the most valid candidate structure when multiple in silico suggestions exist. Therefore, the combination of PRM-based improvement of MS/MS spectra, several in silico annotation tools, and expert curation is the most reliable strategy for accurately identifying metabolites and interpreting biologically relevant features in complex biological matrices.

## Figures and Tables

**Table 1:** Results of metabolite annotation obtained using different in silico structure prediction tools (MassFrontier, MetFrag, MS-FINDER, and SIRIUS/CSI:FingerID).

Compound	*m*/*z*	Formula	Name *	Compound Rating **			
				MassFrontier	MetFrag	MS-FINDER	SIRIUS
**1**	117.0786	C_5_H_11_NO_2_	**Betaine**	**1st**	2nd	1st	1st
			5-Aminopentoic acid	-	**1st**	3rd	-
**2**	145.1095	C_7_H_15_NO_2_	**(2R)-Aminoheptanoic acid**	-	1st	2nd	-
			Actinine	-	-	-	1st
**3**	172.0704	C_6_H_10_N_3_O_3_	**2-Oxoarginine**	-	2nd	-	-
		C_8_H_12_O_4_	Diethyl fumarate	-	-	12th	-
**4**	186.0759	C_8_H_12_NO_4_	2-(2-benzofuranyl)-4,5-dihydro-1H-imidazole	-	-	1st	-
**5**	201.1721	C_11_H_23_NO_2_	**11-Aminoundecanoic acid**	-	-	1st	-
**6**	214.1175	C_9_H_16_N_3_O_3_	1-(2-morpholin-4-ium-4-ylacetyl)pyrazolidin-3-one	-	5th	-	-
		C_11_H_18_O_4_	Alpha-carboxy-delta-decdlactone	-	-	5th	-
**7**	216.0964	C_10_H_16_O_5_	Aspyranol	-	-	1st	-
		C_13_H_16_OSi	1-Napthol	-	2nd	-	-
		C_12_H_8_O_4_	Methoxsalen	-	1st	-	-
**8**	269.3070	C_18_H_39_N	**Octadecane-1-amine**	1st	1st	1st	1st
**9**	272.1586	C_18_H_21_FO	(R*,S*)-4-[1-Ethyl-2-(4-fluorop+henyl)butyl]phenol	-	1st	-	-
		C_21_H_20_	1,1,2-Triphenylpropane	-	2nd	-	-
		C_14_H_24_O_5_	UNPD129718	-	-	1st	-
**10**	280.1296	C_15_H_20_O_5_	Crispolide	-	1st	7th	-
			Nigellic acid	-	4th	2nd	-
			13-Hydroxyabscisic acid	-	5th	1st	-
**11**	282.1002	C_14_H_19_O_4_P	8-phenyloct-3-ynyl dihydrogen phosphate	-	1st	-	-
		C_16_H_14_N_2_O_3_	N-(4-ethylphenyl)-5-(2-furanyl)-3-isoxazolecarboxamide	-	-	2nd	-
**12**	285.1351	C_17_H_19_NO_3_	**Piperine**	1st	1st	1st	1st
**13**	295.2490	C_18_H_33_NO_2_	**(4E,8E,10E-d18:3)sphingosine**	-	1st	2nd	-
**14**	306.2391	C_20_H_34_O_2_	4,5,13-Duratriene-1,3-diol;2,7,11-Cembratrien-4,6-diol	-	-	1st	-
		C_20_H_34_O_2_	Sagittariol	-	-	4th	-
**15**	319.2464	C_20_H_33_NO_2_	17beta-Hydroxy-17-methyl-5alpha-androstan-3-one oxime	1st	-	-	-
			**Anandamide (18:4, n-3)**	-	1st	4th	4th
**16**	326.3282	C_20_H_42_N_2_O	(7R)-18-(2-ethylhydrazino)octadic-9-en-7-ol	-	1st	-	-
			Palmitoylputrescine	-	-	1st	4th
			1-Hexadecyl-3-propylurea	-	-	-	1st
**17**	367.4168	C_25_H_53_N	**N,N-dimethyltricosan-1-amine**	-	1st	-	4th
			N-dodecyl-N-methyldodecan-1-amine	-	-	1st	-
**18**	383.1940	C_20_H_25_N_5_O_3_	Ethyl 4-[[1-[(3-ethoxyphenyl)methyl]pyrazolo [3,4-d]pyrimidin-4-yl]amino]butanoate	-	2nd	-	-
**19**	390.2752	C_24_H_38_O_4_	9″-Carboxy-alpha-chromanol	-	1st	-	-
			**Nutriacholic acid**	-	4th	5th	-
**20**	437.4946	C_30_H_63_N	N-decyl-N-ethyl-octadecan-1-amine	-	1st	-	-
**21**	465.5261	C_32_H_67_N	-	-	-	-	-
**22**	498.8993	C_7_H_7_Na_8_O_14_	sodium formate cluster	-	-	-	-
**23**	519.3309	C_26_H_50_NO_7_P	**LysoPC(18:2(9Z, 12Z))**	-	1st	1st	1st
**24**	608.2188	C_27_H_36_N_4_O_10_S	[(2R,3S,4R,5R,6S)-5-acetamido-3,4-diacetoxy-6-[[5-[2-(3,4-dimethoxyphenyl)ethyl]-4-methyl-1,2,4-triazol-3-yl]sulfanyl]tetrahydropyran-2-yl]methyl acetate	-	-	1st	-
**25**	702.8610	C_10_H_10_Na_11_O_20_	sodium formate cluster	-	-	-	-
**26**	838.8346	C_12_H_12_Na_13_O_24_					
**27**	906.8223	C_13_H_13_Na_14_O_26_					
**28**	1232.8473	-	-	-	-	-	-

* Candidate structures potentially associated with depressive disorders are highlighted in bold. ** Annotation numbers assigned in the list of proposed structures.

**Table 2:** Summary of the in silico structure prediction tools used in this study.

Tool	Input	Databases Queried	Primary Output	Role in Workflow
MassFrontier (version 8.0.0)	.raw files	mzCloud, MassFrontier libraries	Fragment lists (*m*/*z* + intensities), candidate structures with scores	Raw processing, noise filtering, isotope/adduct grouping, initial spectral search
MetFrag (last accessed 30 June 2025)	fragments, precursor *m*/*z*	HMDB, ChEBI, LIPIDMAPS, PubChem (optional)	Ranked candidate structures with scores	Candidate generation based on combinatorial bond-breaking and MS^2^ matching.
MS-FINDER (version 3.60)	fragments, precursor *m*/*z*	MS-FINDER internal libraries (e.g., UNPD, HMDB)	Ranked candidates with scores, additional candidate formulas	Broad candidate generation based on hydrogen rearrangement-based predictions.
SIRIUS/CSI:FingerID (version 6.3.3)	fragments, precursor *m*/*z*	PubChem, HMDB, others	Molecular formula, fragmentation trees, fingerprint scores	Formula reassignment via fragmentation trees; fingerprint-based candidate generation.

## Data Availability

The original contributions presented in this study are included in the article/Supplementary Materials. Raw data are uploaded to the Metabolights repository (https://www.ebi.ac.uk/metabolights/MTBLS14115).

## Supplementary Materials

The following supporting information can be downloaded at https://www.mdpi.com/article/10.3390/biomedicines14040872/s1, Figure S1: MS/MS spectra obtained by t-Sim and PRM methods; Figure S2: Schematic of the integrated annotation workflow; Figure S3: Results of compound annotation by different in silico tools; Figure S4: Mass spectrum of sodium formate cluster ions.
