# Supplementary material for: Evaluation of MassFrontier, MetFrag, MS-FINDER, and SIRIUS for Metabolite Annotation Using an Experimental LC–HRMS Dataset
Source: Biomedicines. 2026 Apr 10;14(4):872. doi: 10.3390/biomedicines14040872 (PMC13113853; doi:10.3390/biomedicines14040872)
Supplement: Supplementary file 1 [file biomedicines-14-00872-s001.zip › Fig. S2.pdf]

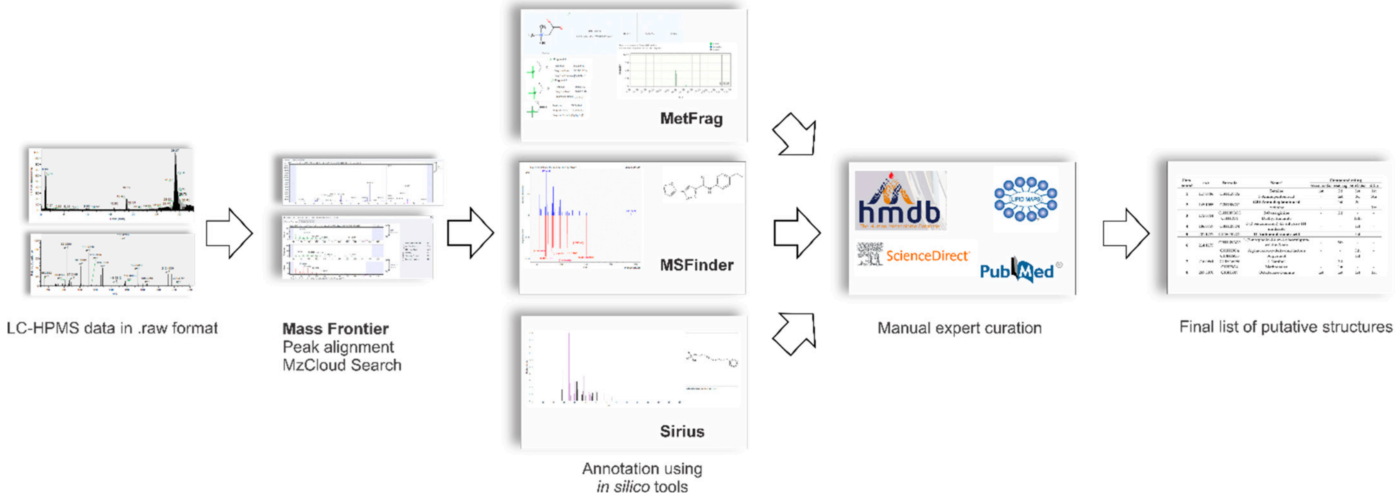

Figure. S2. Schematic of the integrated annotation workflow. Raw .raw files were processed in MassFrontier for noise filtering and isotope/adduct grouping. Fragment lists (m/z + relative intensity) from MassFrontier were exported and submitted to MetFrag, MS-Finder, and SIRIUS/CSI:FingerID. Candidate lists were compiled and cross-referenced; selected top-ranked candidates were subjected to manual expert curation. Final annotations were assigned MSI level 2 and 3 as described in Methods.
