# Supplementary material for: Evaluation of MassFrontier, MetFrag, MS-FINDER, and SIRIUS for Metabolite Annotation Using an Experimental LC–HRMS Dataset
Source: Biomedicines. 2026 Apr 10;14(4):872. doi: 10.3390/biomedicines14040872 (PMC13113853; doi:10.3390/biomedicines14040872)

1) 117.07860 – C5H11NO2 Betaine MassFrontier

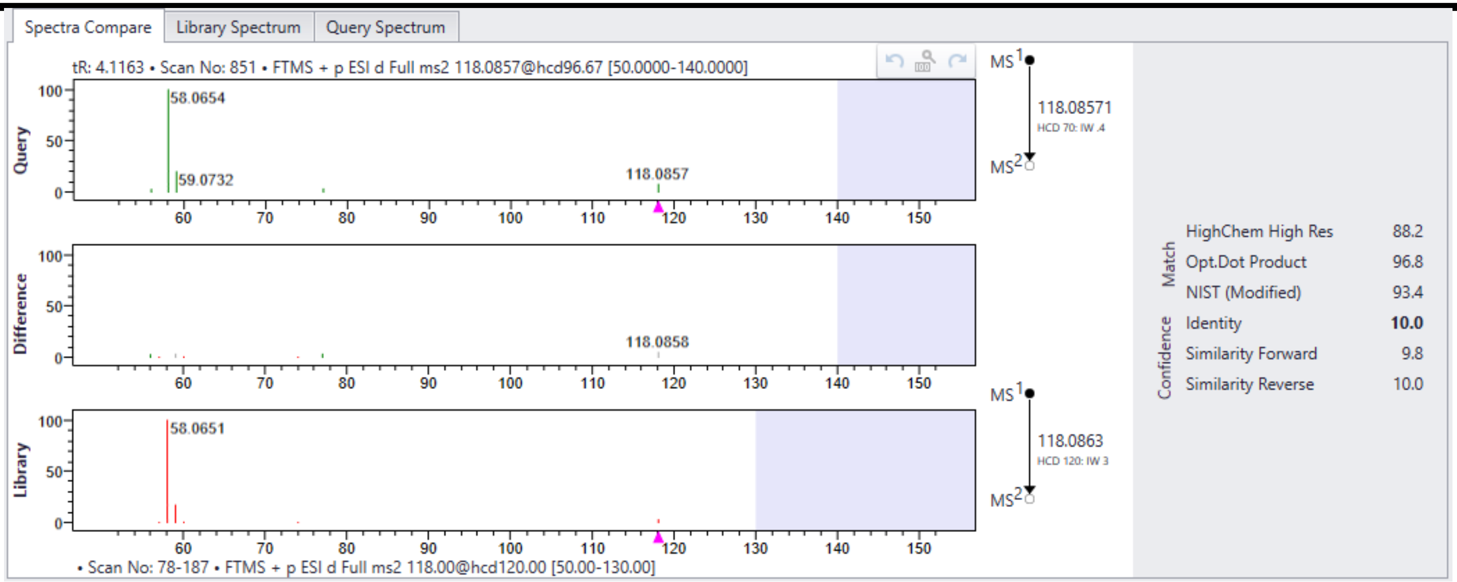

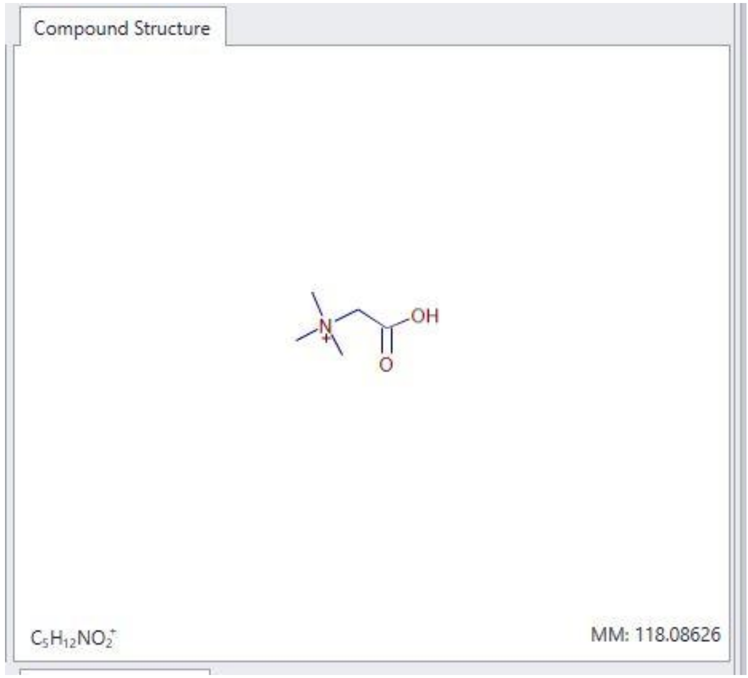

1) 117.07860 – C5H11NO2 MetFrag Betaine

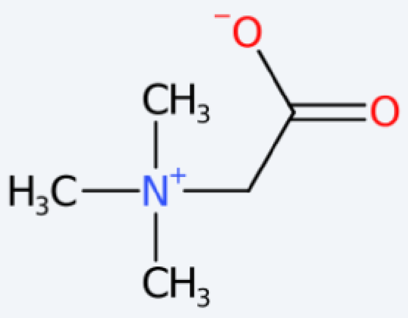

Betaine

HMDB00043<br>InChIKeyBlock1 = KWIUHFFTVRNATP	117.079	$C_5H_{11}NO_2$	0.9822

Select area to zoom in. Double click to return.
Click on apex of explained peak to select fragment.

matched
not matched
excluded

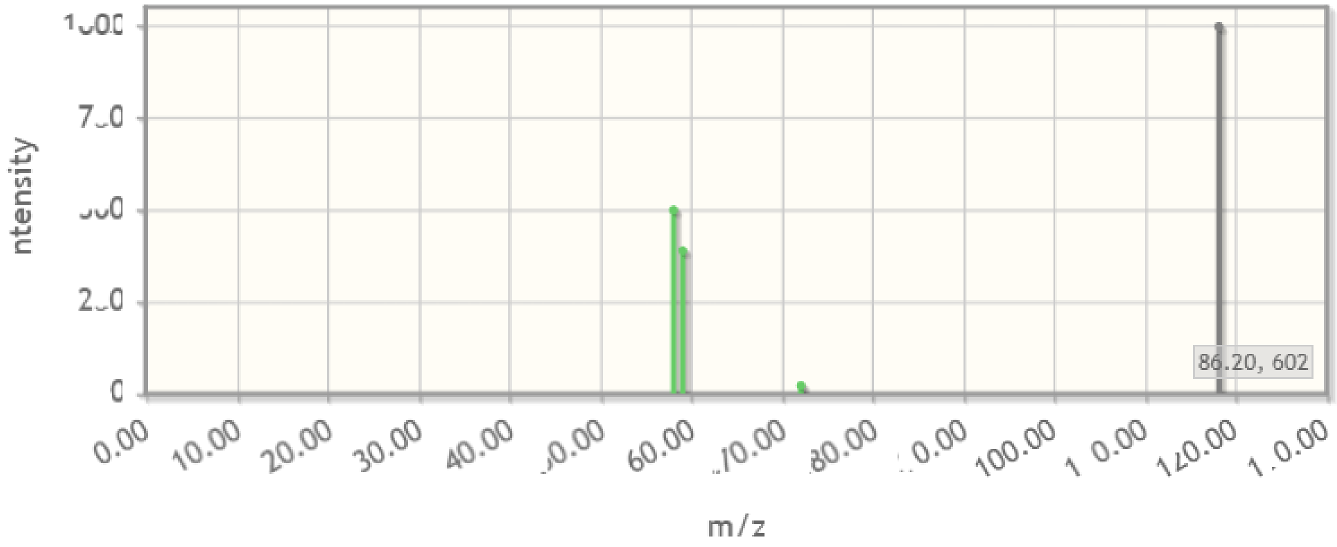

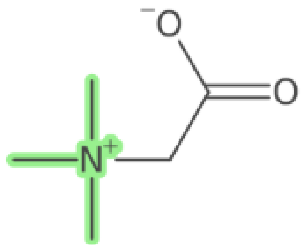

Fragment 1

Peak m/z: 58.065449

Fragment Mass: 58.06516 Da

Fragment Formula: $[C_3H_9N\text{-}H]^+$

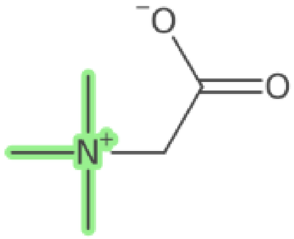

Fragment 2

Peak m/z: 59.073265

Fragment Mass: 59.07299 Da

Fragment Formula: $[C_3H_9N]^+$

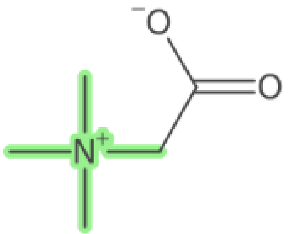

Peak m/z: 72.080902

Fragment Mass: 72.08082 Da

Fragment Formula: $[C_4H_{11}N\text{-}H]^+$

1) 117.07860 – C5H11NO2 MSFinder Betaine \ 5-Aminopentanoic acid

Formula	Error [mD	Error [ppr	Score	Resource	Select
C5H11NO2	0.2550	2.1780	5.2710	HMDB,KNApSA	✓
Spectral DB sea	0.0000	0.0000	5.0000		✓

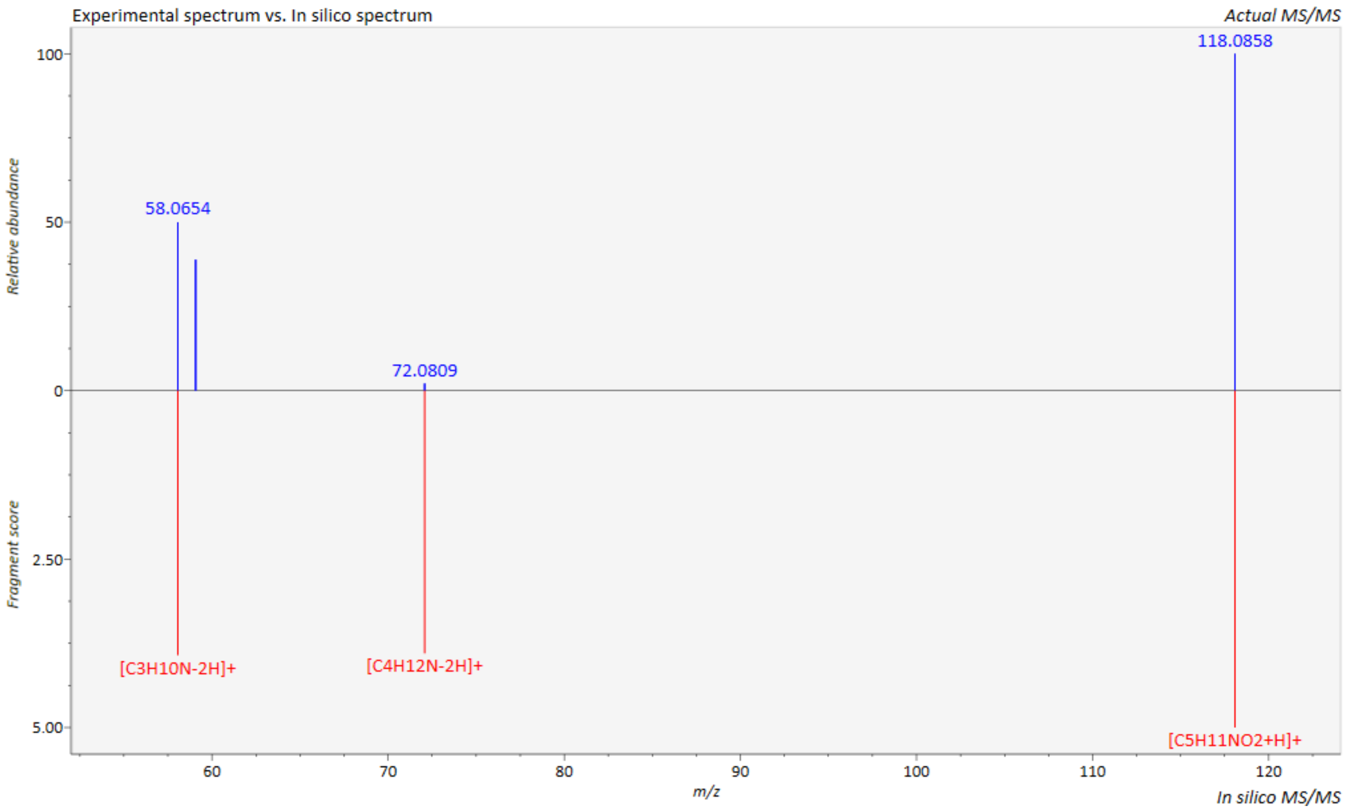

Name	Score (max=10)	Ontology	InChIKey
Betaine	9.41	Alpha amino acids	KWIUHFFTVRNATP-UHFFFAOYSA-N
L-Valine	9.34	Valine and derivatives	KZSNJWFQEVHDMF-SGAVLPGINA-N
5-Aminopentanoic acid	9.08	Delta amino acids and derivatives	JJMDCOVWQOJGCB-UHFFFAOYSA-N
Norvaline	8.90	L-alpha-amino acids	SNDPXSYFESPGGJ-SCSAIBSYSA-N
N,N-dimethyl-beta-alanine	8.60	Trialkylamines	JMOXSQYGVIXBBZ-UHFFFAOYSA-N

1) 117.07860 – C5H11NO2 Sirius Betaine \ 5-Aminopentanoic acid

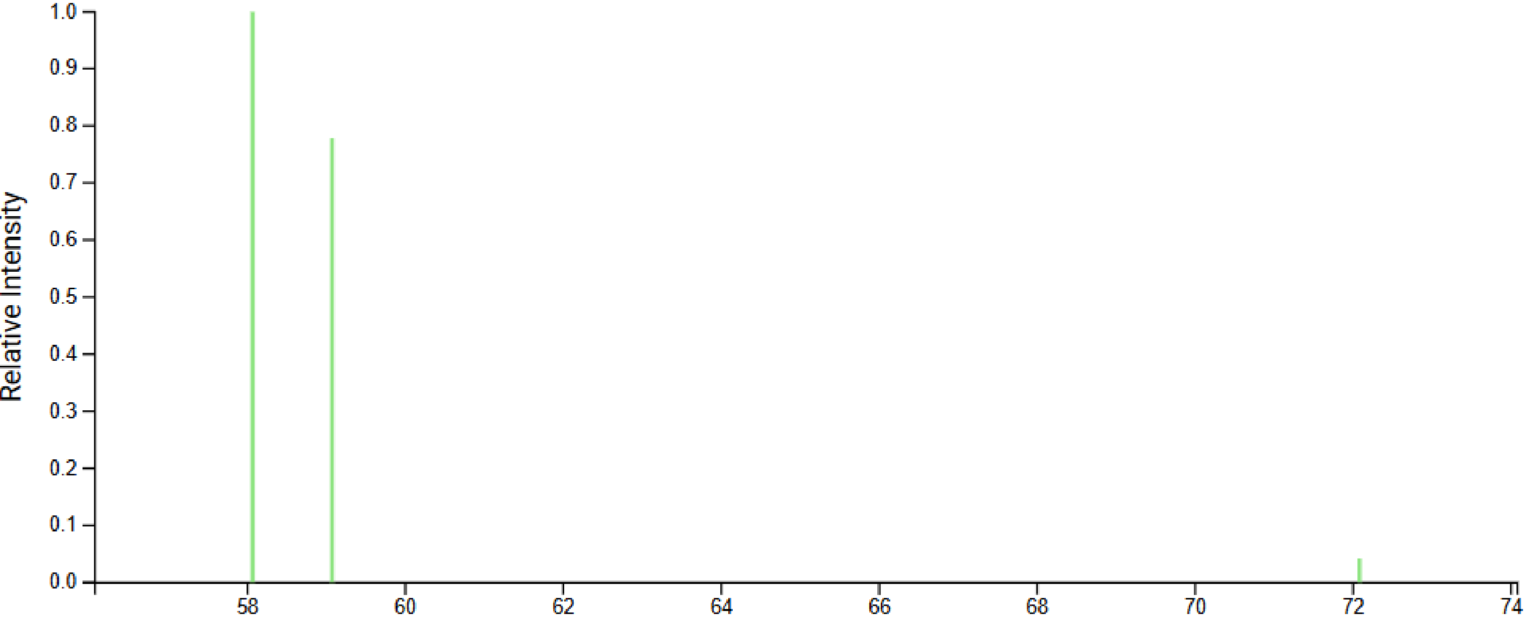

MS2 merged

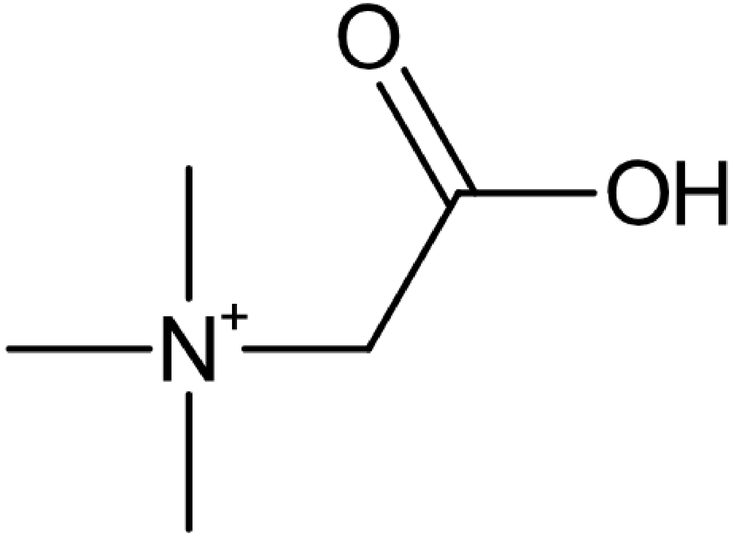

(carboxymethyl)trimethylazanium

Rank	Name	SMILES	Molecular Formula	Adduct	CSI:FingerID Score	Tanimoto Similarity	XLogP	InChIKey	Lipid Class	Database	De Novo
1	(carboxymethyl)tri...	C[N+](C)(C)CC(=O...	C5H11NO2	[M + H]+	-27,463	91,837%	-0.1	KWIUHFFTVRNATP		■	

1) 117.07860 – C5H11NO2 MetFrag 5-Aminopentanoic acid

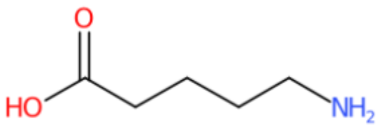

5-Aminopentanoic acid

HMDB03355<br>InChIKeyBlock1 = JJMDCOVWQOJGCB	117.079	$C_5H_{11}NO_2$	1.0

Fragment 1

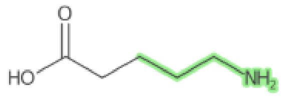

Peak m/z: 58.065449

Fragment Mass: 58.06516 Da

Fragment Formula: $[C_3H_8N]^+$

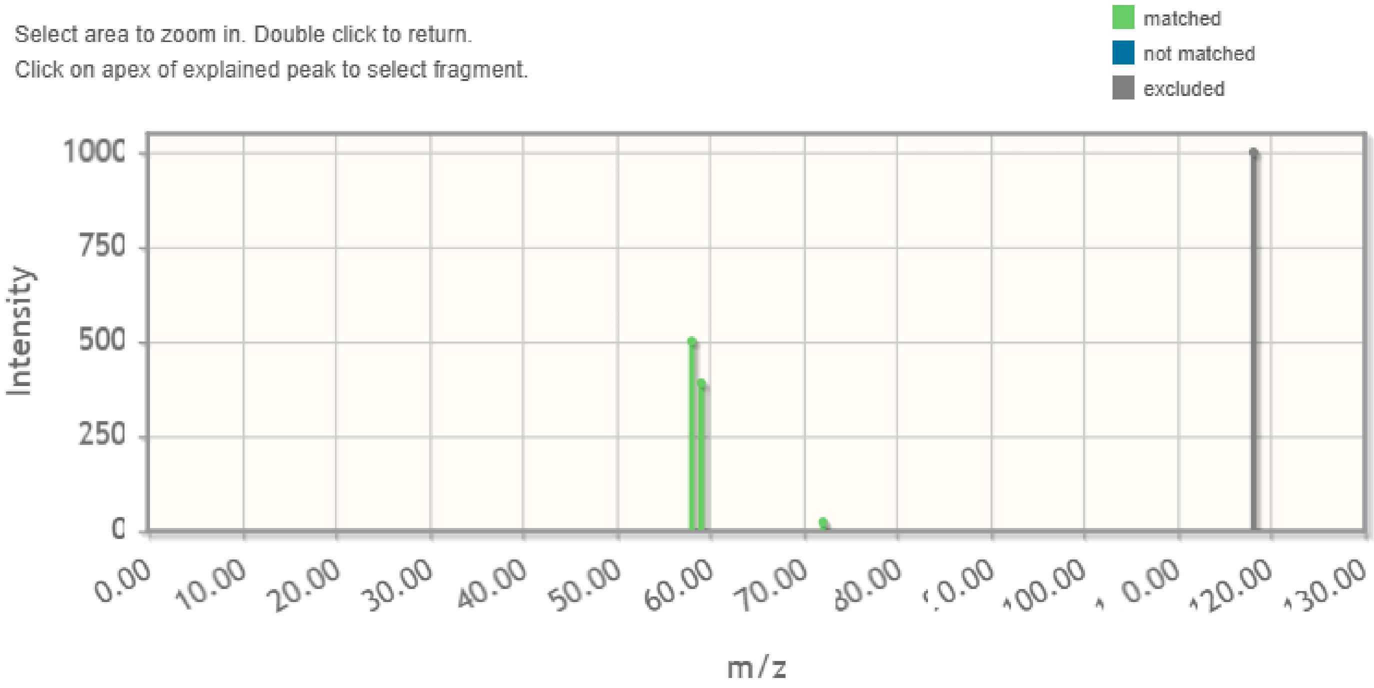

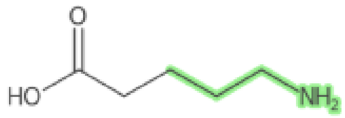

Peak m/z: 59.073265

Fragment Mass: 59.07299 Da

Fragment Formula: $[C_3H_8N]+H^+$

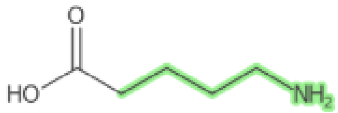

Peak m/z: 72.080902

Fragment Mass: 72.08082 Da

Fragment Formula: $[C_4H_{10}N]^+$

2) 145.10947 – C7H15NO2 MetFrag (2R)-aminoheptanoic acid

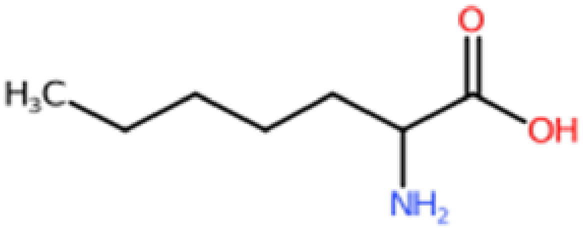

**Identifier**	CHEBI:73738
**SMILES**	CCCCCC(C(=O)O)N
**Monoisot**	145.11
**InChI**	InChI=1S/C7H15NO2/c1-2-3-4-5-6(8)7(9)10/h6H,2-5,8H2,1H3,(H,9,10)/t6-/m1/s1
**MetFrag**	205.171626548776
**Compoun**	(2R)-aminoheptanoic acid
**Molecular**	C7H15NO2
**Identifier**	CHEBI:73738

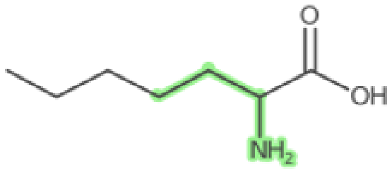

**Fragment 1**	
**Formula**	[C3H7N+2H]+H+
**Mass**	60.08082
**Peak m/z**	60.0815

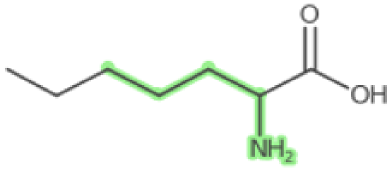

**Fragment 2**	
**Formula**	[C4H9N]+H+
**Mass**	72.08082
**Peak m/z**	72.08144

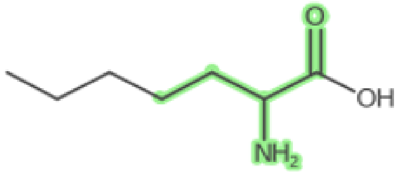

**Fragment 3**	
**Formula**	[C4H7NO-H]+
**Mass**	84.04441
**Peak m/z**	84.04488

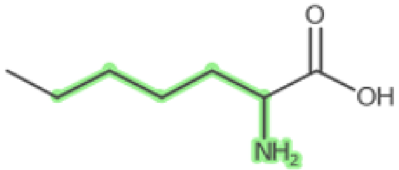

**Fragment 4**	
**Formula**	[C5H11N-H]+
**Mass**	84.08082
**Peak m/z**	84.08121

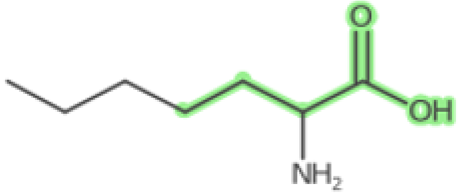

**Fragment 5**	
**Formula**	[C4H6O2]+H+
**Mass**	87.04408
**Peak m/z**	87.04445

https://hmdb.ca/metabolites/HMDB0094649

2) 145.10947 – C7H15NO2 MSFinder (2R)-aminoheptanoic acid

Formula	Error [mDa]	Error [ppm]	Score	Resource	Select
Spectral DB search	0.0000	0.0000	5.0000		☑
C7H15NO2	-0.0448	-0.3087	4.6920	HMDB,ChEBI,DrugBank,Lipid	☑

Name	Score (max=10)	Ontology	InChIKey
UNPD129892	7.92	Acetamides	OTDAUCSBBYTJHP-IFSCUNPJNA-N
2-Aminoheptanoate	7.89	L-alpha-amino acids	RDFMDVXONNIGBC-UHFFFAOYNA-N
N-methylleucine	7.86	Leucine and derivatives	XJODGRWDFZVTKW-UHFFFAOYNA-I
N-methyl-L-isoleucine	7.82	Isoleucine and derivatives	KSPIYJQBLVDRRI-WDSKDSINSA-N
UNPD52667	7.74	Fatty acid methyl esters	ZJEZSISCHHJCIO-UHFFFAOYSA-N
UNPD39350	7.73	Pyrrolidines	PETFZPRPCAYEAH-JHPDDGAFNA-N
ethyl (2S)-2-amino-3-me	7.72	Alpha amino acid esters	BQIVJVAZDJHDJF-LURJTMIESA-N
methyl 2-amino-3-methy	7.72	Isoleucine and derivatives	YXMMTUJDQTVJEN-UHFFFAOYSA-N
(2S)-2-(ethylamino)-3-me	7.71	Valine and derivatives	QHRMEJWWMGUKAM-LURJTMIESA-
3-amino-4-methylhexan	7.67	Beta amino acids and derivatives	JHEDYGILOIBOTL-UHFFFAOYSA-N
UNPD77842	7.67	L-alpha-amino acids	KWSUGULOZFMUDH-MZQZIECVNA-
3-morpholin-4-ylpropan-	7.66	Morpholines	VZKSLWJLGAGPIU-UHFFFAOYSA-N
2-(methylamino)hexanoi	7.66	Alpha amino acids	FPDYKABXINADKS-UHFFFAOYSA-N
4-aminoheptanoic acid	7.65	Gamma amino acids and derivatives	RTSZVAMHOHOBIK-UHFFFAOYSA-N

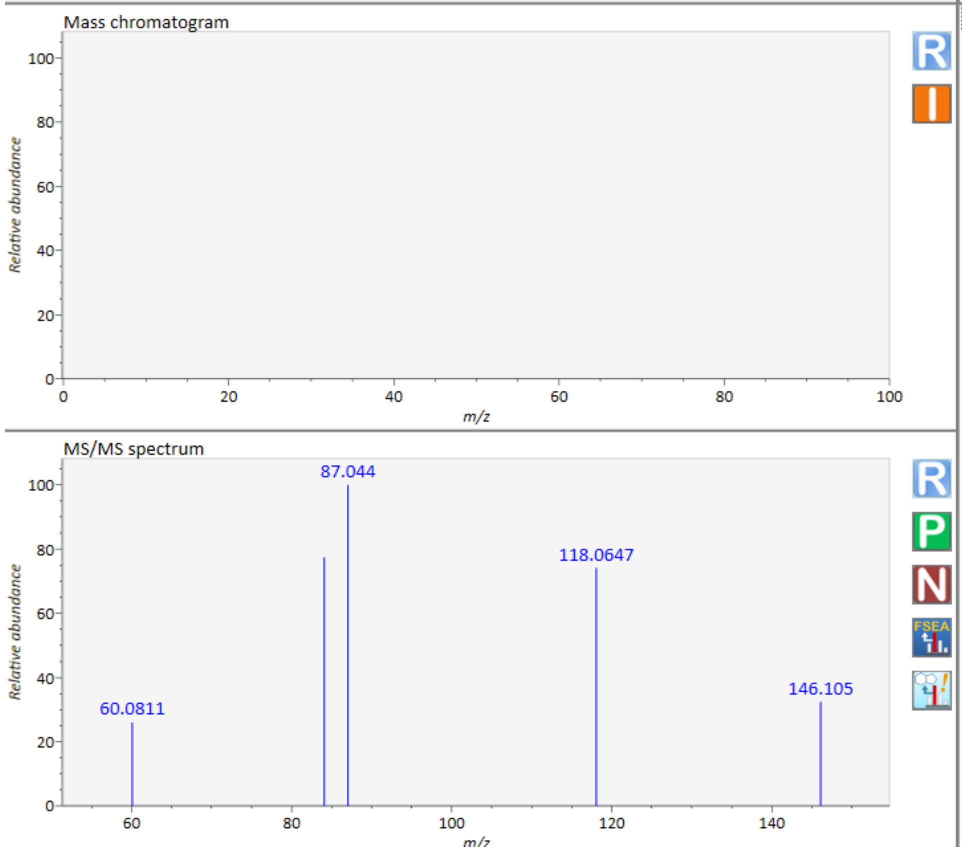

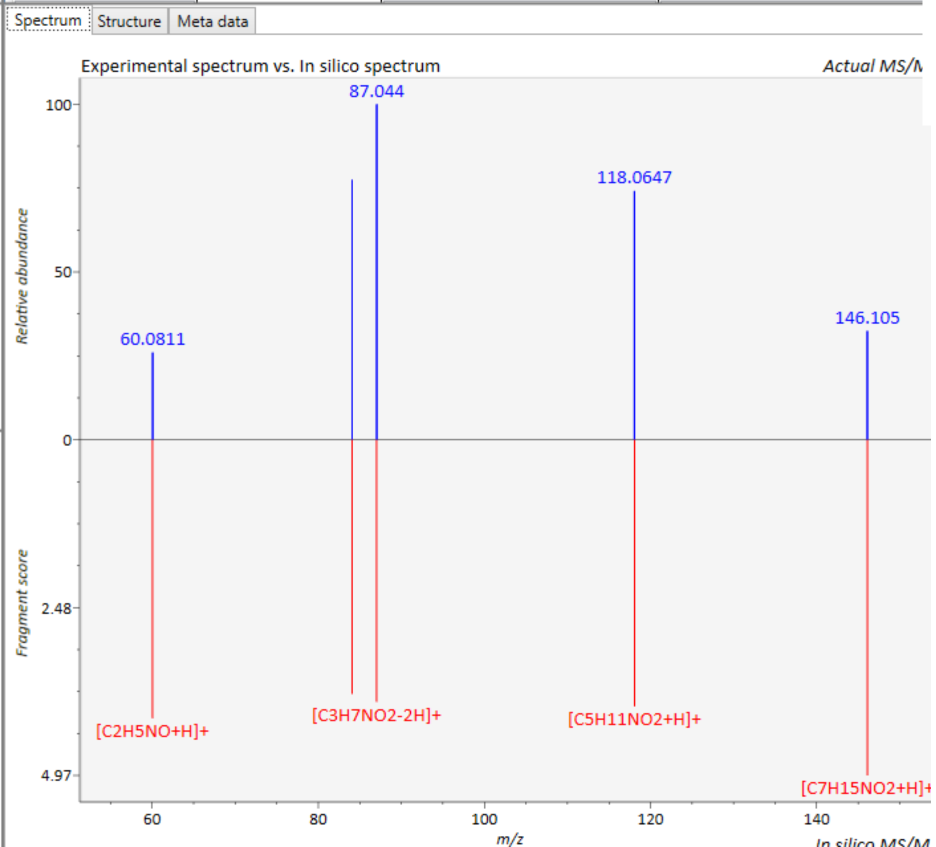

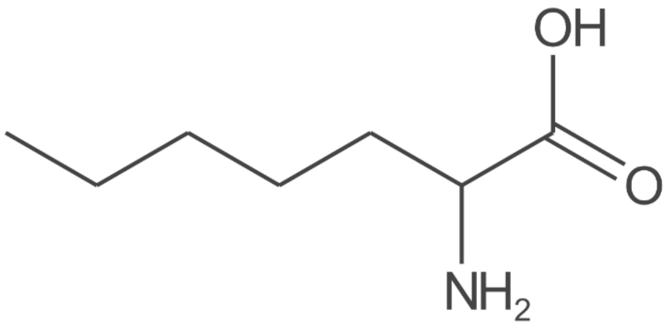

2) 145.10947 – C7H15NO2 Sirius Actinine

Rank	Name	SMILES	Molecular Formula	Adduct	CSI:FingerID Score	Tanimoto Similarity	XLogP	InChIKey	Lipid Class	Database	De Novo
1	4-(trimethylazaniu...	C[N+](C)(C)CCCC...	C7H15NO2	[M + H]+	-76,173	77,381%	0.1	JHPNVNIEXXLNTR		■	

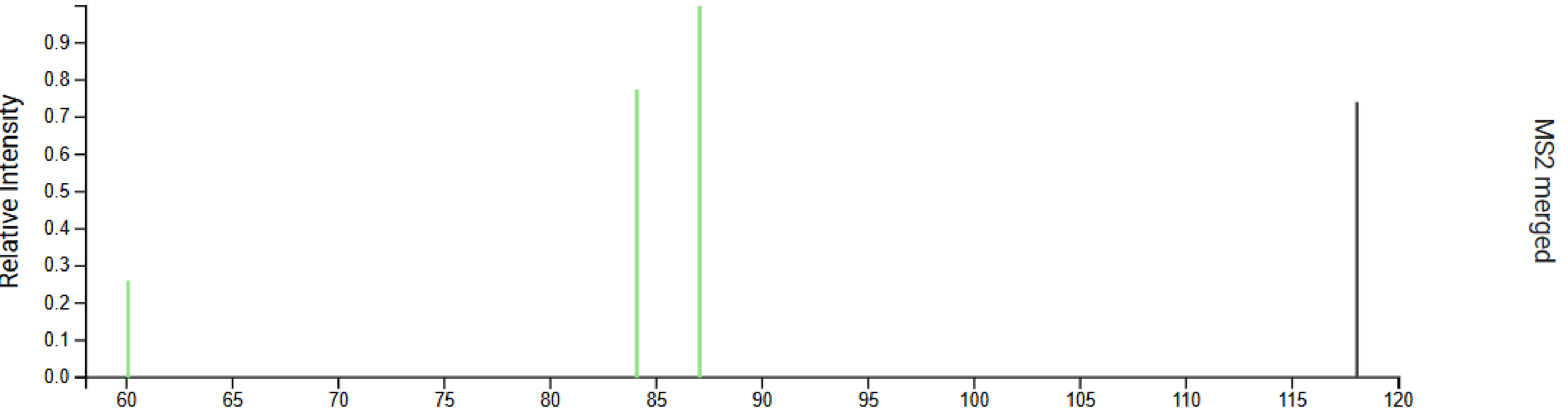

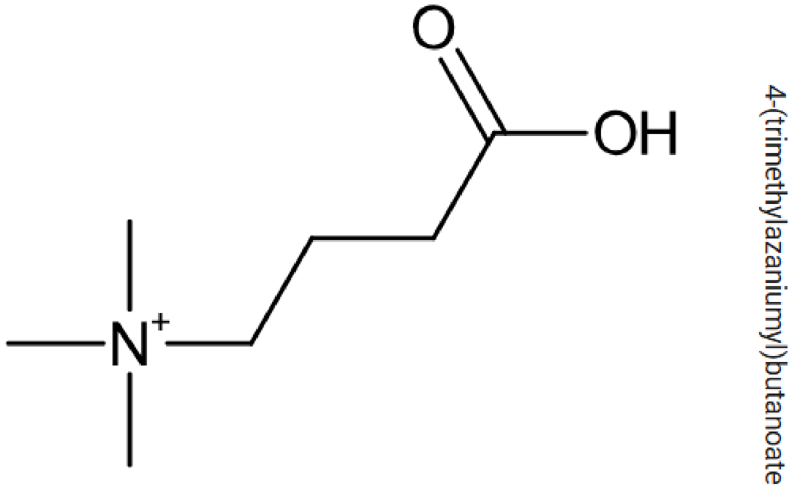

3) 172.07038 – C6H10N3O3 MetFrag 2-Oxoarginine

2	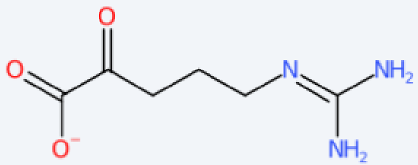 5-guanidino-2-oxopentanoate	CHEBI:18253<br>InChIKeyBlock1 = ARBHXJXXVVHMET	172.072	$C_6H_{10}N_3O_3$	0.7359	Peaks: 1 / 6<br>Fragments<br>Scores<br>Download

Select area to zoom in. Double click to return.
Click on apex of explained peak to select fragment.

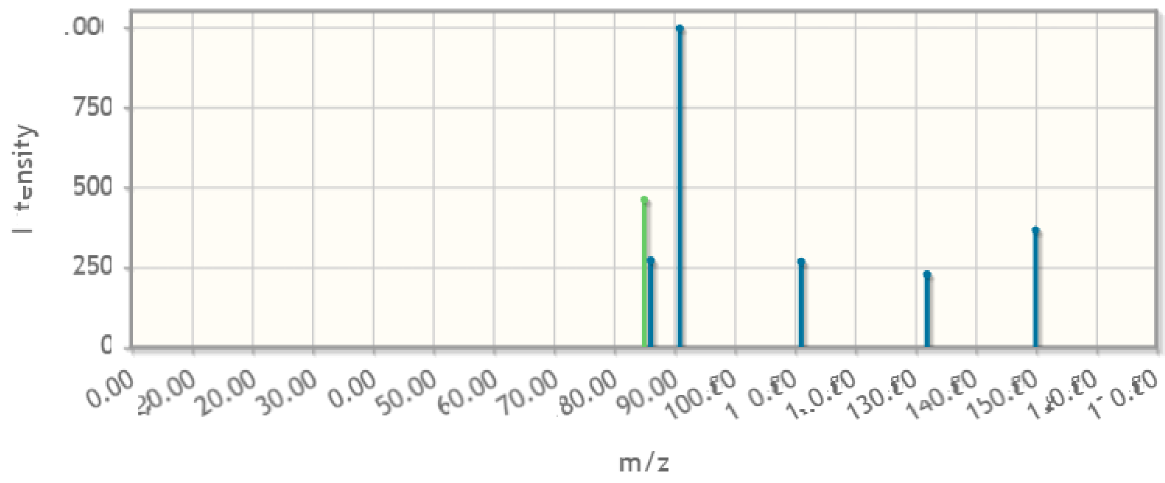

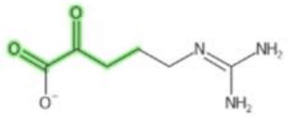

Fragment 1

Peak m/z: 85.0284423828125

Fragment Mass: 85.02842 Da

Fragment Formula: $[C_4H_4O_2]+H^+$

3) 172.07038 – C6H10N3O3 MSFinder Diethyl fumarate

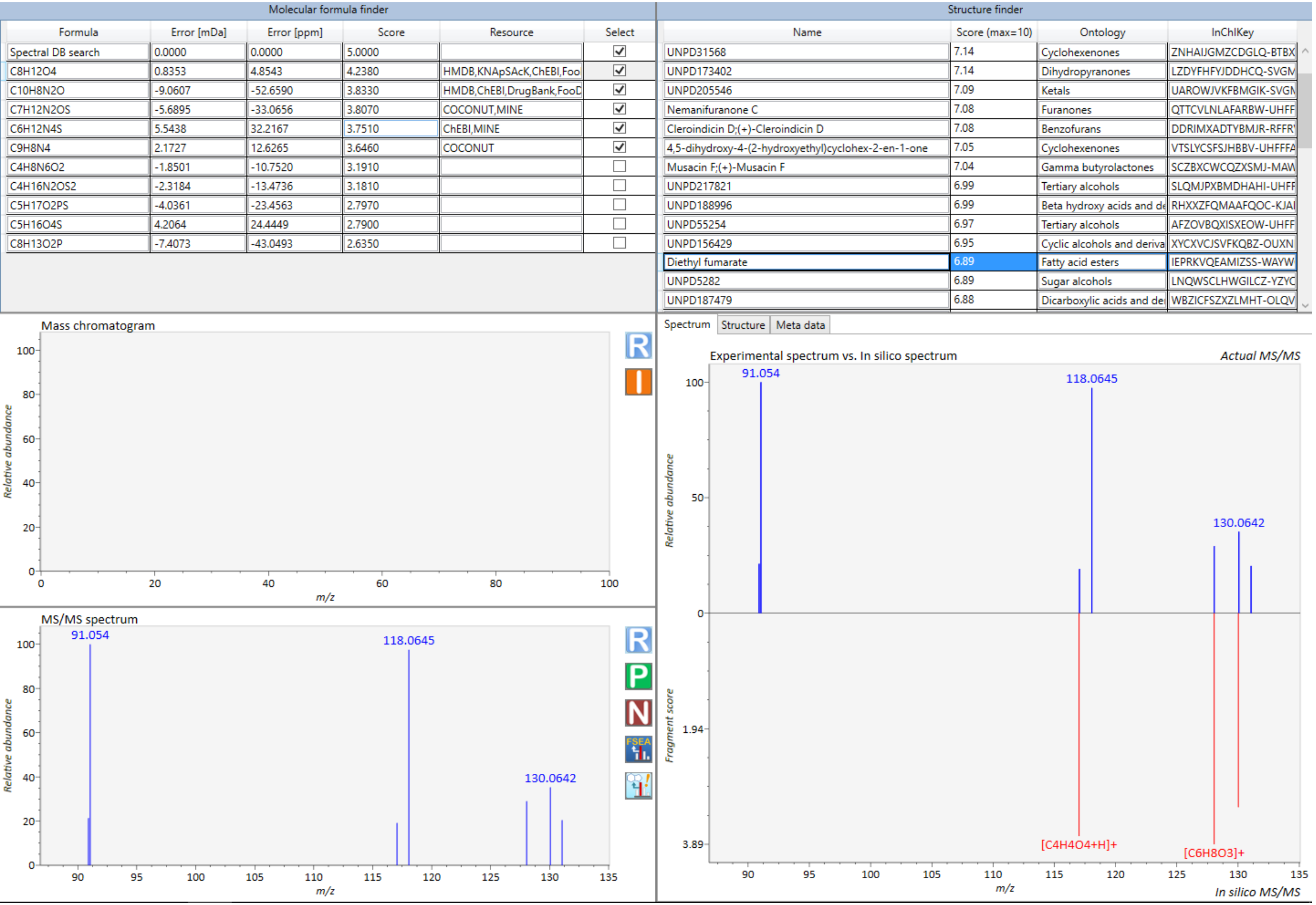

Molecular formula finder

Formula	Error [mDa]	Error [ppm]	Score	Resource	Select
Spectral DB search	0.0000	0.0000	5.0000		☑
C8H12O4	0.8353	4.8543	4.2380	HMDB,KNApSAcK,ChEBI,Foo	☑
C10H8N2O	-9.0607	-52.6590	3.8330	HMDB,ChEBI,DrugBank,FooD	☑
C7H12N2OS	-5.6895	-33.0656	3.8070	COCONUT,MINE	☑
C6H12N4S	5.5438	32.2167	3.7510	ChEBI,MINE	☑
C9H8N4	2.1727	12.6265	3.6460	COCONUT	☑
C4H8N6O2	-1.8501	-10.7520	3.1910		☐
C4H16N2OS2	-2.3184	-13.4736	3.1810		☐
C5H17O2PS	-4.0361	-23.4563	2.7970		☐
C5H16O4S	4.2064	24.4449	2.7900		☐
C8H13O2P	-7.4073	-43.0493	2.6350		☐

Structure finder

Name	Score (max=10)	Ontology	InChIKey
UNPD31568	7.14	Cyclohexenones	ZNHAIJGMZCDGLQ-BTBX
UNPD173402	7.14	Dihydropyranones	LZDYFHFYJDDHCQ-SVGM
UNPD205546	7.09	Ketals	UAROWJVKFBMGIK-SVGN
Nemanifuranone C	7.08	Furanones	QTTCVLNLAFARBW-UHFF
Cleroindicin D;(+)-Cleroindicin D	7.08	Benzofurans	DDRIMXADTYBMJR-RFFR
4,5-dihydroxy-4-(2-hydroxyethyl)cyclohex-2-en-1-one	7.05	Cyclohexenones	VTSLYCSFSJHBBV-UHFFFA
Musacin F;(+)-Musacin F	7.04	Gamma butyrolactones	SCZBXCWCQZXSMJ-MAW
UNPD217821	6.99	Tertiary alcohols	SLQMJPXBMDHAHI-UHFF
UNPD188996	6.99	Beta hydroxy acids and de	RHXXZFQMAAFQOC-KJAI
UNPD55254	6.97	Tertiary alcohols	AFZOVBQXISXEOW-UHFF
UNPD156429	6.95	Cyclic alcohols and deriva	XYCXVCJSVFKQBZ-OUXN
Diethyl fumarate	6.89	Fatty acid esters	IEPRKVQEAMIZSS-WAYW
UNPD5282	6.89	Sugar alcohols	LNQWSCLHWGILCZ-YZYC
UNPD187479	6.88	Dicarboxylic acids and de	WBZICFSZXZLMHT-OLQV

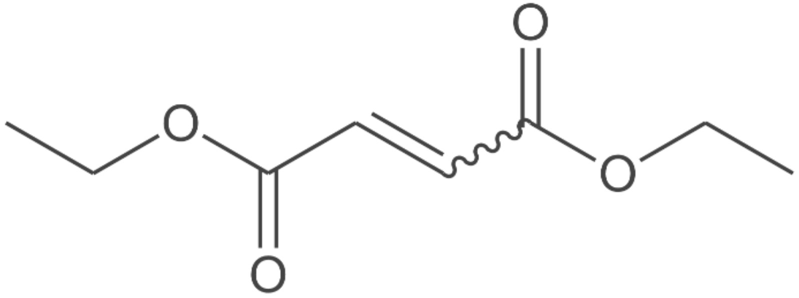

4) 186.07594 – C8H12NO4 MSFinder 2-(2-benzofuranyl)-4,5-dihydro-1H-imidazole

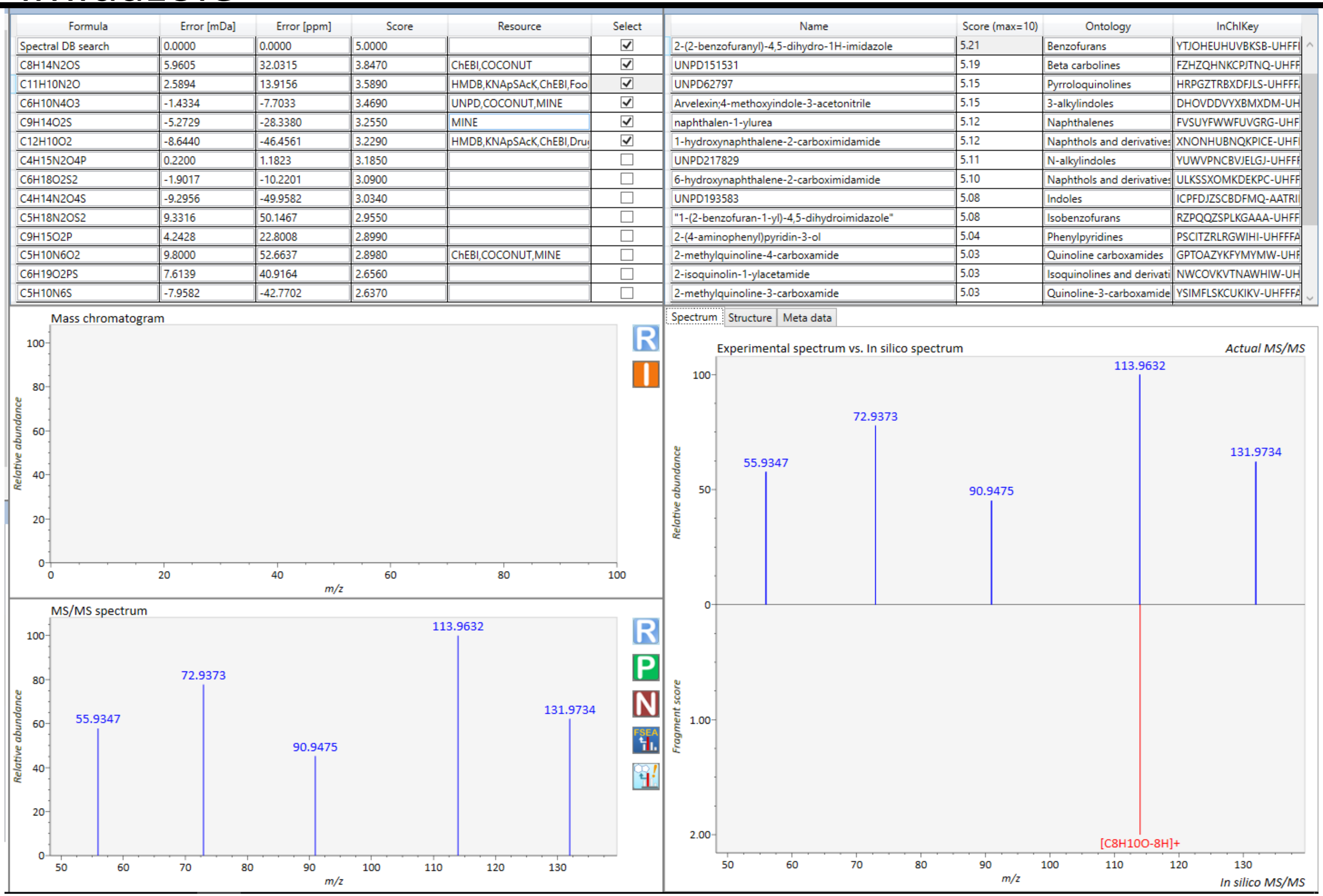

Formula	Error [mDa]	Error [ppm]	Score	Resource	Select
Spectral DB search	0.0000	0.0000	5.0000		☑
C8H14N2OS	5.9605	32.0315	3.8470	ChEBI,COCONUT	☑
C11H10N2O	2.5894	13.9156	3.5890	HMDB,KNApSAcK,ChEBI,Foo	☑
C6H10N4O3	-1.4334	-7.7033	3.4690	UNPD,COCONUT,MINE	☑
C9H14O2S	-5.2729	-28.3380	3.2550	MINE	☑
C12H10O2	-8.6440	-46.4561	3.2290	HMDB,KNApSAcK,ChEBI,Dru	☑
C4H15N2O4P	0.2200	1.1823	3.1850		☐
C6H18O2S2	-1.9017	-10.2201	3.0900		☐
C4H14N2O4S	-9.2956	-49.9582	3.0340		☐
C5H18N2OS2	9.3316	50.1467	2.9550		☐
C9H15O2P	4.2428	22.8008	2.8990		☐
C5H10N6O2	9.8000	52.6637	2.8980	ChEBI,COCONUT,MINE	☐
C6H19O2PS	7.6139	40.9164	2.6560		☐
C5H10N6S	-7.9582	-42.7702	2.6370		☐

Name	Score (max=10)	Ontology	InChIKey
2-(2-benzofuranyl)-4,5-dihydro-1H-imidazole	5.21	Benzofurans	YTJOHEUHUVBKSB-UHFFI
UNPD151531	5.19	Beta carbolines	FZHZQHNKCPJTNQ-UHFF
UNPD62797	5.15	Pyrroloquinolines	HRPGZTRBXDFJLS-UHFFF
Arvelexin;4-methoxyindole-3-acetonitrile	5.15	3-alkylindoles	DHOVDDVYXBMXDM-UH
naphthalen-1-ylurea	5.12	Naphthalenes	FVSUYFWWFUVGRG-UHF
1-hydroxynaphthalene-2-carboximidamide	5.12	Naphthols and derivatives	XNONHUBNQKPICE-UHFI
UNPD217829	5.11	N-alkylindoles	YUWVPNCBVJELGJ-UHFFI
6-hydroxynaphthalene-2-carboximidamide	5.10	Naphthols and derivatives	ULKSSXOMKDEKPC-UHFF
UNPD193583	5.08	Indoles	ICPFDJZSCBDFMQ-AATRII
"1-(2-benzofuran-1-yl)-4,5-dihydroimidazole"	5.08	Isobenzofurans	RZPQQZSPLKGAAA-UHFF
2-(4-aminophenyl)pyridin-3-ol	5.04	Phenylpyridines	PSCITZRLRGWIHI-UHFFFA
2-methylquinoline-4-carboxamide	5.03	Quinoline carboxamides	GPTOAZYKFYMYMW-UHF
2-isoquinolin-1-ylacetamide	5.03	Isoquinolines and derivati	NWCOVKVTNAWHIW-UH
2-methylquinoline-3-carboxamide	5.03	Quinoline-3-carboxamide	YSIMFLSKCUKIKV-UHFFFA

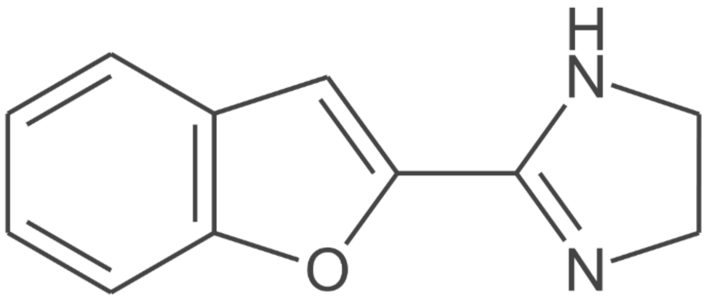

5) 201.17209 – C11H23NO2 MSFinder 11-Aminoundecanoic acid

Molecular formula finder

Formula	Error [mDa]	Error [ppm]	Score	Resource	Select
Spectral DB search	0.0000	0.0000	5.0000		☑
C11H23NO2	0.1554	0.7725	4.5100	ChEBI,STOFF,LipidMAPS,BLEX	☑
C7H19N7	-2.5299	-12.5759	3.5250		☑

Structure finder

Name	Score (max=10)	Ontology	InChIKey
11-Aminoundecanoic acid	7.18	Medium-chain fatty acids	GUOSQNAUYHMCRU-UHI
2-(dimethylamino)-1-(2,2-dimethyloxan-4-yl)ethan-1-ol	7.02	Oxanes	JIWIONXENNGZDF-UHFFF
10-aminoundecanoic acid	6.99	Medium-chain fatty acids	DWHXNKJKNIHNOI-UHFF
4-hydroxy-2,2,6,6-tetramethylpiperidine-1-ethanol	6.64	Piperidines	STEYNUVPFMIUOY-UHFFF
decyl carbamate	6.63	Carbamate esters	ANWCICBGKAHUEY-UHFF
N-(2-hydroxyethyl)-3,5,5-trimethylhexanamide	6.56	N-acylethanolamines	JECPTUXNSPVAST-UHFFFA
n-(7-hydroxy-6-methyl-octyl)-acetamide	6.37	Acetamides	ZJIMVXLDDNBLAK-UHFFF

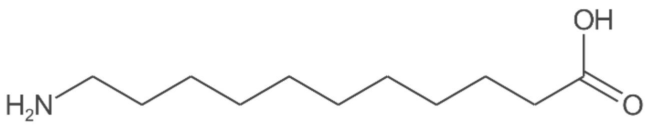

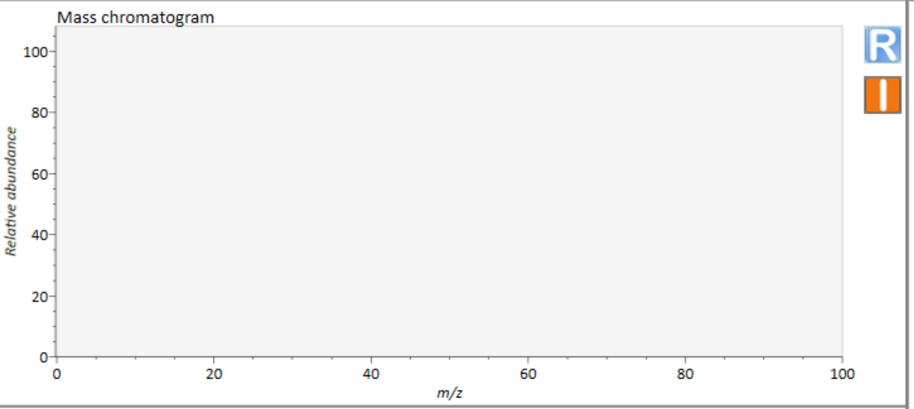

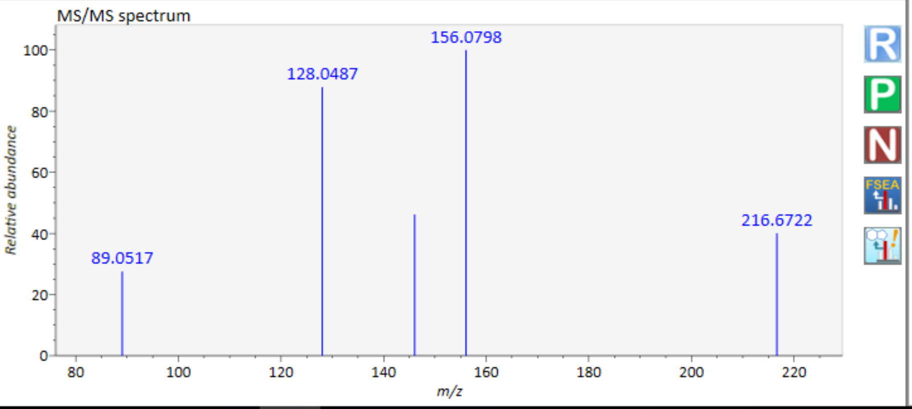

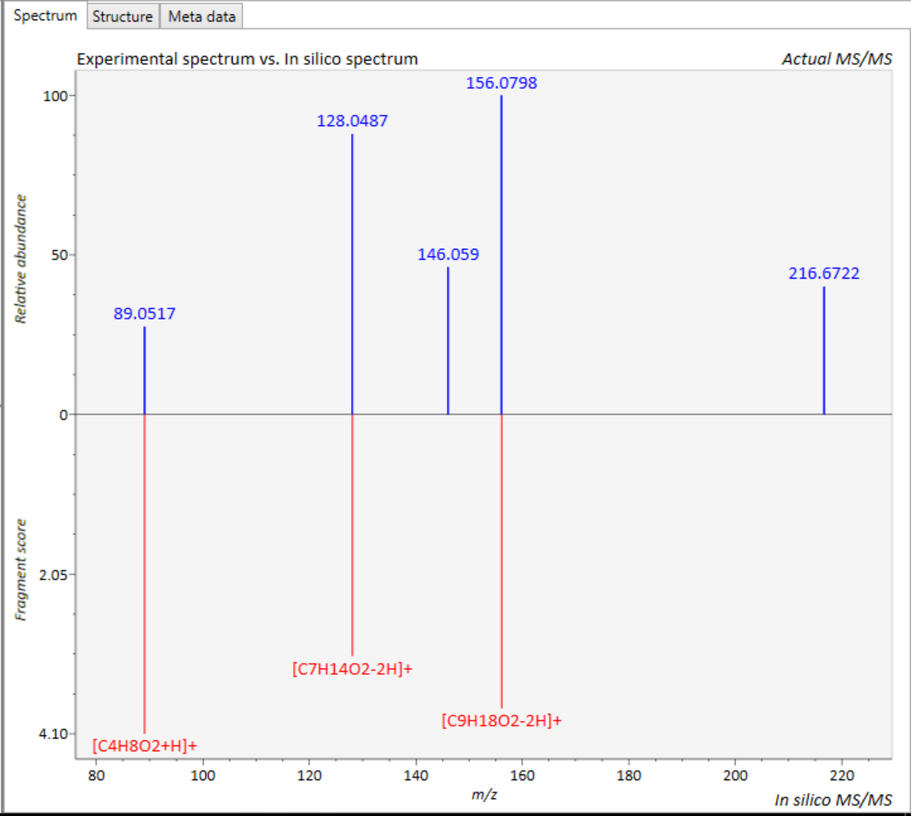

6) 214.11747 – C9H16N3O3 MetFrag 1-(2-morpholin-4-ium-4-ylacetyl)pyrazolidin-3-one

5	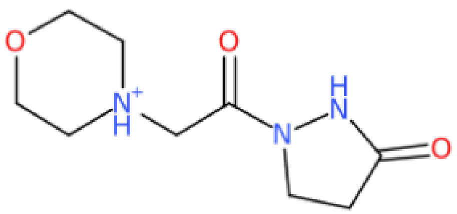	6986976<br>InChIKeyBlock1 = GHMOWHFWDCBAJM	214.119	$C_9H_{16}N_3O_3$	0.6448	Peaks: 3 / 4<br>Fragments<br>Scores<br>Download

Select area to zoom in. Double click to return.
Click on apex of explained peak to select fragment.

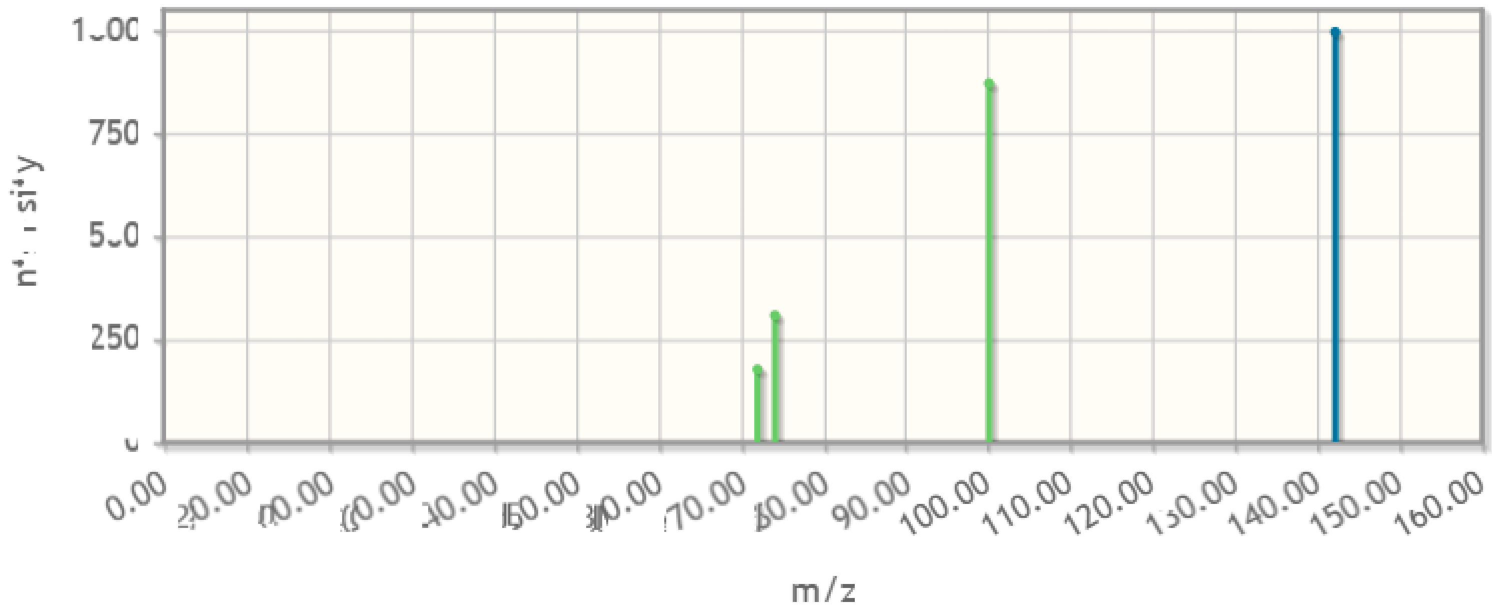

Fragment 1

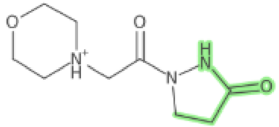

Peak m/z: 72.0451354980469
Fragment Mass: 72.04441 Da
Fragment Formula: $[C_3H_5NO]+H^+$

Fragment 2

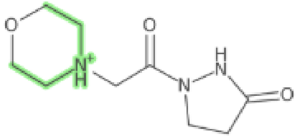

Peak m/z: 74.0971298217773
Fragment Mass: 74.09648 Da
Fragment Formula: $[C_4H_9N+2H]+H^+$

Fragment 3

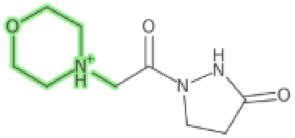

Peak m/z: 100.076271057129
Fragment Mass: 100.07573 Da
Fragment Formula: $[C_5H_{11}NO-H]^+$

6) 214.11747 – C9H16N3O3 MSFinder Alpha-carboxy-delta-decdlactone

Molecular formula finder

Formula	Error [mDa]	Error [ppm]	Score	Resource	Select
Spectral DB search	0.0000	0.0000	5.0000		☑
C9H18N4S	6.4940	30.3280	4.3360	ChEBI,MINE	☑
C7H14N6O2	-0.8999	-4.2028	4.2980	COCONUT	☑
C11H18O4	1.7855	8.3388	4.2910	HMDB,KNApSAcK,FooDB,STC	☑
C10H18N2OS	-4.7394	-22.1349	4.2430	ChEBI,STOFF,COCONUT	☑
C13H14N2O	-8.1105	-37.8800	4.2180	HMDB,KNApSAcK,ChEBI,Dru	☑
C12H14N4	3.1229	14.5847	4.2120	ChEBI,DrugBank,STOFF,BLEXI	☐
C5H19N4O3P	0.7536	3.5195	3.7750		☐
C5H18N4O3S	-8.7621	-40.9234	3.4590		☐
C7H22N2OS2	-1.3682	-6.3900	3.4080		☐
C8H23O2PS	-3.0859	-14.4123	3.3710		☐
C8H22O4S	5.1566	24.0823	3.2930		☐
C11H19O2P	-6.4571	-30.1575	3.2290		☐
C6H22N4S2	9.8652	46.0714	3.0320		☐

Structure finder

Name	Score (max=10)	Ontology	InChIKey
UNPD45331	6.97	Heterocyclic fatty acids	JWXVXVPNKJNCSL-MSFVIGDY
Citeodiol	6.96	Fatty alcohols	VRPFJYGFBAJGLE-HDZCBHPBN
UNPD31871	6.96	Oxanes	XTZATOSHIQEMFD-IMWMWJC
UNPD87977	6.95	Ketals	ZWJRQBZCMYIWBQ-RVUYLQF
alpha-Carboxy-delta-decalactone	6.94	Delta valerolactones	UYZBRJHRPDMUHS-UHFFFAO
CNP0437955	6.93	Lactones	LZMPXUPAQWKNKZ-UHFFFAC
(-)-Pestalotin;LL-P880alpha	6.93	Dihydropyranones	YFIMUDXPJZVJJO-WSYQHHSTI
UNPD87263	6.90	Oxolanes	YDZGIHHZMNNFMM-UOMFB
UNPD101503	6.86	Terpene lactones	RXWSBWAEIRXXDN-HCIYVAPX
2-Carboxy-5,7-dimethyl-4-octanolide	6.85	Gamma butyrolactones	FEFSWWMYJINUMK-UHFFFAO
Tetradenolide	6.85	Fatty alcohols	NNTBVWFIJFDEPF-UHFFFAOY
UNPD183485	6.84	Gamma butyrolactones	VRNXAQRALVPTGJ-SSEDHECM
5-Hexyltetrahydro-2-oxo-3-furancarboxylic a	6.84	Gamma butyrolactones	ZHNHBUOFMLNZNN-UHFFFA
UNPD150367	6.81	Benzofurans	XSZPDSCYNQJNEM-FHGUKDS

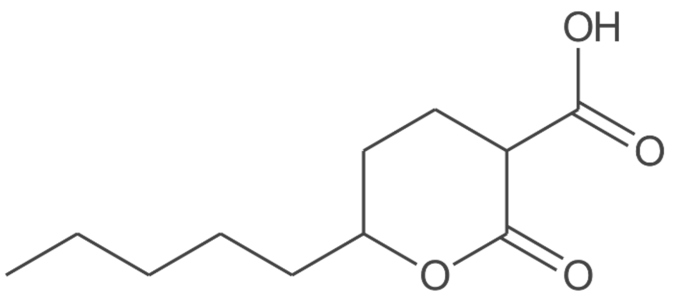

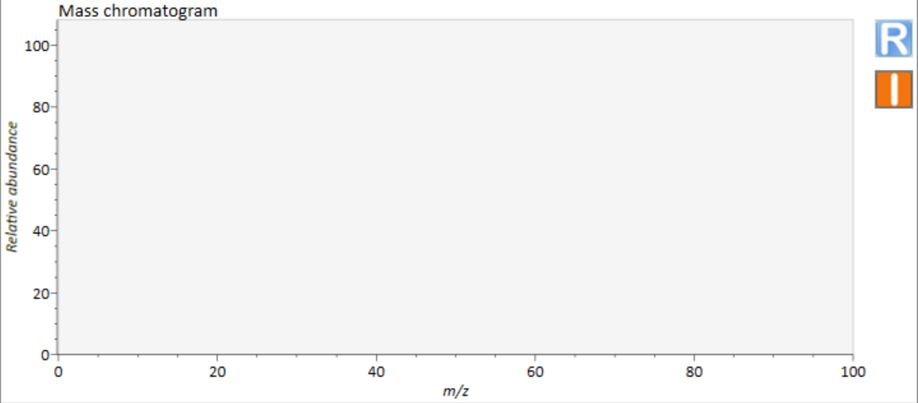

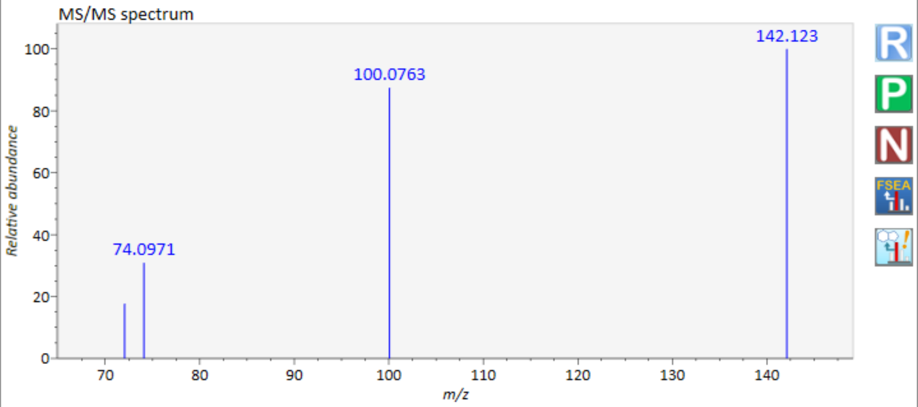

Spectrum Structure Meta data

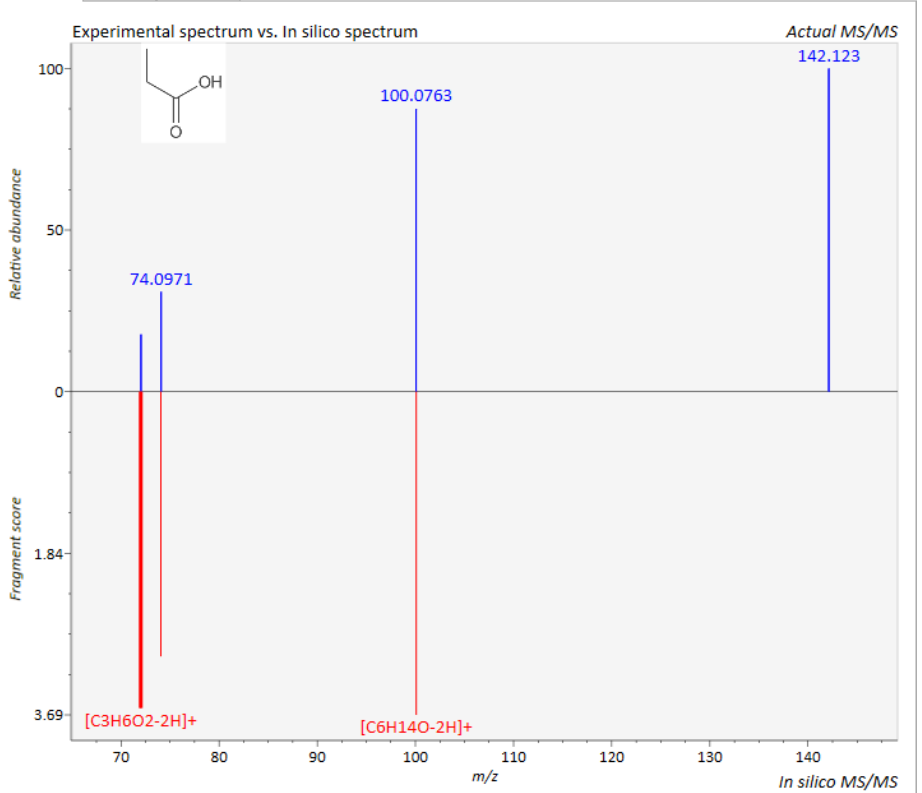

7) 216.09639 – $C10H16O5$ MetFrag 1-Napthol

1	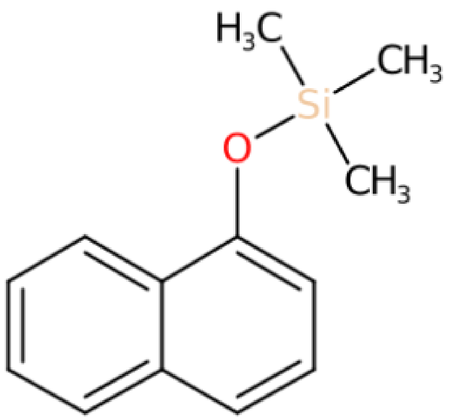	C11714-1<br>InChIKeyBlock1 = YDJRVQVCDFEELI	216.097	$C_{13}H_{16}OSi$	1.0	Peaks: 5 / 11<br>Fragments<br>Scores<br>Download

Select area to zoom in. Double click to return.
Click on apex of explained peak to select fragment.

matched
not matched
excluded

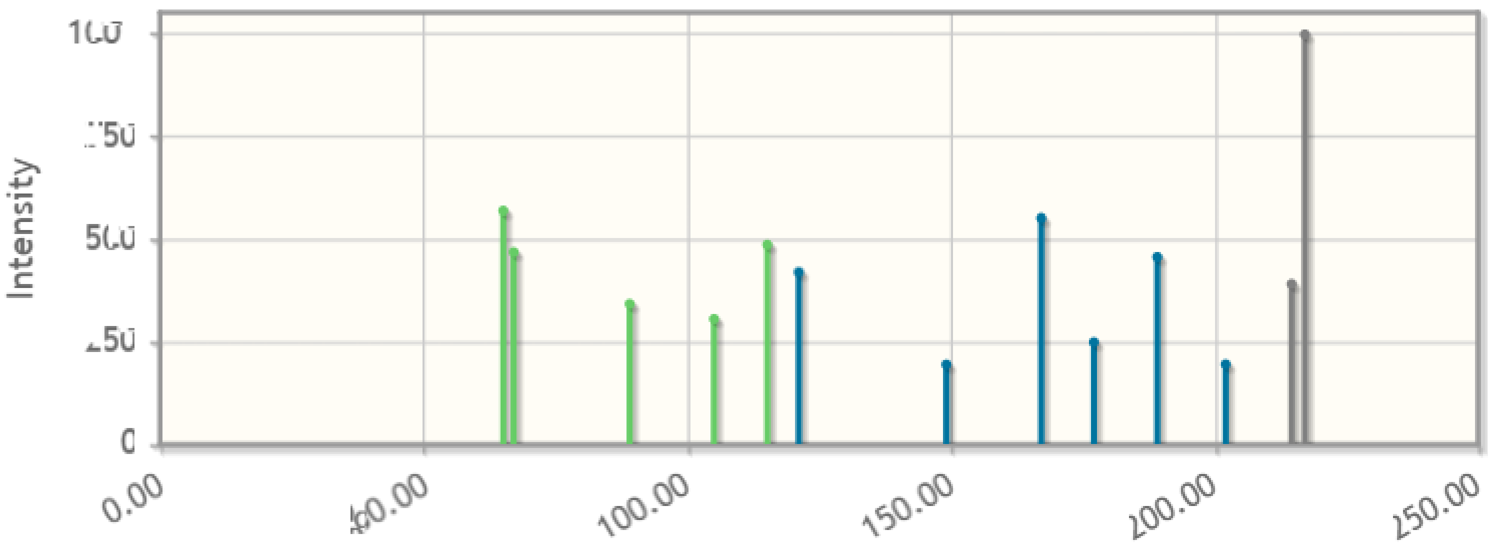

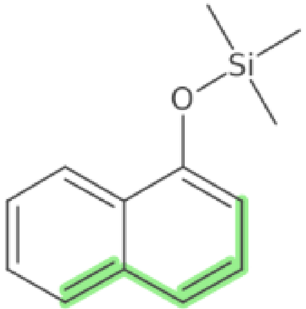

Fragment 1

Peak m/z: 65.0394134521484

Fragment Mass: 65.0386 Da

Fragment Formula: $[C_5H_4]+H^+$

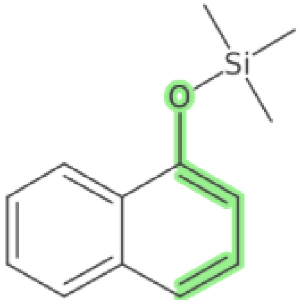

Fragment 2

Peak m/z: 67.0186004638672

Fragment Mass: 67.01785 Da

Fragment Formula: $[C_4H_3O]^+$

7) 216.09639 – $C10H16O5$ MetFrag

Full-MS / t-SIM / PRM

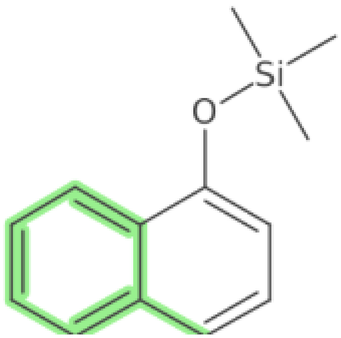

Fragment 3

Peak m/z: 89.0392379760742

Fragment Mass: 89.0386 Da

Fragment Formula: $[C_7H_5]^+$

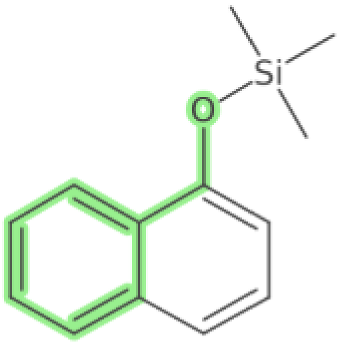

Fragment 4

Peak m/z: 105.034111022949

Fragment Mass: 105.03351 Da

Fragment Formula: $[C_7H_4O]+H^+$

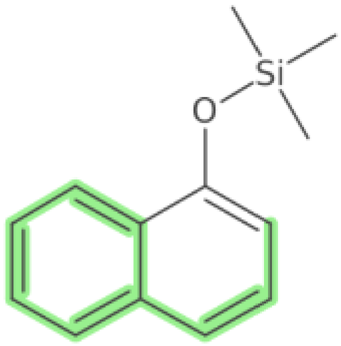

Fragment 5

Peak m/z: 115.054710388184

Fragment Mass: 115.05426 Da

Fragment Formula: $[C_9H_7]^+$

7) 216.09639 – C10H16O5 MetFrag Methoxsalen

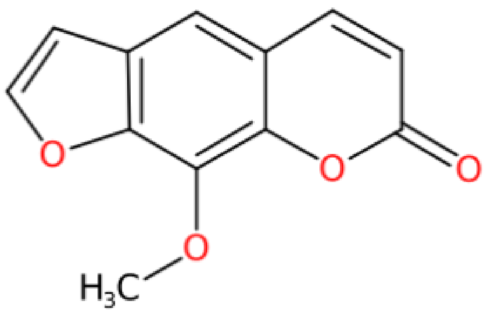

Methoxsalen

Methoxsalen	HMDB14693<br>InChIKeyBlock1 = QXKHYNVANLEOEG	216.042	$C_{12}H_8O_4$	1.0	Peaks: 10 / 11<br>Fragments<br>Scores<br>Download

Select area to zoom in. Double click to return.
Click on apex of explained peak to select fragment.

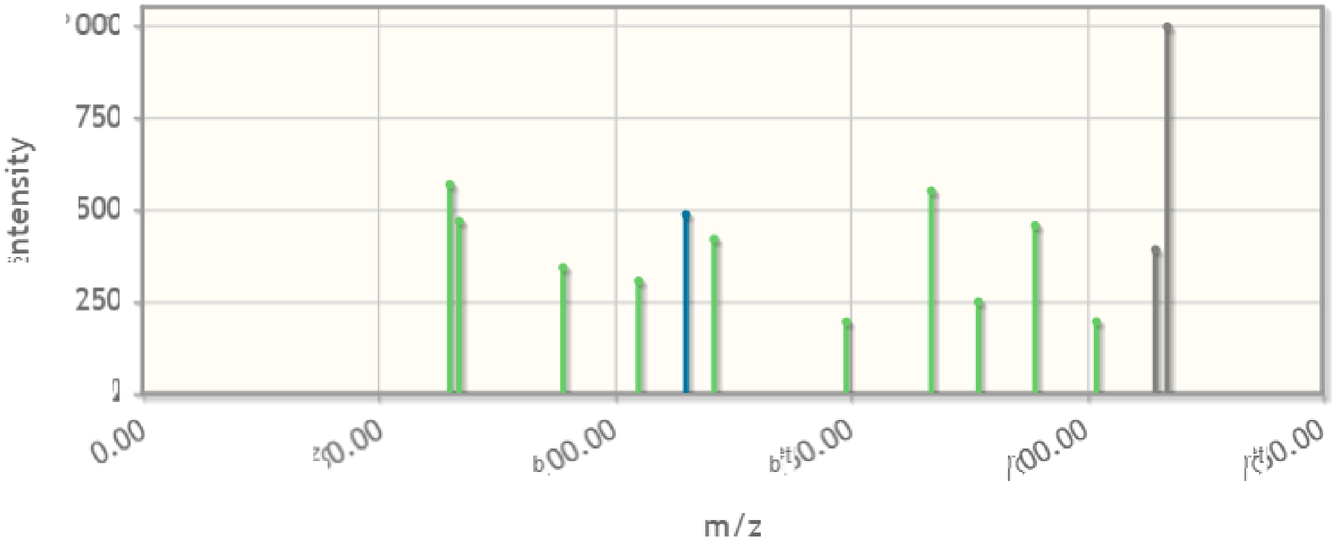

Fragment 1

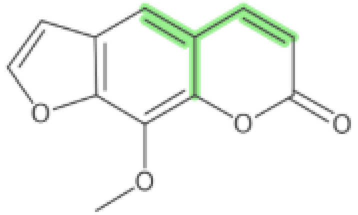

Peak m/z: 65.0394134521484

Fragment Mass: 65.0386 Da

Fragment Formula: $[C_5H_3+H]+H^+$

Fragment 2

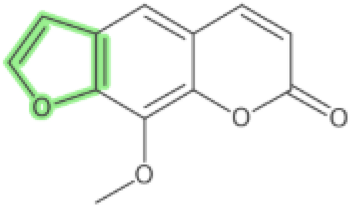

Peak m/z: 67.0186004638672

Fragment Mass: 67.01785 Da

Fragment Formula: $[C_4H_2O]+H^+$

7) 216.09639 – C10H16O5 MetFrag

Full-MS / t-SIM / PRM

Fragment 3

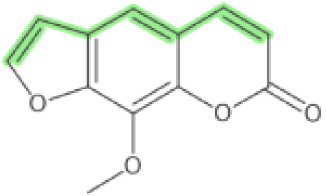

Peak m/z: 89.0392379760742

Fragment Mass: 89.0386 Da

Fragment Formula: $[C_7H_5]^+$

Fragment 4

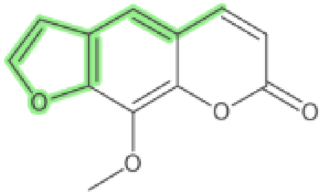

Peak m/z: 105.034111022949

Fragment Mass: 105.03351 Da

Fragment Formula: $[C_7H_4O]{+}H^+$

Fragment 5

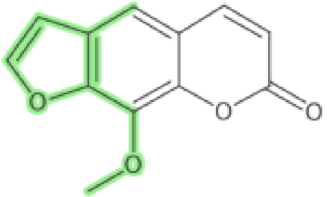

Peak m/z: 121.028945922852

Fragment Mass: 121.02842 Da

Fragment Formula: $[C_7H_6O_2{-}H]^+$

Fragment 6

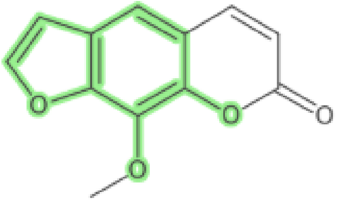

Peak m/z: 149.023590087891

Fragment Mass: 149.02333 Da

Fragment Formula: $[C_8H_3O_3{+}H]{+}H^+$

Fragment 7

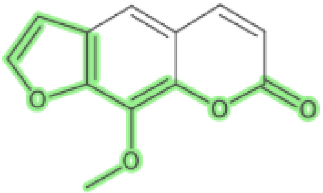

Peak m/z: 167.034332275391

Fragment Mass: 167.0339 Da

Fragment Formula: $[C_8H_5O_4{+}H]{+}H^+$

Fragment 8

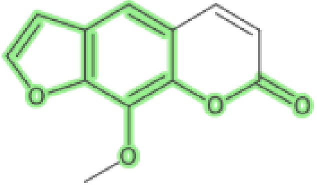

Peak m/z: 177.018585205078

Fragment Mass: 177.01824 Da

Fragment Formula: $[C_9H_3O_4{+}H]{+}H^+$

Fragment 9

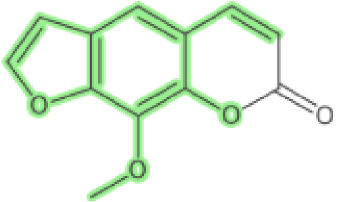

Peak m/z: 189.055099487305

Fragment Mass: 189.05465 Da

Fragment Formula: $[C_{11}H_8O_3]{+}H^+$

Fragment 10

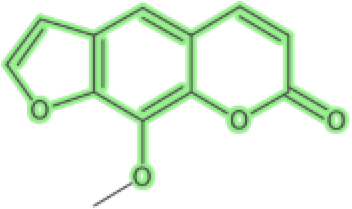

Peak m/z: 202.026382446289

Fragment Mass: 202.02607 Da

Fragment Formula: $[C_{11}H_5O_4]{+}H^+$

7) 216.09639 – C10H16O5 MSFinder Aspyronol

Molecular formula finder

Formula	Error [mDa]	Error [ppm]	Score	Resource	Select
Spectral DB search	0.0000	0.0000	5.0000		☑
C10H16O5	3.0501	14.1143	4.0480	KNApSAcK,ChEBI,DrugBank,	☑
C12H12N2O2	-6.8459	-31.6808	3.8800	HMDB,KNApSAcK,ChEBI,Foc	☑
C9H16N2O2S	-3.4748	-16.0801	3.8380	BLEXP,COCONUT,MINE	☑
C11H12N4O	4.3875	20.3030	3.7580	ChEBI,BLEXP,COCONUT,MIN	☑
C6H12N6O3	0.3647	1.6877	3.5780	BLEXP	☑
C17H12	-2.8232	-13.0647	3.5190	ChEBI,STOFF,UNPD,BLEXP,C(	☐
C7H21O3PS	-1.8214	-8.4287	3.0820		☐
C10H17O3P	-5.1925	-24.0292	3.0770		☐
C6H20N2O2S2	-0.1037	-0.4799	3.0690		☐
C8H16N4OS	7.7586	35.9021	3.0090	MINE	☐
C7H20O5S	6.4212	29.7136	2.9400		☐
C9H17N2O2P	6.0409	27.9538	2.9310		☐
C14H16S	0.5480	2.5359	2.8960		☐

Structure finder

Name	Score (max=10)	Ontology	InChIKey
Aspyronol	6.78	Dihydropyranones	HPKIOTFRXLRBAB-MTSNS
Aspilactonol D	6.77	Butenolides	GIDACBQCHAAPHA-HIOF
Decarestrictin N; Decarestrictine N	6.75	Oxocins	HWMMWMJBUOCCFZ-M
Xylogiblactone C	6.73	Butenolides	FUNQMDCXBHDBED-FNC
UNPD223104	6.72	Dihydropyranones	PYNPHESJRWRPGG-POYE
Ophiocerin D;(+)-Ophiocerin D	6.72	Fatty acid esters	ZKDAKCXDTRJOGZ-MWJH
UNPD221083	6.66	Ketals	TZUFEMKJENBYIQ-WHQC
UNPD140473	6.65	Oxocins	KYLGPSQYGNCTQJ-PGAQ
Longiflorone;(+)-Longiflorone	6.64	Terpene lactones	CCTSZLOYMHLMHP-UHFI
UNPD130307	6.60	Terpene lactones	KPDCJYWUNAHYDR-TXCI
UNPD172611	6.60	1,4-dioxepanes	ACOYLHVUGSEGKA-MAID
5,6,9-trihydroxy-10-methyl-3,4,5,6,9,10-hexahydro-2H-oxe	6.60	Oxocins	IDESAYMOFUISQT-UHFFF
5-(hydroxymethyl)-6-(1-hydroxypropyl)-4-methoxy-2H-py	6.56	Pyrans	OEFHOBYLAXIGLN-UHFFF
CNP0429714	6.56	Cyclitols and derivatives	CSBVPLTZWBYCDB-UHFFI

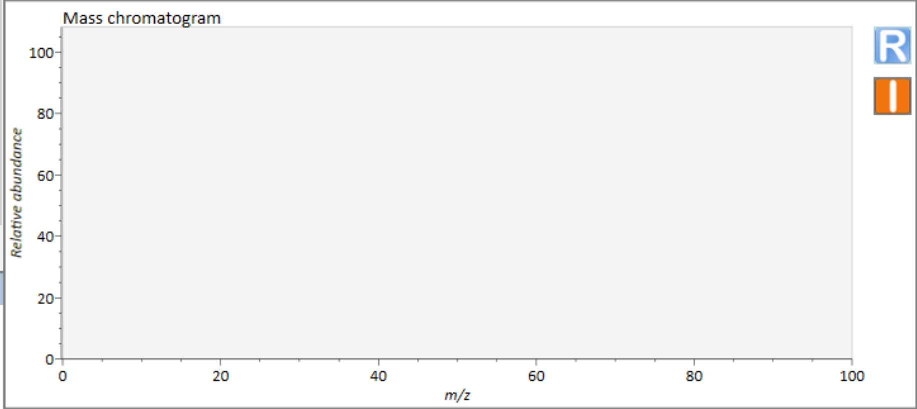

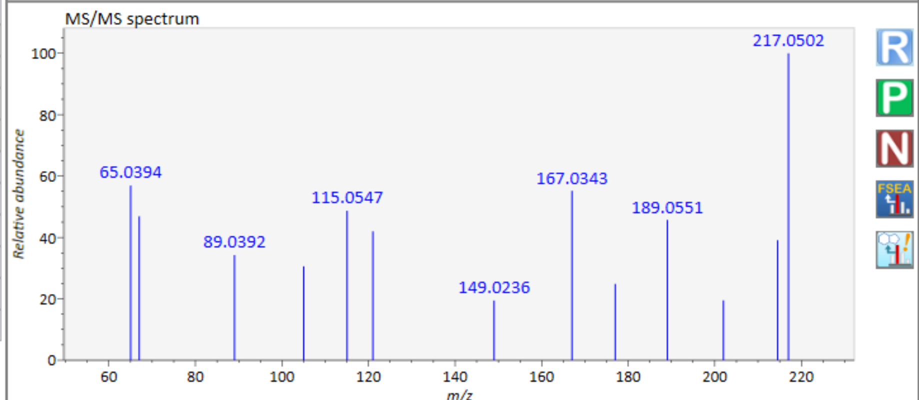

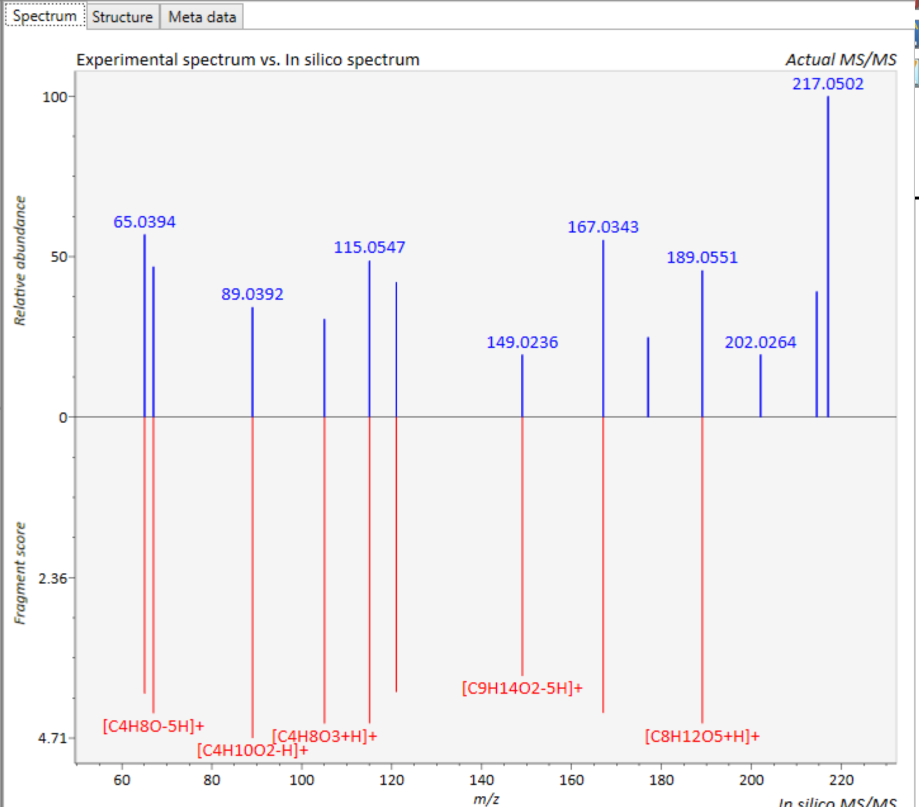

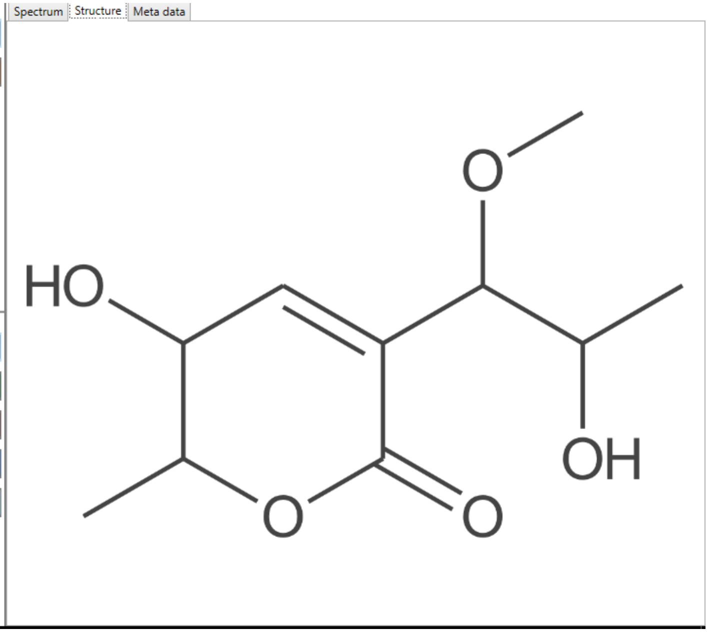

8) 269.30702 – C18H39N MassFrontier Octadecane-1-amine

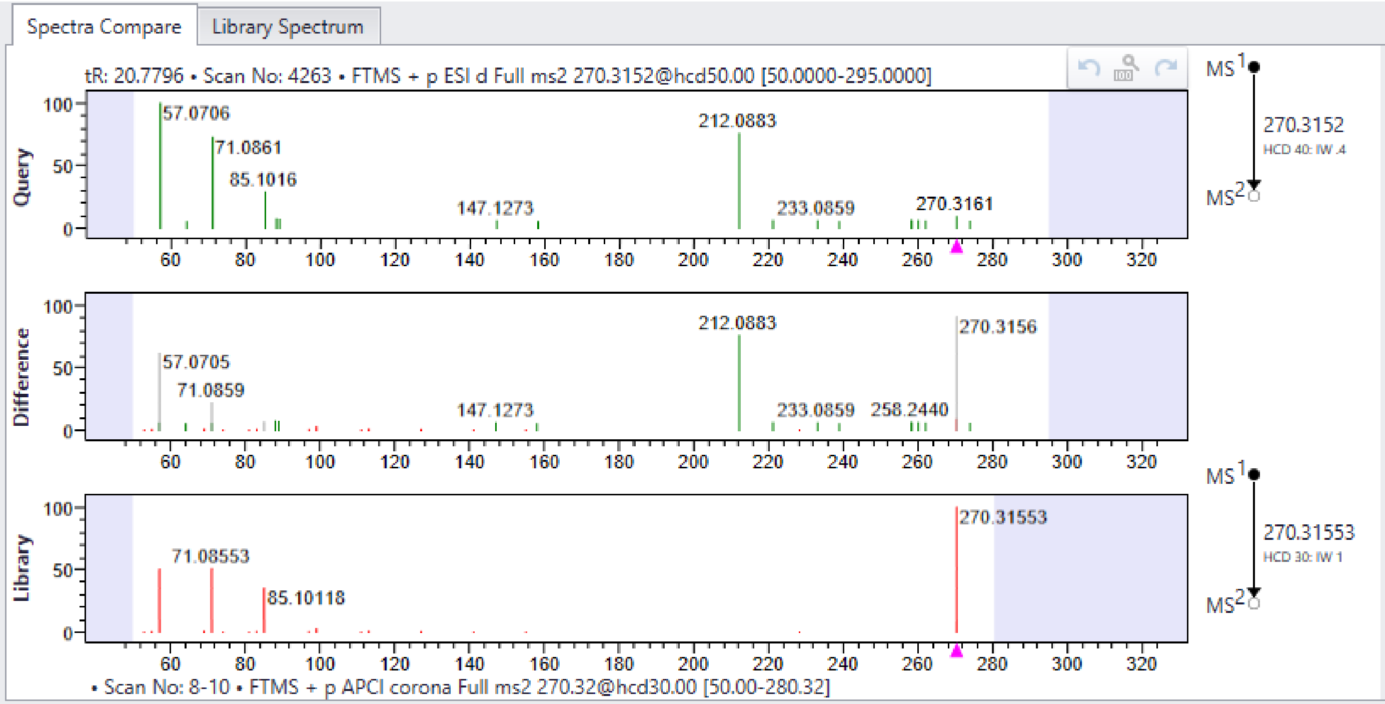

Match	HighChem High Res	43.9
	Opt.Dot Product	4.4
	NIST (Modified)	19.6
Confidence	Identity	**23.5**
	Similarity Forward	19.1
	Similarity Reverse	6.0

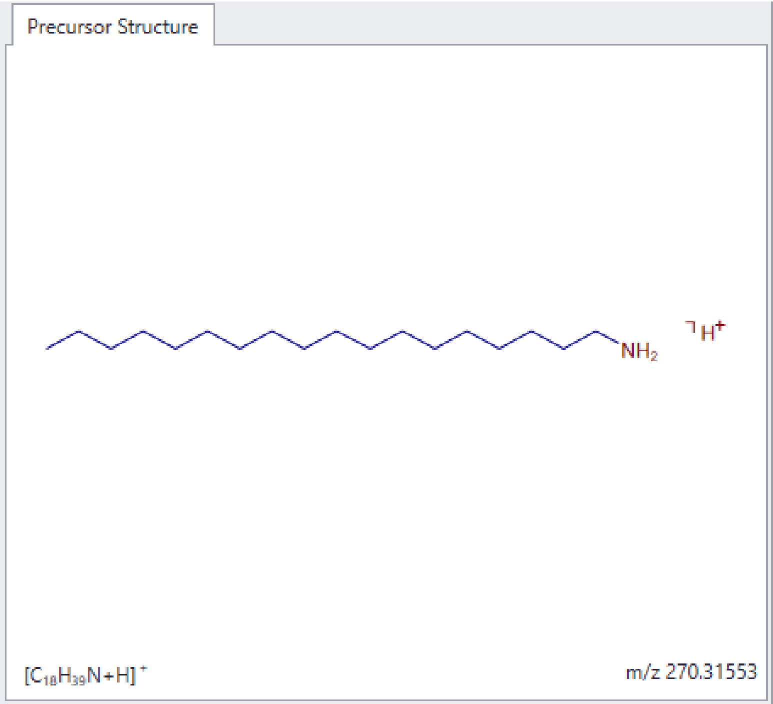

8) 269.30702 – C18H39N MetFrag Octadecane-1-amine

**Identifier**	CHEBI:63866
**MetFrag**	52.571658305074855
**InChI**	InChI=1S/C18H39N/c1-2-3-4-5-6-7-8-9-10-11-12-13-14-15-16-17-18-19/h2-19H2,1H3
**LossStat**	-129.55160672744813
**Monoisot**	269.308
**Compoun**	octadecan-1-amine
**Identifier**	CHEBI:63866
**Molecular**	C18H39N
**SMILES**	CCCCCCCCCCCCCCCCCCN
**SpectralS**	2.567755750106315
**PeakStat**	-40.79396373764532
**ExactSpe**	0.52636

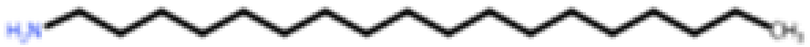

Fragment 1

**Formula**	[C4H9]+
**Mass**	57.06992
**Peak m/z**	57.070556640625

Fragment 2

**Formula**	[C4H9]+
**Mass**	57.06992
**Peak m/z**	57.0713043212891

Fragment 3

**Formula**	[C5H11]+
**Mass**	71.08558
**Peak m/z**	71.0860977172852

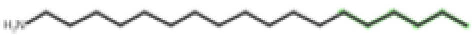

Fragment 4

**Formula**	[C6H13]+
**Mass**	85.10124
**Peak m/z**	85.101646423398

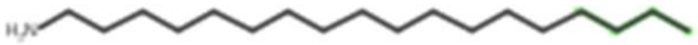

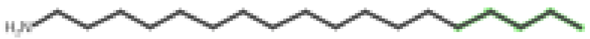

8) 269.30702 – C18H39N MSFinder Octadecane-1-amine

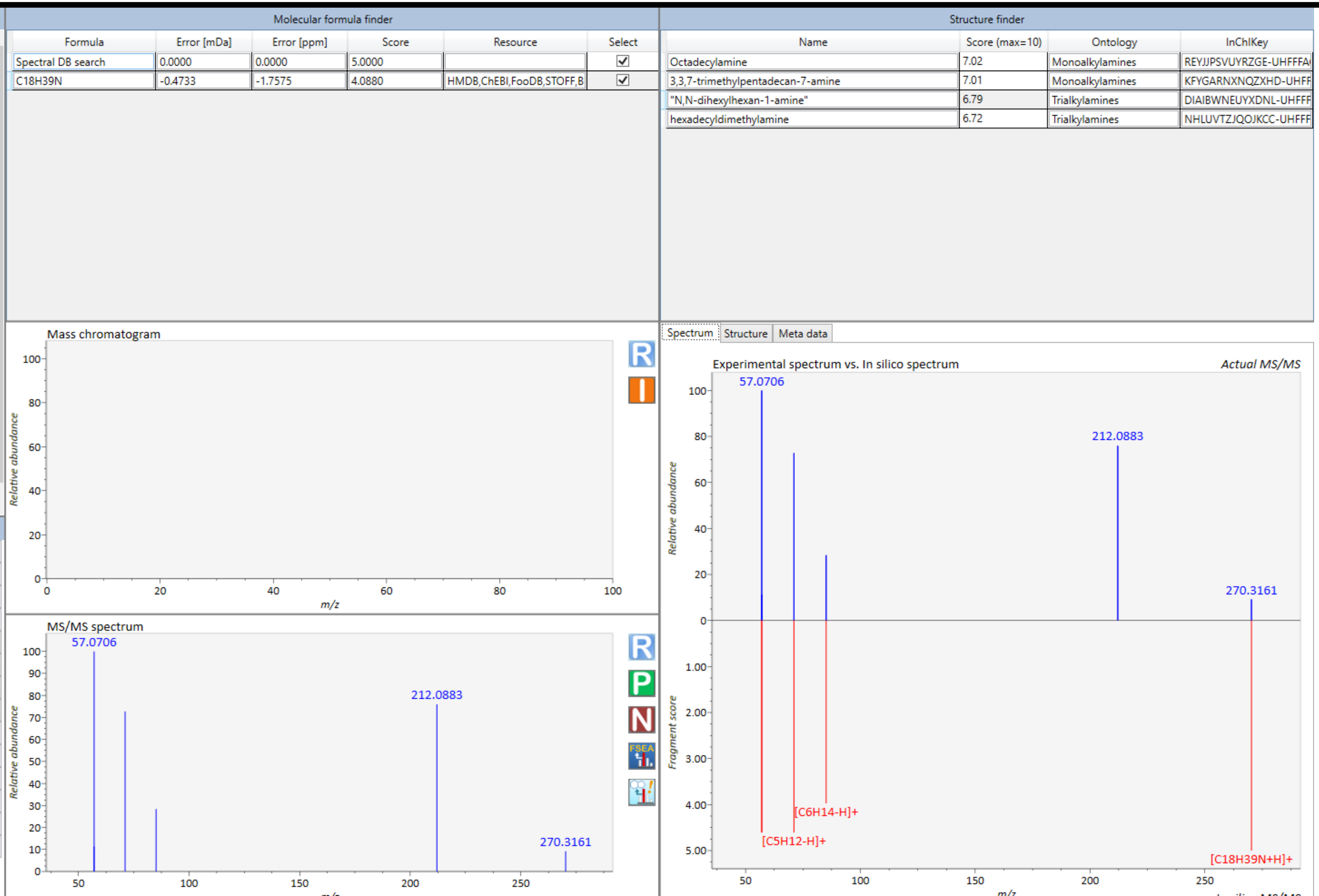

Molecular formula finder

Formula	Error [mDa]	Error [ppm]	Score	Resource	Select
Spectral DB search	0.0000	0.0000	5.0000		☑
C18H39N	-0.4733	-1.7575	4.0880	HMDB,ChEBI,FooDB,STOFF,B	☑

Structure finder

Name	Score (max=10)	Ontology	InChIKey
Octadecylamine	7.02	Monoalkylamines	REYJJPSVUYRZGE-UHFFFA
3,3,7-trimethylpentadecan-7-amine	7.01	Monoalkylamines	KFYGARNXNQZXHD-UHFF
"N,N-dihexylhexan-1-amine"	6.79	Trialkylamines	DIAIBWNEUYXDNL-UHFF
hexadecyldimethylamine	6.72	Trialkylamines	NHLUVTZJQOJKCC-UHFF

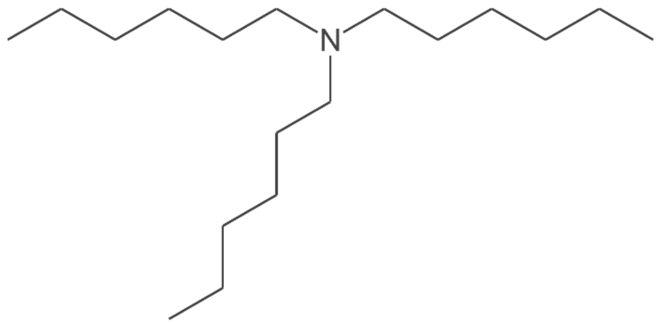

8) 269.30702 – C18H39N Sirius Octadecane-1-amine

Rank	Name	SMILES	Molecular Formula	Adduct	CSI:FingerID Score	Tanimoto Similarity	XLogP	InChIKey	Lipid Class	Database	De Novo
1	octadecan-1-amine	CCCCCCCCCCCC...	C18H39N	[M + H]+	-36,308	85,897%	6.8259177	REYJJPSVUYRZGE		■	■

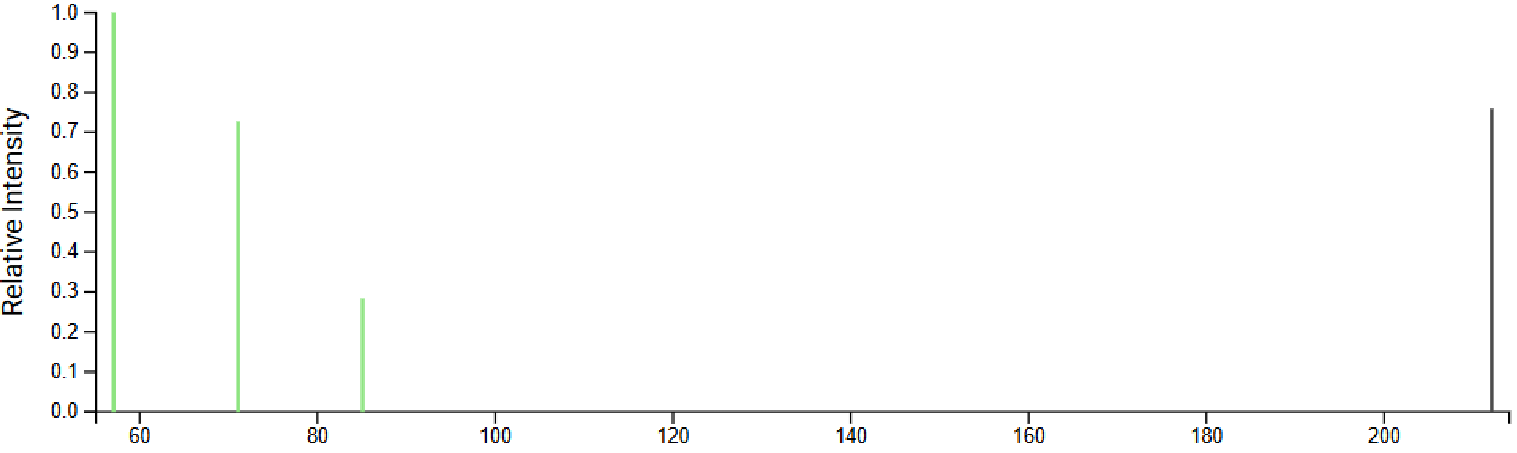

MS2 merged

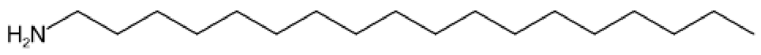

octadecan-1-amine

9) 272.15857 – C10H20N6O3 MetFrag (R*,S*)-4-[1-Ethyl-2-(4-fluorop+henyl)butyl]phenol

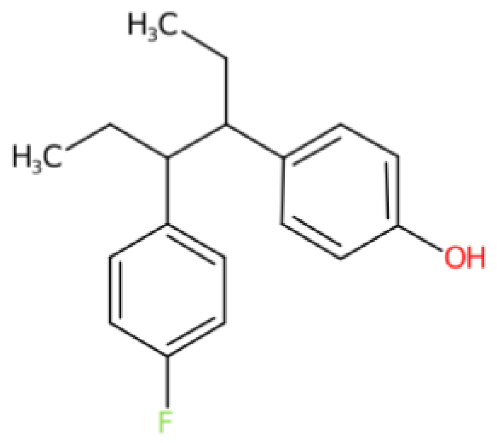 (R*,S*)-4-[1-Ethyl-2-(4-fluorophenyl)butyl]phenol	CHEBI:79960<br>InChIKeyBlock1 = IPXYROUXWHVCDA	272.158	$C_{18}H_{21}FO$	1.0	Peaks: 5 / 13<br>Fragments<br>Scores<br>Download

Select area to zoom in. Double click to return.
Click on apex of explained peak to select fragment.

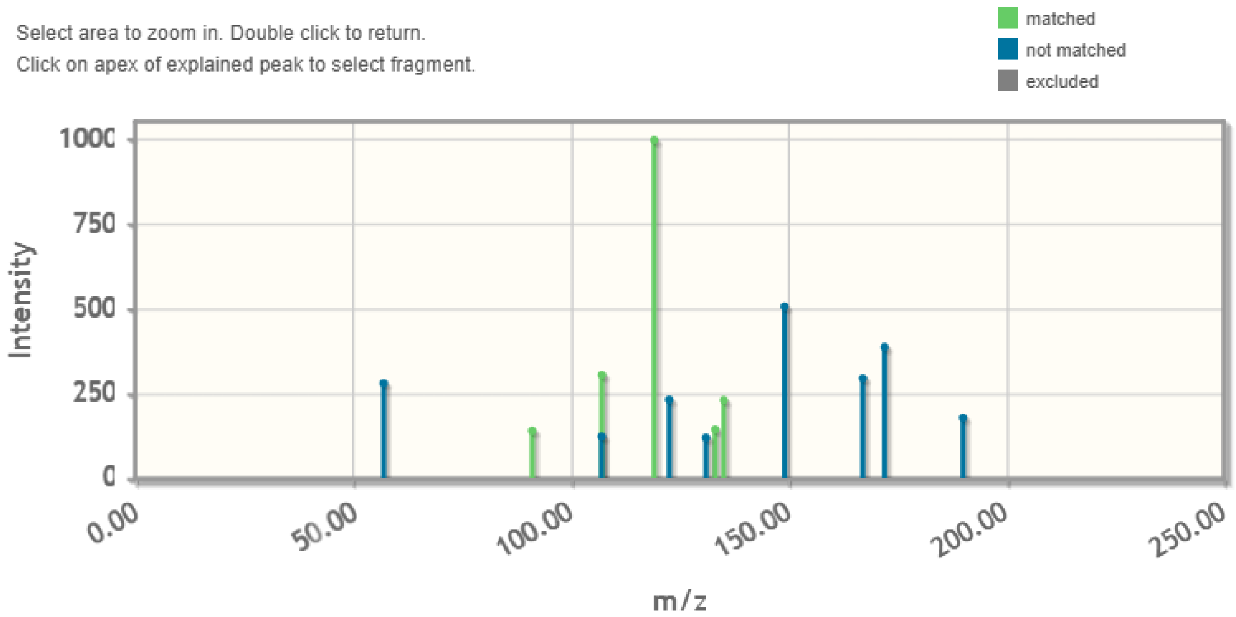

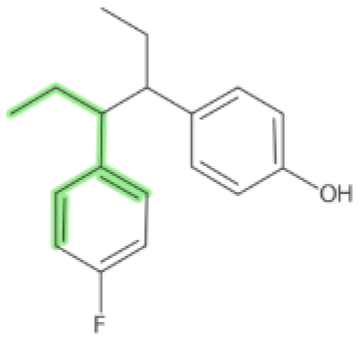

Fragment 1

Peak m/z: 91.0546112060547

Fragment Mass: 91.05426 Da

Fragment Formula: $[C_7H_9\text{-}2H]^+$

9) 272.15857 – C10H20N6O3 MetFrag (R*,S*)-4-[1-Ethyl-2-(4-fluorop+henyl)butyl]phenol

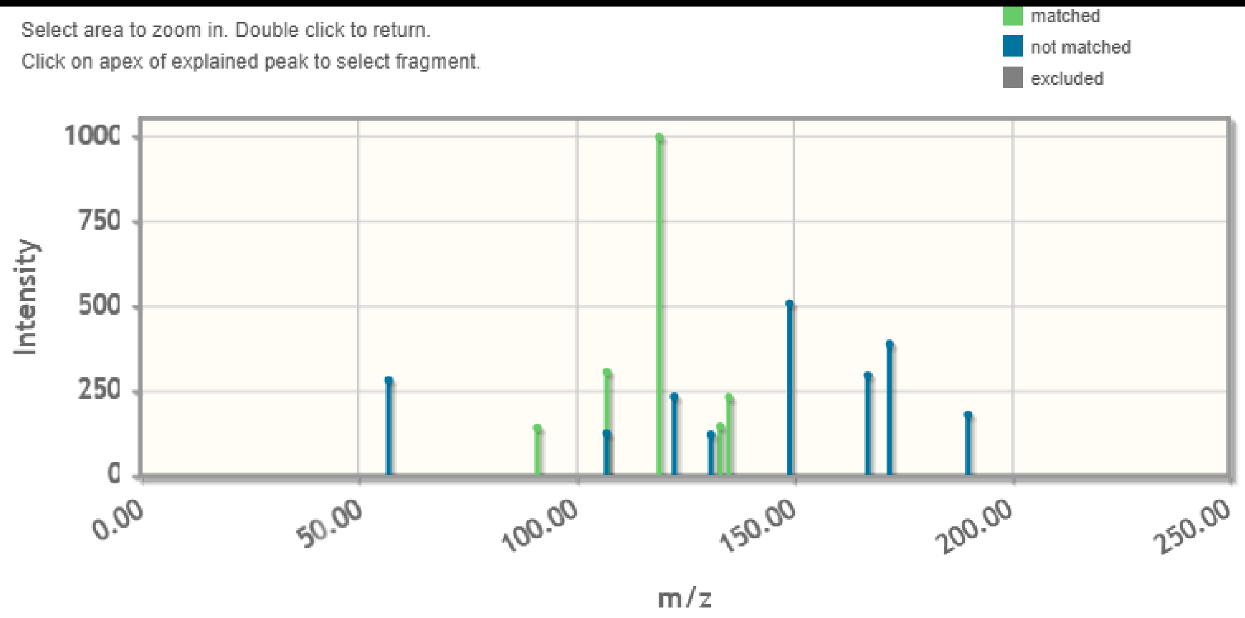

Fragment 1

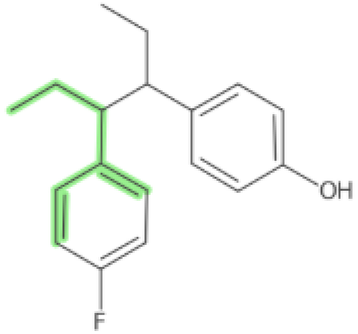

Peak m/z: 91.0546112060547

Fragment Mass: 91.05426 Da

Fragment Formula: $[C_7H_9\text{-}2H]^+$

Fragment 2

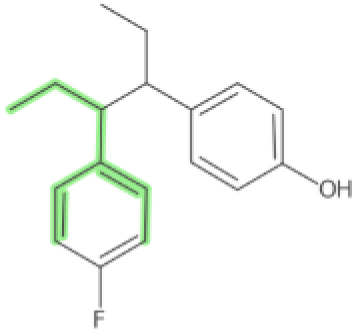

Peak m/z: 107.085807800293

Fragment Mass: 107.08558 Da

Fragment Formula: $[C_8H_{10}]\text{+}H^+$

Fragment 3

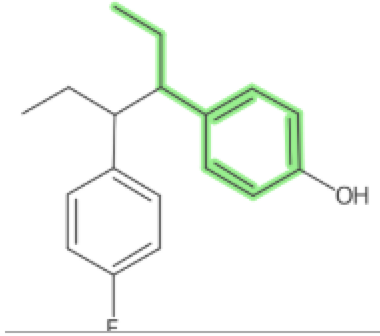

Peak m/z: 119.085716247559

Fragment Mass: 119.08558 Da

Fragment Formula: $[C_9H_{10}]\text{+}H^+$

Fragment 4

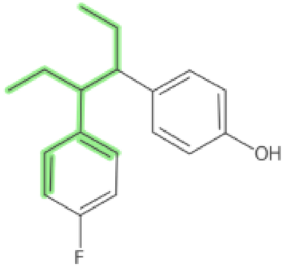

Peak m/z: 133.101150512695

Fragment Mass: 133.10124 Da

Fragment Formula: $[C_{10}H_{15}\text{-}2H]^+$

Fragment 5

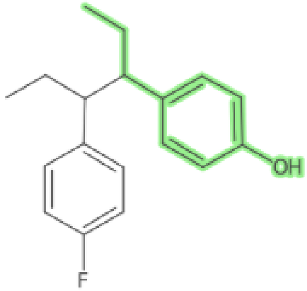

Peak m/z: 135.080383300781

Fragment Mass: 135.08049 Da

Fragment Formula: $[C_9H_{11}O]^+$

9) 272.15857 – C10H20N6O3 MetFrag 1,1,2-Triphenylpropane

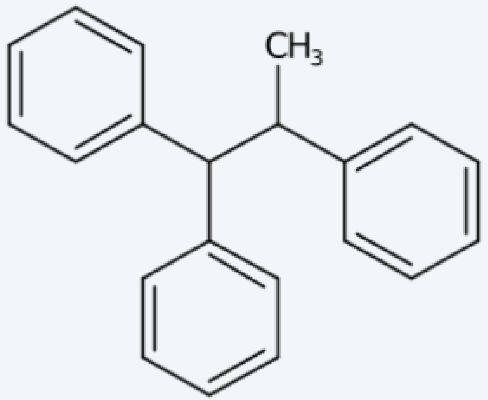

1,1,2-Triphenylpropane

CHEBI:34028<br>InChIKeyBlock1 = UVGJVHUDMMBHRM	272.157	$C_{21}H_{20}$	0.8172	Peaks: 4 / 13<br>Fragments<br>Scores<br>Download

Select area to zoom in. Double click to return.
Click on apex of explained peak to select fragment.

matched
not matched
excluded

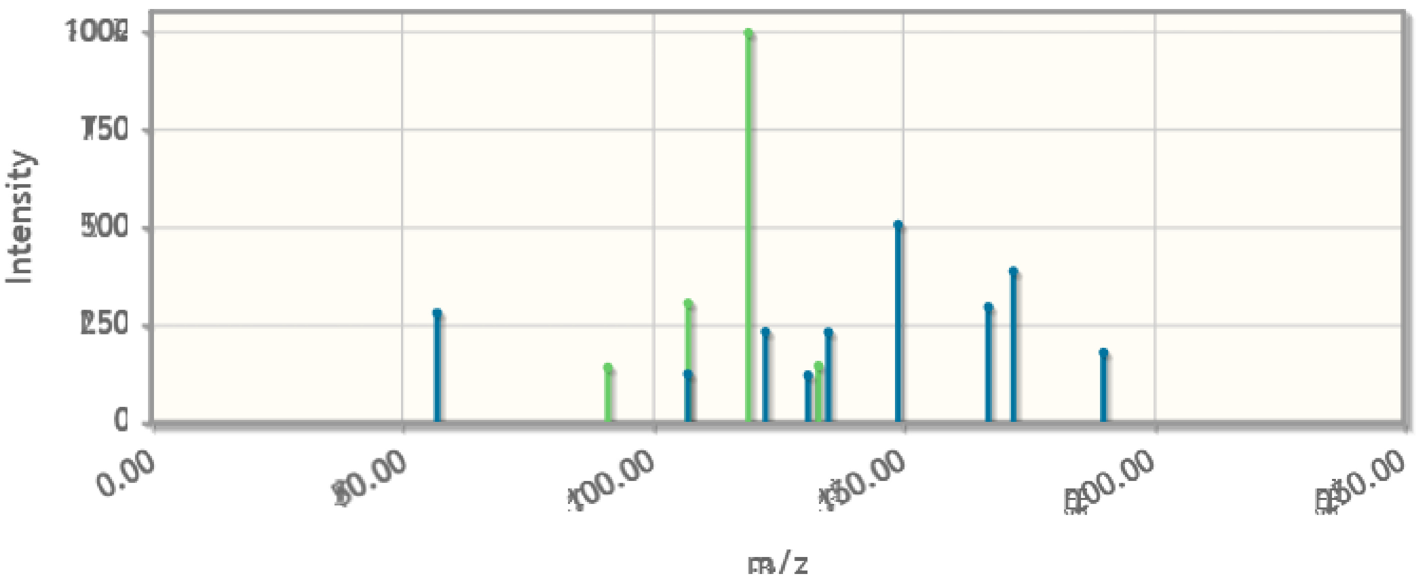

Fragment 1

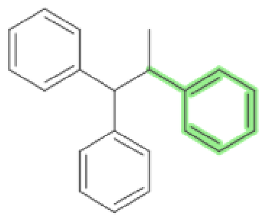

Peak m/z: 91.0546112060547

Fragment Mass: 91.05426 Da

Fragment Formula: $[C_7H_6]+H^+$

Fragment 2

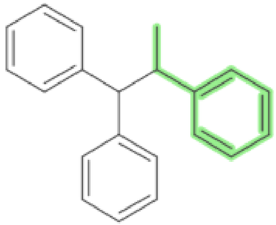

Peak m/z: 107.085807800293

Fragment Mass: 107.08558 Da

Fragment Formula: $[C_8H_9+H]+H^+$

9) 272.15857 – C10H20N6O3 MetFrag 1,1,2-Triphenylpropane

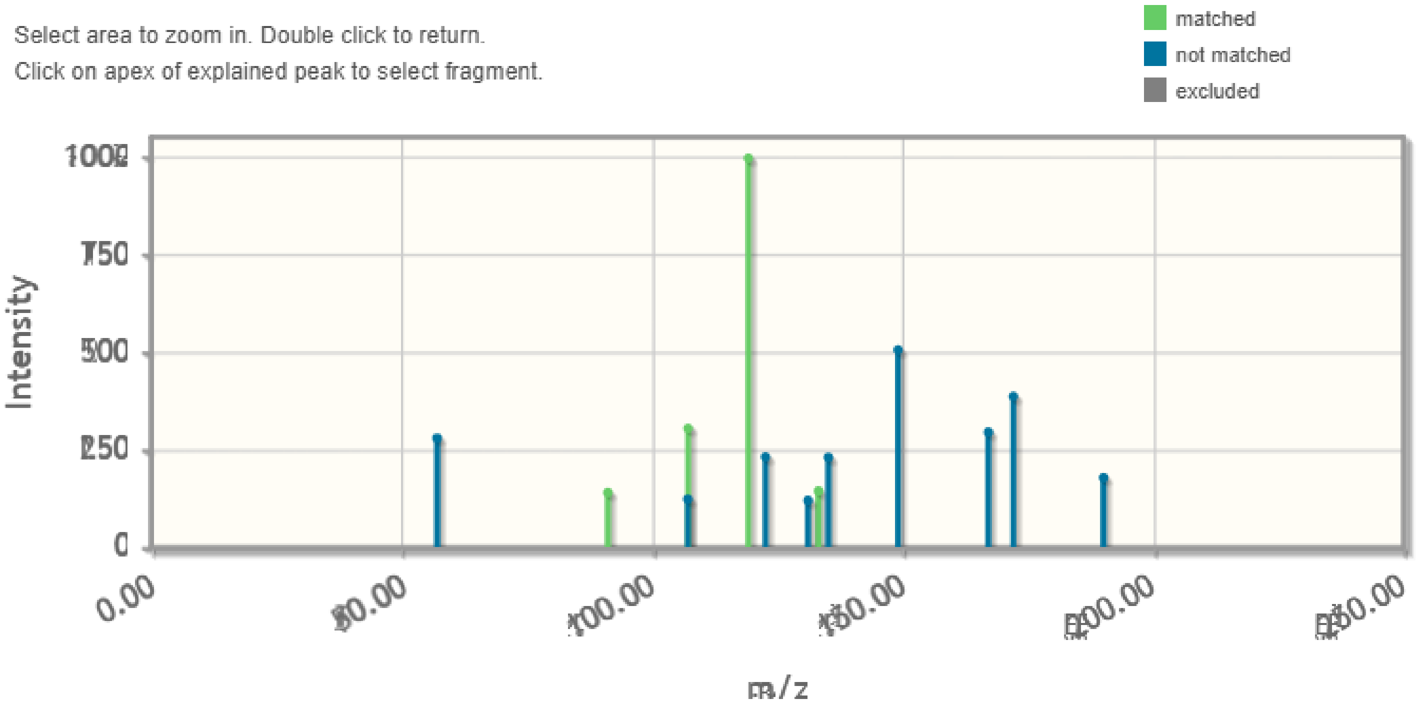

Fragment 1

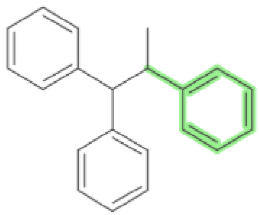

Peak m/z: 91.0546112060547

Fragment Mass: 91.05426 Da

Fragment Formula: $[C_7H_6]+H^+$

Fragment 2

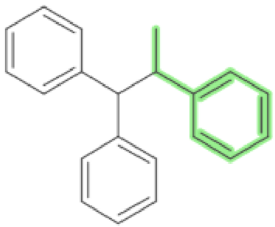

Peak m/z: 107.085807800293

Fragment Mass: 107.08558 Da

Fragment Formula: $[C_8H_9+H]+H^+$

Fragment 3

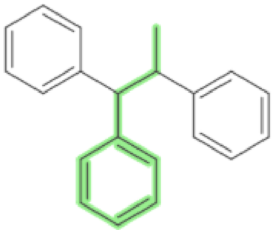

Peak m/z: 119.085716247559

Fragment Mass: 119.08558 Da

Fragment Formula: $[C_9H_{10}]+H^+$

Fragment 4

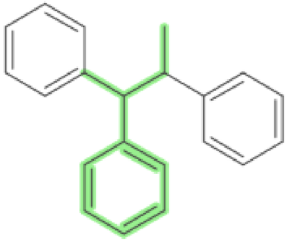

Peak m/z: 133.101150512695

Fragment Mass: 133.10124 Da

Fragment Formula: $[C_{10}H_{10}+2H]+H^+$

9) 272.15857 – C10H20N6O3 MSFinder UNPD129718

Molecular formula finder

Formula	Error [mDa]	Error [ppm]	Score	Resource	Select
Spectral DB search	0.0000	0.0000	5.0000		☑
C14H24O5	2.6503	9.7379	3.8830	UNPD,NPA,COCONUT,MINE	☑
C13H24N2O2S	-3.8745	-14.2363	3.8660	COCONUT,MINE	☑
C15H20N4O	3.9877	14.6518	3.8220	COCONUT,MINE	☑
C16H20N2O2	-7.2457	-26.6237	3.8220	KNApSAcK,ChEBI,PubChem,	☑
C10H20N6O3	-0.0350	-0.1286	3.1630		☑
C10H28N2O2S2	-0.5034	-1.8497	3.1620		☐
C12H16N8	-9.9310	-36.4909	3.1410	COCONUT	☐
C8H25N4O4P	1.6184	5.9465	3.1370		☐
C21H20	-3.2229	-11.8421	3.1100	KNApSAcK,ChEBI,STOFF,UNI	☐
C7H24N6O3S	3.3361	12.2577	3.0820		☐
C6H16N12O	-2.7204	-9.9957	3.0470		☐
C5H20N8O5	-4.0578	-14.9098	3.0440		☐
C11H29O3PS	-2.2211	-8.1611	3.0350		☐

Structure finder

Name	Score (max=10)	Ontology	InChIKey
UNPD129718	6.76	Fatty alcohols	AKVNYEYIRKXNJP-JBGNCALVNA-N
UNPD218984	6.71	C-glycosyl compounds	CCLSDVDYIRVEGS-QOWYYABVSA-N
CNP0446976	6.68	Fatty alcohols	VQGZYKWPLALSMC-UHFFFAOYSA-N
CNP0433812	6.67	Fatty alcohols	HVNQKXWOAYJQBY-UHFFFAOYSA-N
UNPD217745	6.62	Lactones	QEJBWCWBMPNCHV-GVXVVHGQSA-N
UNPD148844	6.44	Fatty alcohol esters	CZKCMXHMOKKOER-XGJSPVFONA-N
UNPD77208	6.42	Gamma butyrolactones	GGZMQQUHNRCSRE-XHSQLUNUNA-N
3,7-dihydroxy-4,4,6,8-tetr	6.17	Medium-chain hydroxy acids and deriv	WBUJALCHSNKAMZ-UHFFFAOYSA-N
UNPD163878	5.97	Medium-chain fatty acids	PJQUDRJHWUHSGO-WEUYFXHZNA-N

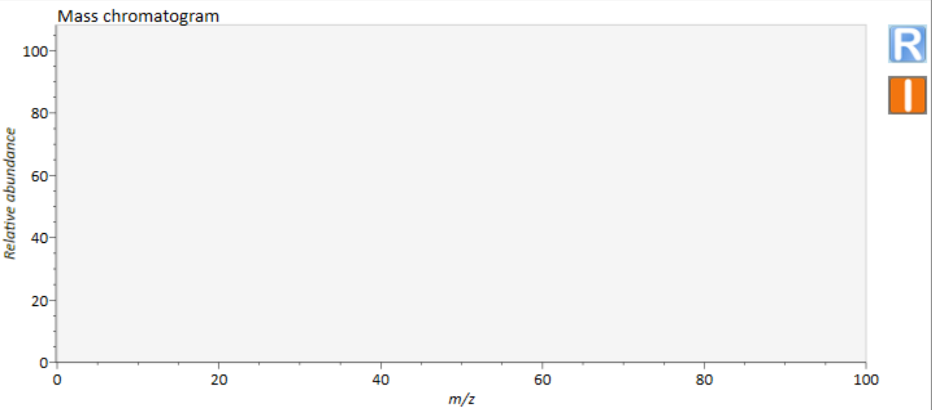

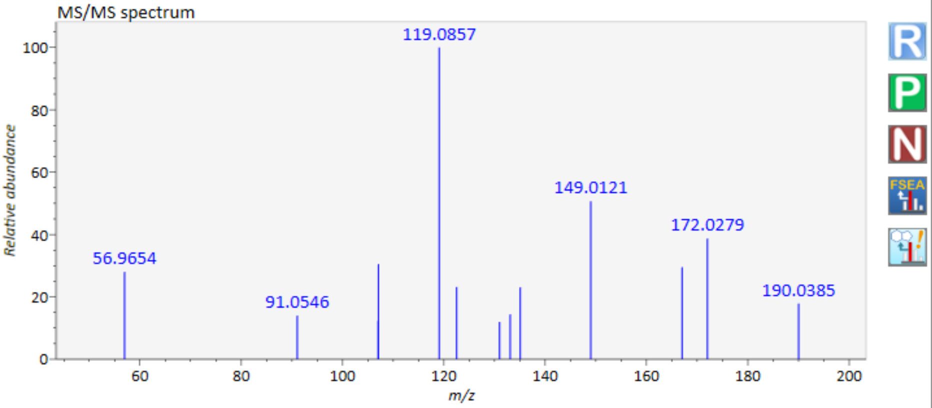

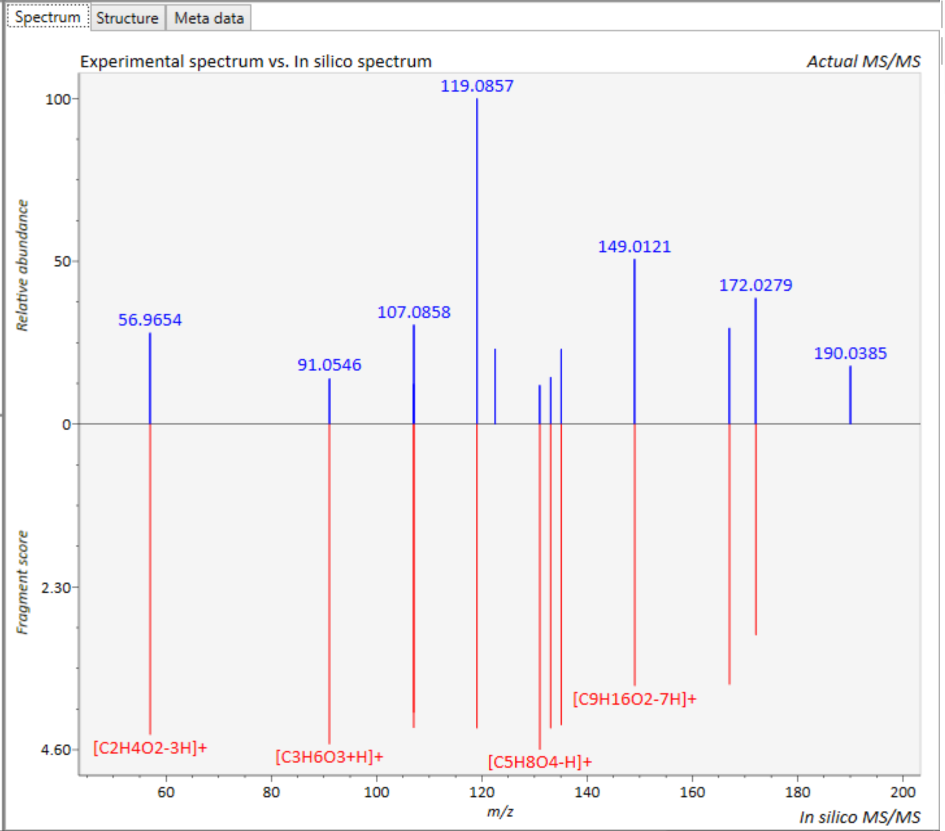

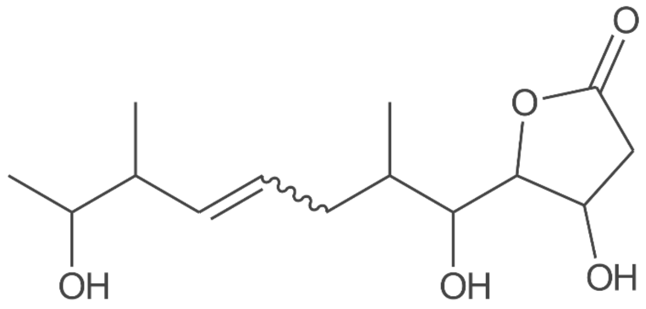

10) 280.12957 – C15H20O5 MetFrag Crispolide

**Identifier**	HMDB36695
**MetFrag**	64.23316888278195
**InChI**	InChI=1S/C15H20O5/c1-8-10-4-3-9-7-15(2,6-5-11(9)20-18)13(16)12(10)19-14(8)17/h3,10-13,16,18H,1,4-7H2,2H3/b9-3-/t10-,11+,12-,13+,15?/m0/s1
**LossStat**	-311.6940550956965
**Monoisot**	280.13107375
**Compoun**	Crispolide
**Identifier**	HMDB36695
**Molecular**	C15H20O5
**SMILES**	CC12CC[C@@H](OO)C(C1)=CC[C@@H]1[C@H](OC(=O)C1=C)[C@H]2O
**SpectralS**	5.741013748563642
**PeakStat**	-111.59131124980028
**ExactSpe**	0.0

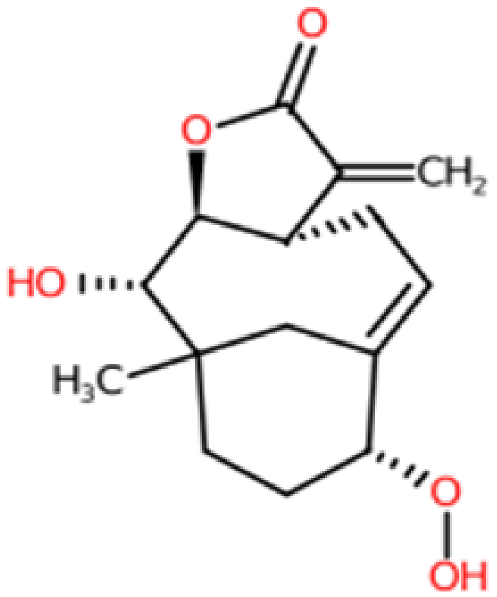

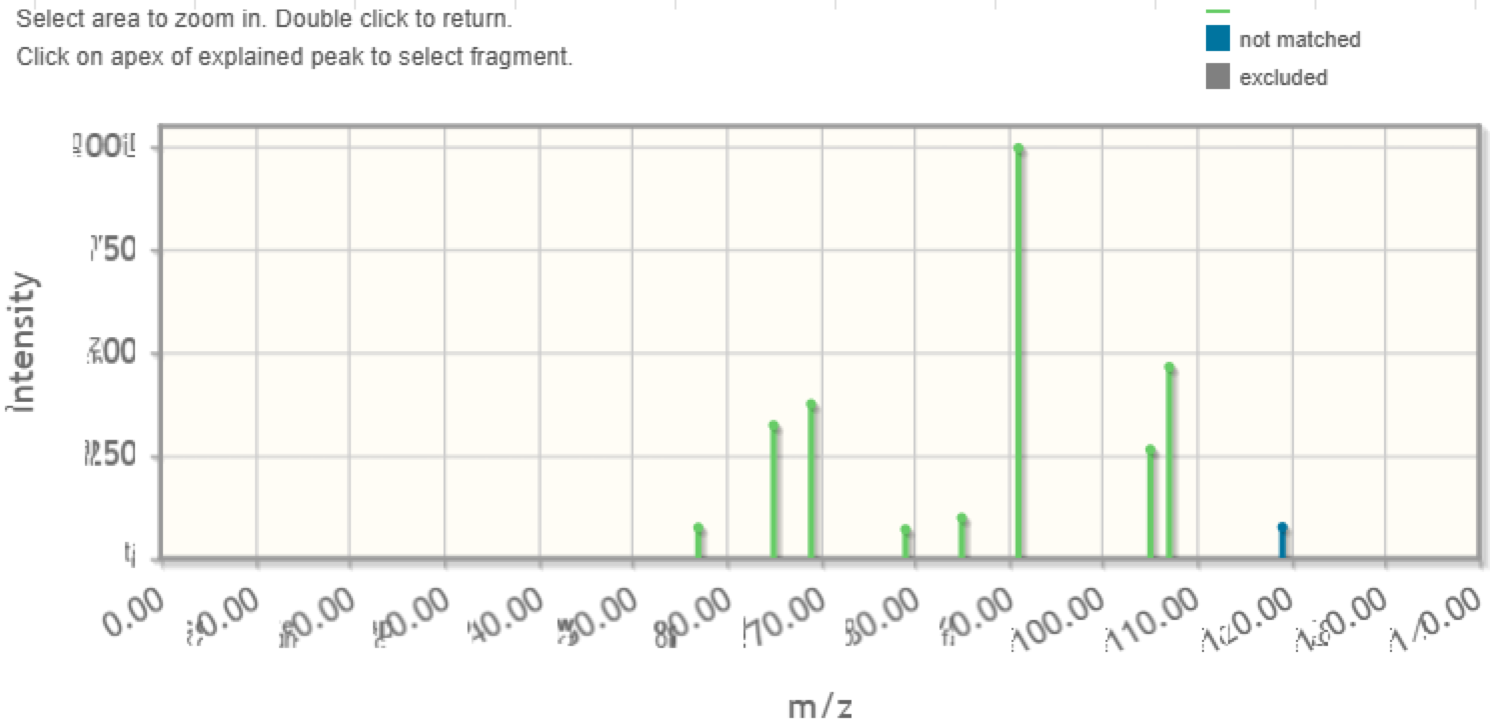

10) 280.12957 – C15H20O5 MetFrag Crispolide

Fragments

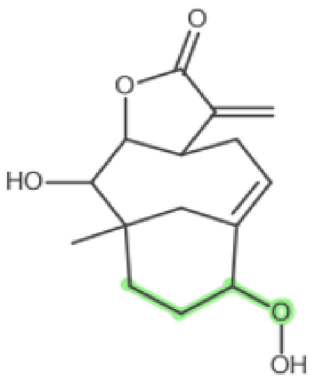

**Fragment 1**	
**Formula**	[C3H5O]+
**Mass**	57.03351
**Peak m/z**	57.0343322753906

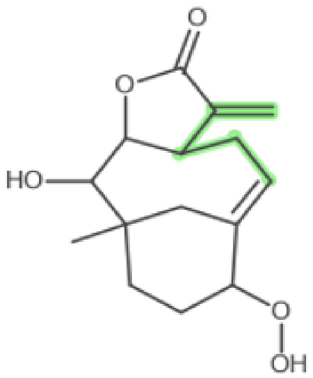

**Fragment 2**	
**Formula**	[C5H6-H]+
**Mass**	65.0386
**Peak m/z**	65.0393981933594

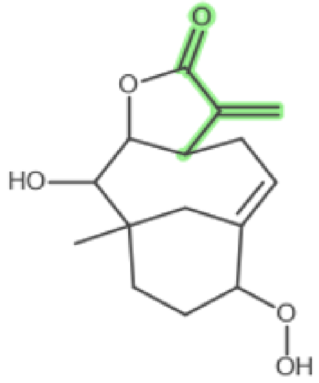

**Fragment 3**	
**Formula**	[C4H3O+H]+H+
**Mass**	69.03351
**Peak m/z**	69.0342864990234

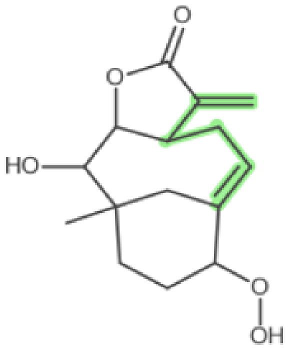

**Fragment 4**	
**Formula**	[C6H6]+H+
**Mass**	79.05426
**Peak m/z**	79.0549621582031

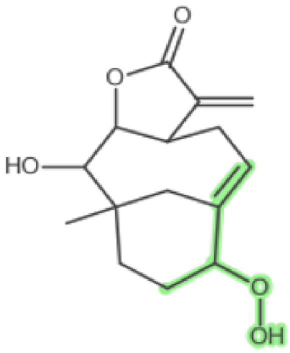

**Fragment 5**	
**Formula**	[C4H5O2]+
**Mass**	85.02842
**Peak m/z**	85.0291366577148

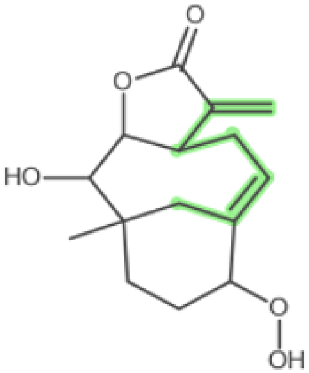

**Fragment 6**	
**Formula**	[C7H8-H]+
**Mass**	91.05426
**Peak m/z**	91.0549240112305

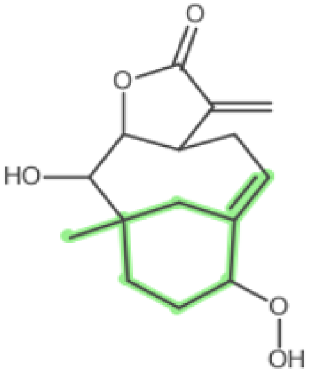

**Fragment 7**	
**Formula**	[C8H11-2H]+
**Mass**	105.06992
**Peak m/z**	105.07048034668

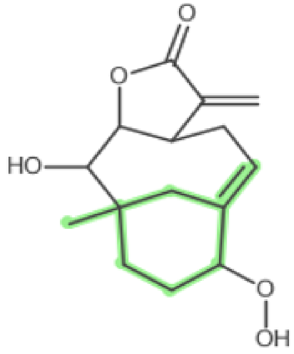

**Fragment 8**	
**Formula**	[C8H11]+
**Mass**	107.08558
**Peak m/z**	107.086120605469

10) 280.12957 – C15H20O5 MSFinder Crispolide

Molecular formula finder

Formula	Error [mDa]	Error [ppm]	Score	Resource	Select
Spectral DB search	0.0000	0.0000	5.0000		☑
C15H20O5	0.3502	1.2501	4.4320	HMDB,KNApSAcK,ChEBI,Foc	☑
C11H16N6O3	-2.3352	-8.3362	4.2820	UNPD,COCONUT,MINE	☑
C16H16N4O	1.6876	6.0243	4.1900	ChEBI,DrugBank,BLEXP,COC	☑
C17H16N2O2	-9.5458	-34.0774	3.9770	ChEBI,PubChem,UNPD,BLEX	☑
C12H24O5S	3.7213	13.2840	3.9190	BLEXP	☑
C14H20N2O2S	-6.1747	-22.0427	3.8500	COCONUT	☐
C22H16	-5.5230	-19.7162	3.7840	UNPD,COCONUT,MINE	☐
C10H20N2O7	-3.6726	-13.1105	3.6350	MINE	☐
C13H20N4OS	5.0587	18.0580	3.5030	MINE	☐
C9H21N4O4P	-0.6818	-2.4339	3.4600		☐
C8H20N6O3S	1.0360	3.6983	3.4570		☐
C11H24N2O2S2	-2.8035	-10.0079	3.4230		☐
C9H29O3PS2	-1.1501	-4.1056	3.4160		☐

Structure finder

Name	Score (max=10)	Ontology	InChIKey
13-Hydroxyabscisic acid	8.04	Abscisic acids and derivatives	AVFORCKFTWHFAR-UMCKCUICNA-N
Nigellic acid	7.92	Abscisic acids and derivatives	ZGHRCSAIMSBFLK-UMCKCUICNA-N
UNPD21179	7.91	Sesquiterpenoids	YVJGXGDZGSMGMH-PLLRQYQGNA-N
Illioliganone B;(-)-Illioliga	7.90	1,3-dioxolanes	LDVFQSQSHHWAPD-OPNJQGHXNA-N
Illioliganone C;(-)-Illioliga	7.87	Fatty alcohols	VONUNFAGLHFGLJ-UYWMCZCJNA-N
UNPD5624	7.86	Monoterpenoids	YLSDOVQJLALFS-SNOQWYRQNA-N
Crispolide	7.85	Germacranolides and derivatives	JXXWNBNYEWOORY-IIHAKNSESA-N
UNPD213174	7.85	Sesquiterpenoids	GLHDTCDWPSSAKH-YJBMVLHGSA-N
UNPD34455	7.85	Ketals	XZZQHLCFYXEGRV-FJBXCPOZNA-N
UNPD103104	7.83	1,2-dioxanes	YGYHRCDXYXLQEH-ACXZQQMLNA-N
UNPD163454	7.83	Phenylpropanoic acids	MQACDUIIGJPLQZ-WPDMJZCKNA-N
UNPD141804	7.82	Germacrane sesquiterpenoids	WBDLQMCDPMDZDU-ILKOCUMGNA-
UNPD114531	7.81	1,2-dioxanes	LBMUHGYTAMVKOW-SQIVJZECNA-N
Limacellone	7.79	Ketals	JJKCMWNJRYLLKG-PTKJDVLHSA-N

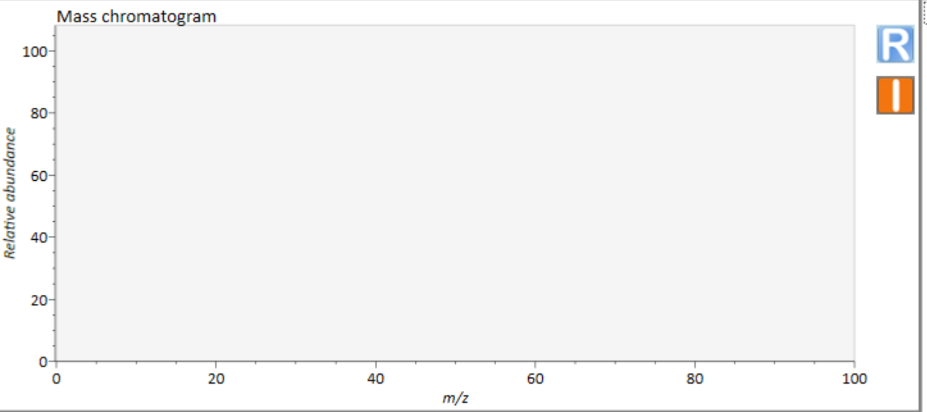

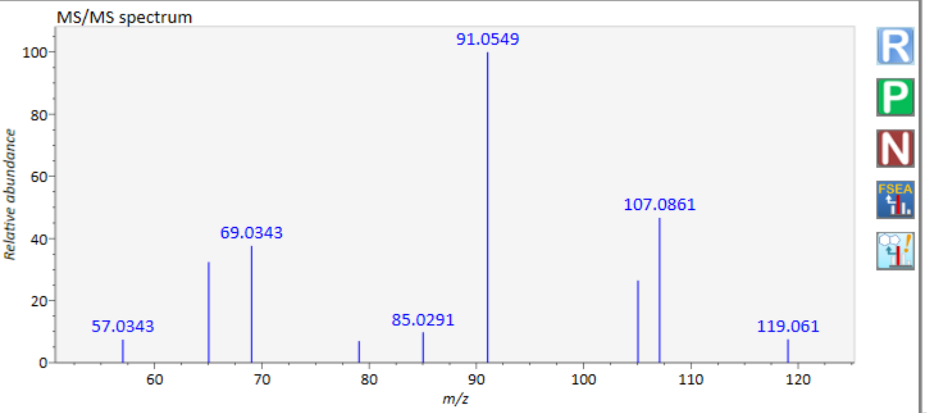

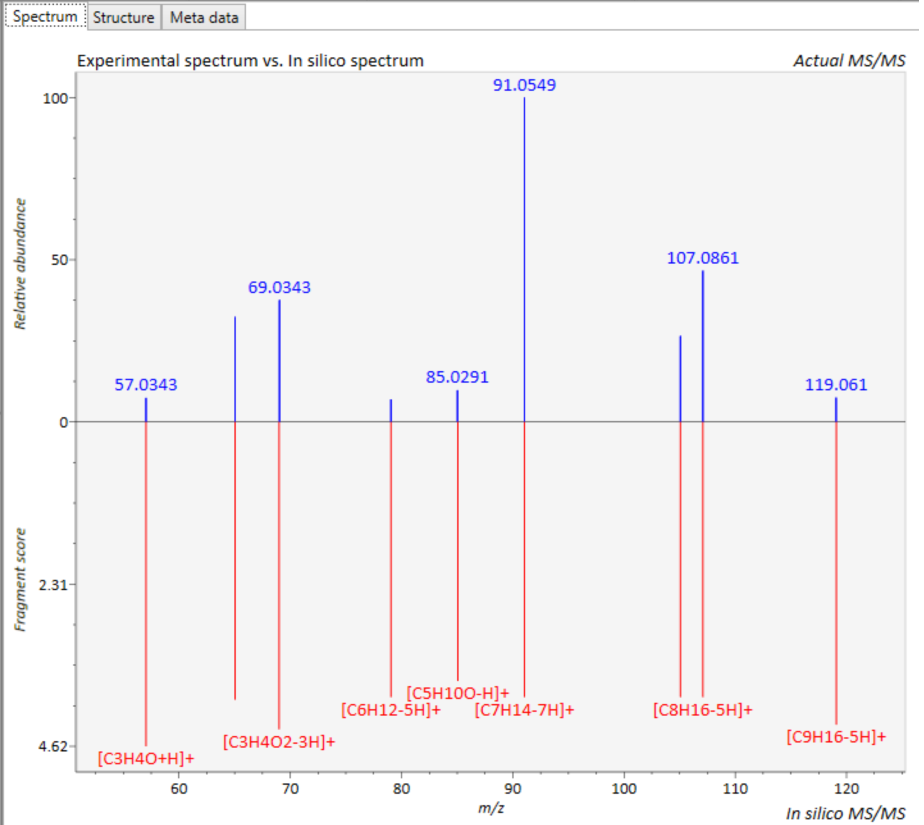

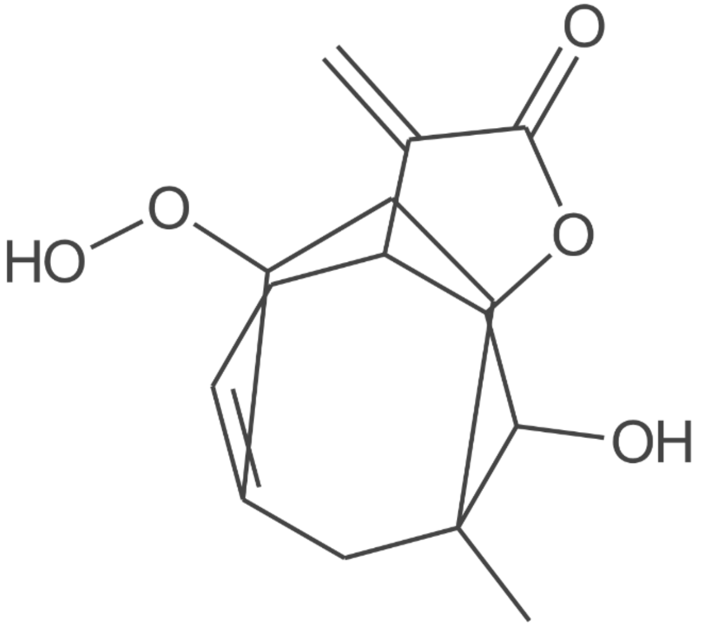

10) 280.12957 – C15H20O5 MetFrag Nigellic acid

4	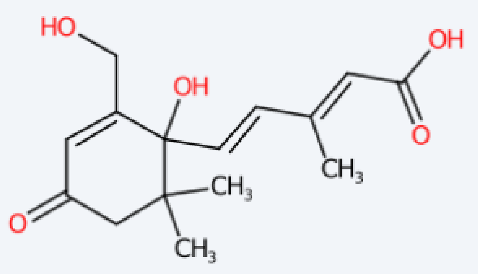<br><br>Nigellic acid	HMDB36094<br>InChIKeyBlock1 = ZGHRCSAIMSBFLK	280.13107	$C_{15}H_{20}O_5$	0.4052	Peaks: 5 / 9<br>Fragments<br>Scores<br>Download

Select area to zoom in. Double click to return.
Click on apex of explained peak to select fragment.

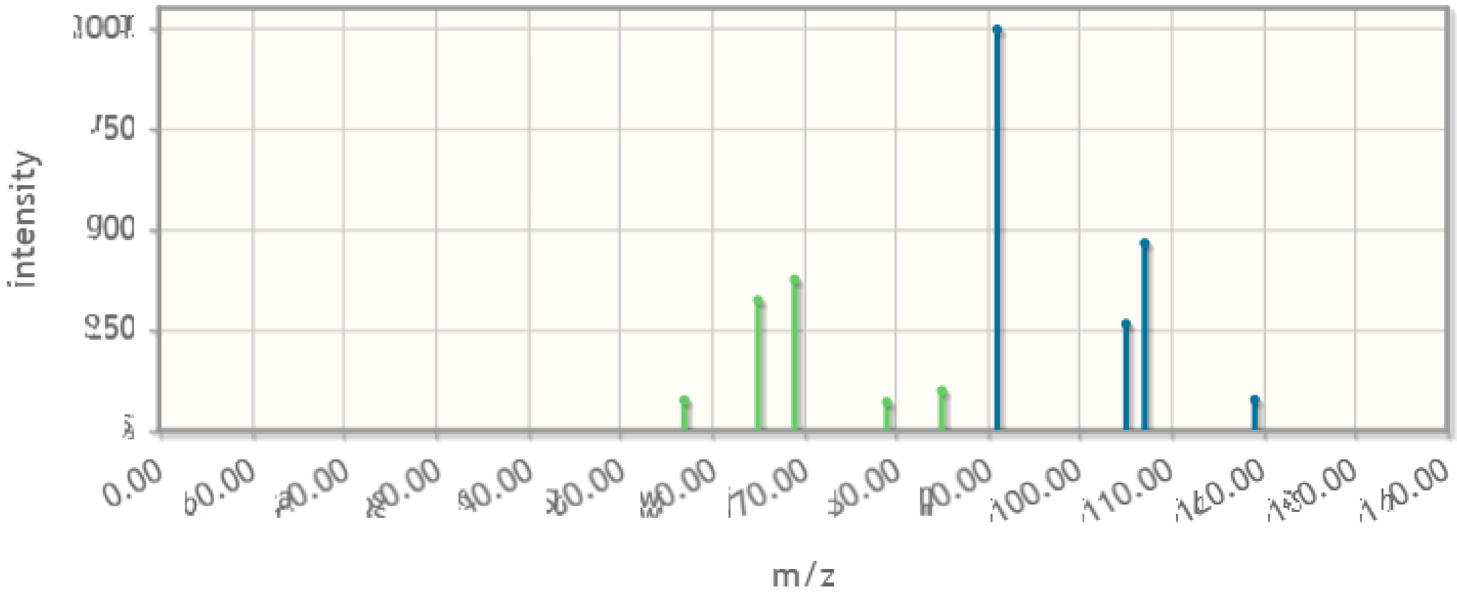

Fragment 1

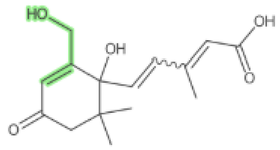

Peak m/z: 57.0343322753906

Fragment Mass: 57.03351 Da

Fragment Formula: $[C_3H_4O]{+}H^+$

Fragment 2

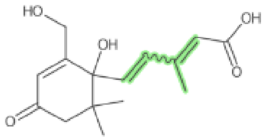

Peak m/z: 65.0393981933594

Fragment Mass: 65.0386 Da

Fragment Formula: $[C_5H_6{-}H]^+$

10) 280.12957 – C15H20O5 MetFrag Nigellic acid

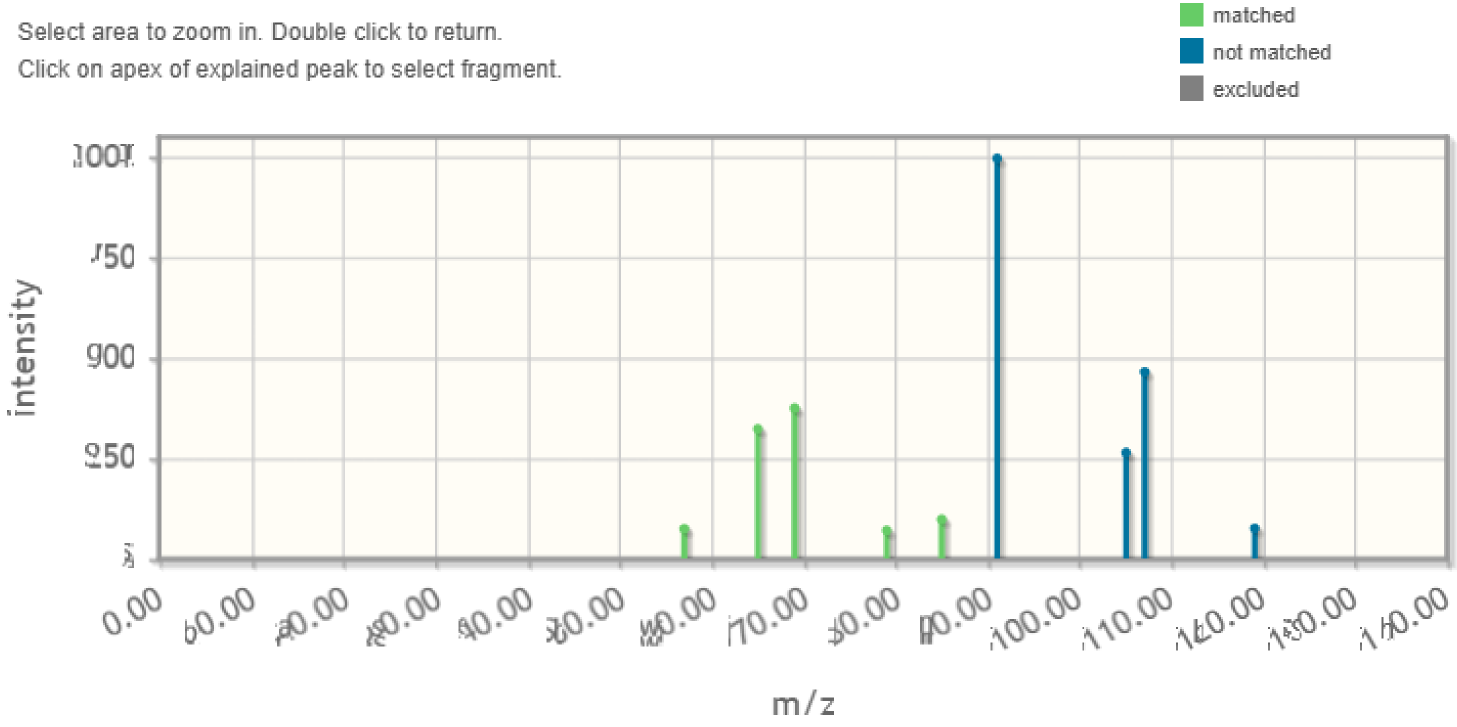

Fragment 1

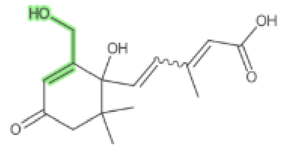

Peak m/z: 57.0343322753906

Fragment Mass: 57.03351 Da

Fragment Formula: $[C_3H_4O]{+}H^+$

Fragment 2

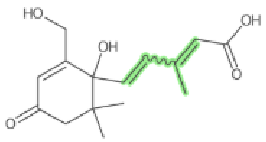

Peak m/z: 65.0393981933594

Fragment Mass: 65.0386 Da

Fragment Formula: $[C_5H_6{-}H]^+$

Fragment 3

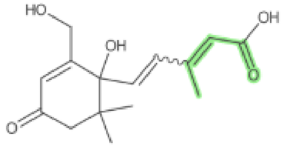

Peak m/z: 69.0342864990234

Fragment Mass: 69.03351 Da

Fragment Formula: $[C_4H_4O]{+}H^+$

Fragment 4

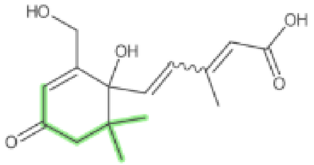

Peak m/z: 79.0549621582031

Fragment Mass: 79.05426 Da

Fragment Formula: $[C_6H_9{-}2H]^+$

Fragment 5

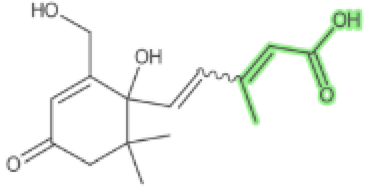

Peak m/z: 85.0291366577148

Fragment Mass: 85.02842 Da

Fragment Formula: $[C_4H_5O_2]^+$

10) 280.12957 – C15H20O5 MSFinder Nigellic acid

Formula	Error [mDa]	Error [ppm]	Score	Resource	Select
Spectral DB search	0.0000	0.0000	5.0000		☑
C15H20O5	0.3502	1.2501	4.4320	HMDB,KNApSAcK,ChEBI,Foo	☑
C11H16N6O3	-2.3352	-8.3362	4.2820	UNPD,COCONUT,MINE	☑
C16H16N4O	1.6876	6.0243	4.1900	ChEBI,DrugBank,BLEXP,COC	☑
C17H16N2O2	-9.5458	-34.0774	3.9770	ChEBI,PubChem,UNPD,BLEX	☑
C12H24O5S	3.7213	13.2840	3.9190	BLEXP	☑
C14H20N2O2S	-6.1747	-22.0427	3.8500	COCONUT	☐
C22H16	-5.5230	-19.7162	3.7840	UNPD,COCONUT,MINE	☐
C10H20N2O7	-3.6726	-13.1105	3.6350	MINE	☐
C13H20N4OS	5.0587	18.0580	3.5030	MINE	☐
C9H21N4O4P	-0.6818	-2.4339	3.4600		☐
C8H20N6O3S	1.0360	3.6983	3.4570		☐
C11H24N2O2S2	-2.8035	-10.0079	3.4230		☐
C9H29O3PS2	-1.1501	-4.1056	3.4160		☐

Name	Score (max=10)	Ontology	InChIKey
13-Hydroxyabscisic acid	8.04	Abscisic acids and derivatives	AVFORCKFTWHFAR-UMCKCUICNA-N
Nigellic acid	7.92	Abscisic acids and derivatives	ZGHRCSAIMSBFLK-UMCKCUICNA-N
UNPD21179	7.91	Sesquiterpenoids	YVJGXGDZGSMGMH-PLLRQYQGNA-N
Illioliganone B;(-)-Illioliga	7.90	1,3-dioxolanes	LDVFQSQSHHWAPD-OPNJQGHXNA-N
Illioliganone C;(-)-Illioliga	7.87	Fatty alcohols	VONUNFAGLHFGLJ-UYWMCZCJNA-N
UNPD5624	7.86	Monoterpenoids	YLSDOVQJJLALFS-SNOQWYRQNA-N
Crispolide	7.85	Germacranolides and derivatives	JXXWNBNYEWOORY-IIHAKNSESA-N
UNPD213174	7.85	Sesquiterpenoids	GLHDTCDWPSSAKH-YJBMVLHGSA-N
UNPD34455	7.85	Ketals	XZZQHLCFYXEGRV-FJBXCPOZNA-N
UNPD103104	7.83	1,2-dioxanes	YGYHRCDXYXLQEH-ACXZQQMLNA-N
UNPD163454	7.83	Phenylpropanoic acids	MQACDUIIGJPLQZ-WPDMJZCKNA-N
UNPD141804	7.82	Germacrane sesquiterpenoids	WBDLQMCDPMDZDU-ILKOCUMGNA-
UNPD114531	7.81	1,2-dioxanes	LBMUHGYTAMVKOW-SQIVJZECNA-N
Limacellone	7.79	Ketals	JJKCMWNJRYLLKG-PTKJDVLHSA-N

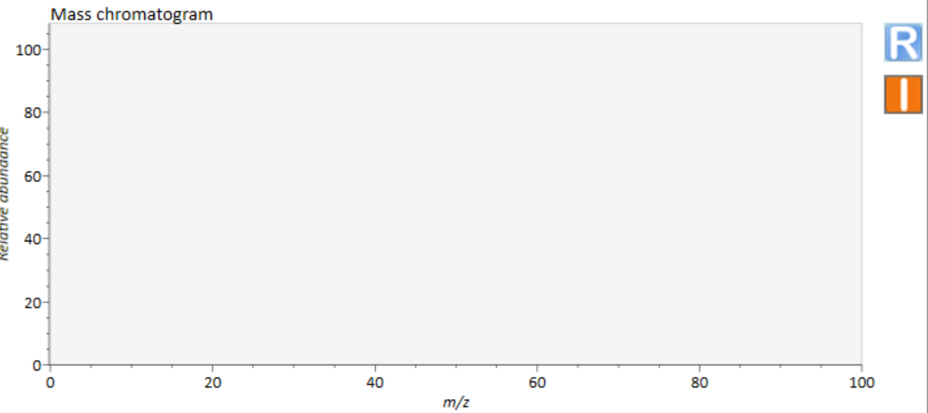

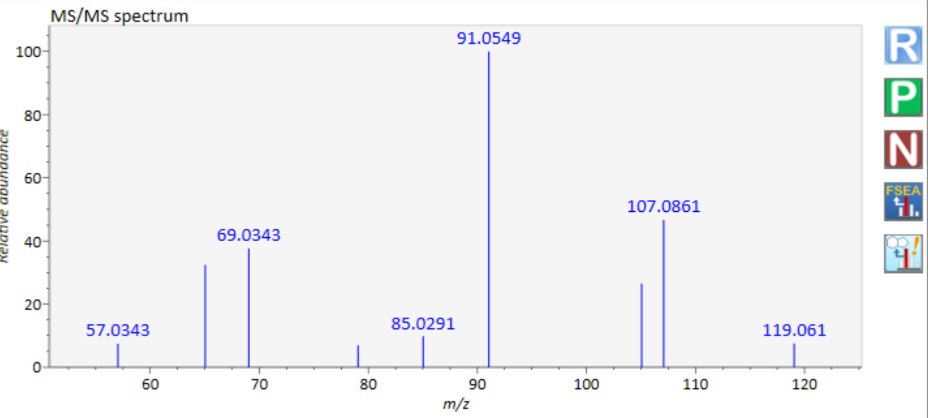

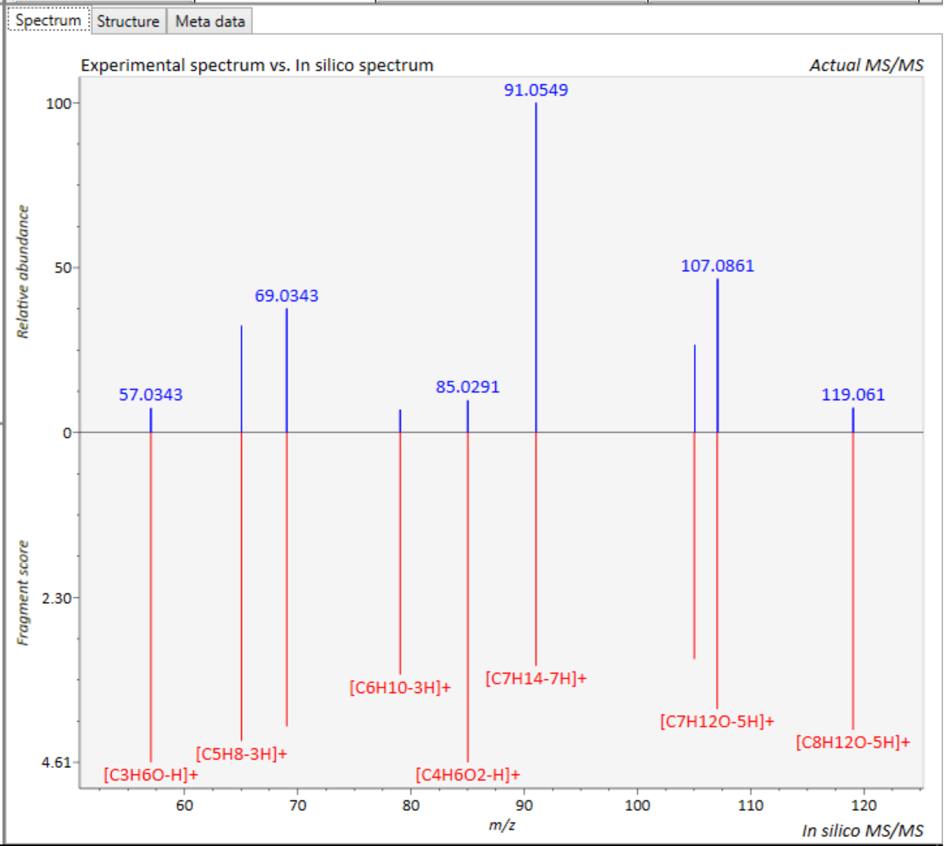

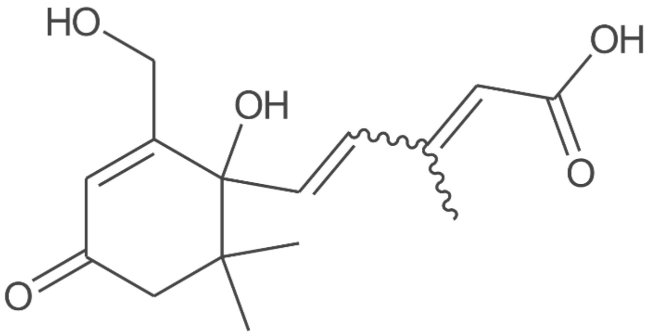

10) 280.12957 – C15H20O5 MetFrag 13-Hydroxyabscisic acid

5	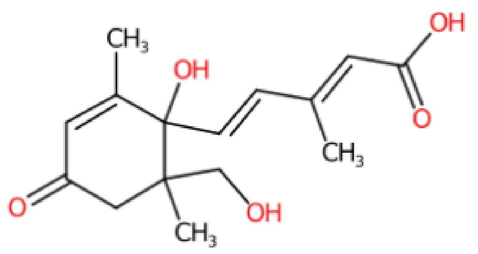 13-Hydroxyabscisic acid	HMDB36095<br>InChIKeyBlock1 = AVFORCKFTWHFAR	280.13107	$C_{15}H_{20}O_5$	0.3672	Peaks: 4 / 9<br>Fragments<br>Scores<br>Download

Select area to zoom in. Double click to return.
Click on apex of explained peak to select fragment.

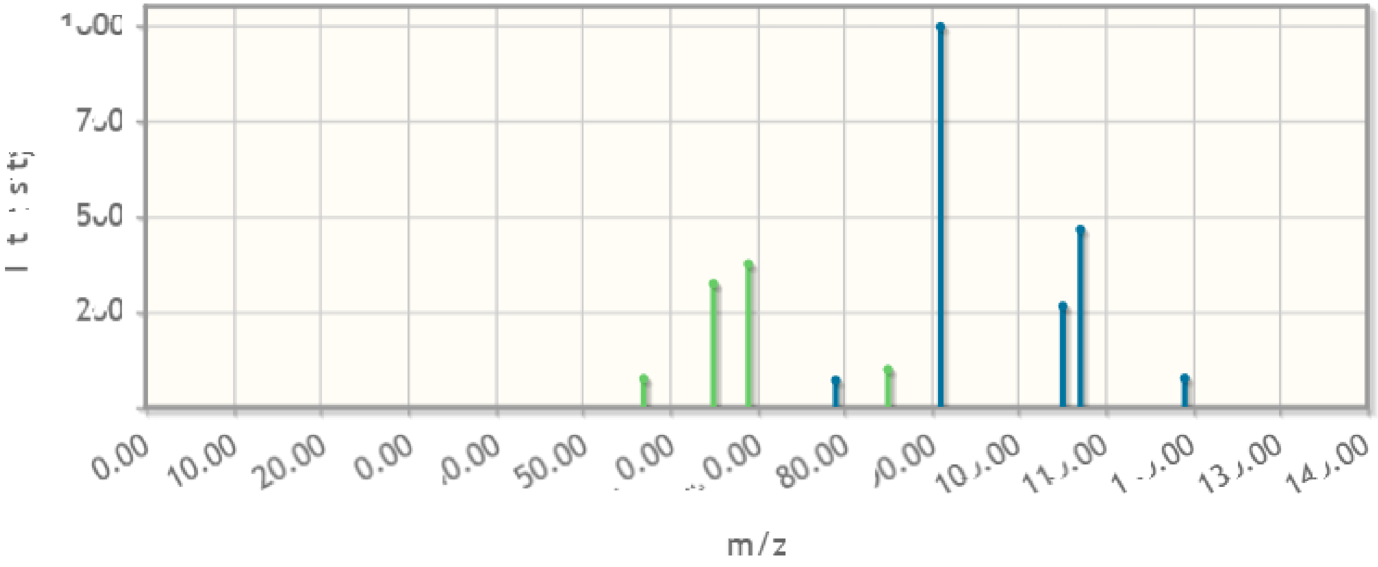

Fragment 1

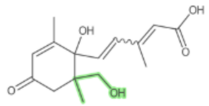

Peak m/z: 57.0343322753906
Fragment Mass: 57.03351 Da
Fragment Formula: $[C_3H_6O\text{-}H]^+$

Fragment 2

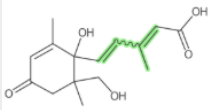

Peak m/z: 65.0393981933594
Fragment Mass: 65.0386 Da
Fragment Formula: $[C_5H_6\text{-}H]^+$

Fragment 3

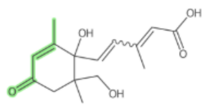

Peak m/z: 69.0342864990234
Fragment Mass: 69.03351 Da
Fragment Formula: $[C_4H_4O]\text{+}H^+$

Fragment 4

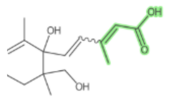

Peak m/z: 85.0291366577148
Fragment Mass: 85.02842 Da
Fragment Formula: $[C_4H_5O_2]^+$

10) 280.12957 – $C_{15}H_{20}O_5$ MSFinder 13-Hydroxyabscisic acid

Molecular formula finder

Formula	Error [mDa]	Error [ppm]	Score	Resource	Select
Spectral DB search	0.0000	0.0000	5.0000		☑
C15H20O5	0.3502	1.2501	4.4320	HMDB,KNApSAcK,ChEBI,Foc	☑
C11H16N6O3	-2.3352	-8.3362	4.2820	UNPD,COCONUT,MINE	☑
C16H16N4O	1.6876	6.0243	4.1900	ChEBI,DrugBank,BLEXP,COC	☑
C17H16N2O2	-9.5458	-34.0774	3.9770	ChEBI,PubChem,UNPD,BLEX	☑
C12H24O5S	3.7213	13.2840	3.9190	BLEXP	☑
C14H20N2O2S	-6.1747	-22.0427	3.8500	COCONUT	☐
C22H16	-5.5230	-19.7162	3.7840	UNPD,COCONUT,MINE	☐
C10H20N2O7	-3.6726	-13.1105	3.6350	MINE	☐
C13H20N4OS	5.0587	18.0580	3.5030	MINE	☐
C9H21N4O4P	-0.6818	-2.4339	3.4600		☐
C8H20N6O3S	1.0360	3.6983	3.4570		☐
C11H24N2O2S2	-2.8035	-10.0079	3.4230		☐
C9H29O3PS2	-1.1501	-4.1056	3.4160		☐

Structure finder

Name	Score (max=10)	Ontology	InChIKey
13-Hydroxyabscisic acid	8.04	Abscisic acids and d	AVFORCKFTWHFAR
Nigellic acid	7.92	Abscisic acids and d	ZGHRCSAIMSBFLK-
UNPD21179	7.91	Sesquiterpenoids	YVJGXGDZGSMGMI
Illioliganone B;(-)-Illioliganone B	7.90	1,3-dioxolanes	LDVFQSQSHHWAPI
Illioliganone C;(-)-Illioliganone C	7.87	Fatty alcohols	VONUNFAGLHFGLJ
UNPD5624	7.86	Monoterpenoids	YLSDOVQJJLALFS-S
Crispolide	7.85	Germacranolides ar	JXXWNBNYEWOOR
UNPD213174	7.85	Sesquiterpenoids	GLHDTCDWPSSAKH
UNPD34455	7.85	Ketals	XZZQHLCFYXEGRV-
UNPD103104	7.83	1,2-dioxanes	YGYHRCDXYXLQEH
UNPD163454	7.83	Phenylpropanoic ac	MQACDUIIGJPLQZ-
UNPD141804	7.82	Germacrane sesqui	WBDLQMCDPMDZI
UNPD114531	7.81	1,2-dioxanes	LBMUHGYTAMVKO
Limacellone	7.79	Ketals	JJKCMWNJRYLLKG-

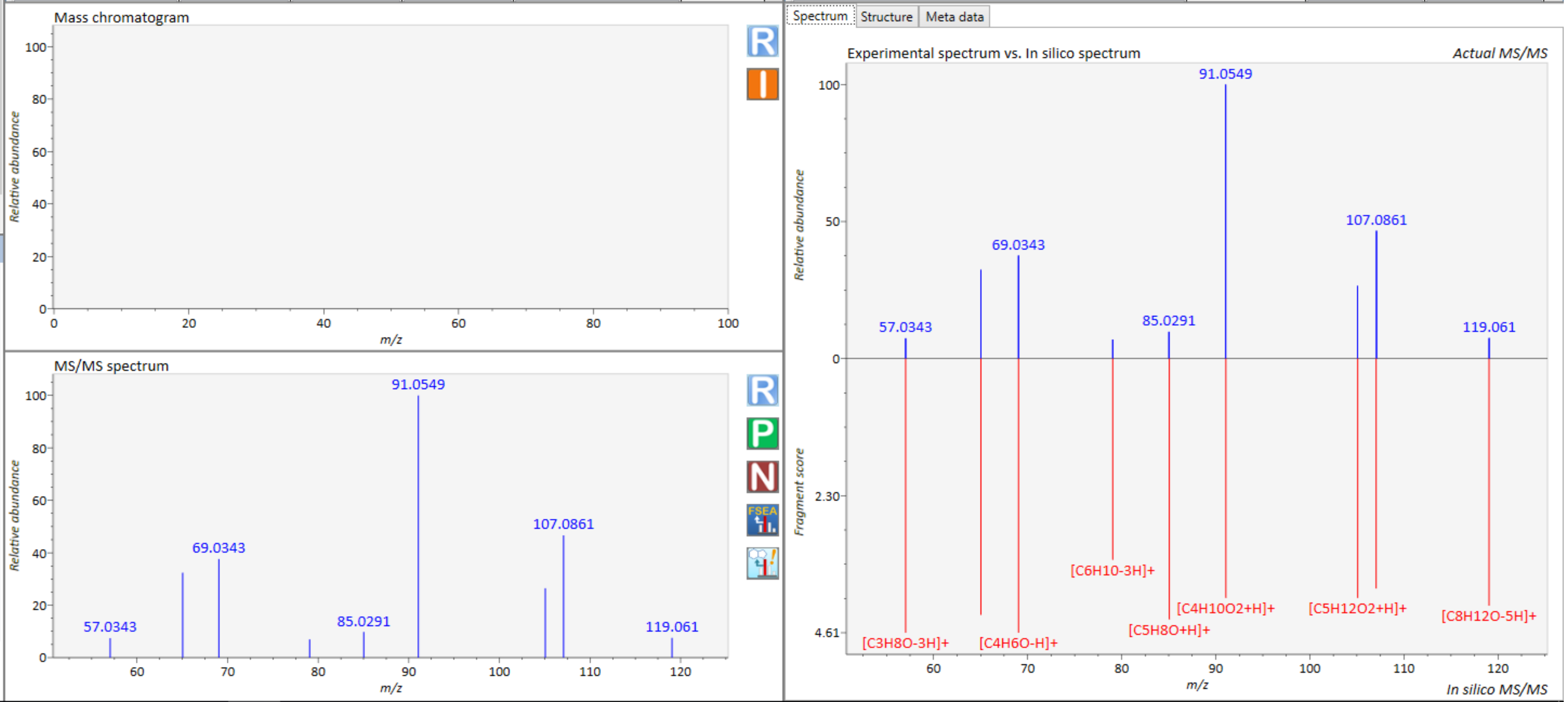

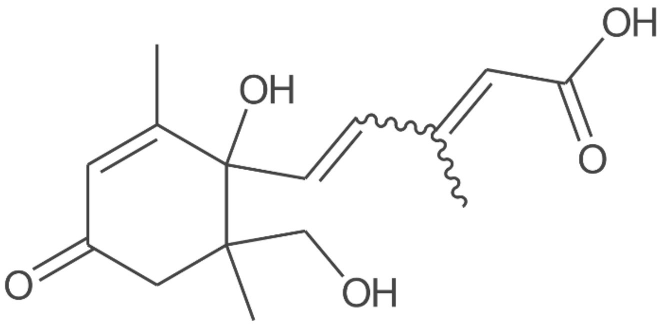

11) 282.10018 – C14H19O4P MetFrag 8-phenyloct-3-ynyl dihydrogen phosphate

**Identifier**	54437192
**MetFrag**	177.60577226167163
**InChI**	InChI=1S/C14H19O4P/c15-19(16,17)18-13-9-4-2-1-3-6-10-14-11-7-5-8-12-14/h5,7-8,11-12H,1,3,6,9-10,13H2,(H2,15,16,17)
**LossStat**ʻ	-4750.133351658279
**Monoisot**ʻ	282.102
**Compoun**	8-phenyloct-3-ynyl dihydrogen phosphate
**Identifier**	54437192
**Molecular**	C14H19O4P
**SMILES**	C1=CC=C(C=C1)CCCCC#CCCOP(=O)(O)O
**SpectralS**	0.8950105768856457
**PeakStat**ʻ	-273.52848920600485
**ExactSpe**	0.0

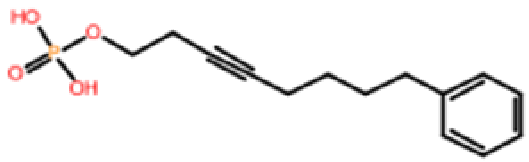

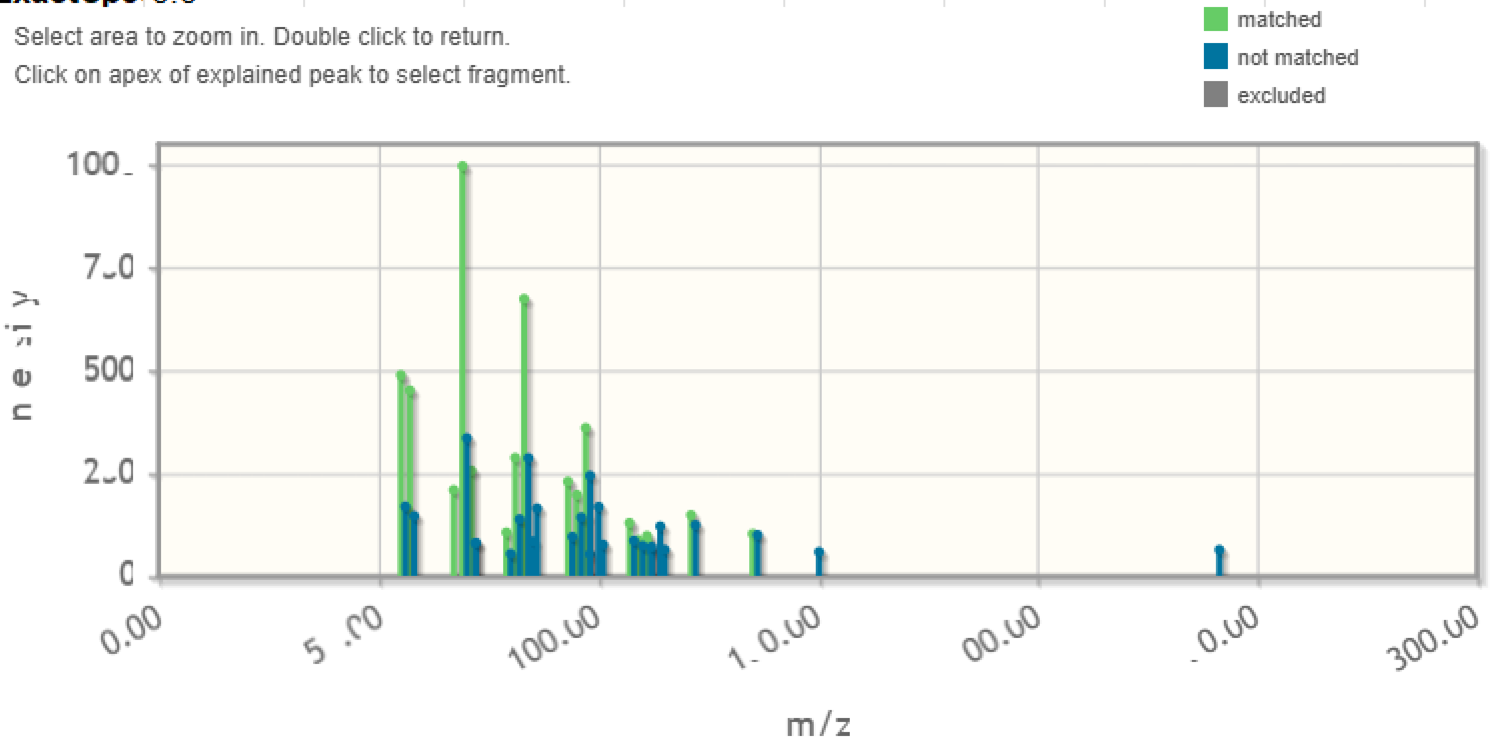

11) 282.10018 – C14H19O4P MetFrag 8-phenyloct-3-ynyl dihydrogen phosphate

Identifier	54437192
MetFrag	177.60577226167163
InChI	InChI=1S/C14H19O4P/c15-19(16,17)18-13-9-4-2-1-3-6-10-14-11-7-5-8-12-14/h5,7-8,11-12H,1,3,6,9-10,13H2,(H2,15,16,17)
LossStat	-4750.133351658279
Monoisot	282.102
Compoun	8-phenyloct-3-ynyl dihydrogen phosphate
Identifier	54437192
Molecular	C14H19O4P
SMILES	C1=CC=C(C=C1)CCCCC#CCCOP(=O)(O)O
SpectralS	0.8950105768856457
PeakStat	-273.52848920600485
ExactSpe	0.0

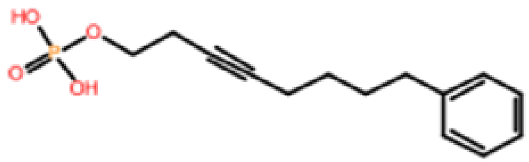

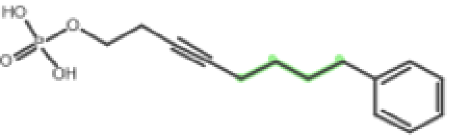

Fragment 1	
Formula	[C4H8-H]+
Mass	55.05426
Peak m/z	55.0550384521484

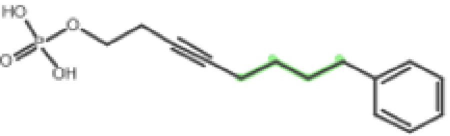

Fragment 2	
Formula	[C4H8]+H+
Mass	57.06992
Peak m/z	57.0706787109375

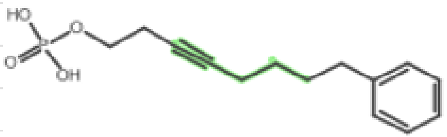

Fragment 3	
Formula	[C5H6]+H+
Mass	67.05426
Peak m/z	67.0549926757813

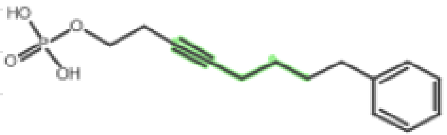

Fragment 4	
Formula	[C5H6+2H]+H+
Mass	69.06992
Peak m/z	69.0706329345703

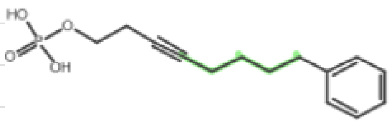

Fragment 5	
Formula	[C5H8+2H]+H+
Mass	71.08558
Peak m/z	71.0862731933594

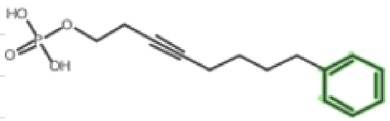

Fragment 6	
Formula	[C6H5+H]+H+
Mass	79.05426
Peak m/z	79.0549468994141

11) 282.10018 – C14H19O4P MetFrag 8-phenyloct-3-ynyl dihydrogen phosphate

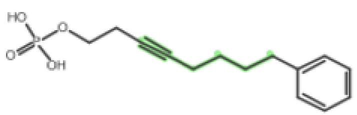

Fragment 7	
Formula	[C6H8]+H+
Mass	81.06992
Peak m/z	81.0705871582031

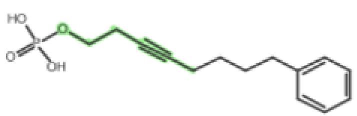

Fragment 8	
Formula	[C5H6O]+H+
Mass	83.04917
Peak m/z	83.0498123168945

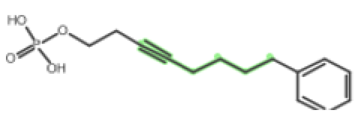

Fragment 9	
Formula	[C6H8+2H]+H+
Mass	83.08558
Peak m/z	83.0862045288086

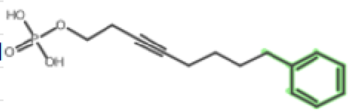

Fragment 10	
Formula	[C7H7+H]+H+
Mass	93.06992
Peak m/z	93.0704803466797

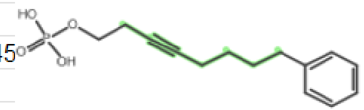

Fragment 11	
Formula	[C7H10]+H+
Mass	95.08558
Peak m/z	95.0860977172852

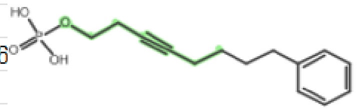

Fragment 12	
Formula	[C6H8O]+H+
Mass	97.06483
Peak m/z	97.0653991699219

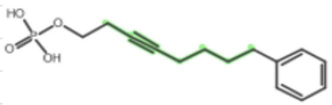

Fragment 13	
Formula	[C7H10+2H]+H+
Mass	97.10124
Peak m/z	97.1017684936523

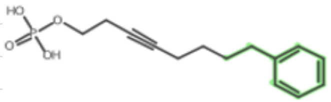

Fragment 14	
Formula	[C8H9+H]+H+
Mass	107.08558
Peak m/z	107.086036682129

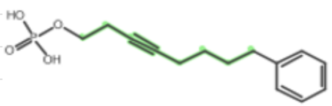

Fragment 15	
Formula	[C8H12]+H+
Mass	109.10124
Peak m/z	109.101669311523

Fragment 19	
Formula	[C10H13+H]+H+
Mass	135.1169
Peak m/z	135.117156982422

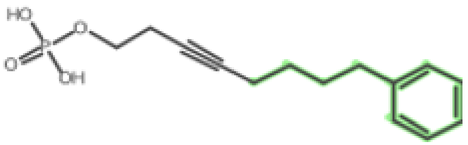

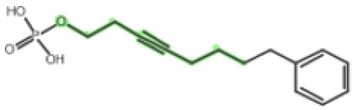

Fragment 16	
Formula	[C7H10O]+H+
Mass	111.08049
Peak m/z	111.080917358398

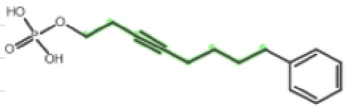

Fragment 17	
Formula	[C8H12+2H]+H+
Mass	111.1169
Peak m/z	111.117286682129

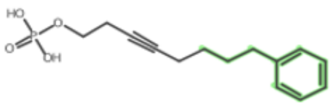

Fragment 18	
Formula	[C9H11+H]+H+
Mass	121.10124
Peak m/z	121.101669311523

11) 282.10018 – C14H19O4P Sirius 8-phenyloct-3-ynyl dihydrogen phosphate

YMDB YMDB Mine ZINC bio

Rank	Name	SMILES	Molecular Formula	Adduct	CSI:FingerID Score	Tanimoto Similarity	#PubMed IDs	XLogP	InChIKey	Lipid Class
1	Di(3-butenyl) phenyl phos...	C=CCCOP(=O)(OCCC=C...	C14H19O4P	[M + H]+	-123.666	40.476%		3.594	JMGQLNXBPAMTPR	
2	null	CCOP(=O)(CC1=CC=C(...	C14H19O4P	[M + H]+	-124.924	33.163%		1.800	CFOFNXCUJXNHIY	
3	null	CCOC1=CC=C(C=C1)P(...	C14H19O4P	[M + H]+	-128.208	34.066%		2.700	DHCXOMFZGQXPFD	
4	null	CCOP(=O)(OCC)OCC=C...	C14H19O4P	[M + H]+	-135.604	35.366%		2.000	UIBZIPXFMOGEBC	
5	null	C1=CC=C(C=C1)CCCC...	C14H19O4P	[M + H]+	-146.318	37.143%		2.500	WLOISDPJEFIZHZ	
6	(E)-4-diethoxyphosphoryl-...	CCOP(=O)(CC=CC(=O)C...	C14H19O4P	[M + H]+	-150.801	39.153%		1.800	AMDWVFYIJFVNGY	
7	3-(diethoxyphosphorylmet...	CCOP(=O)(CC1=COC2=...	C14H19O4P	[M + H]+	-152.363	31.696%		2.500	NKVYYLYMEFXHED	
8	null	CCC(=C)C#CC(=C)C(C(...	C14H19O4P	[M + H]+	-153.015	49.664%		3.000	NREVNEDLGOEAJP	
9	2-diethoxyphosphoryl-3,4-	CCOP(=O)(C1CCC2=CC	C14H19O4P	[M + H]+	-154.060	35.714%		2.100	XTOZTFPYBSIKIB	

Mode MS2 merged

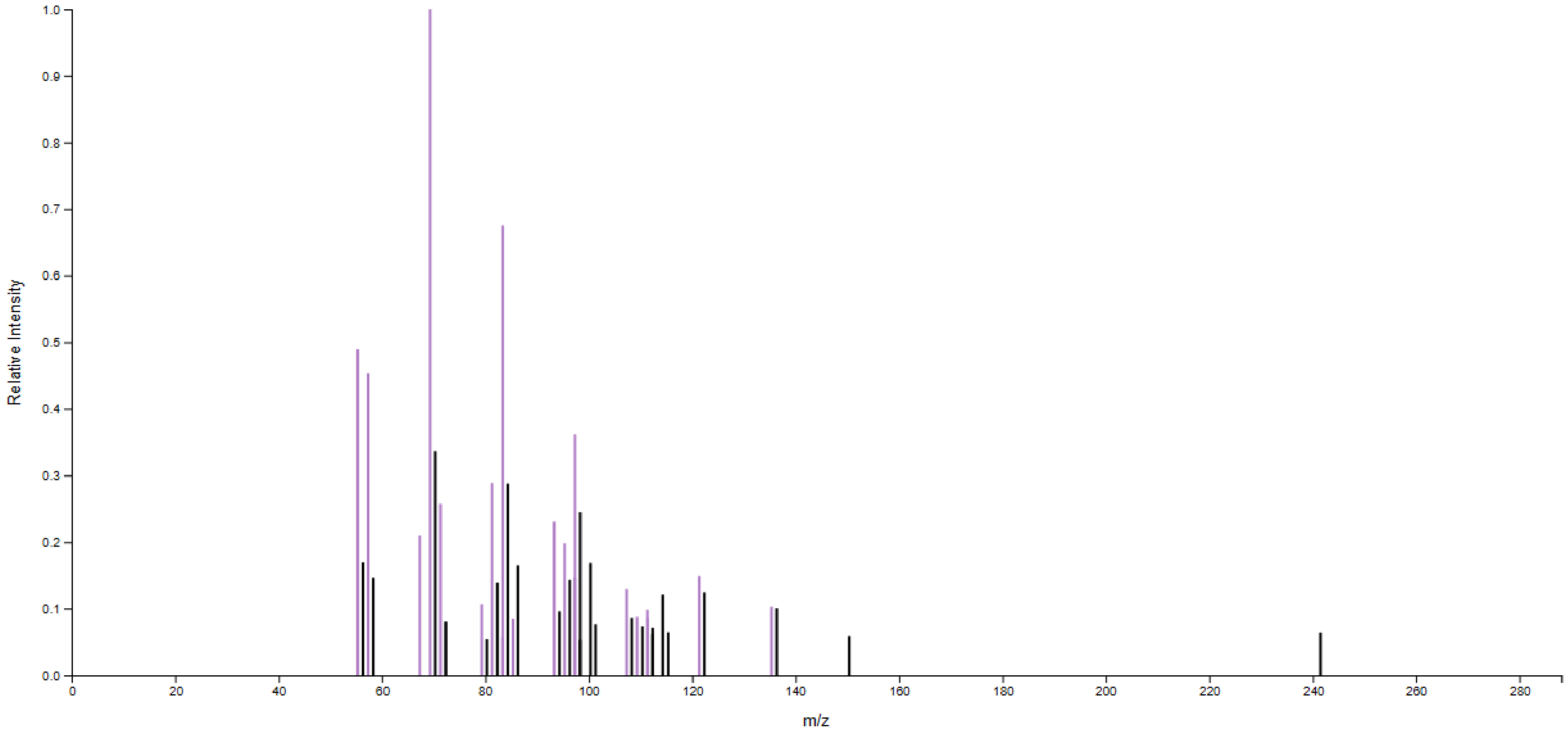

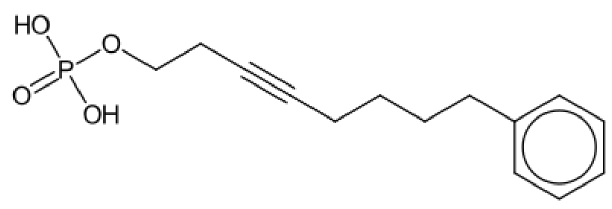

Left click to choose a purple or green peak...

11) 282.10018 – C14H19O4P MSFinder N-(4-ethylphenyl)-5-(2-furanyl)-3-isoxazolecarboxamide

Molecular formula finder

Formula	Error [mDa]	Error [ppm]	Score	Resource	Select
Spectral DB search	0.0000	0.0000	5.0000		☑
C16H14N2O3	-1.2812	-4.5416	4.0820	KNApSAcK,ChEBI,DrugBank,	☑
C11H14N4O5	-5.3040	-18.8021	4.0490	HMDB,ChEBI,FooDB,BMDB,U	☑
C13H18N2O3S	2.0899	7.4083	4.0050	ChEBI,BLEXP,COCONUT,MIN	☑
C10H14N6O4	5.9294	21.0182	3.8930	ChEBI,UNPD,BLEXP,NPA,CO(	☑
C10H18O9	-6.6414	-23.5431	3.8810	HMDB,KNApSAcK,ChEBI,Dru	☑
C15H14N4O2	9.9521	35.2772	3.7950	HMDB,ChEBI,DrugBank,SMP	☐
C14H18O6	8.6147	30.5366	3.7380	HMDB,KNApSAcK,ChEBI,UN	☐
C21H14O	2.7415	9.7180	3.7170	ChEBI,BLEXP,COCONUT,MIN	☐
C12H18N4S2	-4.4350	-15.7215	3.6030	DrugBank,BLEXP,COCONUT	☐
C15H14N4S	-7.8061	-27.6720	3.4580	ChEBI,DrugBank,STOFF,COC	☐
C9H19N2O6P	-3.6506	-12.9409	3.3780	MINE	☐
C18H18OS	6.1126	21.6676	3.3660	BLEXP	☐
C14H18O4S	-9.1435	-32.4131	3.3430	NPA,COCONUT	☐

Structure finder

Name	Score (max=10)	Ontology	InChIKey
CNP0440046	7.01	Phenylpyrazoles	OAEJJGNHVXYALK-
N-(4-ethylphenyl)-5-(2-furanyl)-3-isoxazolecarboxamide	6.89	Aromatic anilides	UCQATMAWCAUGC
N-(3,4-dimethylphenyl)-5-(2-furanyl)-3-isoxazolecarboxamide	6.89	Aromatic anilides	VETPWJIYZLGEPA-U
2-(2-furanyl)-3,5-dimethyl-4-oxido-6-phenylpyrazin-1-ium 1-oxide	6.75	Benzene and substi	TXQSLPNOAMHPEH
8-methyl-4-phenyl-4,8-diazatricyclo[5.2.2.0?,?]undec-10-ene-3,5,9-t	6.72	Phenylpyrrolidines	CKMSWRIKDHFUHI
CNP0450190	6.69	Hydroxyquinolones	YWTWNRLFLRRQDI
3-hydroxy-3-[2-(hydroxyimino)-2-phenylethyl]-2,3-dihydro-1H-indc	6.62	Indolines	IWJVRVUHUSZENV
1-[2-(1H-indol-3-yl)ethyl]-6-oxo-1,6-dihydropyridine-3-carboxylic a	6.57	3-alkylindoles	RFOCXDBKBAMNM
1-(3,4-dihydroxyphenyl)-2-(2-methyl-1-benzimidazolyl)ethanone	6.54	Alkyl-phenylketone	BSXRJIBWLOPQGM
3-[2-(4-aminophenyl)-2-oxoethyl]-3-hydroxy-2,3-dihydro-1H-indol-	6.53	Alkyl-phenylketone	KFRYAJUTOBAFDT-I
1-(2,4-dihydroxyphenyl)-2-(1-methyl-1H-1,3-benzodiazol-2-yl)ethar	6.52	Alkyl-phenylketone	TYWYQQIZHCOYAT
3-(2-oxo-1,3-benzoxazol-3-yl)-N-phenylpropanamide	6.52	Benzoxazolones	IUGHQEKVXISSCI-U
7-methoxy-N-[(pyridin-3-yl)methyl]-1-benzofuran-2-carboxamide	6.49	Benzofurans	AXTGTWYRQIFRBS-
7-methoxy-N-[(pyridin-2-yl)methyl]-1-benzofuran-2-carboxamide	6.49	Benzofurans	HGCWOJVDJNJVDK

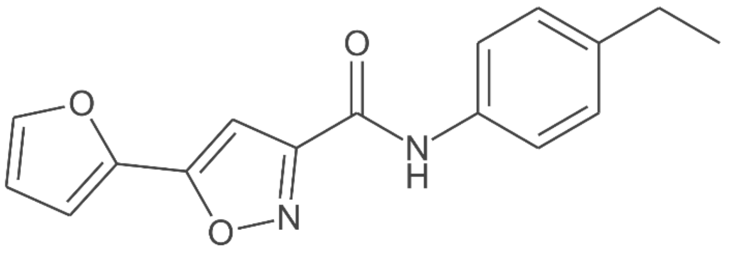

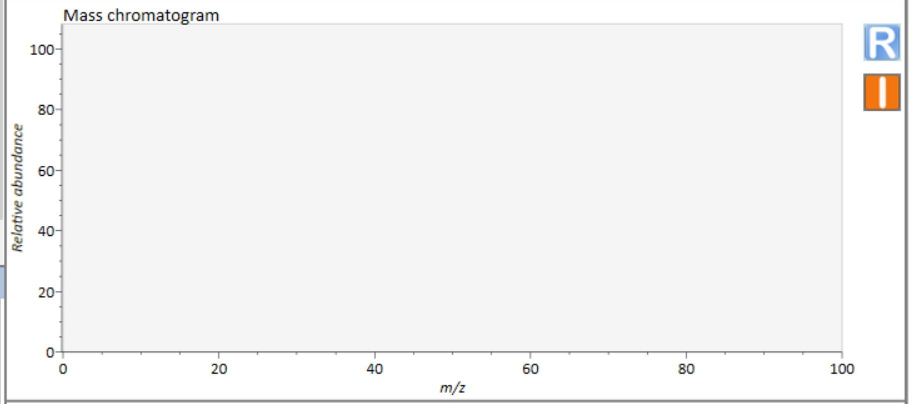

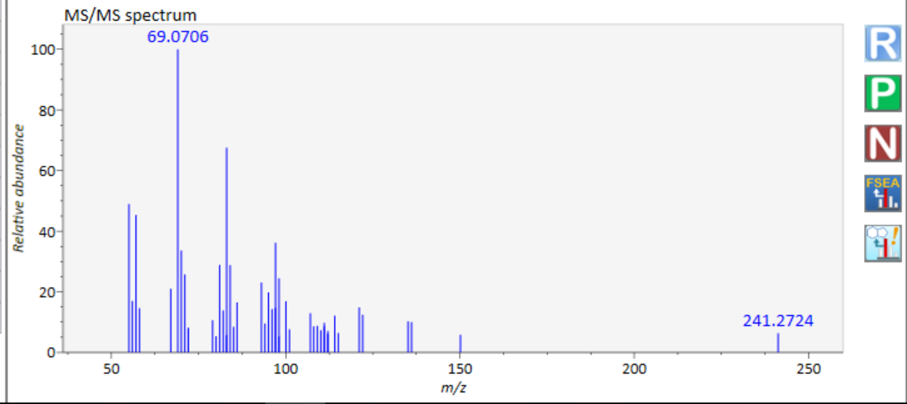

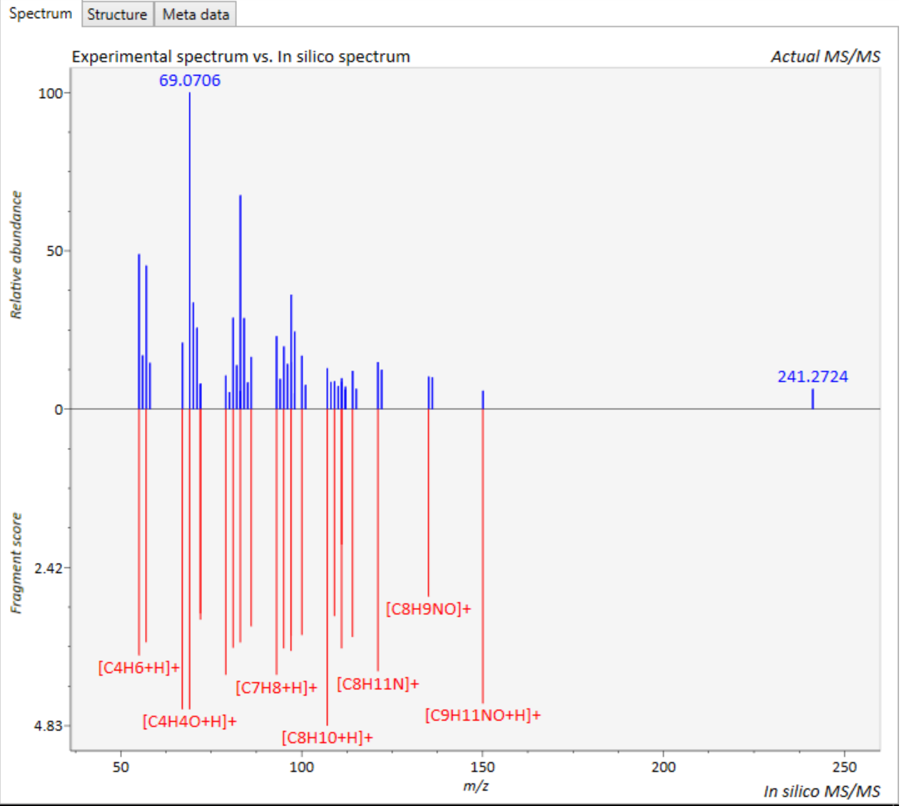

12) 285.13511 – C17H19NO3 MassFrontier Piperine

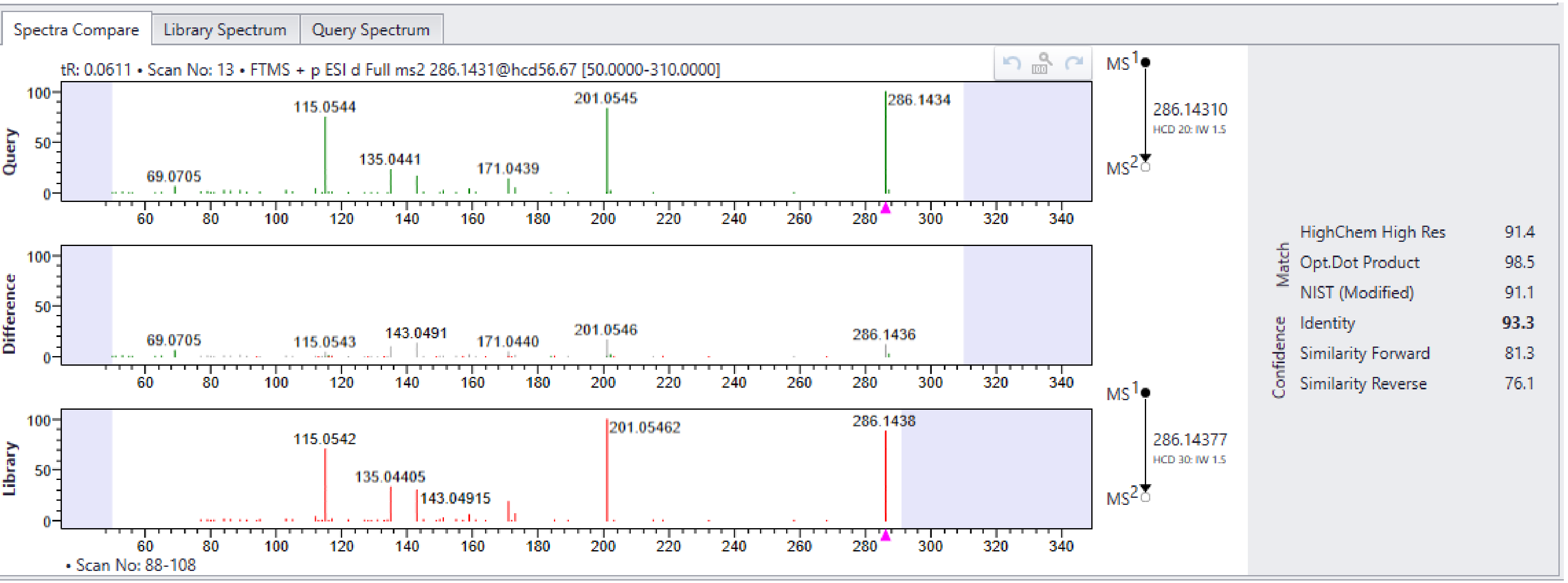

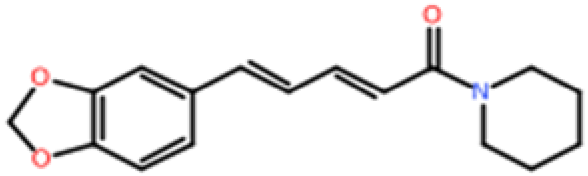

12) 285.13511 – C17H19NO3 MetFrag Piperine

Fragments View

Select area to zoom in. Double click to return.
Click on apex of explained peak to select fragment.

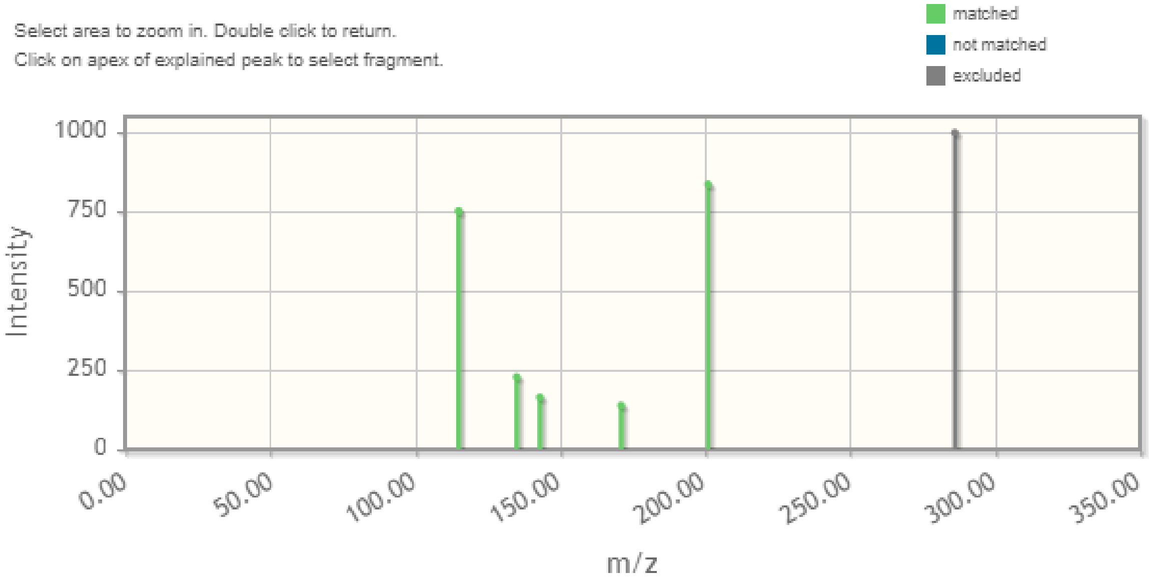

Fragments

Fragment 1

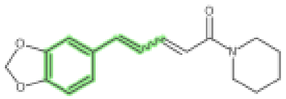

Peak m/z: 115.054443359375

Fragment Mass: 115.05426 Da

Fragment Formula: $[C_9H_6]+H^+$

**Identifier**	C03882
**SMILES**	C1CCN(CC1)C(C(=C(C([H])=C([H])C=2C=CC3=C(C2)OCO3)[H])[H])=O
**Monoisot**	285.13657
**InChI**	InChI=1S/C17H19NO3/c19-17(18-10-4-1-5-11-18)7-3-2-6-14-8-9-15-16(12-14)21-13-20-15/h2-3,6-9,12H,1,4-5,10-11,13H2/b6-2+,7-3+
**MetFrag**	231.12286390846984
**Compoun**	Piperine
**Molecular**	C17H19NO3
**Identifier**	C03882

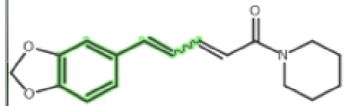

Fragment 1

**Formula**	[C9H6]+H+
**Mass**	115.05426
**Peak m/z**	115.054443359375

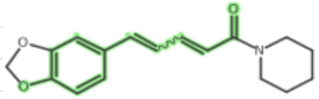

Fragment 4

**Formula**	[C11H7O2]+
**Mass**	171.04408
**Peak m/z**	171.0439453125

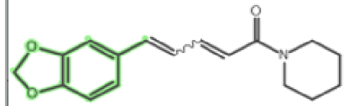

Fragment 2

**Formula**	[C8H6O2]+H+
**Mass**	135.04408
**Peak m/z**	135.044097900391

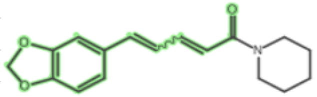

Fragment 5

**Formula**	[C12H9O3]+
**Mass**	201.05465
**Peak m/z**	201.054504394531

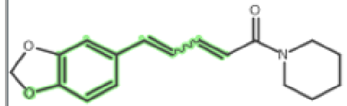

Fragment 3

**Formula**	[C10H7O]+
**Mass**	143.04917
**Peak m/z**	143.049072265625

12) 285.13511 – C17H19NO3 MSFinder Piperine

Formula	Error [mDa]	Error [ppm]	Score	Resource	Select
Spectral DB search	0.0000	0.0000	5.0000		☑
C17H19NO3	-0.0001	-0.0004	4.7670	HMDB,KNApSAcK,ChEBI,Dru	☑
C12H19N3O5	-4.0228	-14.1085	4.4270	HMDB,ChEBI,FooDB,STOFF,E	☑
C14H23NO3S	3.3710	11.8223	4.3080	COCONUT,MINE	☑
C10H24NO6P	-2.3694	-8.3098	4.1110	ChEBI	☑
C16H19N3S	-6.5249	-22.8840	3.8830	HMDB,ChEBI,DrugBank,STO	☑
C19H15N3	-9.8961	-34.7077	3.6870	KNApSAcK,ChEBI,UNPD,NPA	☐
C9H23N3O5S	-0.6517	-2.2856	3.6130		☐
C11H20N5O2P	-1.0320	-3.6193	3.6100		☐
C11H19N5O4	7.2105	25.2872	3.5860	MINE	☐
C10H23NO8	5.8731	20.5971	3.4570		☐
C8H24N5O2PS	2.3391	8.2034	3.4350		☐
C11H27NO3S2	6.7422	23.6450	3.4120		☐
C6H19N7O6	3.1878	11.1798	3.4120		☐

Name	Score (max=10)	Ontology	InChIKey
Piperine	8.34	Alkaloids and derivatives	MXXWOMGUGJBKIW-YPCIICBESA-N
Morphine	8.22	Morphinans	BQJCRHHNABKAKU-KBQPJGBKSA-N
Hydromorphone	8.00	Morphinans	WVLOADHCBXTIJK-YNHQPCIGSA-N
UNPD129642	7.81	Benzodioxoles	WVWHRXVVAYXKDE-RZLXVLJANA-N
Strobolamine	7.68	Dihydropyranones	VMLHZHSLMGIVED-UHFFFAOYNA-N
Erysonine	7.65	Erythrinanes	OOQFZQDSQKMUFW-INQHYVNLNA-
N-[(furan-2-yl)methyl]-4-	7.65	Phenylacetamides	RCPDBFSVEQVRGR-UHFFFAOYSA-N
Erysopine	7.63	Erythrinanes	GNBQGLMYBIWCOO-UHFFFAOYNA-N
UNPD27567	7.63	Dihydropyranones	XHMPBHLVMQWJLB-FOJWECFANA-N
UNPD16985	7.61	Beta amino acids and derivatives	QOUZNKNYXDVAOK-YUTMIAQJNA-N
Jacularine	7.61	Proaporphines	AIXJLWHFXRWKJG-UPOXFFQJNA-N
4-[(morpholin-4-yl)methy	7.60	Coumarins and derivatives	YIQHMWIMSUMGLQ-UHFFFAOYSA-N
1-(6-hydroxy-3,6-dimeth	7.58	Benzene and substituted derivatives	PNBALNBXZGZUSR-UHFFFAOYNA-N
Dihydroisomorphine	7.53	Morphinans	BISKFRUZYNZQKW-AQHSOABDNA-M

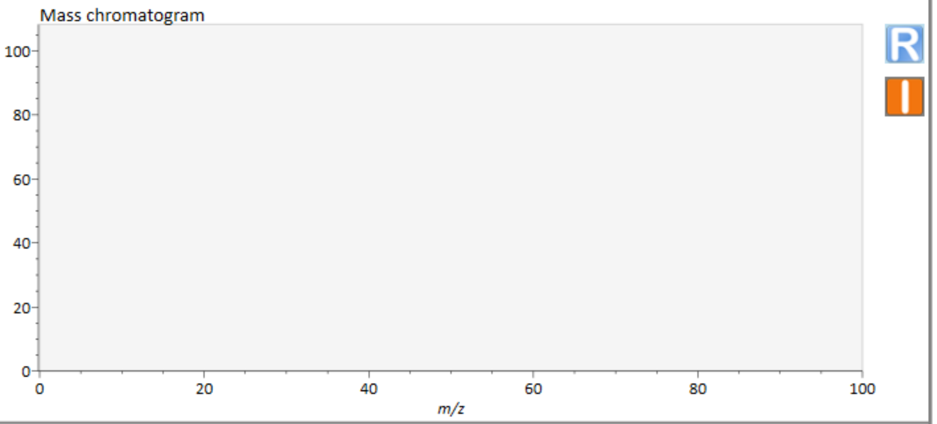

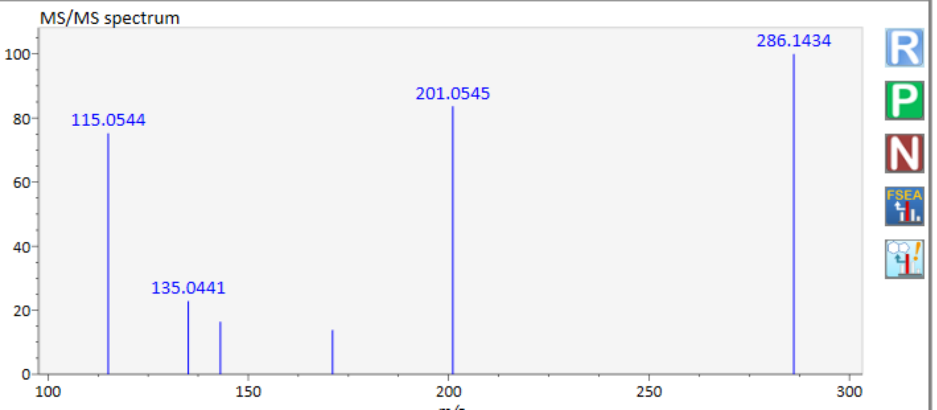

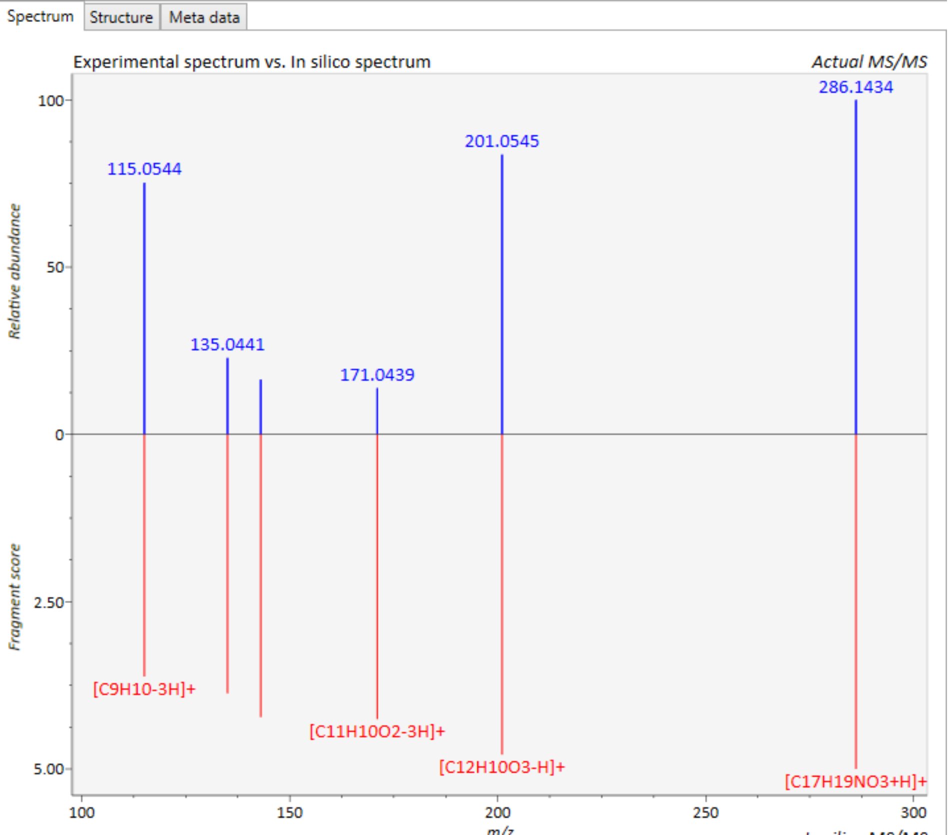

12) 285.13511 – C17H19NO3 Sirius Piperine

Rank	Name	SMILES	Molecular Formula	Adduct	CSI:FingerID Score	Tanimoto Similarity	XLogP	InChIKey	Lipid Class	Database	De Novo
1	Piperin	C1CCN(CC1)C(=O...	C17H19NO3	[M + H]+	-14,870	95,349%	3.3276005	MXXWOMGUGJBK...		■	■

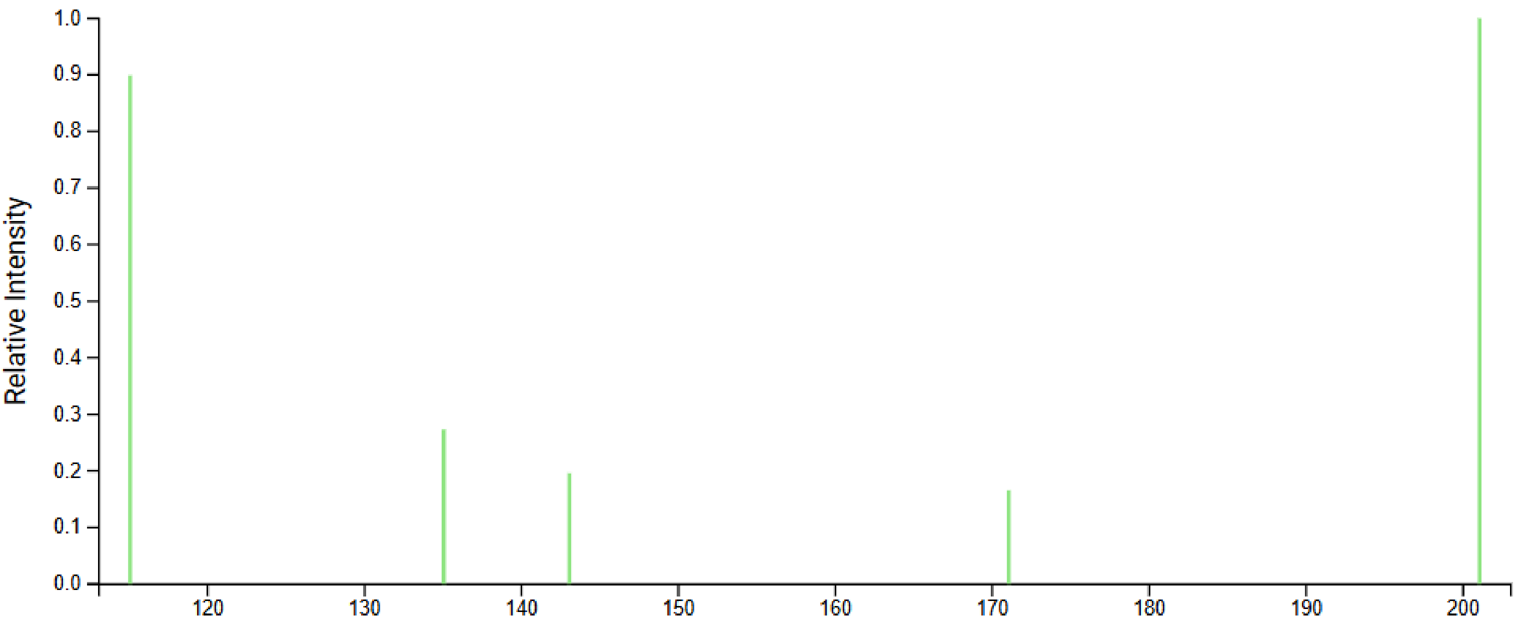

MS2 merged

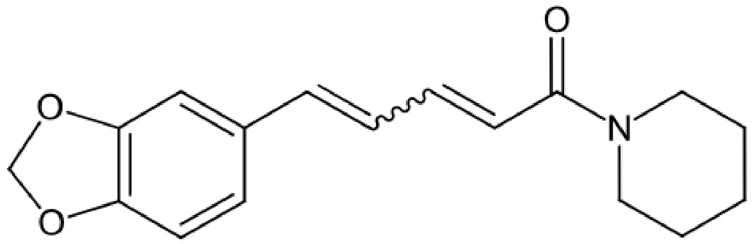

Piperin

13) 295.24900 – C18H33NO2 MetFrag (4E,8E,10E-d18:3)sphingosine

Identifier	LMSP01080013
MetFrag	0.4207450779839173
InChI	InChI=1S/C18H33NO2/c1-2-3-4-5-6-7-8-9-10-11-12-13-14-15-18(21)17(19)16-20/h8-11,14-15,17-18,20-21H,2-7,12-13,16,19H2,1H3/b9-8+,11-10+,15-14+/t17-,18+/m0/s1
LossStat	-1928.852281605282
Monoisoto	295.251
Compoun	(4E,8E,10E-d18:3)sphingosine
Identifier	LMSP01080013
Molecular	C18H33NO2
SMILES	CCCCCCCC=CC=CCCC=CC(C(CO)N)O
SpectralS	2.9161143056417678
PeakStat	-257.9489650749097
ExactSpe	0.0

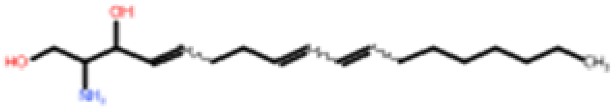

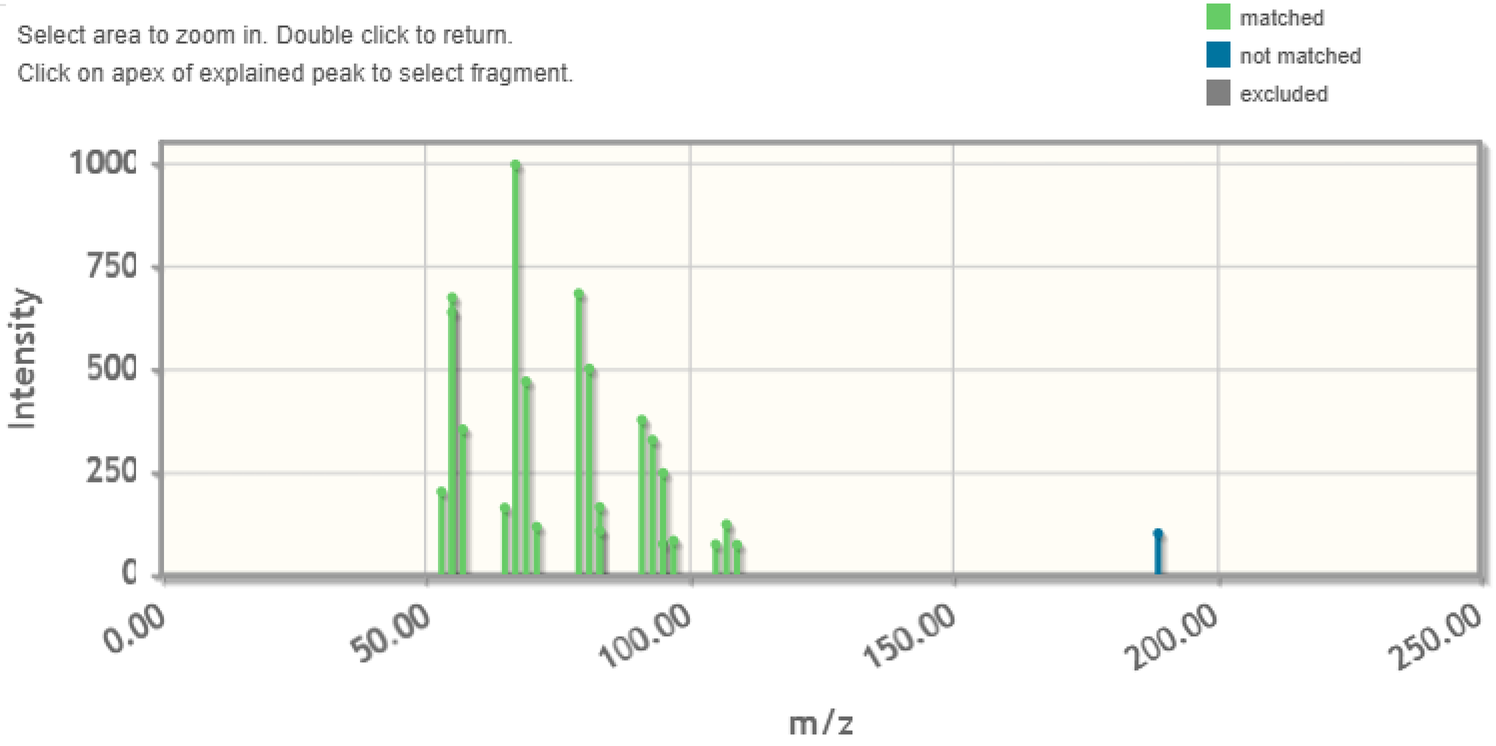

13) 295.24900 – C18H33NO2 MetFrag (4E,8E,10E-d18:3)sphingosine

**Identifier**	LMSP01080013
**MetFrag**	0.4207450779839173
**InChI**	InChI=1S/C18H33NO2/c1-2-3-4-5-6-7-8-9-10-11-12-13-14-15-18(21)17(19)16-20/h8-11,14-15,17-18,20-21H,2-7,12-13,16,19H2,1H3/b9-8+,11-10+,15-14+/t17-,18+/m0/s1
**LossStat**	-1928.852281605282
**Monoisot**	295.251
**Compoun**	(4E,8E,10E-d18:3)sphingosine
**Identifier**	LMSP01080013
**Molecular**	C18H33NO2
**SMILES**	CCCCCCCC=CC=CCCC=CC(C(CO)N)O
**SpectralS**	2.9161143056417678
**PeakStat**	-257.9489650749097
**ExactSpe**	0.0

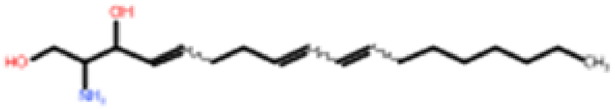

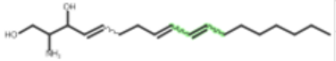

Fragment 1

**Formula**	[C4H5]+
**Mass**	53.0386
**Peak m/z**	53.0393371582031

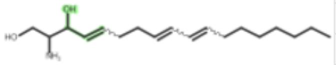

Fragment 2

**Formula**	[C3H4O-H]+
**Mass**	55.01785
**Peak m/z**	55.0185852050781

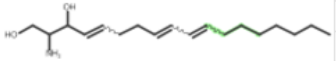

Fragment 3

**Formula**	[C4H7]+
**Mass**	55.05426
**Peak m/z**	55.0549659729004

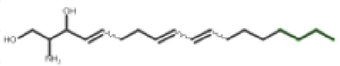

Fragment 4

**Formula**	[C4H9]+
**Mass**	57.06992
**Peak m/z**	57.0706062316895

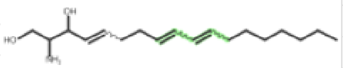

Fragment 5

**Formula**	[C5H6-H]+
**Mass**	65.0386
**Peak m/z**	65.0392761230469

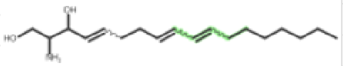

Fragment 6

**Formula**	[C5H7]+
**Mass**	67.05426
**Peak m/z**	67.054931640625

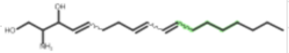

Fragment 7

**Formula**	[C5H9]+
**Mass**	69.06992
**Peak m/z**	69.070556640625

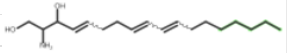

Fragment 8

**Formula**	[C5H11]+
**Mass**	71.08558
**Peak m/z**	71.0861663818359

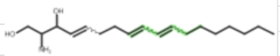

Fragment 9

**Formula**	[C6H8-H]+
**Mass**	79.05426
**Peak m/z**	79.0548400878906

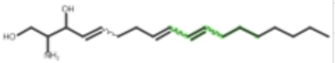

Fragment 10

**Formula**	[C6H9]+
**Mass**	81.06992
**Peak m/z**	81.0705032348633

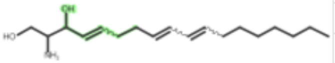

Fragment 11

**Formula**	[C5H8O-H]+
**Mass**	83.04917
**Peak m/z**	83.0497436523438

13) 295.24900 – C18H33NO2 MetFrag (4E,8E,10E-d18:3)sphingosine

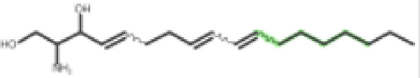

Fragment 12	
Formula	[C6H11]+
Mass	83.08558
Peak m/z	83.0861129760742

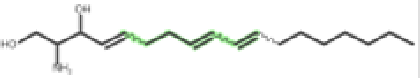

Fragment 13	
Formula	[C7H9-2H]+
Mass	91.05426
Peak m/z	91.0547485351563

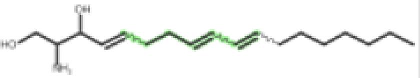

Fragment 14	
Formula	[C7H9]+
Mass	93.06992
Peak m/z	93.0704116821289

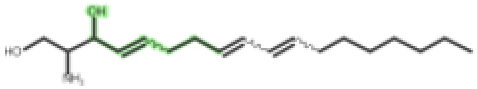

Fragment 15	
Formula	[C6H9O-2H]+
Mass	95.04917
Peak m/z	95.0496520996094

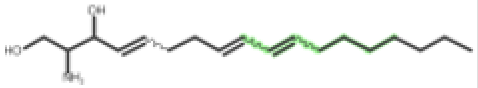

Fragment 16	
Formula	[C7H11]+
Mass	95.08558
Peak m/z	95.0860290527344

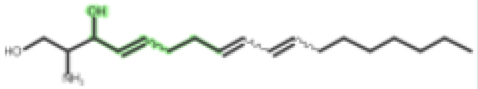

Fragment 17	
Formula	[C6H9O]+
Mass	97.06483
Peak m/z	97.0652770996094

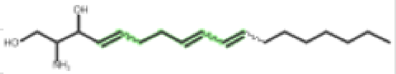

Fragment 18	
Formula	[C8H10-H]+
Mass	105.06992
Peak m/z	105.070281982422

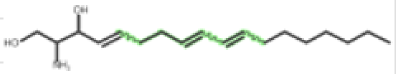

Fragment 19	
Formula	[C8H11]+
Mass	107.08558
Peak m/z	107.085975646973

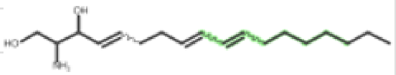

Fragment 20	
Formula	[C8H13]+
Mass	109.10124
Peak m/z	109.101524353027

13) 295.24900 – C18H33NO2 MSFinder (4E,8E,10E-d18:3)sphingosine

Formula	Error [mDa]	Error [ppm]	Score	Resource	Select
Spectral DB search	0.0000	0.0000	5.0000		☑
C18H33NO2	0.4057	1.3741	4.0350	KNApSAcK,LipidMAPS,UNPD	☑
C15H37NO2S	3.7769	12.7920	3.1480		☑
C11H33N7S	1.0915	3.6968	3.1020		☑
C14H29N7	-2.2796	-7.7210	3.0820		☑
C8H29N11O	4.9310	16.7008	2.9940		☑
C9H29N9O2	-6.3024	-21.3464	2.9460		☐

Name	Score (max=10)	Ontology	InChIKey
Tetrahydrobungeanool	7.03	N-acyl amines	GJDPGFHVEKFXEZ-SQIWNDBBSA-N
(4E,8E,10E-d18:3)sphingos	7.02	1,2-aminoalcohols	PZVYDNWEQXJZPQ-RRLMLTBBNA-N
1-(piperidin-1-yl)-3-({1,7,7	6.92	Bicyclic monoterpenoids	KEELYYKHJSSOCT-UHFFFAOYSA-N
UNPD219123	6.75	Enones	QCAPORRNRLFTCV-NTCAYCPXSA-N
(octahydro-1H-quinolizin-	6.68	Lupinine-type alkaloids	SRIUOGOZNQFXLD-UHFFFAOYSA-N
(octahydro-1H-quinolizin-	6.66	Lupinine-type alkaloids	BPIODMZQXSFQCA-UHFFFAOYSA-N
3-{5-amino-1-cyclohexyl-	6.61	Oxanes	REBGEWSLWYYXAI-UHFFFAOYSA-N

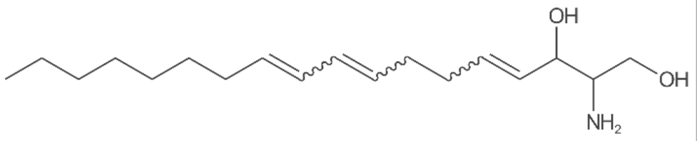

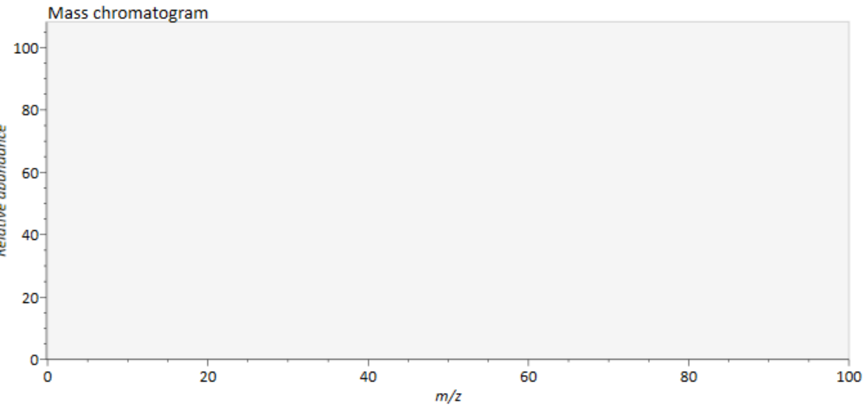

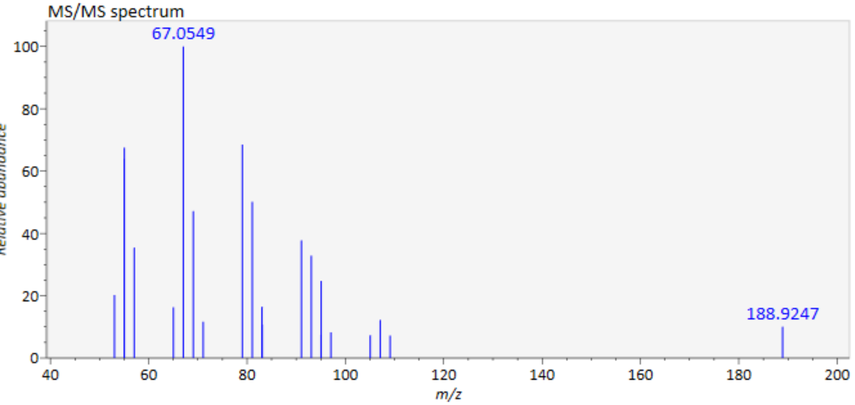

Spectrum Structure Meta data

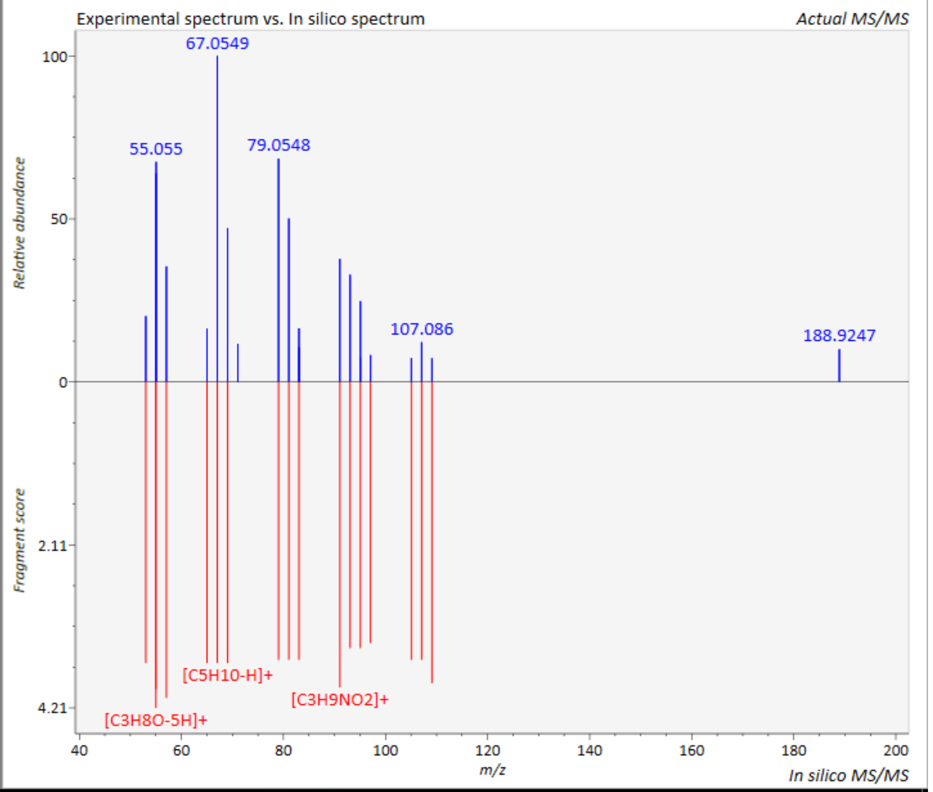

14) 306.23906 – C12H30N6O3 MSFinder 4,5,13-Duratriene-1,3-diol;2,7,11-Cembratrien-4,6-diol

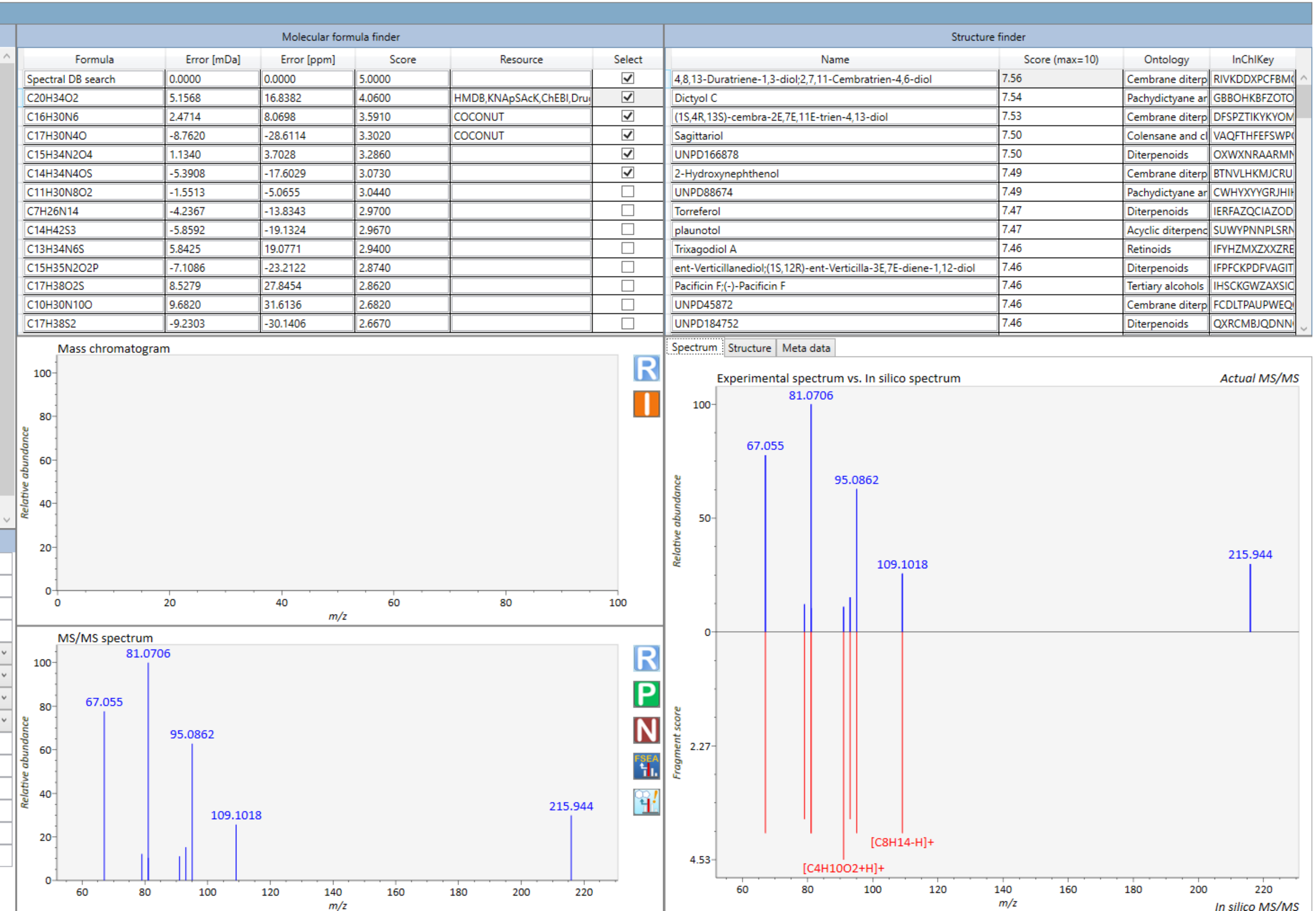

Molecular formula finder

Formula	Error [mDa]	Error [ppm]	Score	Resource	Select
Spectral DB search	0.0000	0.0000	5.0000		☑
C20H34O2	5.1568	16.8382	4.0600	HMDB,KNApSAcK,ChEBI,Dru	☑
C16H30N6	2.4714	8.0698	3.5910	COCONUT	☑
C17H30N4O	-8.7620	-28.6114	3.3020	COCONUT	☑
C15H34N2O4	1.1340	3.7028	3.2860		☑
C14H34N4OS	-5.3908	-17.6029	3.0730		☑
C11H30N8O2	-1.5513	-5.0655	3.0440		☐
C7H26N14	-4.2367	-13.8343	2.9700		☐
C14H42S3	-5.8592	-19.1324	2.9670		☐
C13H34N6S	5.8425	19.0771	2.9400		☐
C15H35N2O2P	-7.1086	-23.2122	2.8740		☐
C17H38O2S	8.5279	27.8454	2.8620		☐
C10H30N10O	9.6820	31.6136	2.6820		☐
C17H38S2	-9.2303	-30.1406	2.6670		☐

Structure finder

Name	Score (max=10)	Ontology	InChIKey
4,8,13-Duratriene-1,3-diol;2,7,11-Cembratrien-4,6-diol	7.56	Cembrane diterp	RIVKDDXPCFBM(
Dictyol C	7.54	Pachydictyane ar	GBBOHKBFZOTO
(1S,4R,13S)-cembra-2E,7E,11E-trien-4,13-diol	7.53	Cembrane diterp	DFSPZTIKYKYOM
Sagittariol	7.50	Colensane and cl	VAQFTHFEFSWP(
UNPD166878	7.50	Diterpenoids	OXWXNRAARMN
2-Hydroxynephthenol	7.49	Cembrane diterp	BTNVLHKMJCRU
UNPD88674	7.49	Pachydictyane ar	CWHYXYYGRJHIH
Torreferol	7.47	Diterpenoids	IERFAZQCIAZOD
plaunotol	7.47	Acyclic diterpenc	SUWYPNNPLSRN
Trixagodiol A	7.46	Retinoids	IFYHZMXZXXZRE
ent-Verticillanediol;(1S,12R)-ent-Verticilla-3E,7E-diene-1,12-diol	7.46	Diterpenoids	IFPFCKPDFVAGIT
Pacificin F;(-)-Pacificin F	7.46	Tertiary alcohols	IHSCKGWZAXSIC
UNPD45872	7.46	Cembrane diterp	FCDLTPAUPWEQ(
UNPD184752	7.46	Diterpenoids	QXRCMBJQDNN(

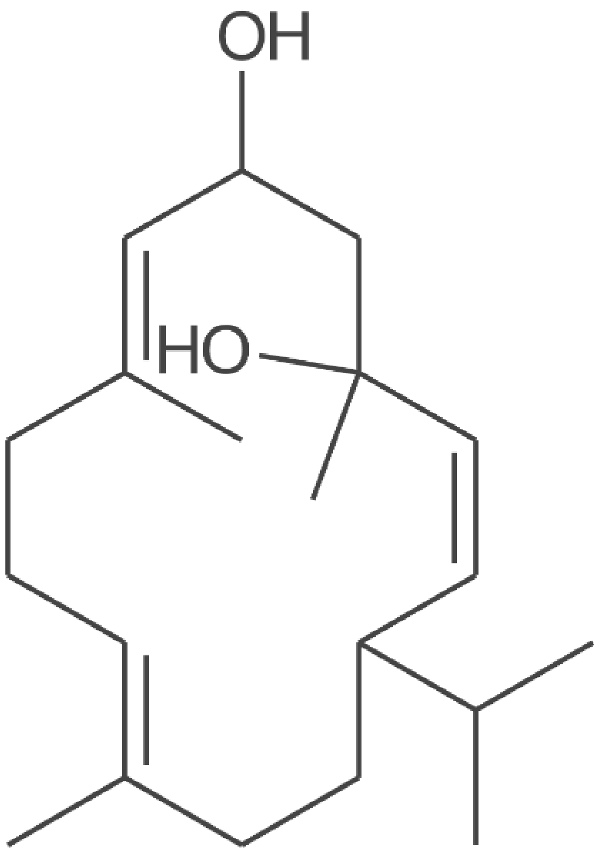

14) 306.23906 – C12H30N6O3 MSFinder Sagittariol

Formula	Error [mDa]	Error [ppm]	Score	Resource	Select
Spectral DB search	0.0000	0.0000	5.0000		☑
C20H34O2	5.1568	16.8382	4.0600	HMDB,KNApSAcK,ChEBI,Dru	☑
C16H30N6	2.4714	8.0698	3.5910	COCONUT	☑
C17H30N4O	-8.7620	-28.6114	3.3020	COCONUT	☑
C15H34N2O4	1.1340	3.7028	3.2860		☑
C14H34N4OS	-5.3908	-17.6029	3.0730		☑
C11H30N8O2	-1.5513	-5.0655	3.0440		☐
C7H26N14	-4.2367	-13.8343	2.9700		☐
C14H42S3	-5.8592	-19.1324	2.9670		☐
C13H34N6S	5.8425	19.0771	2.9400		☐
C15H35N2O2P	-7.1086	-23.2122	2.8740		☐
C17H38O2S	8.5279	27.8454	2.8620		☐
C10H30N10O	9.6820	31.6136	2.6820		☐
C17H38S2	-9.2303	-30.1406	2.6670		☐

Name	Score (max=10)	Ontology	InChIKey
4,8,13-Duratriene-1,3-dic	7.56	Cembrane diterpenoids	RIVKDDXPCFBMOV-RLLOCNTHNA-N
Dictyol C	7.54	Pachydictyane and cneorubin diterpen	GBBOHKBFZOTOSW-ACYKKZAUNA-N
(1S,4R,13S)-cembra-2E,7E	7.53	Cembrane diterpenoids	DFSPZTIKYKYOMU-RWGMZQPBSA-N
Sagittariol	7.50	Colensane and clerodane diterpenoids	VAQFTHFEFSWPGQ-UHFFFAOYNA-N
UNPD166878	7.50	Diterpenoids	OXWXNRAARMNGON-ATWBKXACNA
2-Hydroxynephthenol	7.49	Cembrane diterpenoids	BTNVLHKMJCRURY-ODNNCTDHNA-N
UNPD88674	7.49	Pachydictyane and cneorubin diterpen	CWHYXYYGRJHIHO-ACYKKZAUNA-N
Torreferol	7.47	Diterpenoids	IERFAZQCIAZODG-HGLJTRSHNA-N
plaunotol	7.47	Acyclic diterpenoids	SUWYPNNPLSRNPS-UNTSEYQFSA-N
Trixagodiol A	7.46	Retinoids	IFYHZMXZXXZREG-ODJYXWRFNA-N
ent-Verticillanediol;(1S,12	7.46	Diterpenoids	IFPFCKPDFVAGIT-HJACEESMNA-N
Pacificin F;(-)-Pacificin F	7.46	Tertiary alcohols	IHSCKGWZAXSIOS-WFKWQINVNA-N
UNPD45872	7.46	Cembrane diterpenoids	FCDLTPAUPWEQQW-FPOJDFOKNA-N
UNPD184752	7.46	Diterpenoids	QXRCMBJQDNNQTD-NFWSLAGUNA-I

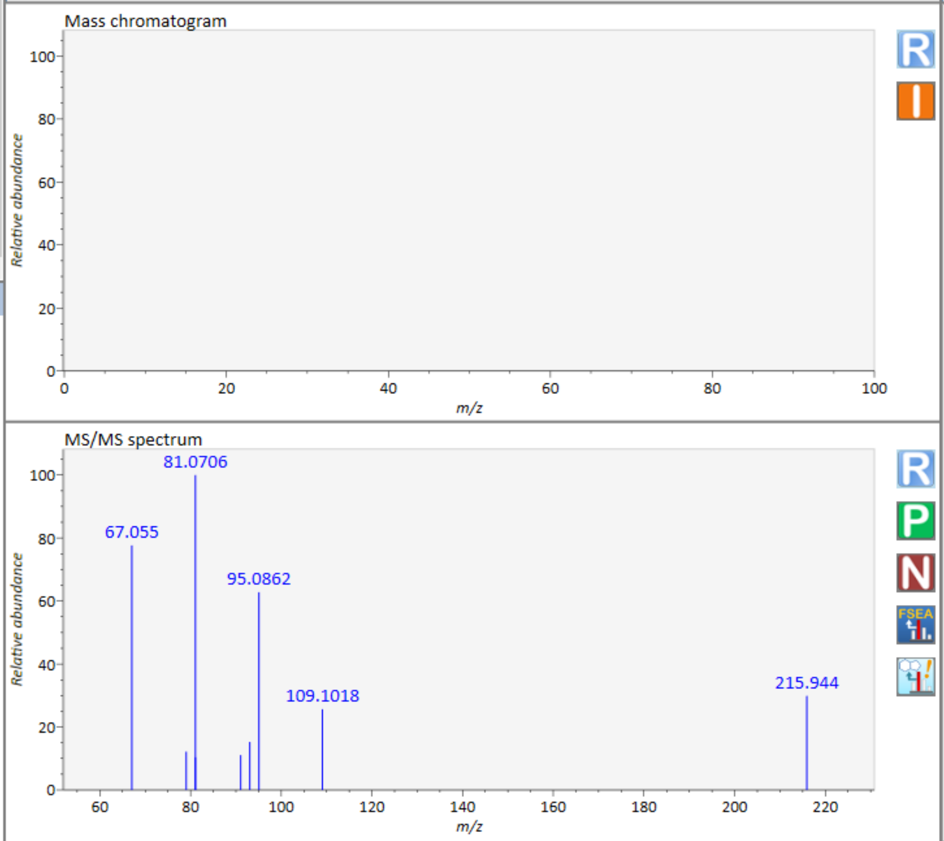

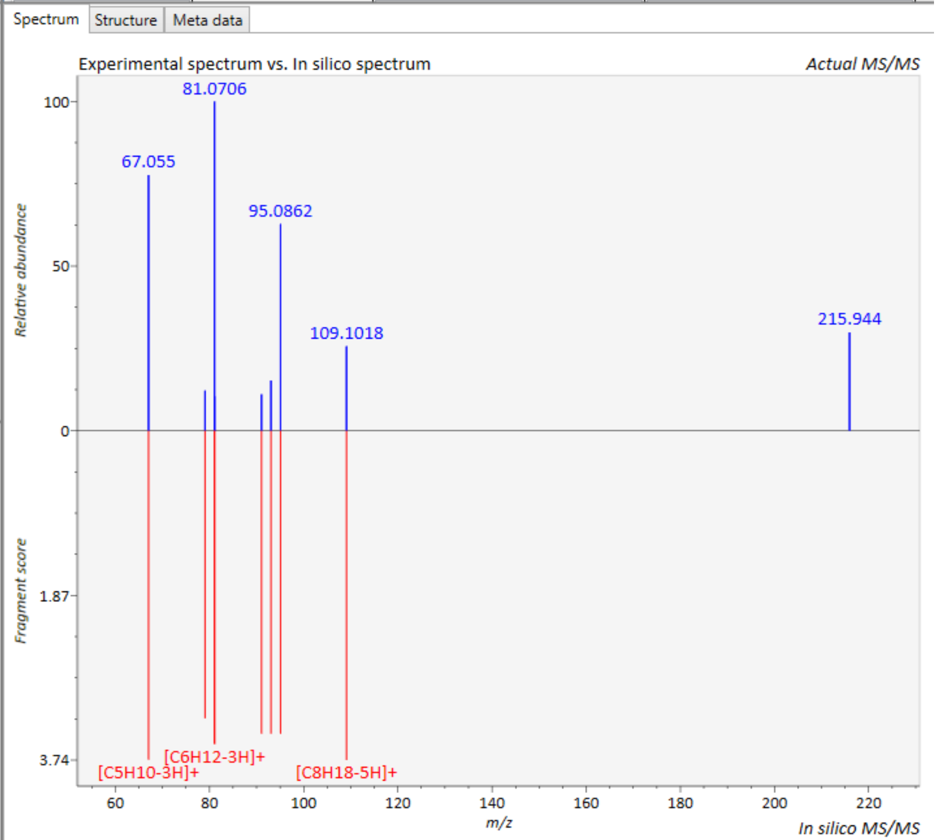

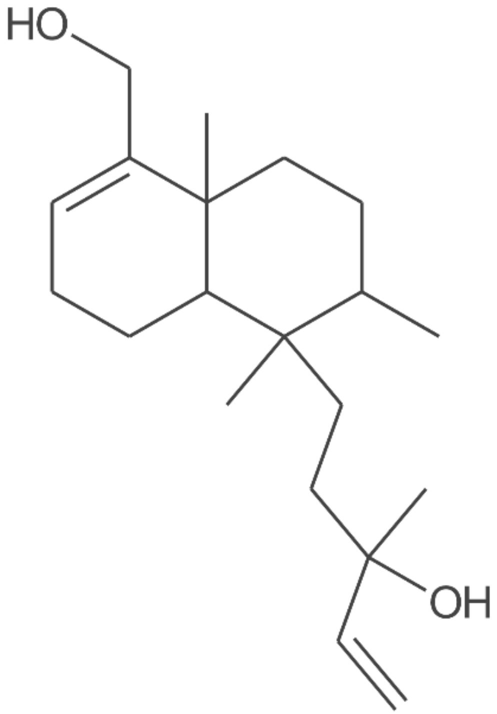

15) 319.24639 – C20H33NO2 MassFrontier 17beta-Hydroxy-17-methyl-5alpha-androstan-3-one oxime

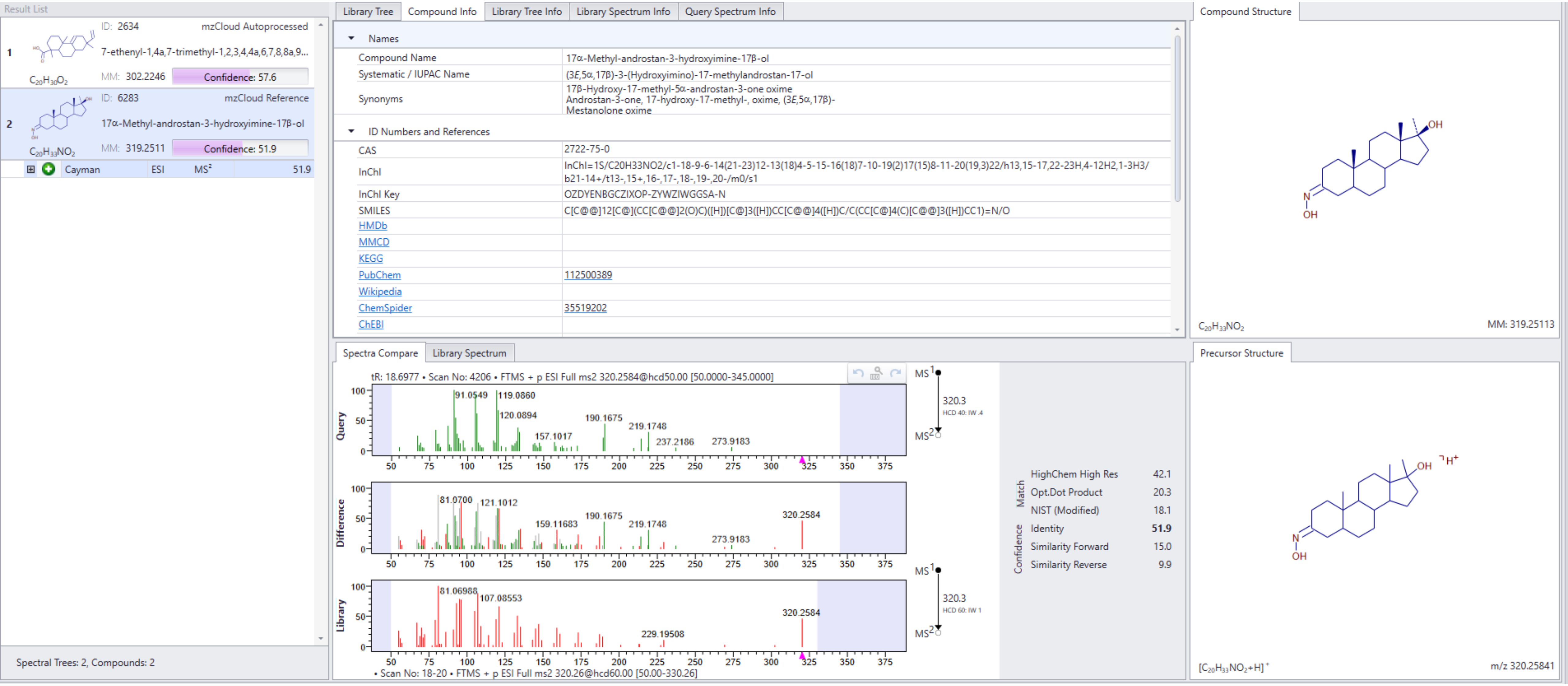

15) 319.24639 – C20H33NO2 MetFrag Anandamine (18:4, n-3)

Identifier	LMFA08040006
MetFrag	0.6206321399956283
InChI	InChI=1S/C20H33NO2/c1-2-3-4-5-6-7-8-9-10-11-12-13-14-15-16-17-20(23)21-18-19-22/h3-4,6-7,9-10,12-13,22H,2,5,8,11,14-19H2,1H3,(H,21,23)/b4-3-,7-6-,10-9-,13-12-
LossStat§	-958.350151112366
Monoisot(	319.251
Compoun	Anandamide (18:4, n-3)
Identifier	LMFA08040006
Molecular	C20H33NO2
SMILES	CCC=CCC=CCC=CCC=CCCCCC(=O)NCCO
SpectralS	3.1982876989592004
PeakStat§	-141.44196290310026
ExactSpe	0.0

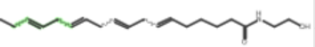

Fragment 1

Formula	[C5H7]+
Mass	67.05426
Peak m/z	67.0549621582031

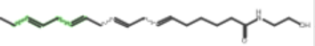

Fragment 2

Formula	[C6H8-H]+
Mass	79.05426
Peak m/z	79.0549774169922

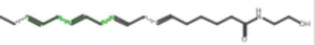

Fragment 3

Formula	[C7H9-2H]+
Mass	91.05426
Peak m/z	91.0548858642578

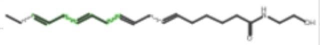

Fragment 4

Formula	[C7H9]+
Mass	93.06992
Peak m/z	93.0705490112305

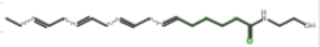

Fragment 5

Formula	[C6H9O]+
Mass	97.06483
Peak m/z	97.0654907226563

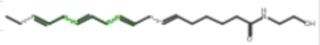

Fragment 6

Formula	[C8H10-H]+
Mass	105.06992
Peak m/z	105.070457458496

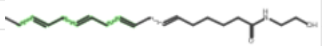

Fragment 7

Formula	[C9H12-H]+
Mass	119.08558
Peak m/z	119.08602142334

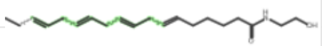

Fragment 8

Formula	[C10H13]+
Mass	133.10124
Peak m/z	133.101547241211

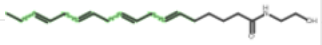

Fragment 9

Formula	[C14H21]+
Mass	189.16388
Peak m/z	189.164245605469

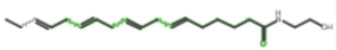

Fragment 10

Formula	[C15H21O+H]+H+
Mass	219.17445
Peak m/z	219.174758911133

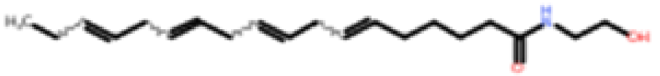

15) 319.24639 – C20H33NO2 MSFinder Anandamine (18:4, n-3)

Molecular formula finder

Formula	Error [mDa]	Error [ppm]	Score	Resource	Select
Spectral DB search	0.0000	0.0000	5.0000		☑
C20H33NO2	0.4057	1.2708	4.2800	STOFF,LipidMAPS,UNPD,BLE)	☑
C15H33N3O4	-3.6170	-11.3298	3.4370		☑
C17H37NO2S	3.7769	11.8304	3.3810		☑
C13H33N7S	1.0915	3.4189	3.2440		☑
C10H29N11O	4.9310	15.4453	3.2360		☑
C16H29N7	-2.2796	-7.1405	3.2240		☐
C14H33N5O3	7.6164	23.8565	3.2230		☐
C11H29N9O2	-6.3024	-19.7416	3.2200		☐
C11H37N5OS2	-6.7707	-21.2085	3.1450		☐
C10H37N7S2	4.4626	13.9782	3.1300		☐

Structure finder

Name	Score (max=10)	Ontology	InChIKey
CNP0435668	7.19	Tertiary alcohols	JQNGQSQFBO\
11-hydroxy-N-(2-methylpropyl)hexadeca-2,4,7,9-tetraenamide	7.14	Secondary alcoh	GBIGUUMWQWI
UNPD188713	7.12	Quinolizines	QFCXPIZNNLLXT
Anandamide (18:4, n-3)	7.11	N-acylethanolam	BSEHZAIYCIVZNE
4-(2-amino-3-hydroxy-8,10-dimethyldodec-6-en-1-yl)phenol	7.07	Amphetamines a	HBGMCLLAYAFYI
CNP0444914	7.05	Amphetamines a	STSAUBXDRWXC
CNP0445985	7.02	Acetamides	UQAALXHUTYUL
N-hydroxy arachidonoyl amine	7.01	Hydroxamic acid	DWUNPFBWVHL
3-(diethylamino)propyl 2-phenylheptanoate	6.92	Fatty acid esters	LLFBJCUVGCPAR
N-[7-hydroxy-9a,11a-dimethyl-hexadecahydro-1H-cyclopenta[a]phe	6.91	Androgens and c	YFIVNBTYXVUCH
2,2,5,9-tetramethyldeca-3,4,8-trien-1-yl 2-(pyrrolidin-1-yl)acetate	6.75	Alpha amino acic	WNBOVRCECFAZ
[(furan-2-yl)methylidene]({2-[4-(3-methylbutyl)-2-(propan-2-yl)oxan	6.75	Oxanes	FKQSUVMEINHM
10-(4-hydroxybutyl)-4,11-dimethyl-11-[2-(methylamino)ethyl]tricycl	6.73	Sesquiterpenoids	CVCNGIHSRMUF
N-dodecyl-4-methoxybenzamide	6.46	Benzamides	KVEZPQNBMSQ!

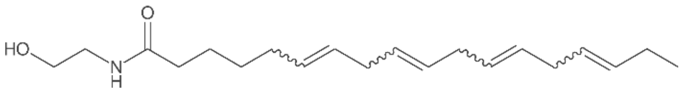

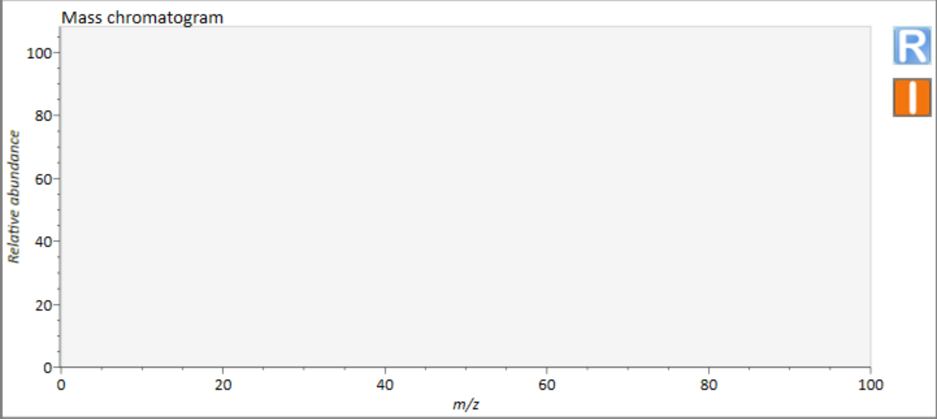

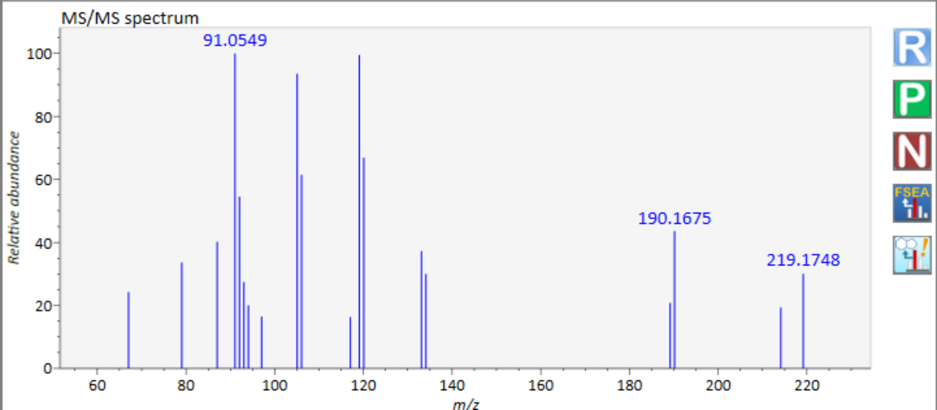

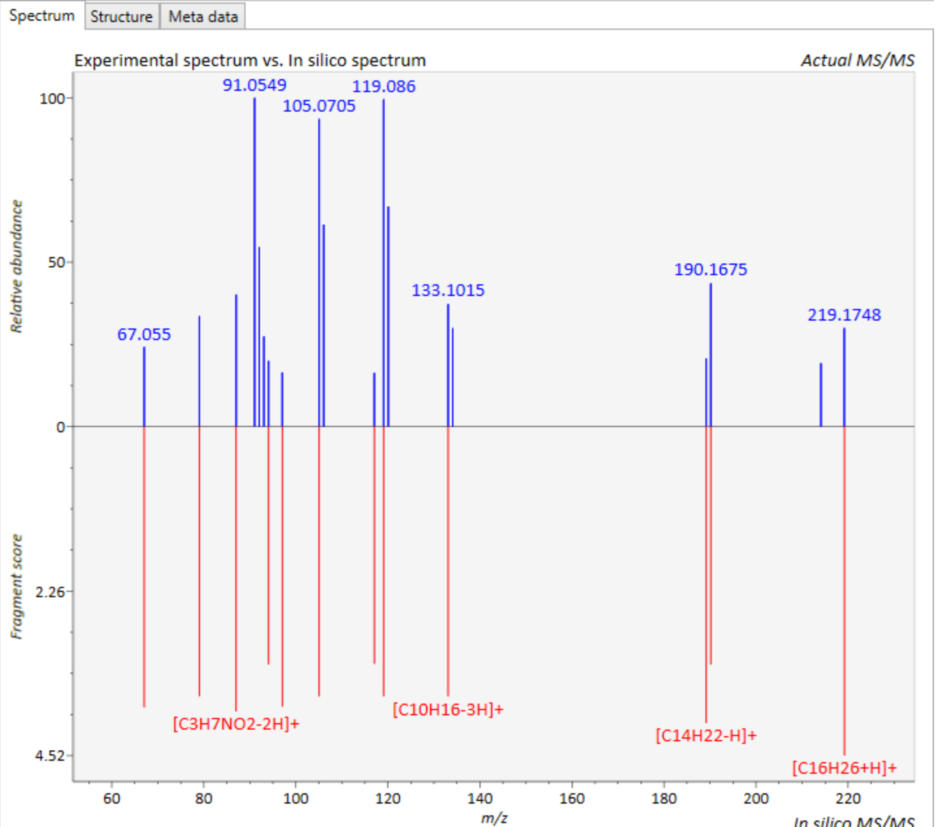

15) 319.24639 – C20H33NO2 Sirius Anandamine (18:4, n-3)

(6Z,9Z,12Z,15E)-N-...	CCC=CCC=CCC=...	C20H33NO2	[M + H]+	-308,640	33,813%	4.3	BSEHZAIYCIVZNE	■

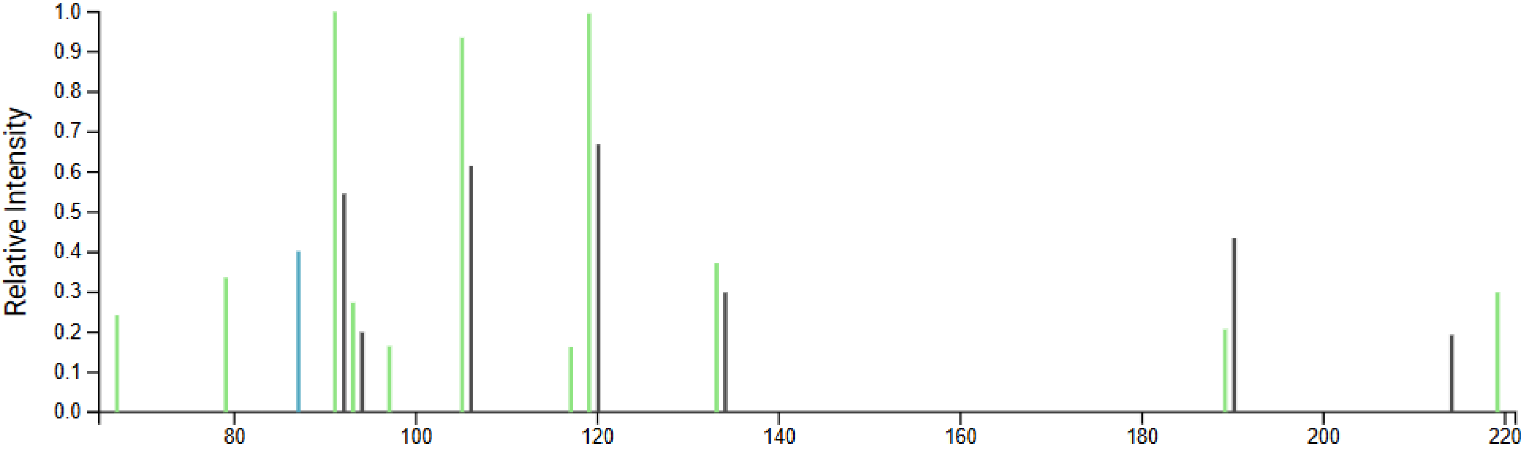

MS2 merged

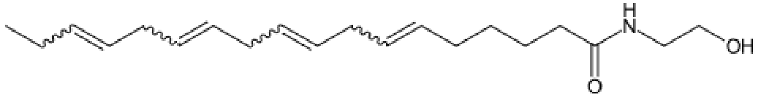

(6Z,9Z,12Z,15E)-N-(2-hydroxyethyl)oct...

16) 326.32814 – C20H42N2O MetFrag (7R)-18-(2-ethylhydrazino)octadic-9-en-7-ol

1	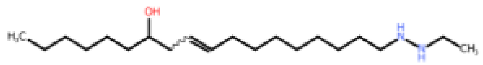 (7R)-18-(2-ethylhydrazino)octadec-9-en-7-ol	57023760<br>InChIKeyBlock1 = MVIGMQJXLMTVGD	326.33	$C_{20}H_{42}N_2O$	1.0	Peaks: 3 / 29<br>Fragments<br>Scores<br>Download

Select area to zoom in. Double click to return.
Click on apex of explained peak to select fragment.

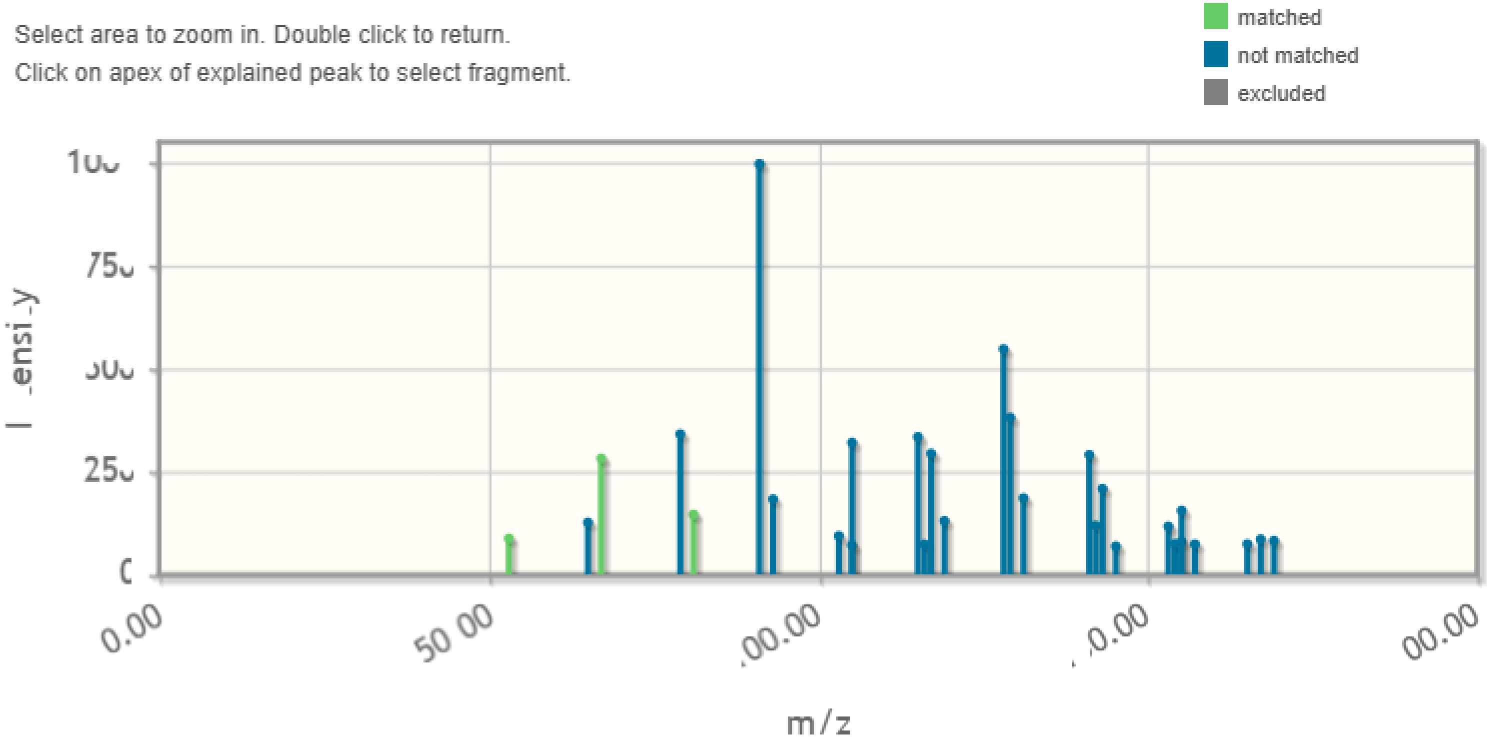

Fragment 1

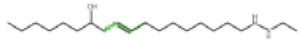

Peak m/z: 53.0393905639648
Fragment Mass: 53.0386 Da
Fragment Formula: $[C_4H_6\text{-}H]^+$

Fragment 2

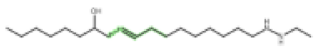

Peak m/z: 67.0550155639648
Fragment Mass: 67.05426 Da
Fragment Formula: $[C_5H_8\text{-}H]^+$

Fragment 3

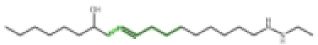

Peak m/z: 81.0706024169922
Fragment Mass: 81.06992 Da
Fragment Formula: $[C_6H_{10}\text{-}H]^+$

16) 326.32814 – C20H42N2O MSFinder Palmitoylputrescine

Formula	Error [mDa]	Error [ppm]	Score	Resource	Select
Spectral DB search	0.0000	0.0000	5.0000		☑
C20H42N2O	-0.0096	-0.0294	3.9770	UNPD,NPA,COCONUT,MINE	☑

Name	Score (max=10)	Ontology	InChIKey
UNPD98355	6.06	N-acyl amines	VWGZHNDNCJLJ

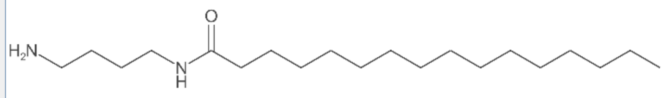

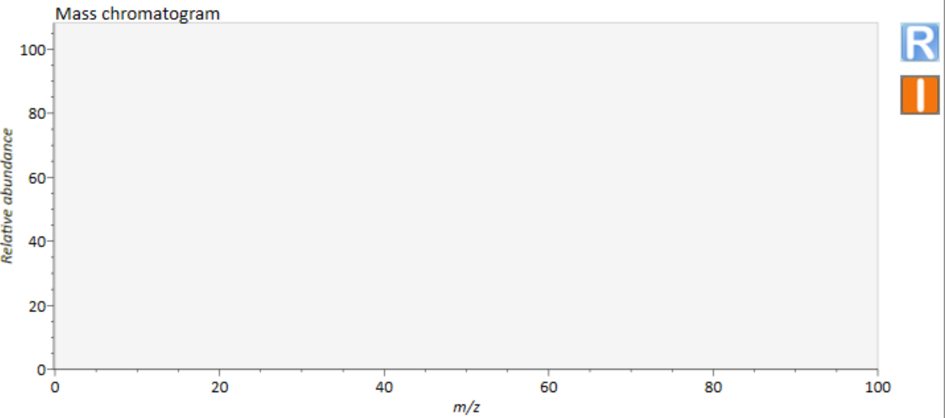

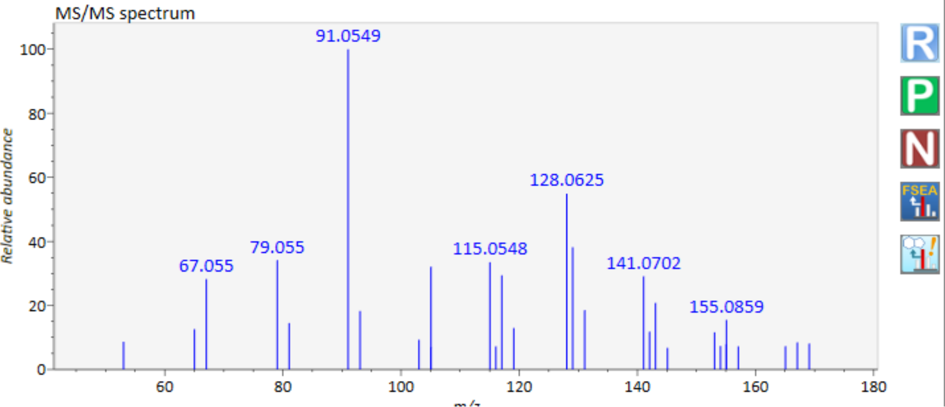

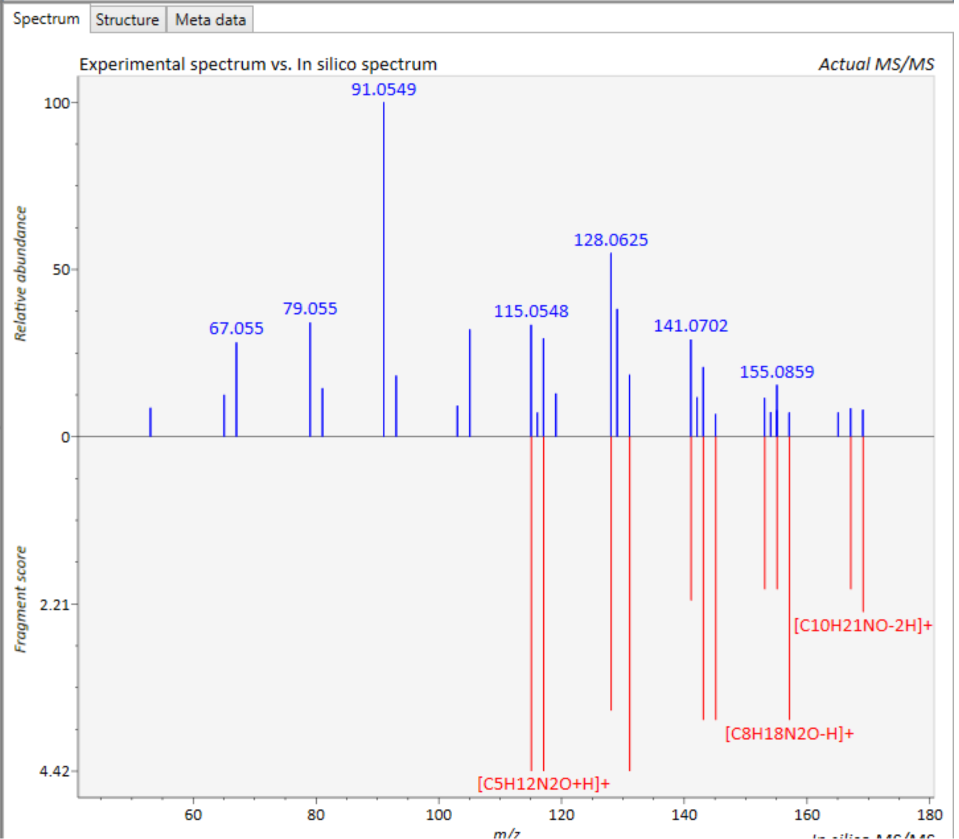

16) 326.32814 – C20H42N2O Sirius Palmitoylputrescine

4	Palmitoylputrescine	CCCCCCCCCCCC...	C20H42N2O	[M + H]+	-256,032	32,558%	6.6	VWGZHNDNCJLJ...		■	

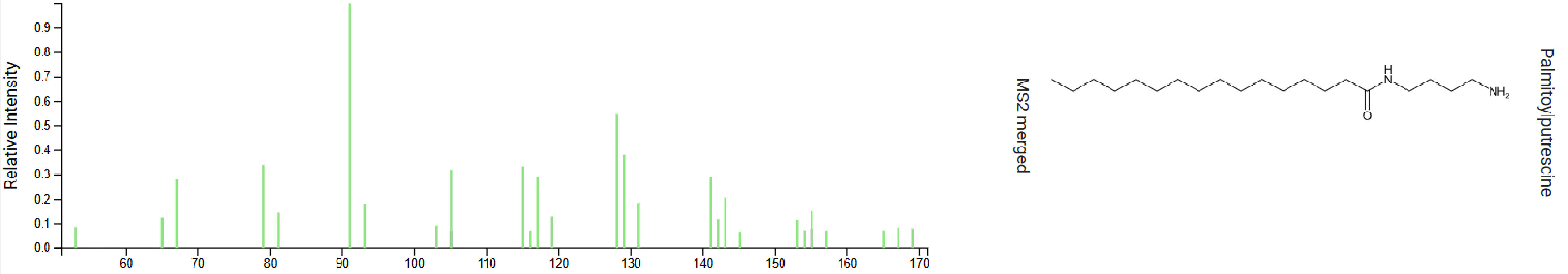

16) 326.32814 – C20H42N2O Sirius 1-Hexadecyl-3-propylurea

Rank	Name	SMILES	Molecular Formula	Adduct	CSI:FingerID Score	Tanimoto Similarity	XLogP	InChIKey	Lipid Class	Database	De Novo
1	1-Hexadecyl-3-pro...	CCCCCCCCCCCC...	C20H42N2O	[M + H]+	-217,575	27,891%	8.0	IJJXDYXCBPUKQZ		■	

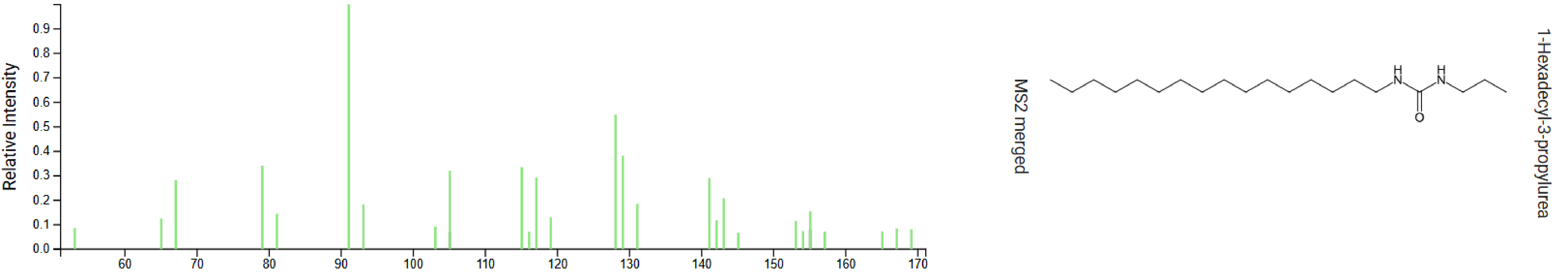

17) 367.41679 – C25H53N MetFrag N,N-dimethyltricosan-1-amine

Identifier	20291939
MetFrag	7.313846910784074
InChI	InChI=1S/C25H53N/c1-4-5-6-7-8-9-10-11-12-13-14-15-16-17-18-19-20-21-22-23-24-25-26(2)3/h4-25H2,1-3H3
LossStat	-198.2524709521697
Monoisoto	367.418
Compoun	N,N-dimethyltricosan-1-amine
Identifier	20291939
Molecular	C25H53N
SMILES	CCCCCCCCCCCCCCCCCCCCCCCN(C)C
SpectralS	0.4283850198238284
PeakStat	-112.32091718321662
ExactSpe	0.0

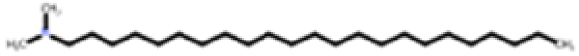

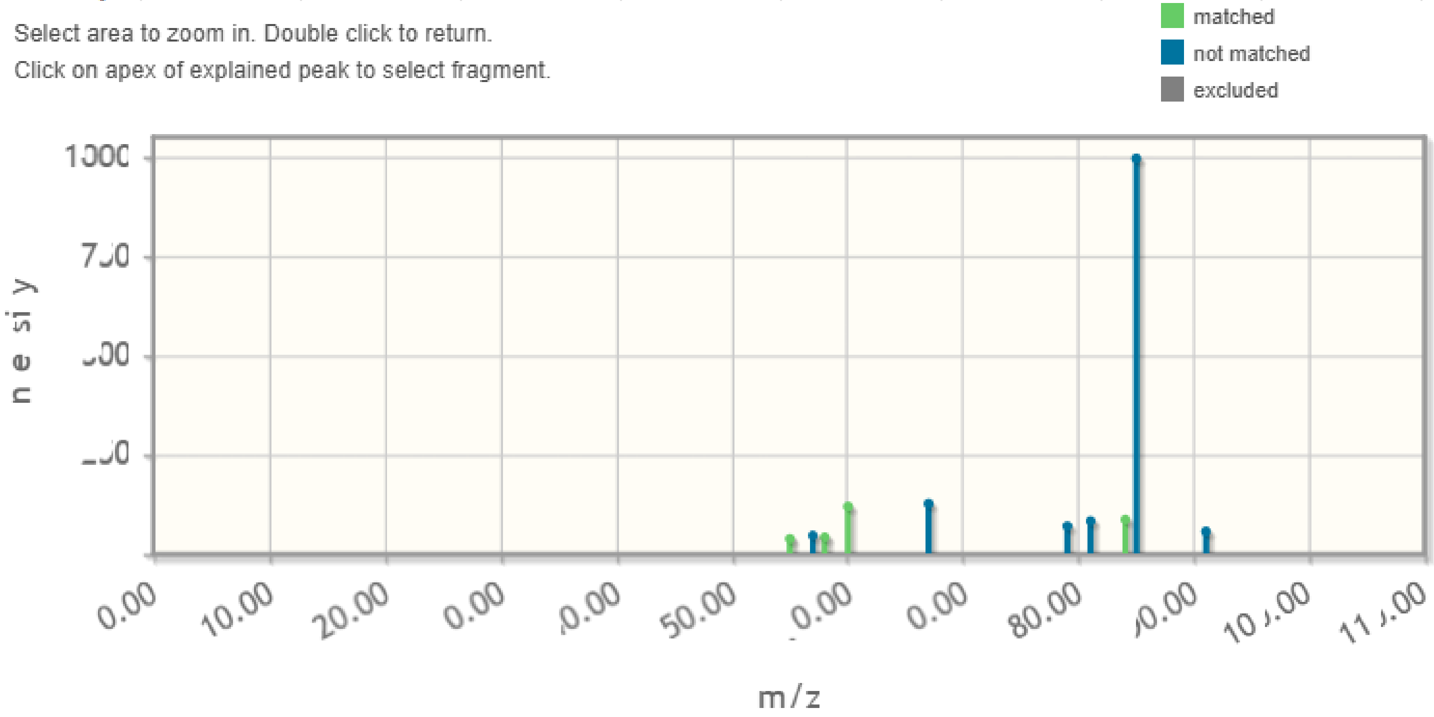

Fragment 1

Formula	[C4H8-H]+
Mass	55.05426
Peak m/z	55.0549049377441

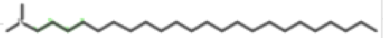

Fragment 2

Formula	[C3H8N]+
Mass	58.06516
Peak m/z	58.065803527832

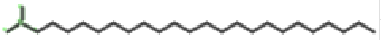

Fragment 3

Formula	[C3H8N+H]+H+
Mass	60.08082
Peak m/z	60.0813941955566

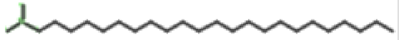

Fragment 4

Formula	[C5H11N-H]+
Mass	84.08082
Peak m/z	84.0812225341797

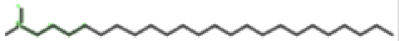

17) 367.41679 – $C_{25}H_{53}N$ Sirius N,N-dimethyltricosan-1-amine

4	Dimethyl(tricosyl)azanium	CCCCCCCCCCCC...	C25H53N	[M + H]+	-167,613	54,717%	10.072639	ORFRQNKZUWEX...	■

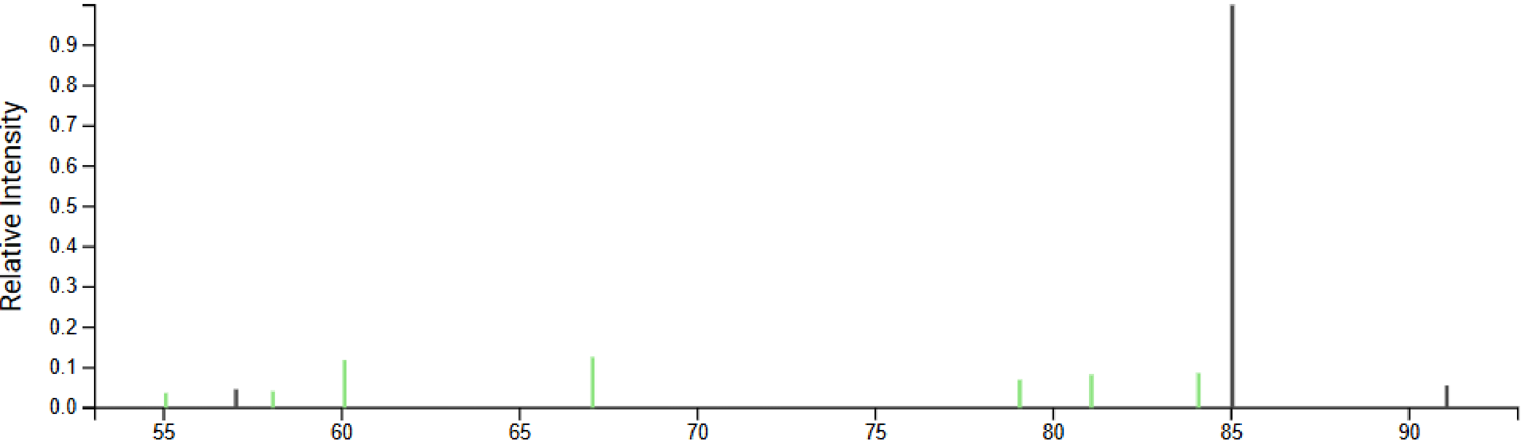

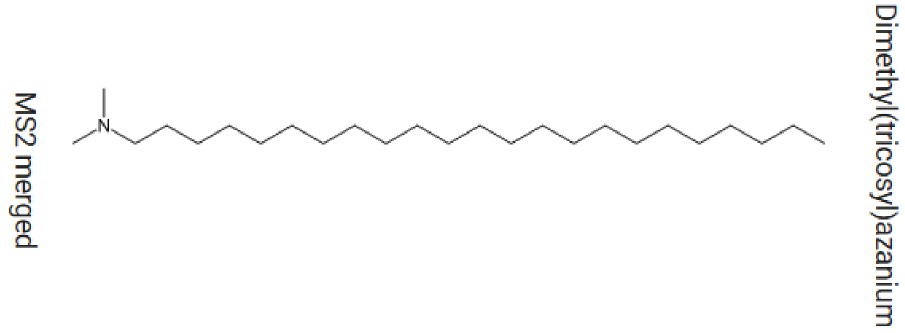

17) 367.41679 – $C_{25}H_{53}N$ Sirius Docosyltrimethylaminium

Rank	Name	SMILES	Molecular Formula	Adduct	CSI:FingerID Score	Tanimoto Similarity	XLogP	InChIKey	Lipid Class	Database	De Novo
1	Docosyltrimethylaminium	CCCCCCCCCCCC...	C25H53N	[M + H]+	-136,030	59,184%	9.248331	UCYFZDNMZYZS...		■	

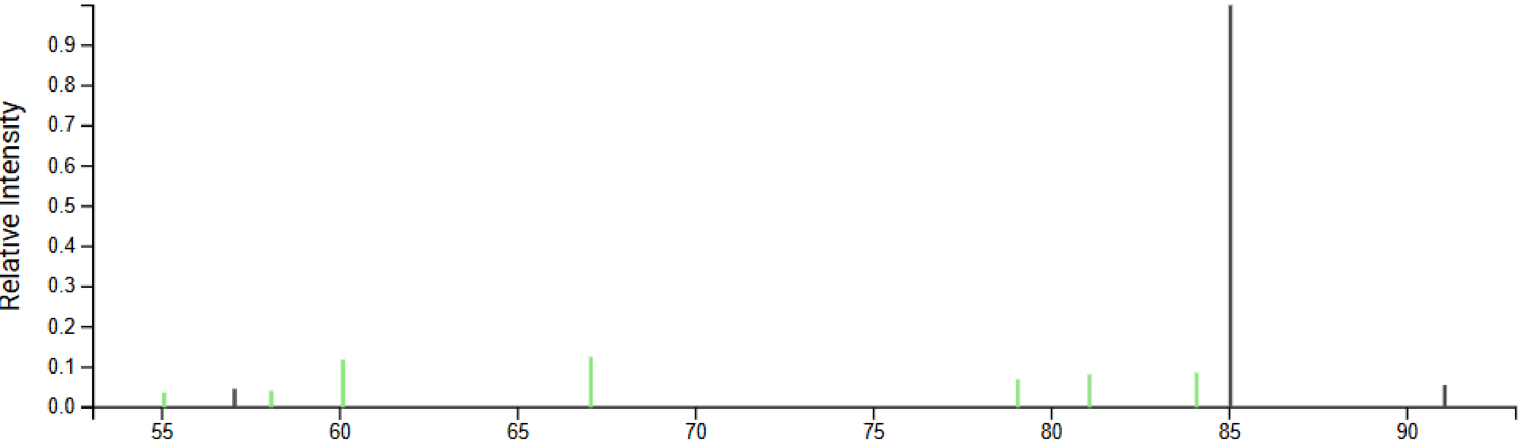

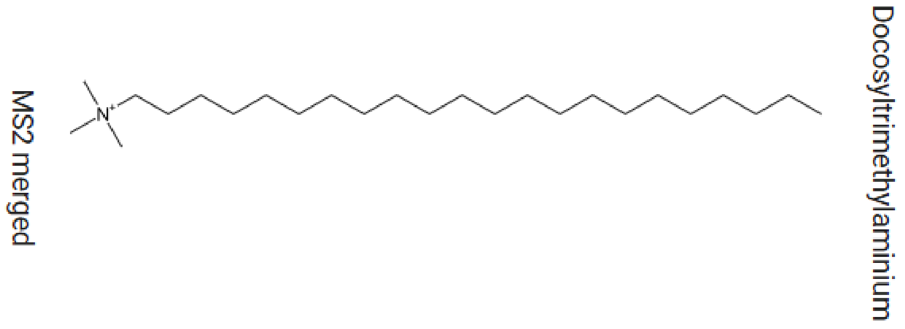

17) 367.41679 – C25H53N MSFinder N-dodecyl-N-methyldodecan-1-amine

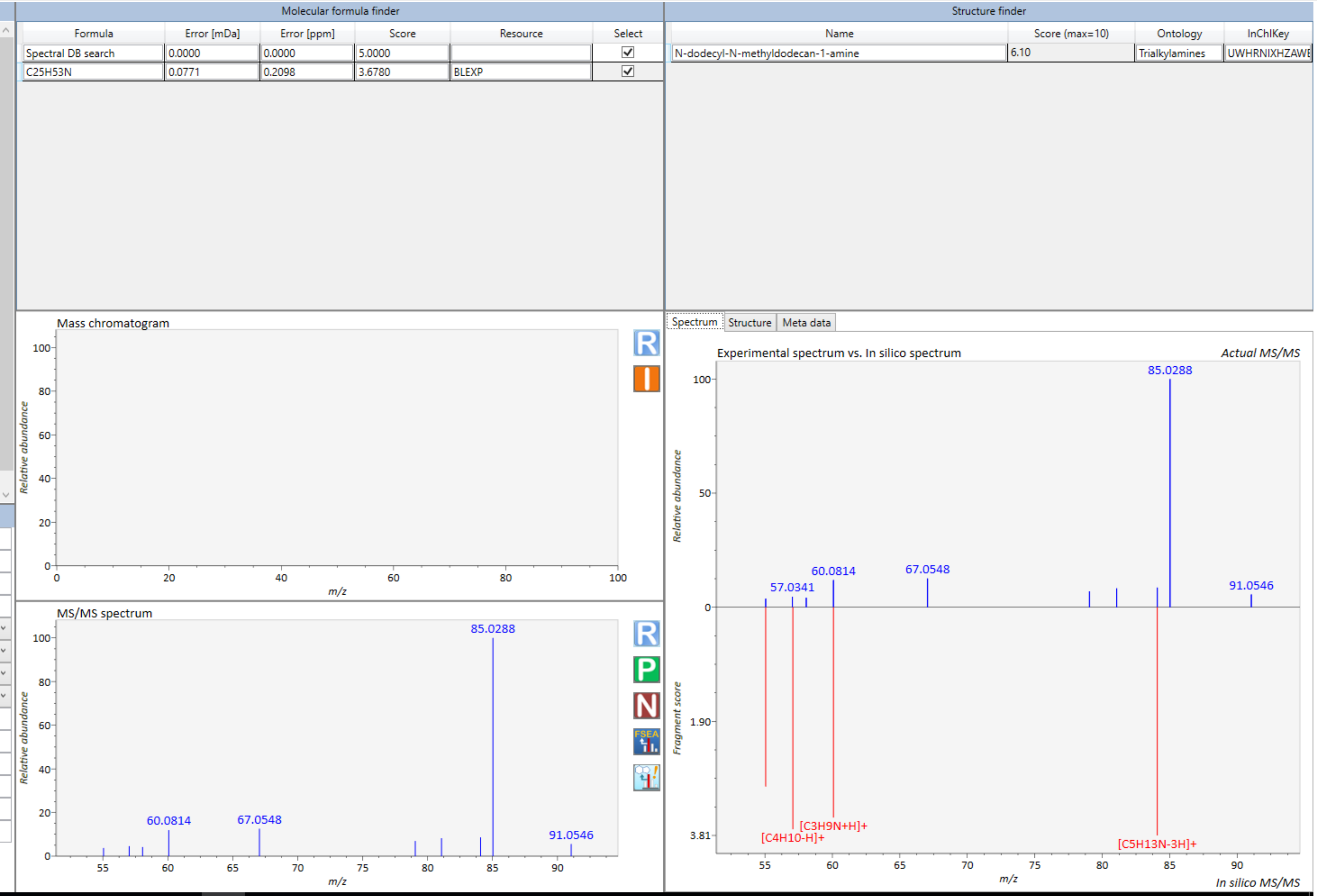

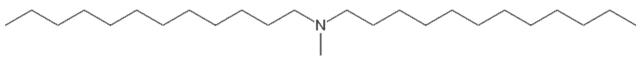

18) 383.19403 – C20H25N5O3 MetFrag Ethyl 4-[[1-[(3-ethoxyphenyl)methyl]pyrazolo[3,4-d]pyrimidin-4-yl]amino]butanoate

2	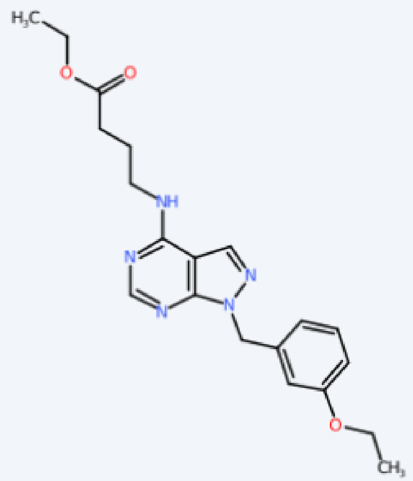 ethyl 4-[[1-[(3-ethoxyphenyl)methyl]pyrazolo[3,4-d]pyrimidin-4-yl]amino]butanoate	69299874<br>InChIKeyBlock1 = BNEKMWFKSVVOFZ	383.196	$C_{20}H_{25}N_5O_3$	0.934	Peaks: 7 / 11<br>Fragments<br>Scores<br>Download

Select area to zoom in. Double click to return.
Click on apex of explained peak to select fragment.

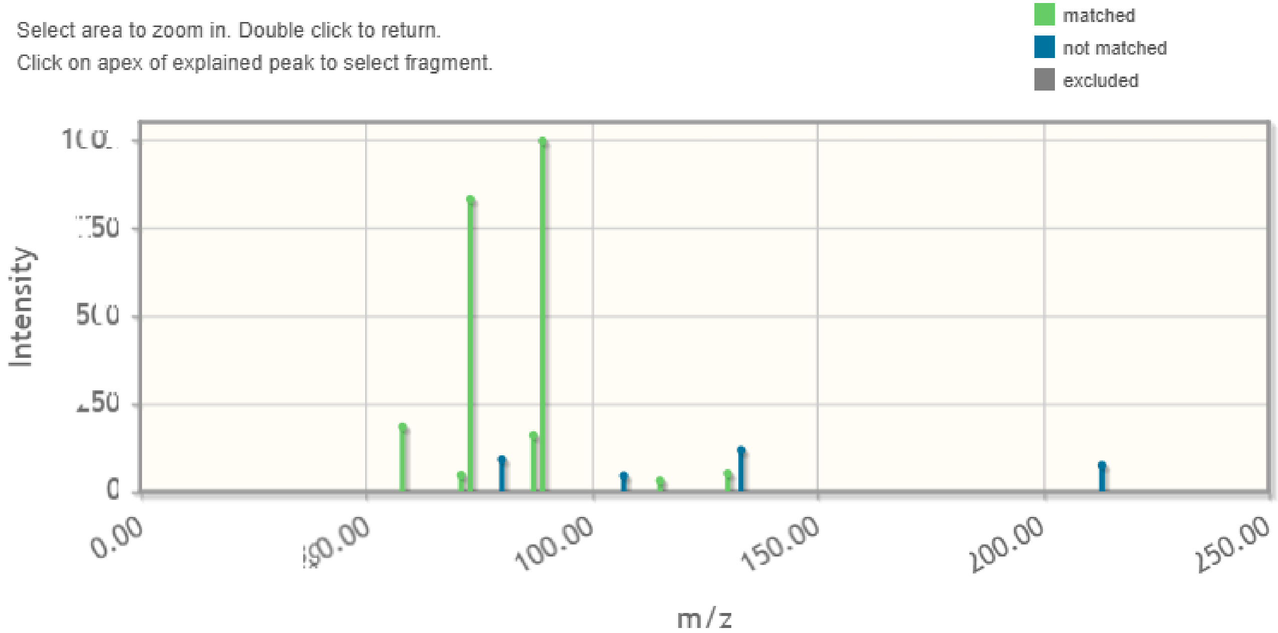

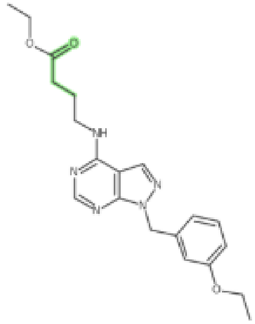

Fragment 1

Peak m/z: 58.0420074462891

Fragment Mass: 58.04134 Da

Fragment Formula: $[C_3H_4O+H]+H^+$

18) 383.19403 – C20H25N5O3 MetFrag Ethyl 4-[[1-[(3-ethoxyphenyl)methyl]pyrazolo[3,4-d]pyrimidin-4-yl]amino]butanoate

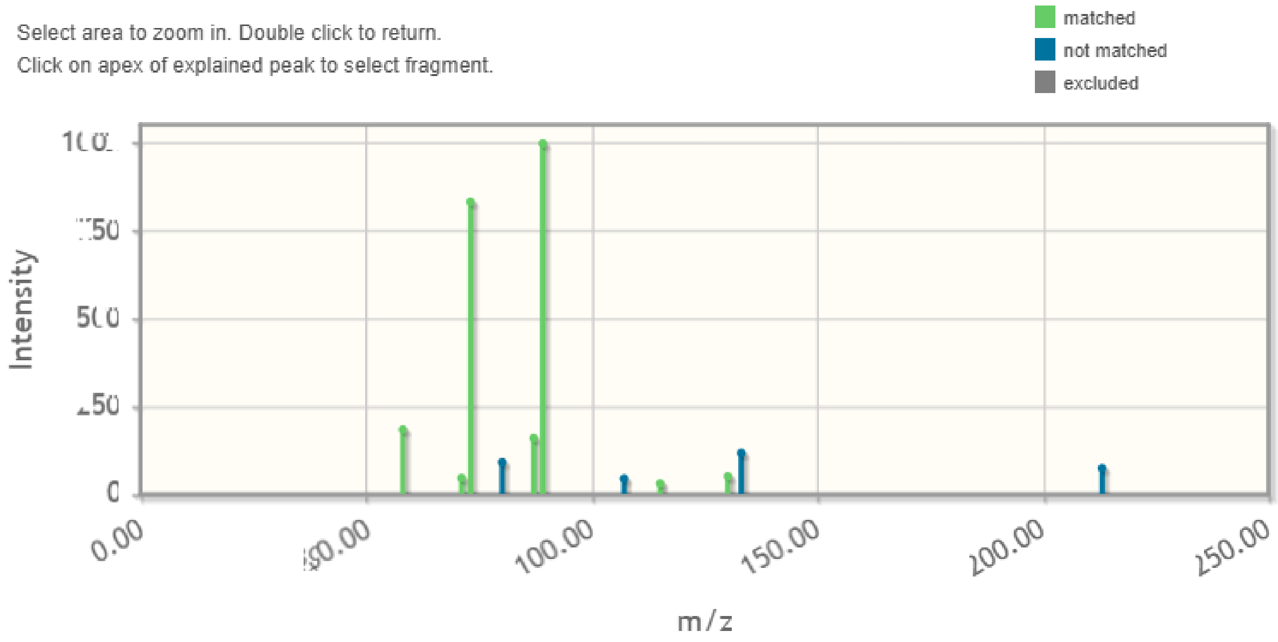

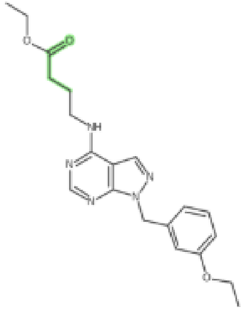

Fragment 1

Peak m/z: 58.0420074462891

Fragment Mass: 58.04134 Da

Fragment Formula: $[C_3H_4O+H]+H^+$

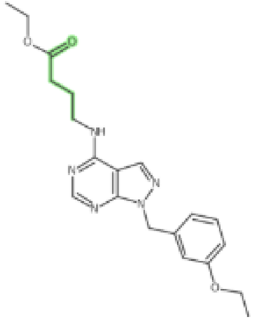

Fragment 2

Peak m/z: 71.0498046875

Fragment Mass: 71.04917 Da

Fragment Formula: $[C_4H_6O]+H^+$

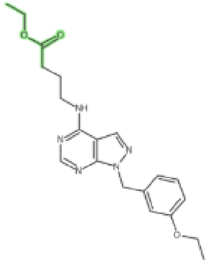

Fragment 4

Peak m/z: 87.0445175170898

Fragment Mass: 87.04408 Da

Fragment Formula: $[C_4H_7O_2]^+$

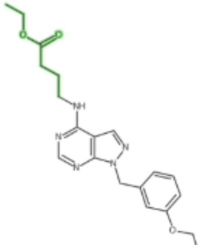

Fragment 6

Peak m/z: 115.075576782227

Fragment Mass: 115.0754 Da

Fragment Formula: $[C_6H_{11}O_2]^+$

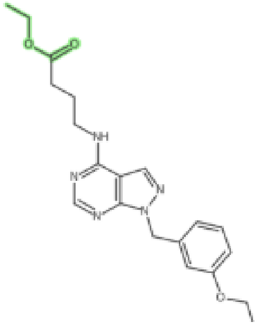

Fragment 3

Peak m/z: 73.0289764404297

Fragment Mass: 73.02842 Da

Fragment Formula: $[C_3H_5O_2]^+$

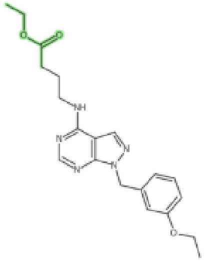

Fragment 5

Peak m/z: 89.060173034668

Fragment Mass: 89.05974 Da

Fragment Formula: $[C_4H_7O_2+H]+H^+$

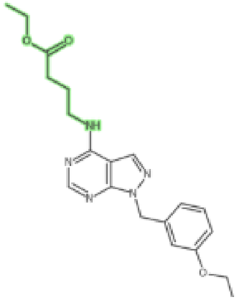

Fragment 7

Peak m/z: 130.08625793457

Fragment Mass: 130.0863 Da

Fragment Formula: $[C_6H_{12}NO_2]^+$

19) 390.27520 – C24H38O4 MetFrag 9”-Carboxy-alpha-chromanol

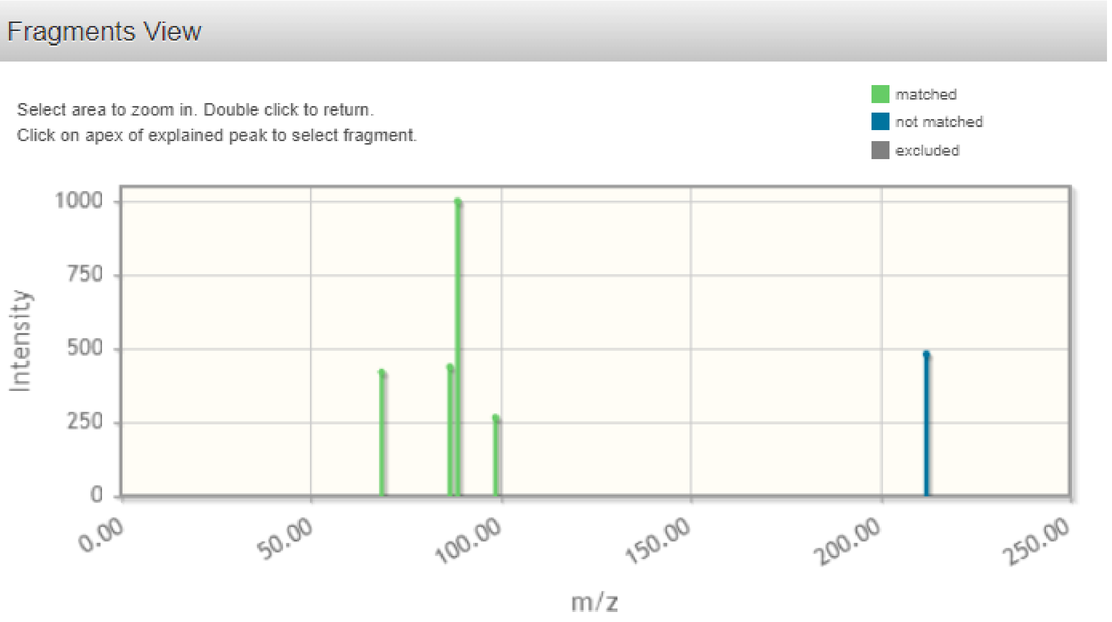

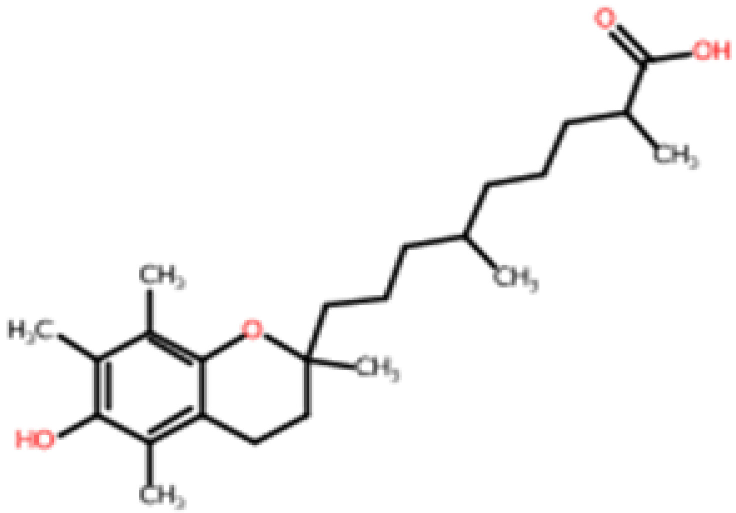

**Identifier**	HMDB12866
**MetFrag**	36.1948621875557
**InChI**	InChI=1S/C24H38O4/c1-15(9-7-11-16(2)23(26)27)10-8-13-24(6)14-12-20-19(5)21(25)17(3)18(4)22(20)28-24/h15-16,25H,7-14H2,1-6H3,(H,26,27)/t15-,16?,24-/m0/s1
**LossStat**	-92.5338098624521
**Monoisoto**	390.277
**Compoun**	9"-Carboxy-alpha-chromanol
**Identifier**	HMDB12866
**Molecular**	C24H38O4
**SMILES**	CC1=C(C(=C2CCC(OC2=C1C)(C)CCCC(C)CCCC(C)C(=O)O)C)O
**SpectralS**	0.16267649530103132
**PeakStat**	-41.85291660150831
**ExactSpe**	0.0

19) 390.27520 – C24H38O4 MetFrag 9”-Carboxy-alpha-chromanol

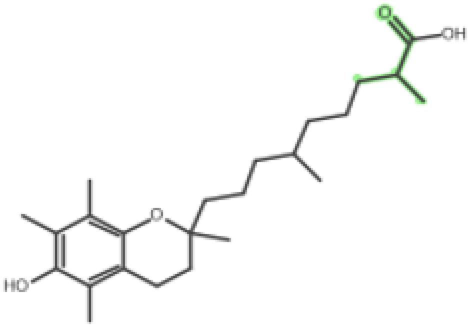

Fragment 1	
Formula	[C4H6O-H]+
Mass	69.03351
Peak m/z	69.0335998535156

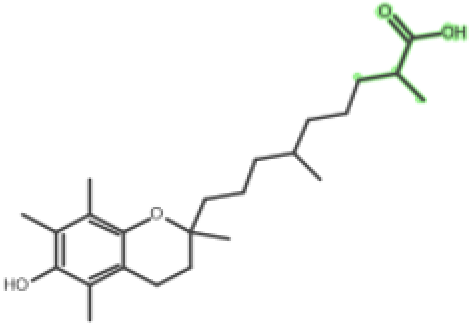

Fragment 2	
Formula	[C4H7O2]+
Mass	87.04408
Peak m/z	87.0439376831055

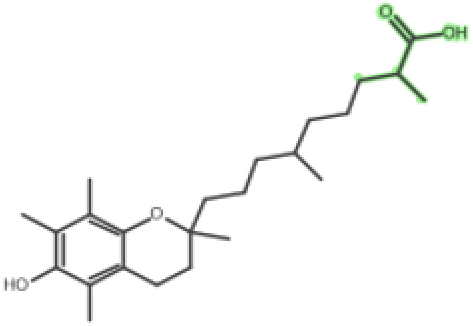

Fragment 3	
Formula	[C4H7O2+H]+H+
Mass	89.05974
Peak m/z	89.0596466064453

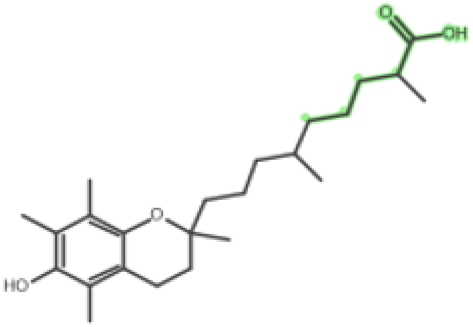

Fragment 4	
Formula	[C5H8O2-H]+
Mass	99.04408
Peak m/z	99.043830871582

19) 390.27520 – C24H38O4 MetFrag Nutriacholic acid

4	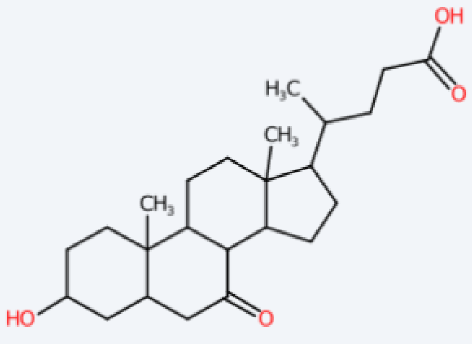 Nutriacholic acid	HMDB00467<br>InChIKeyBlock1 = DXOCDBGWDZAYRQ	390.277	$C_{24}H_{38}O_4$	0.7701	Peaks: 4 / 5<br>Fragments<br>Scores<br>wnload

Select area to zoom in. Double click to return.
Click on apex of explained peak to select fragment.

matched
not matched
excluded

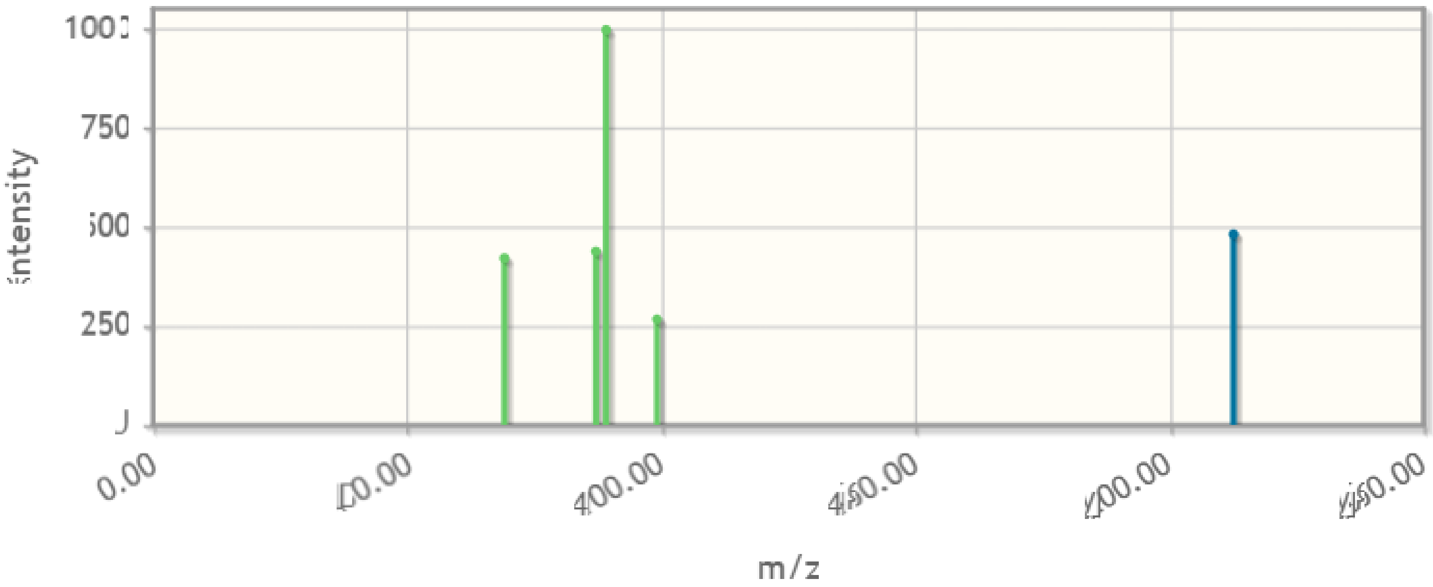

Fragment 1

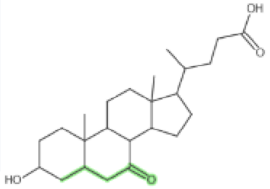

Peak m/z: 69.0335998535156

Fragment Mass: 69.03351 Da

Fragment Formula: $[C_4H_5O]^+$

Fragment 2

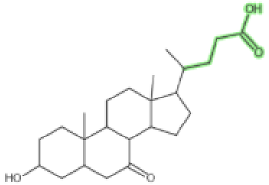

Peak m/z: 87.0439376831055

Fragment Mass: 87.04408 Da

Fragment Formula: $[C_4H_6O_2]+H^+$

Fragment 3

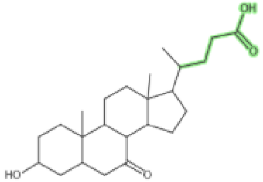

Peak m/z: 89.0596466064453

Fragment Mass: 89.05974 Da

Fragment Formula: $[C_4H_6O_2+2H]+H^+$

Fragment 4

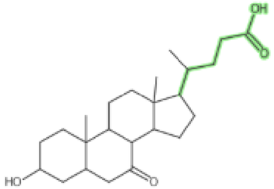

Peak m/z: 99.043830871582

Fragment Mass: 99.04408 Da

Fragment Formula: $[C_5H_7O_2]^+$

19) 390.27520 – C24H38O4 MSFinder Nutriacholic acid

Molecular formula finder

Formula	Error [mDa]	Error [ppm]	Score	Resource	Select
Spectral DB search	0.0000	0.0000	5.0000		☑
C24H38O4	0.2861	0.7331	4.1120	HMDB,KNApSAcK,ChEBI,T3D	☑
C20H34N6O2	-2.3992	-6.1475	3.5510	BLEXP,COCONUT	☑
C26H34N2O	-9.6098	-24.6236	3.4800	KNApSAcK,UNPD,BLEXP,CO	☑
C23H38N2OS	-6.2387	-15.9856	3.3780	COCONUT	☑
C17H46N2OS3	0.5035	1.2901	3.0300		☑
C18H39N4O3P	-0.7458	-1.9110	3.0280		☐
C17H38N6O2S	0.9719	2.4903	3.0260		☐
C18H47O2PS2	-1.2142	-3.1111	3.0240		☐
C25H34N4	1.6235	4.1599	3.0180		☐
C10H38N12S2	1.6577	4.2475	3.0170		☐
C13H34N12S	-1.7135	-4.3905	3.0160		☐
C10H30N16O	2.1260	5.4474	3.0090		☐
C15H43N4O3PS	2.6253	6.7267	2.9970		☐

Structure finder

Name	Score (max=10)	Ontology	InChIKey
UNPD112808	7.27	Eunicellane and a	DUYIECPEKRPRF
UNPD137425	7.24	Eunicellane and a	ZXQCKAYQYGLLE
Amphidinolide W;(+)-Amphidinolide W	7.24	Macrolides and a	VWINOFLMOJPX
UNPD159876	7.22	Annonaceous ac	WPGYLBYQMCFK
Nutriacholic acid	7.20	Monohydroxy bil	DXOCDBGWDZA
UNPD183415	7.17	Oxepanes	GRBBMLGQMUN
antroquinonol	7.17	Diterpenoids	LJTSIMVOOOLKC
UNPD140573	7.13	Macrolides and a	UKGXHGYWYJBR
UNPD118031	7.13	Macrolides and a	YYLMDSJRCIURA
5,5,6-trimethyl-2-(3-methyl-3-[(2-methylbutan-2-yl)peroxy]but-1-yn	7.08	Bicyclic monoter	GAFQCQFVIRUO
1,7,7-trimethyl-2-(3-methyl-3-[(2-methylbutan-2-yl)peroxy]but-1-yn	7.08	Bicyclic monoter	LSQKTLIPGPORG
UNPD96775	7.06	Diterpenoids	HUECDSPUQRRC
UNPD140159	7.05	Diterpenoids	XGDXMNWMKFN
7-Hydroxy-3-oxocholanoic acid	7.05	Monohydroxy bil	KNVADAPHVNKT

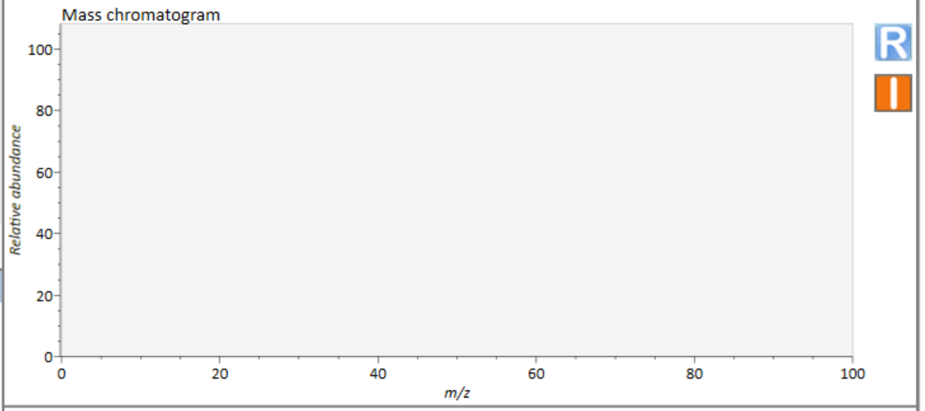

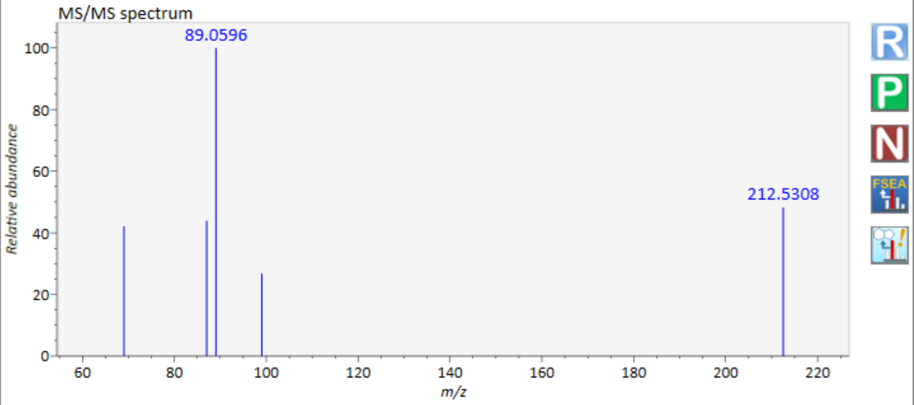

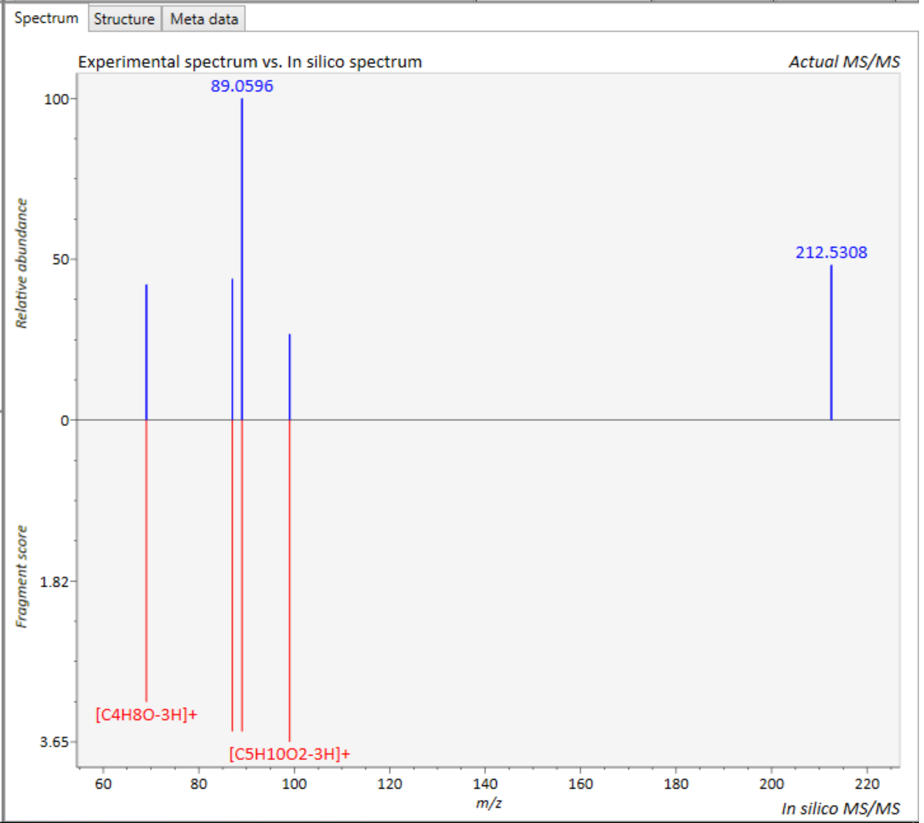

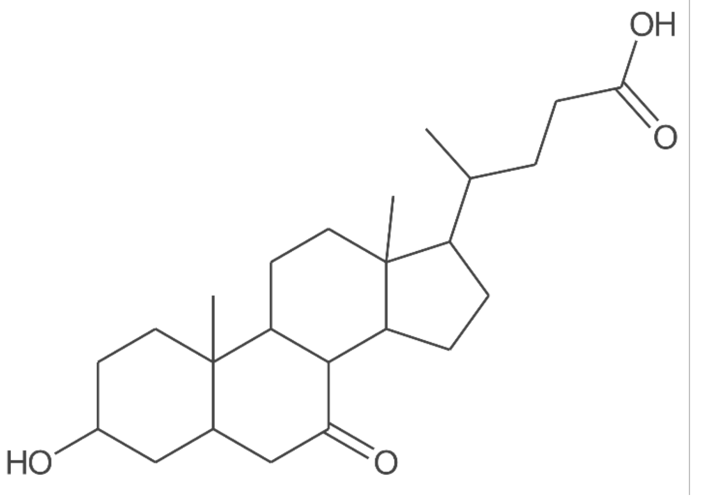

20) 437.49462 – C30H63N MetFrag N-decyl-N-ethyl-octadecan-1-amine

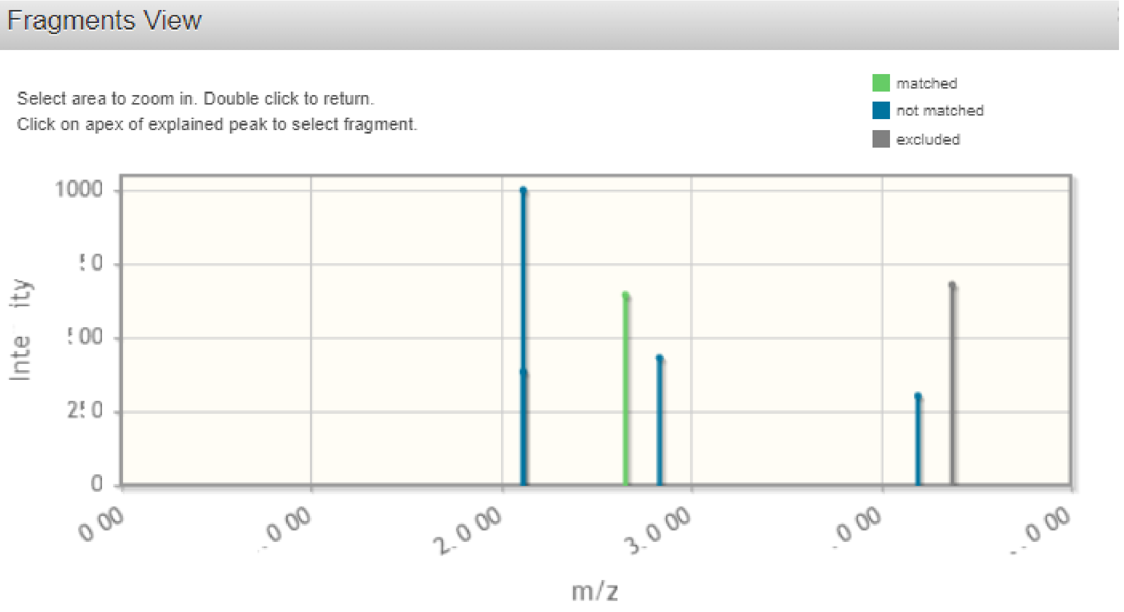

**Identifier**	19913486
**MetFrag**	57.62694167056545
**InChI**	InChI=1S/C30H63N/c1-4-7-9-11-13-15-16-17-18-19-20-21-22-24-26-28-30-31(6-3)29-27-25-23-14-12-10-8-5-2/h4-30H2,1-3H3
**LossStat**	-35.90930586715402
**Monoisot**	437.496
**Compoun**	N-decyl-N-ethyl-octadecan-1-amine
**Identifier**	19913486
**Molecular**	C30H63N
**SMILES**	CCCCCCCCCCCCCCCCCCN(CC)CCCCCCCCCC
**SpectralS**	0.0
**PeakStat**	-19.61500287024794
**ExactSpe**	0.0

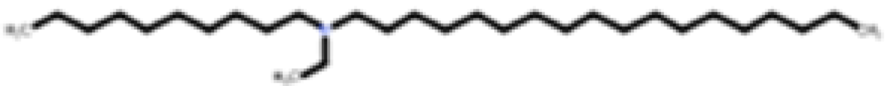

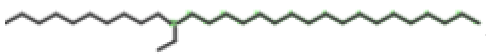

**Fragment 1**	
**Formula**	[C18H37N-H]+
**Mass**	266.2844
**Peak m/z**	266.281921386719

23) 519.33092 – C26H50NO7P MetFrag LysoPC(18:2(9Z, 12Z))

**Identifier**	HMDB10386
**MetFrag**	133.89277933069326
**InChI**	InChI=1S/C26H50NO7P/c1-5-6-7-8-9-10-11-12-13-14-15-16-17-18-19-20-26(29)32-23-25(28)24-34-35(30,31)33-22-21-27(2,3)4/h9-10,12-13,25,28H,5-8,11,14-24H2,1-4H3/b10-9-,13-12-/t25-/m1/s1
**LossStat**	-122.67282209755456
**Monoisot**	519.332
**Compoun**	LysoPC(18:2(9Z,12Z))
**Identifier**	HMDB10386
**Molecular**	C26H50NO7P
**SMILES**	CCCCCC=CCC=CCCCCCCCC(=O)OCC(COP(=O)([O-])OCC[N+](C)(C)C)O
**SpectralS**	1.379833353062319
**PeakStat**	-50.88678014809711
**ExactSpe**	0.0

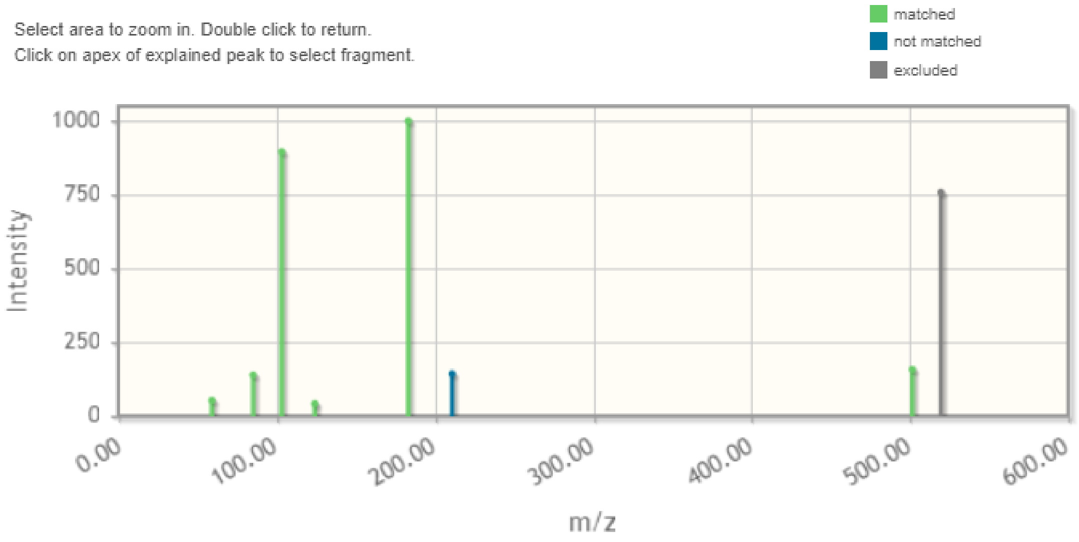

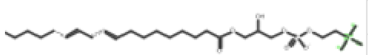

**Fragment 1**	
**Formula**	[C3H9N]+H+
**Mass**	60.08082
**Peak m/z**	60.0810813903809

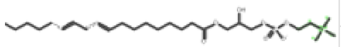

**Fragment 2**	
**Formula**	[C5H13N-H]+
**Mass**	86.09648
**Peak m/z**	86.0963897705078

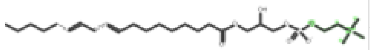

**Fragment 3**	
**Formula**	[C5H13NO]+H+
**Mass**	104.10705
**Peak m/z**	104.106658935547

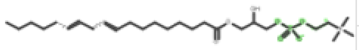

**Fragment 4**	
**Formula**	[C2H4O4P+H]+H+
**Mass**	124.99983
**Peak m/z**	124.999145507813

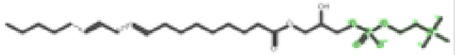

**Fragment 5**	
**Formula**	[C5H13NO4P+H]+H+
**Mass**	184.07337
**Peak m/z**	184.072128295898

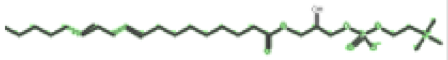

**Fragment 6**	
**Formula**	[C26H49NO6P]+
**Mass**	502.32941
**Peak m/z**	502.325439453125

23) 519.33092 – C26H50NO7P MSFinder LysoPC(18:2(9Z, 12Z))

Molecular formula finder

Formula	Error [mDa]	Error [ppm]	Score	Resource	Select
Spectral DB search	0.0000	0.0000	5.0000		☑
C26H50NO7P	-0.2340	-0.4506	3.9540	HMDB,ChEBI,YMDB,FooDB,E	☑
C33H45NO4	2.1354	4.1118	3.8150	NPA,COCONUT,MINE	☑
C27H45N5O5	9.3460	17.9958	3.5560	ChEBI,DrugBank,UNPD,BLEX	☑
C19H58N3O4PS3	-0.0166	-0.0320	3.1440		☑
C19H50N7O5PS	0.4518	0.8700	3.1430		☑
C20H51N5O6P2	-1.2659	-2.4376	3.1360		☐
C12H42N17O4P	1.6059	3.0922	3.1310		☐
C25H51N3O4P2	2.7568	5.3083	3.1070		☐
C22H46N7O5P	-2.9193	-5.6213	3.1020		☐
C27H46N5O3P	1.1034	2.1246	3.1000		☐
C23H54NO7PS	3.1372	6.0408	3.0960		☐
C22H54N3O4PS2	-3.3877	-6.5232	3.0880		☐
C15H46N13O3PS	-2.2336	-4.3009	3.0810		☐

Structure finder

Name	Score (max=10)	Ontology	InChIKey
LysoPC(18:2(9Z,12Z))	7.45	1-acyl-sn-glycero	SPJFYYJXNPEZDW
2-linoleoyl-sn-glycero-3-phosphocholine	6.93	2-acyl-sn-glycero	LSUXCWJOIAWG(
PC(18:2(2E,4E)/0:0)	6.85	1-acyl-sn-glycero	CLDXAAPENVNFA

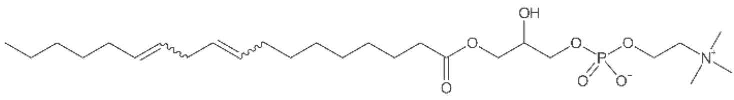

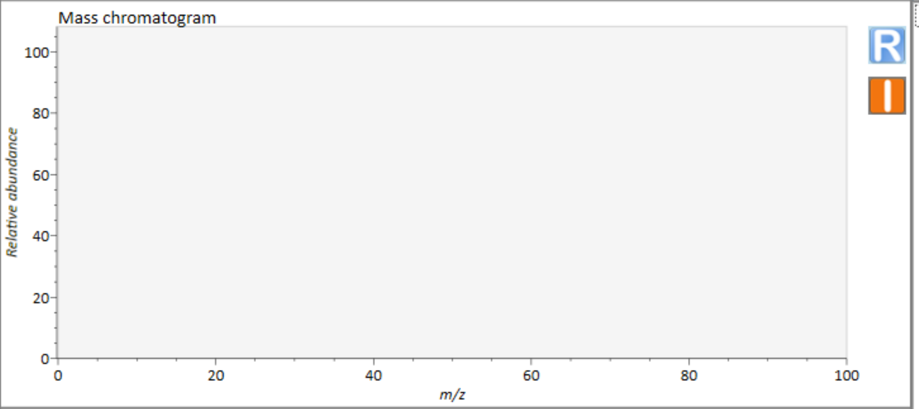

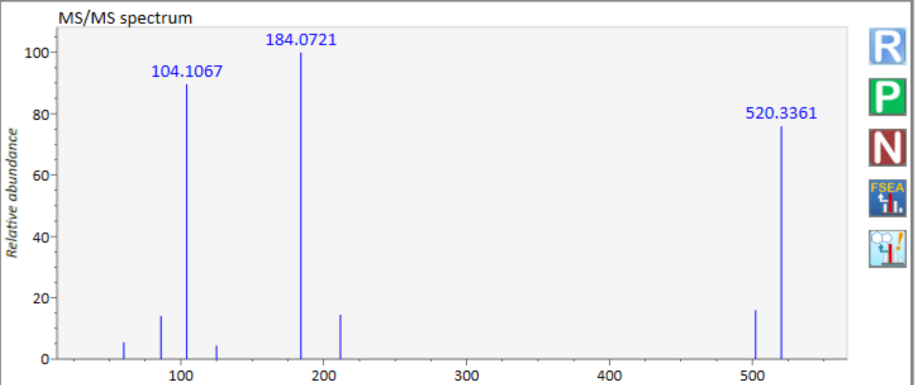

Spectrum | Structure | Meta data

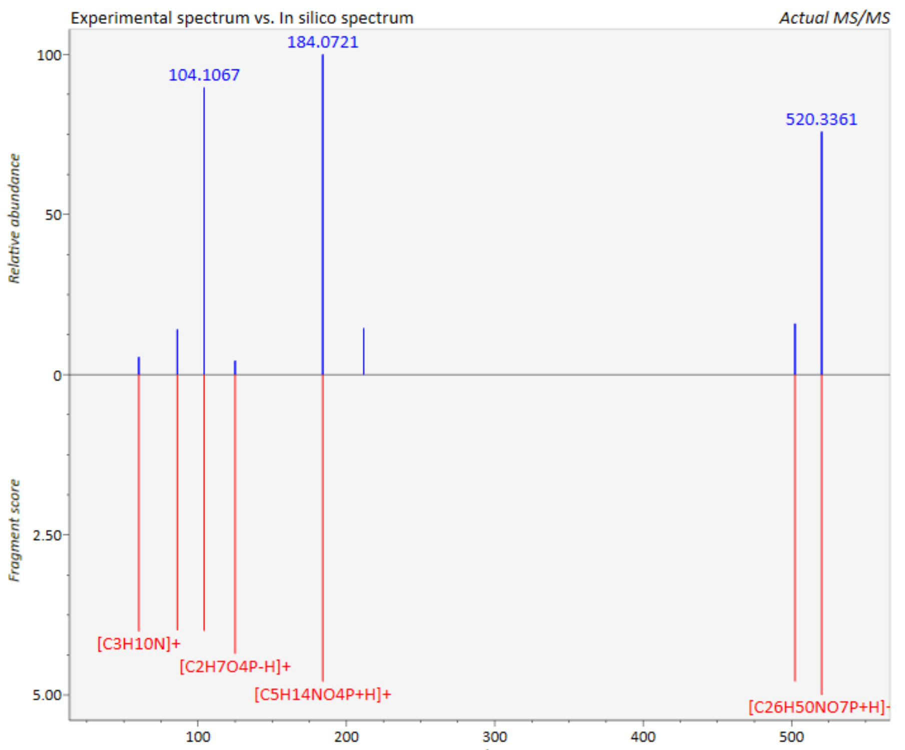

23) 519.33092 – C26H50NO7P Sirius LysoPC(18:2(9Z, 12Z))

Rank	Name	SMILES	Molecular Formula	Adduct	CSI:FingerID Score	Tanimoto Similarity	XLogP	InChIKey	Lipid Class	Database	De Novo
1	[(2R)-2-hydroxy-3-[(9E,11E)-...	CCCCCCC=CC=C...	C26H50NO7P	[M + H]+	-185,526	81,726%	5.3	AXFAVZQXPFQIEI		■	

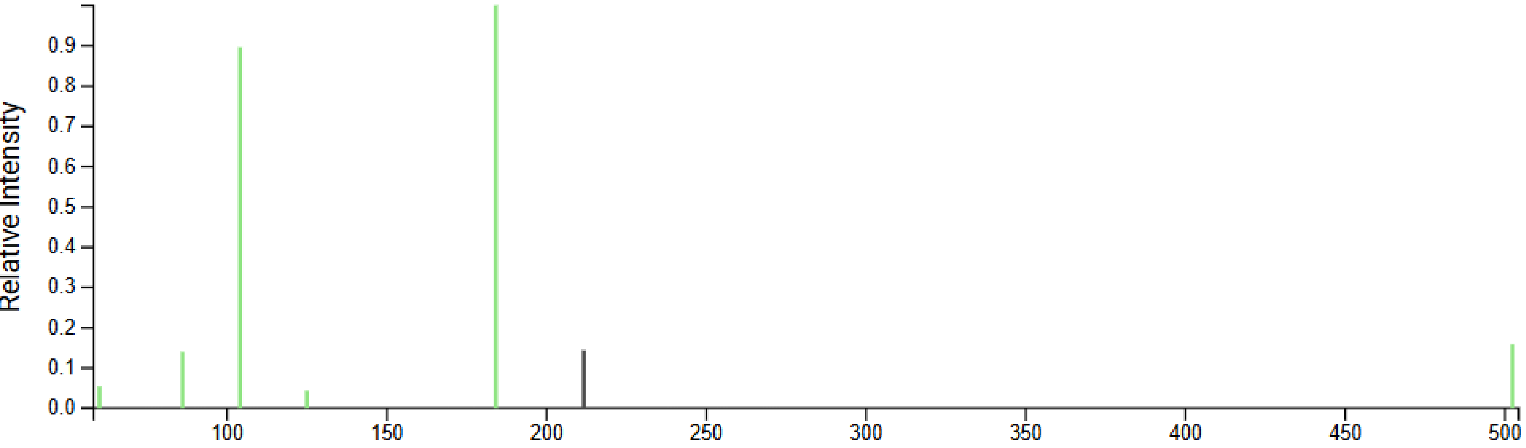

MS2 merged

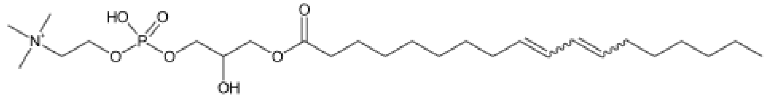

[(2R)-2-hydroxy-3-[(9E,11E)-octadeca-...

23) 608.21883 – C27H39N6O4P2 MSFinder [(2R,3S,4R,5R,6S)-5-acetamido-3,4-diacetoxy-6-[[5-[2-(3,4-dimethoxyphenyl)ethyl]-4-methyl-1,2,4-triazol-3-yl]sulfanyl]tetrahydropyran-2-yl]methyl acetate

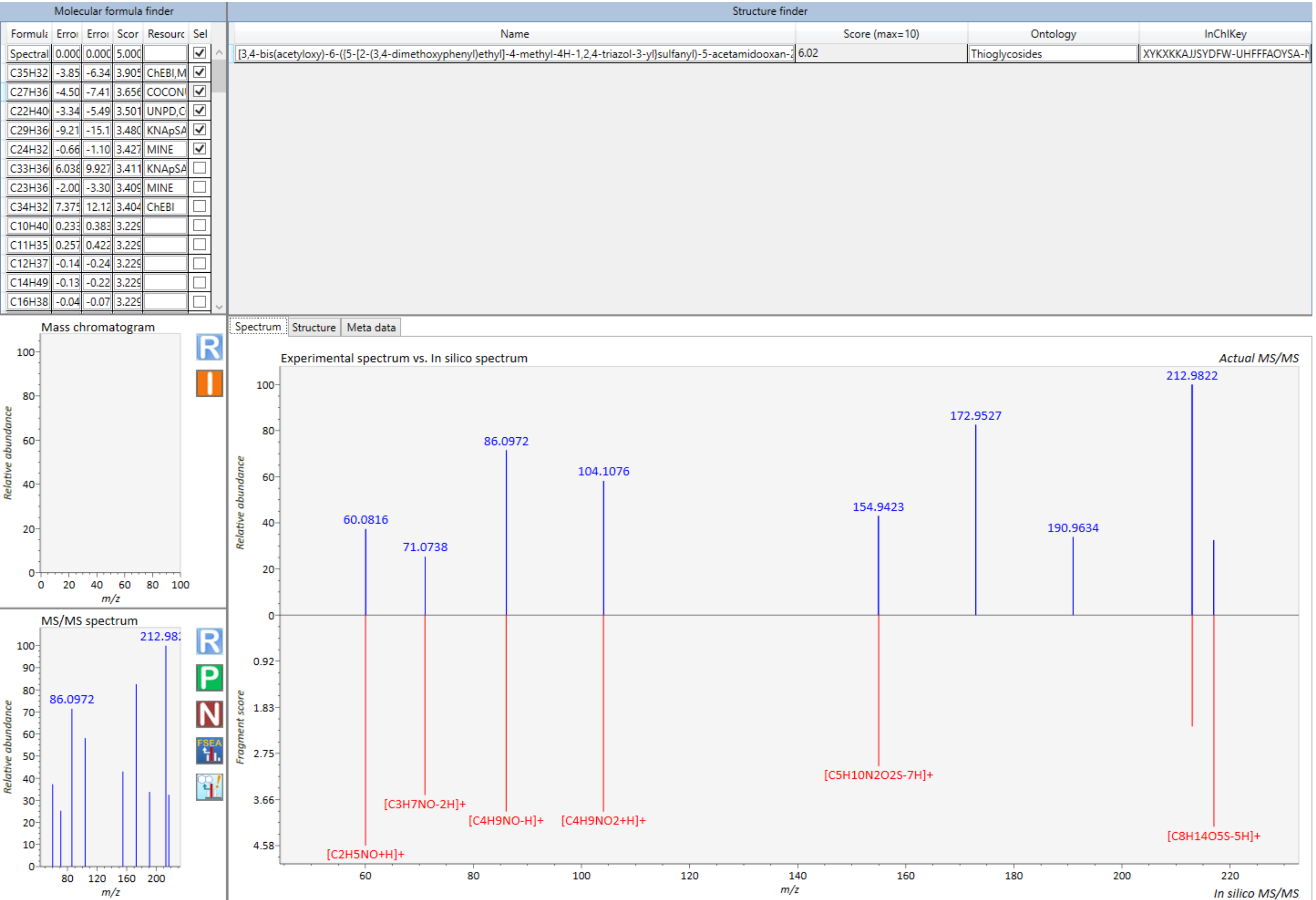

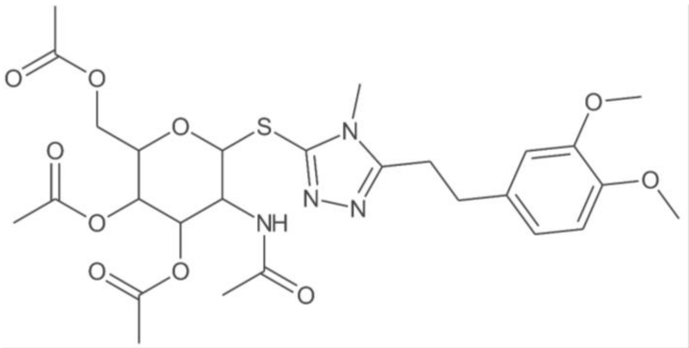

Supplement: Supplementary file 1 [file biomedicines-14-00872-s001.zip › Fig. S3.pdf]
