# Supplementary material for: Evaluation of MassFrontier, MetFrag, MS-FINDER, and SIRIUS for Metabolite Annotation Using an Experimental LC–HRMS Dataset
Source: Biomedicines. 2026 Apr 10;14(4):872. doi: 10.3390/biomedicines14040872 (PMC13113853; doi:10.3390/biomedicines14040872)
Supplement: Supplementary file 1 [file biomedicines-14-00872-s001.zip › Fig. S4.pdf]

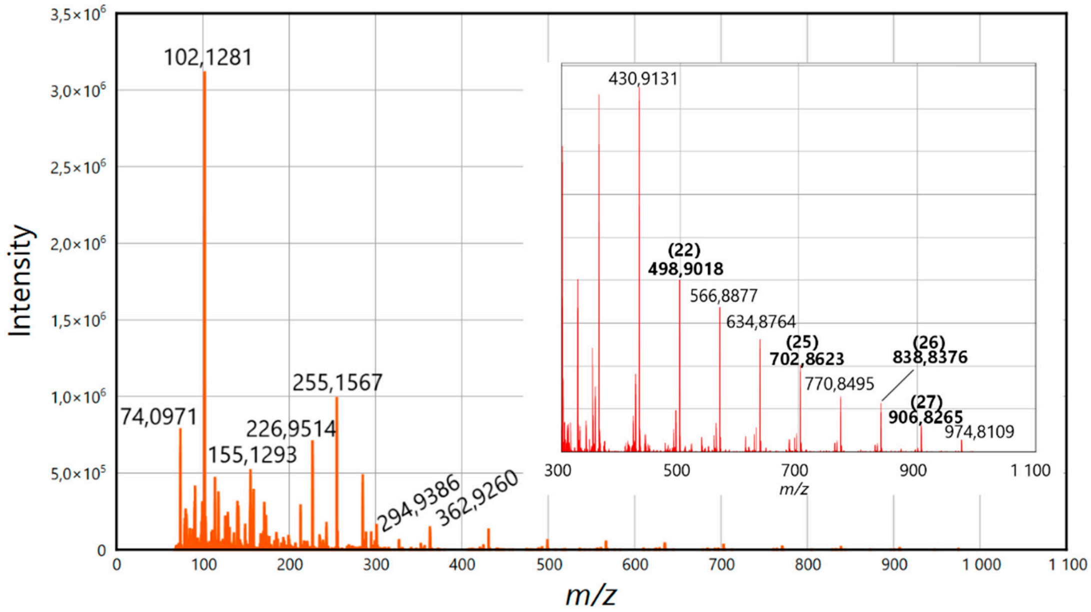

Figure S4. Mass spectrum of sodium formate cluster ions. The m/z of detected sodium formate cluster ions are highlighted in bold, compound numbers are given in parentheses.
