# Supplementary figures and images for: Evaluation of MassFrontier, MetFrag, MS-FINDER, and SIRIUS for Metabolite Annotation Using an Experimental LC–HRMS Dataset
Source: Biomedicines. 2026 Apr 10;14(4):872. doi: 10.3390/biomedicines14040872 (PMC13113853; doi:10.3390/biomedicines14040872)

M/z

t-SIM

PRM

117.07860

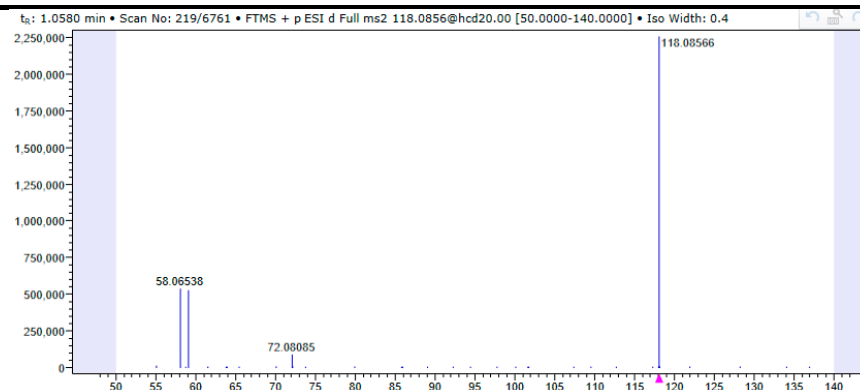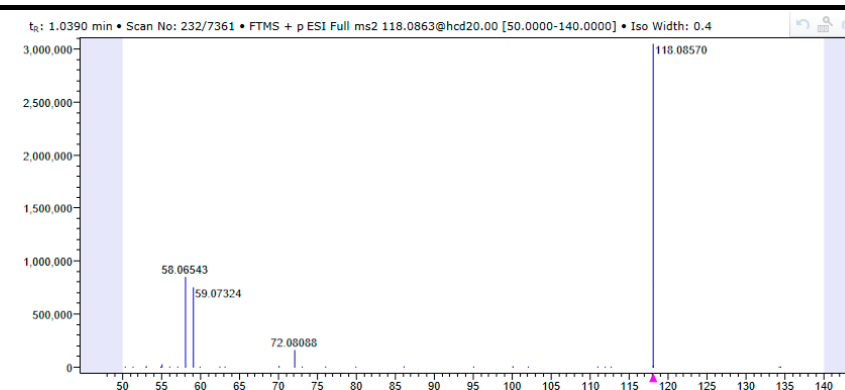

145.10947

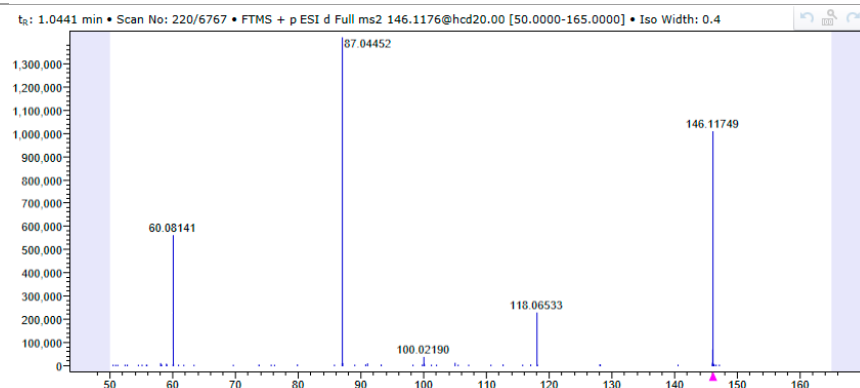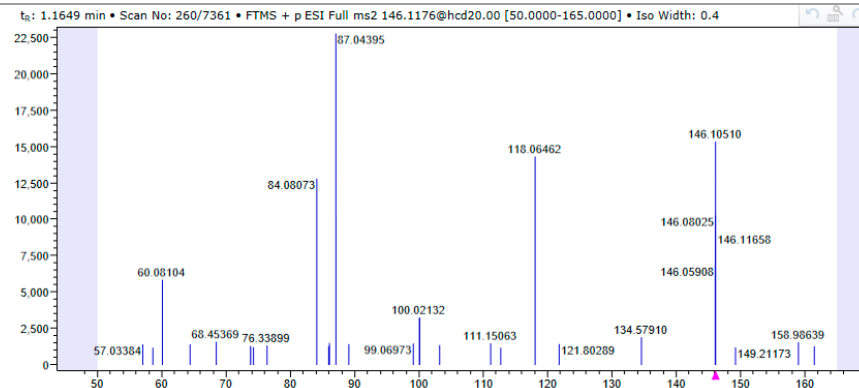

172.07038

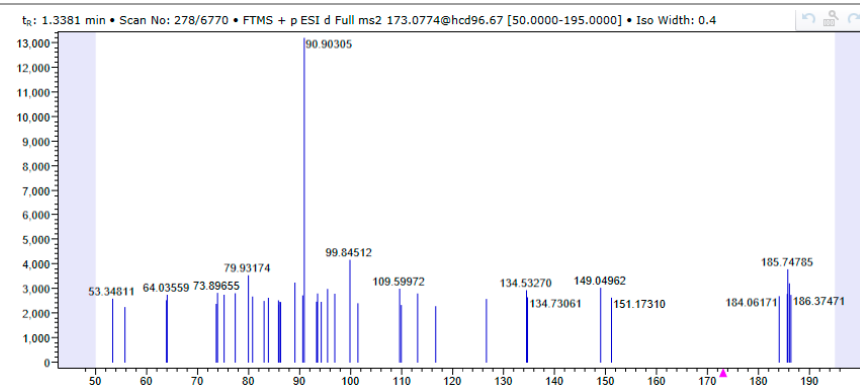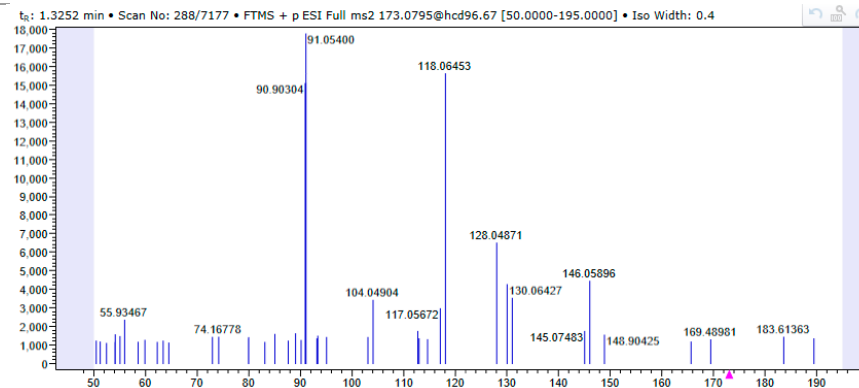

186.07594

-

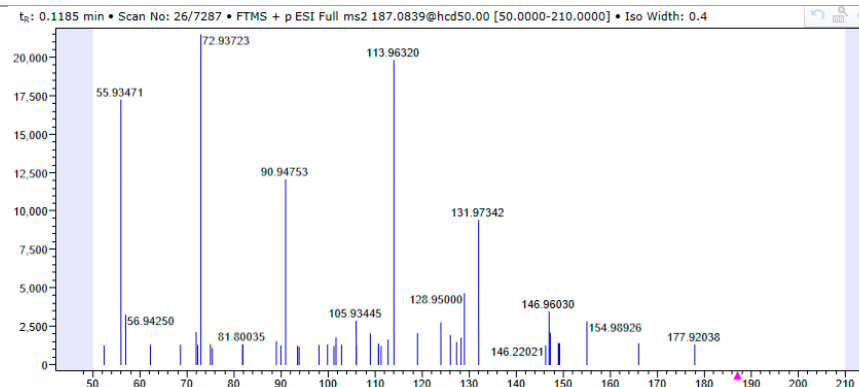

201.17209

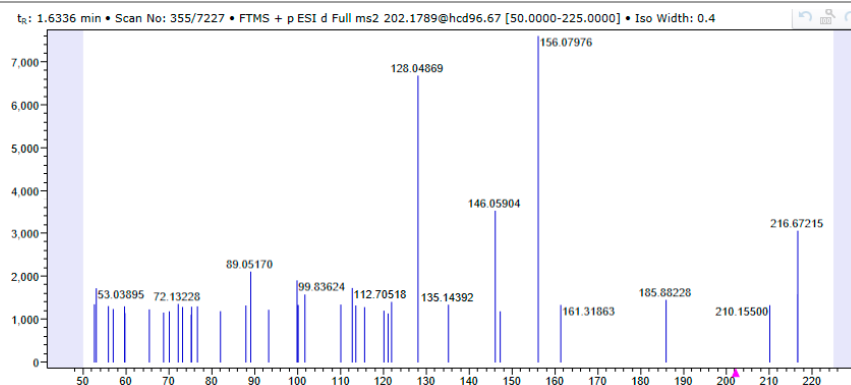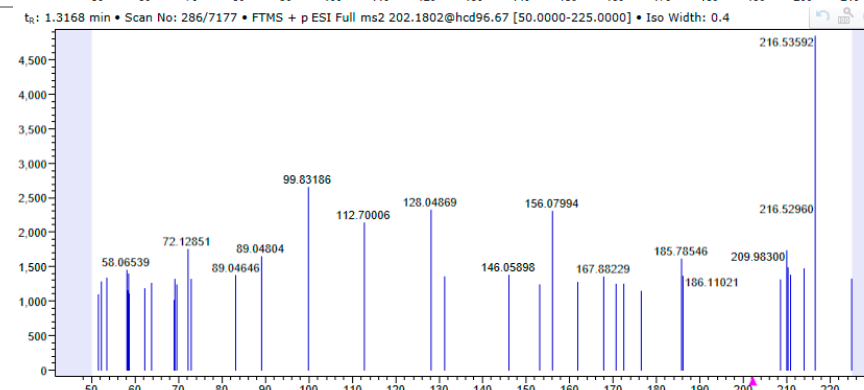

214.11747

-

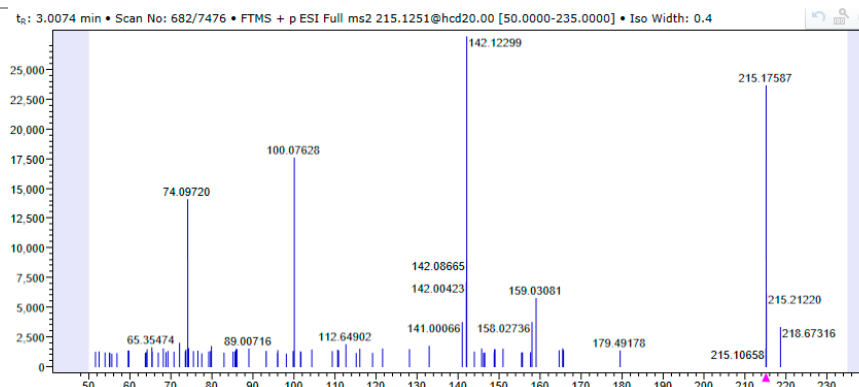

216.09639

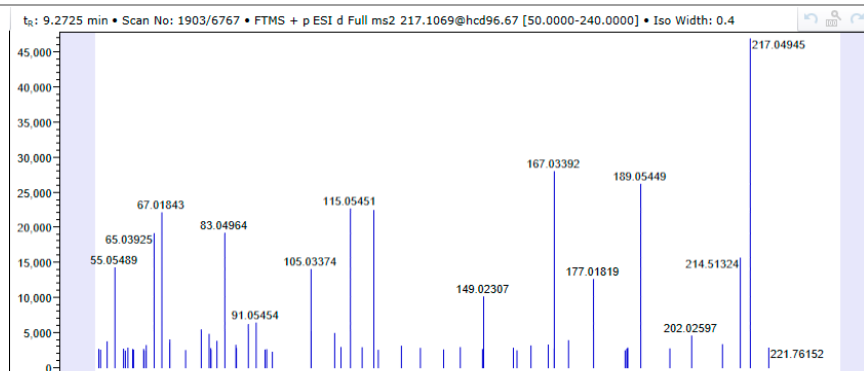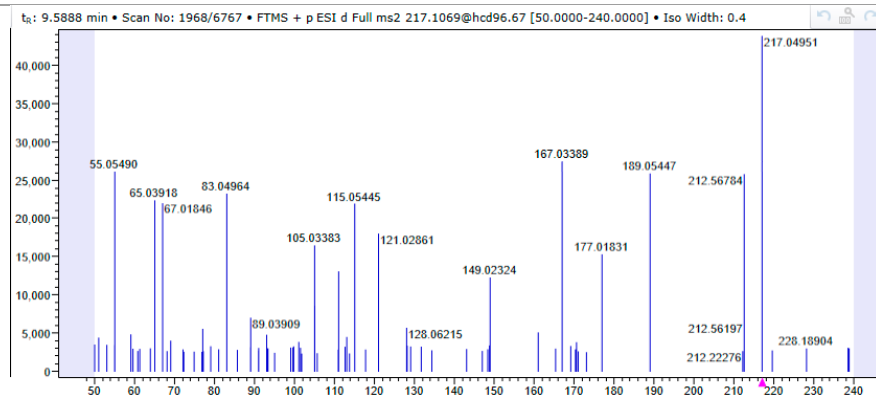

269.30702

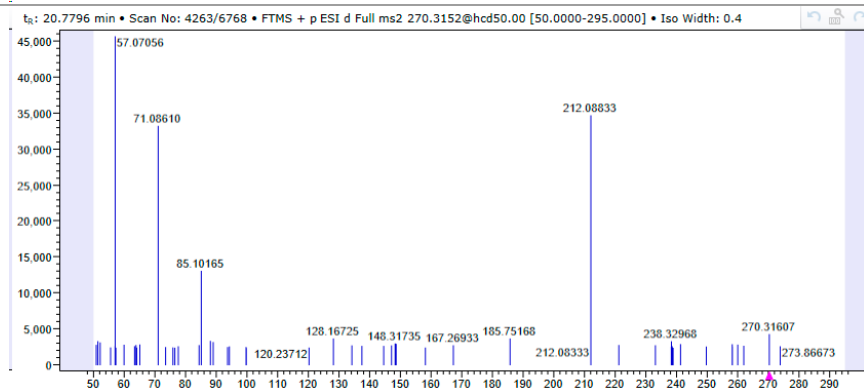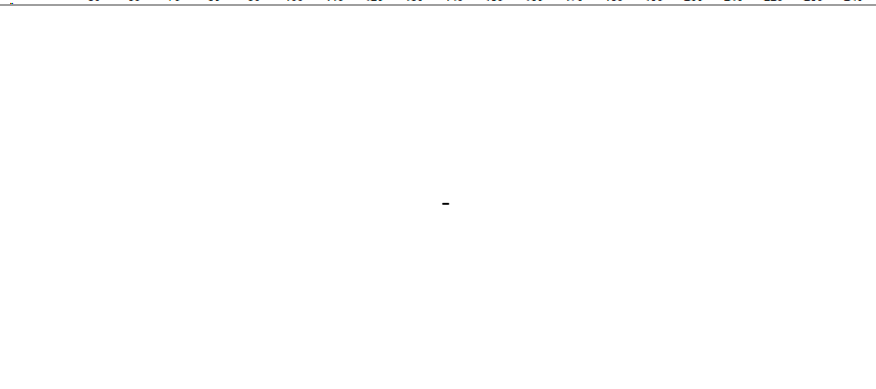

272.15857

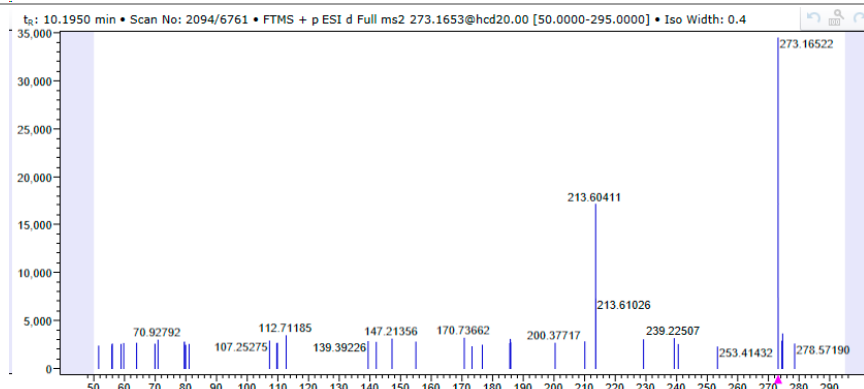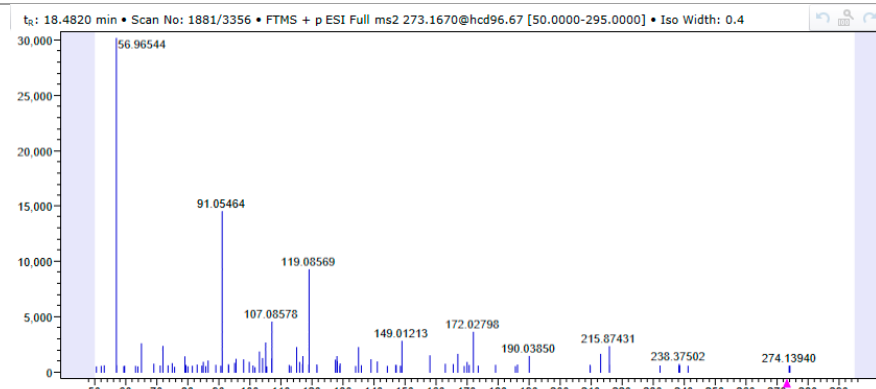

280.12957

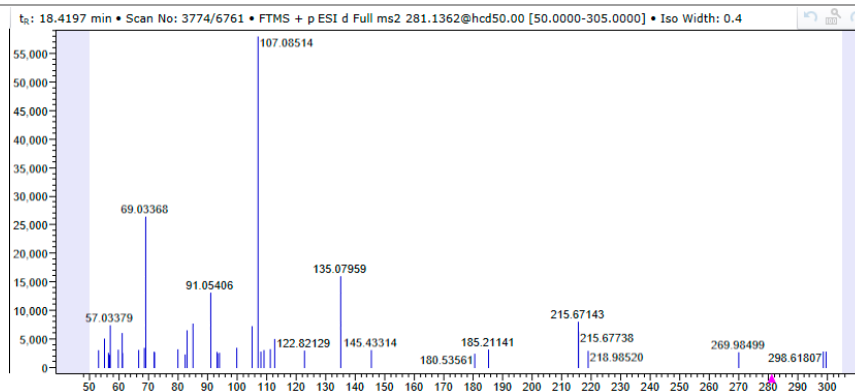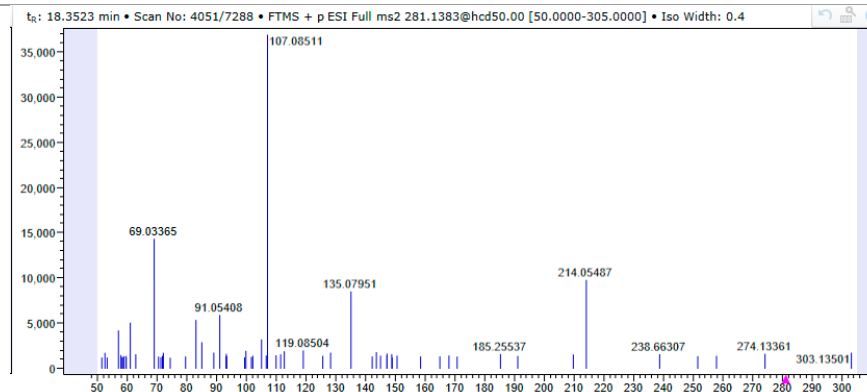

282.10018

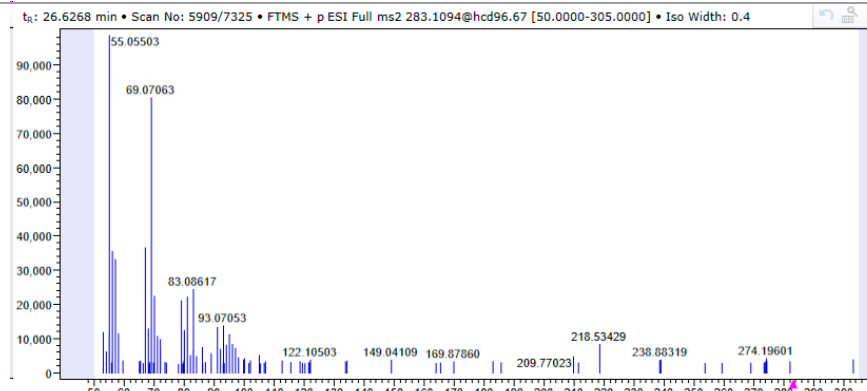

285.13511

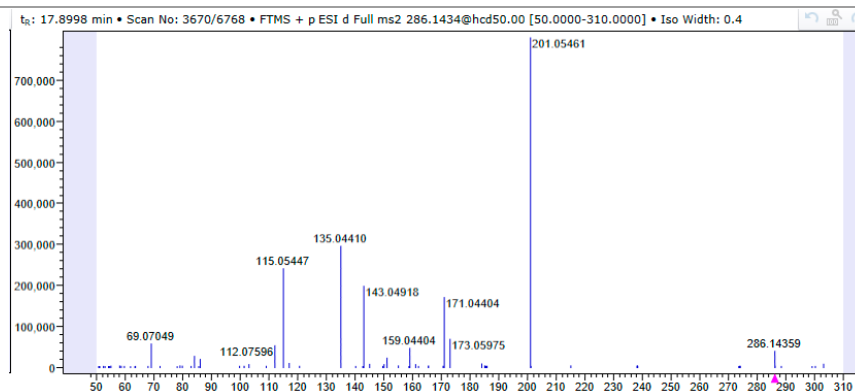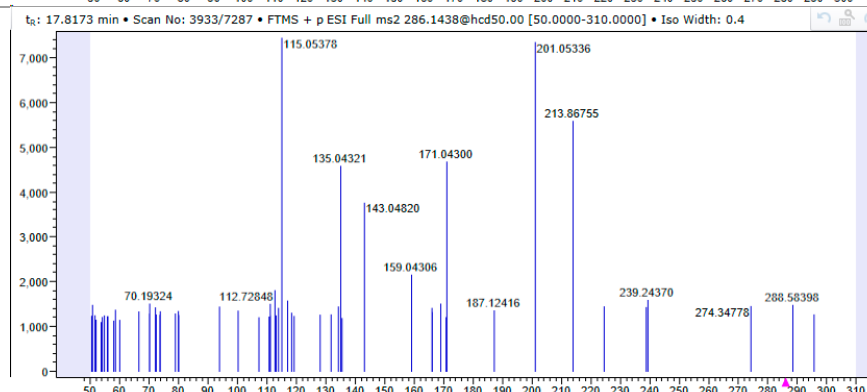

295.24900

-

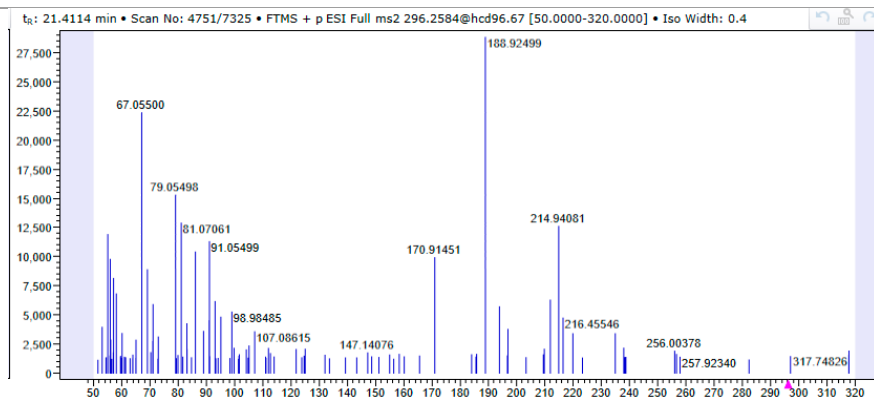

306.23906

-

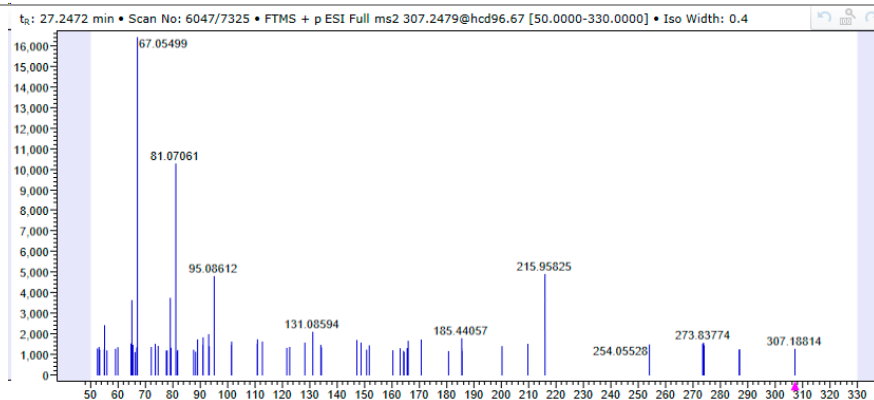

319.24639

-

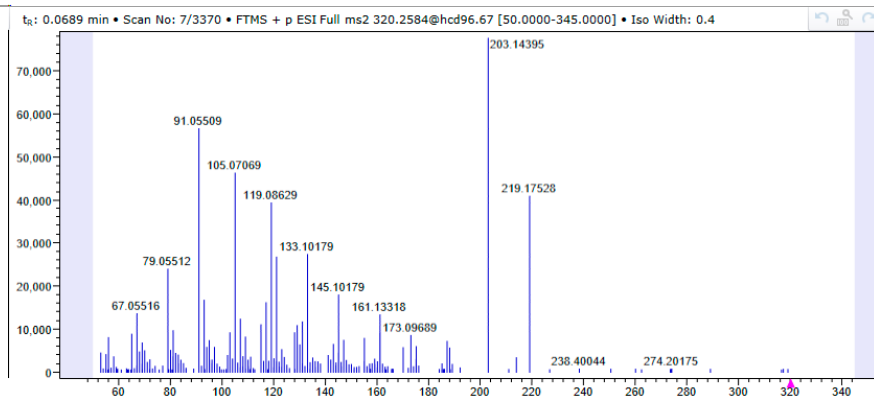

326.32814

-

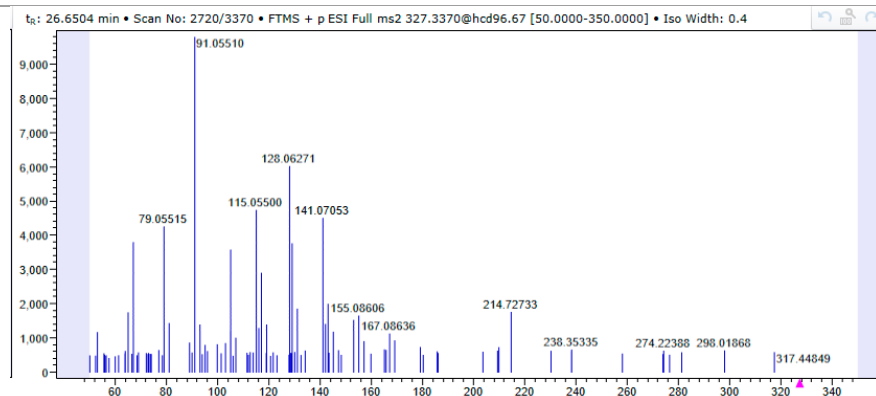

367.41679

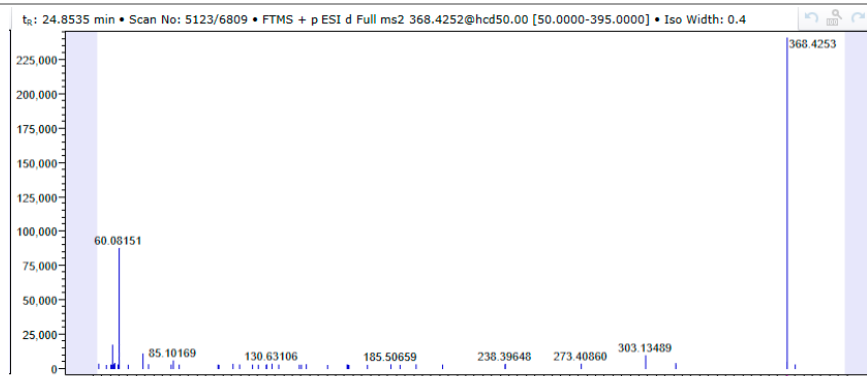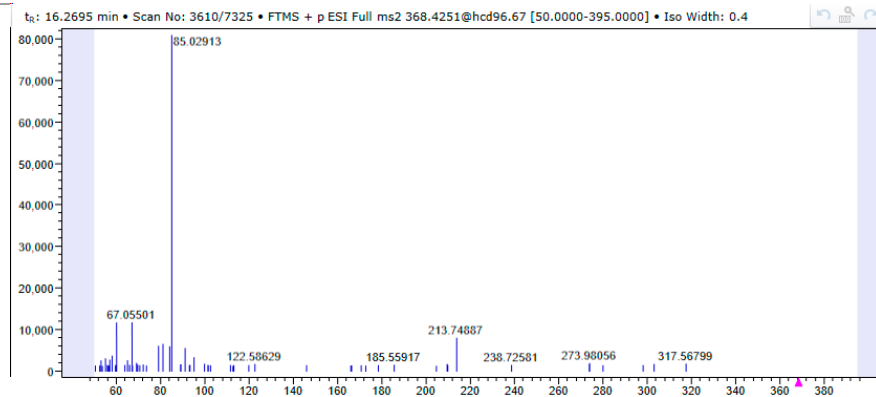

383.19403

-

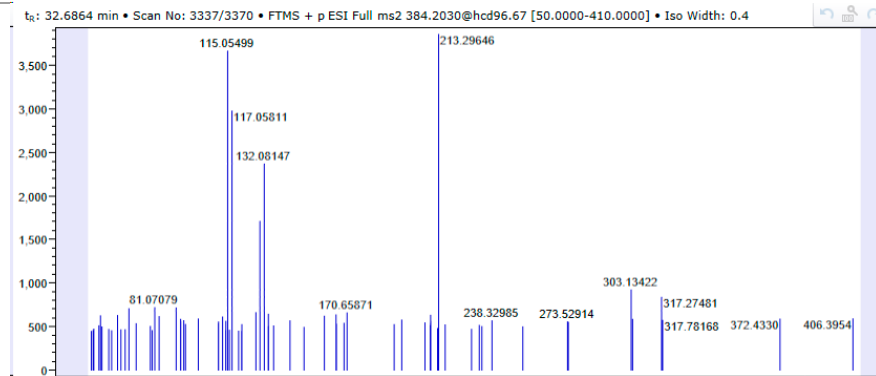

390.27520

-

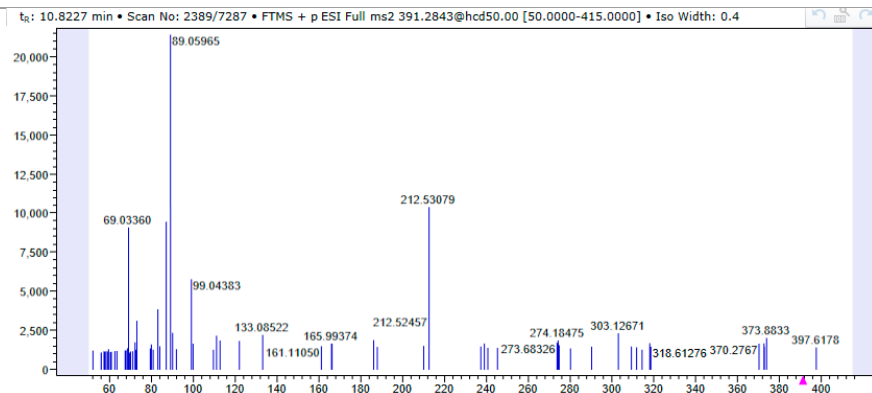

437.49462

-

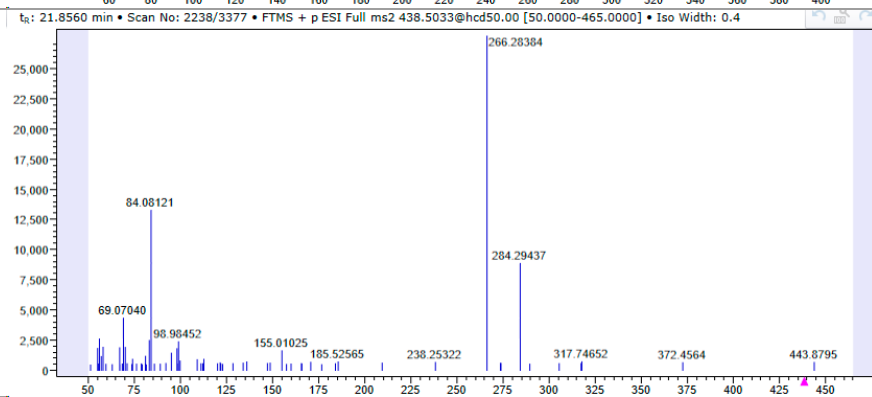

465.52609

-

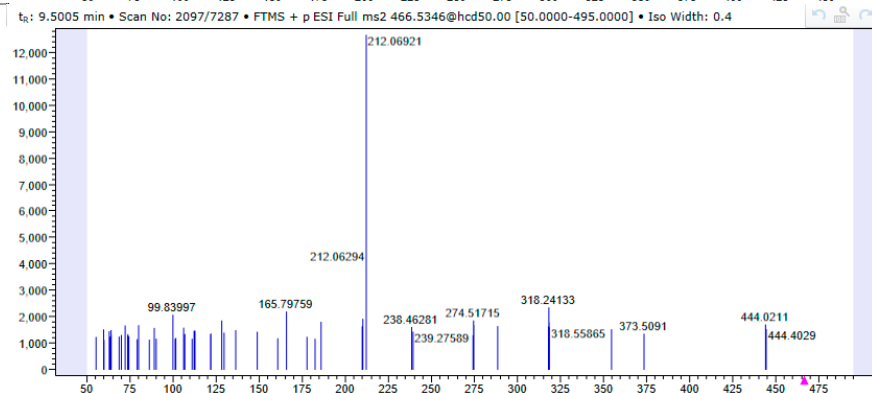

498.89929

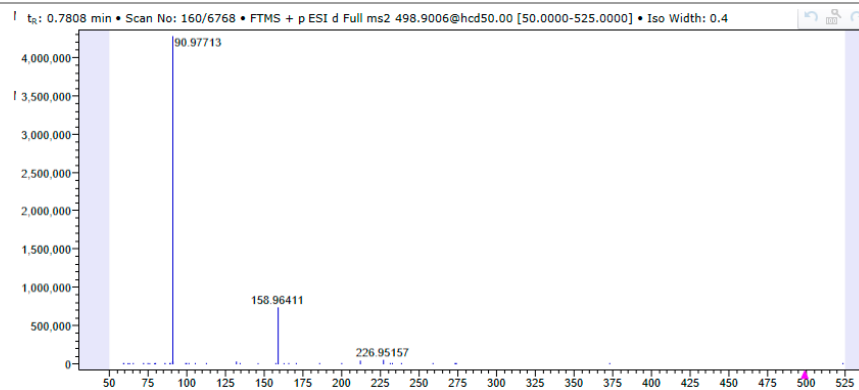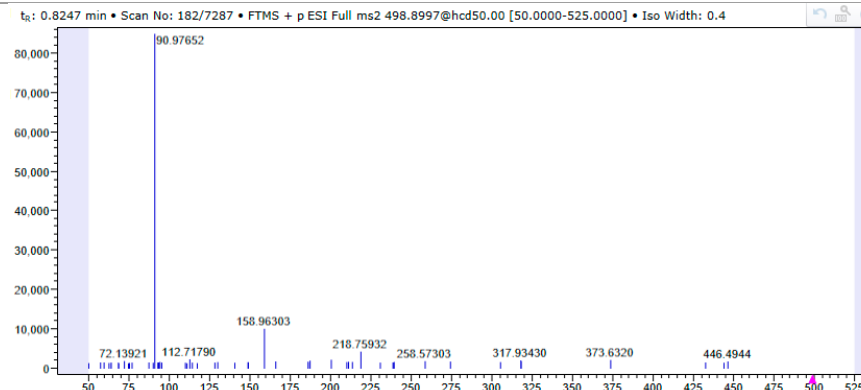

519.33092

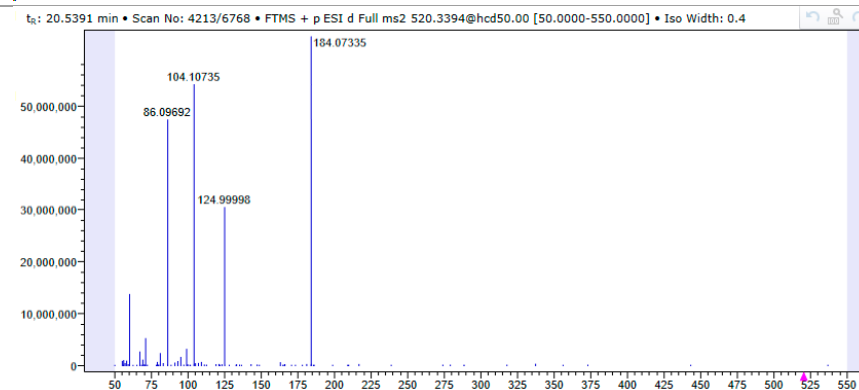

608.21883

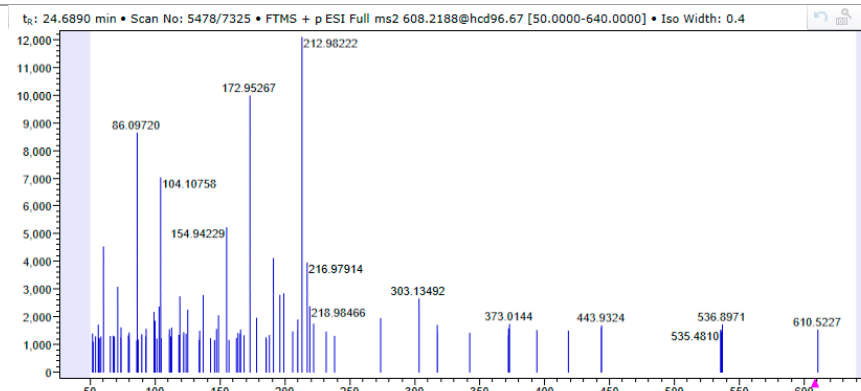

702.86096

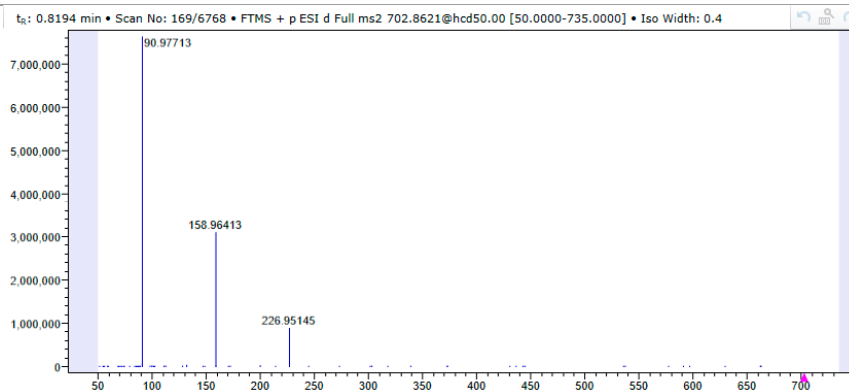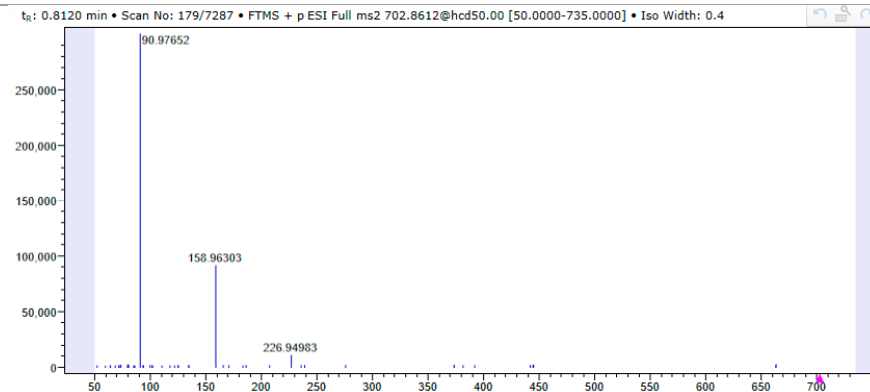

838.83459

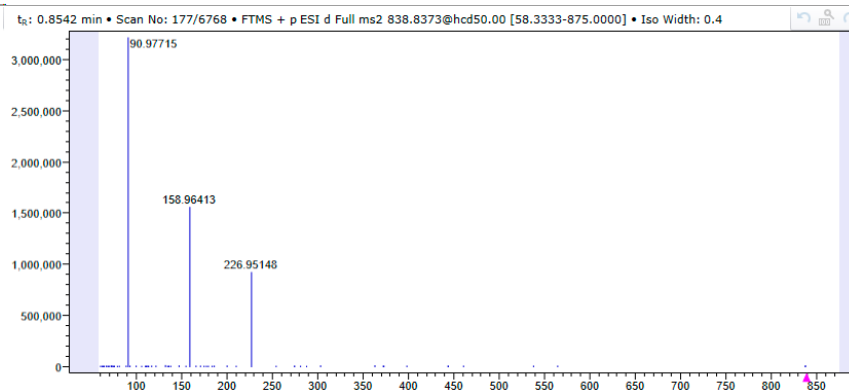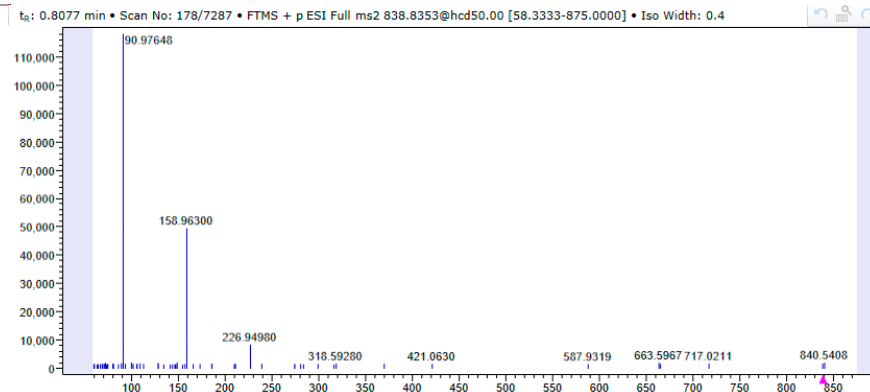

906.82233

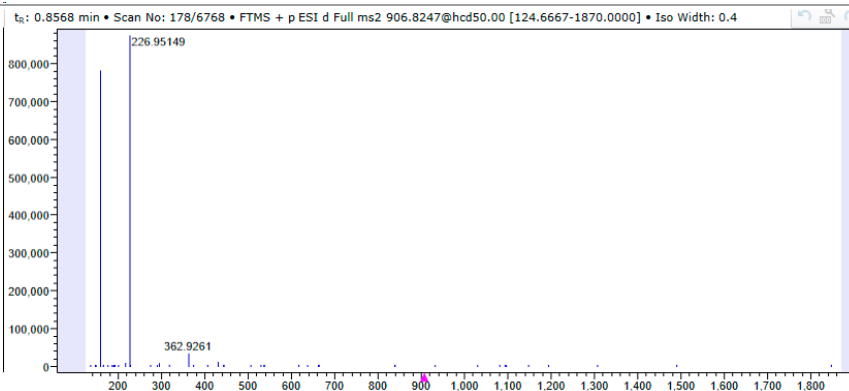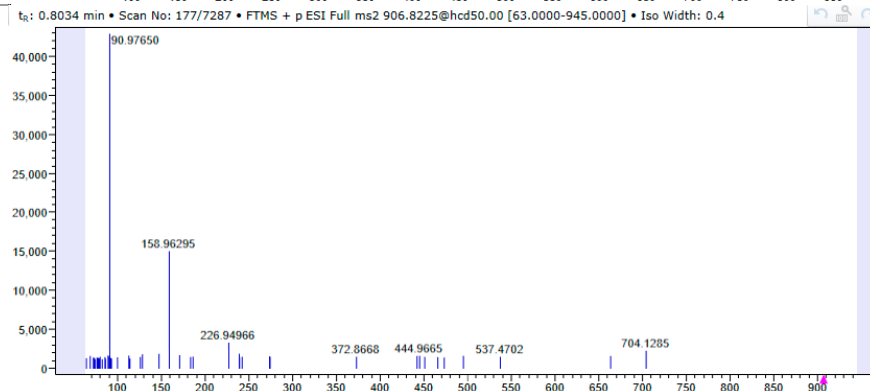

1232.84728

-

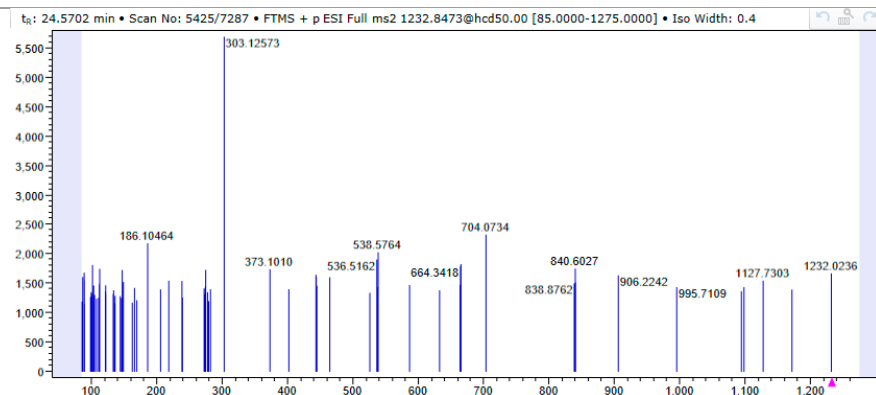

Figure S1. MS/MS spectra obtained by t-Sim and PRM methods.

Supplement: Supplementary file 1 [file biomedicines-14-00872-s001.zip › Fig. S1.pdf]
